# Application of MLP neural network to predict X-ray spectrum from tube voltage, filter material, and filter thickness used in medical imaging systems

**DOI:** 10.1371/journal.pone.0294080

**Published:** 2023-12-07

**Authors:** Jie He, Cai Zhanjian, Jiadi Zheng, Mao Shentong, Mohammad Sh. Daoud, Zhang Hongyu, Ehsan Eftekhari-Zadeh, Xu Guoqiang

**Affiliations:** 1 The First People’s Hospital of Fuyang, Hangzhou, China; 2 Vasculocardiology Department, The Third People’s Hospital of Hangzhou, Hangzhou, China; 3 Wenzhou Hospital of Traditional Chinese Medicine Affiliated to Zhejiang University of Chinese Medicine, Wenzhou, China; 4 Wen Zhou Medical University, Wenzhou, China; 5 College of Engineering, Al Ain University, Abu Dhabi, United Arab Emirates; 6 Shanghai Songjiang District Central Hospital, Shanghai, China; 7 Institute of Optics and Quantum Electronics, Abbe Center of Photonics, Friedrich Schiller University Jena, Jena, Germany; 8 Department of Neurology, Yongkang First People’s Hospital, Yongkang, China; University of Kragujevac, SERBIA

## Abstract

The X-ray energy spectrum is crucial for image quality and dosage assessment in mammography, radiography, fluoroscopy, and CT which are frequently used for the diagnosis of many diseases including but not limited to patients with cardiovascular and cerebrovascular diseases. X-ray tubes have an electron filament (cathode), a tungsten/rubidium target (anode) oriented at an angle, and a metal filter (aluminum, beryllium, etc.) that may be placed in front of an exit window. When cathode electrons meet the anode, they generate X-rays with varied energies, creating a spectrum from zero to the electrons’ greatest energy. In general, the energy spectrum of X-rays depends on the electron beam’s energy (tube voltage), target angle, material, filter thickness, etc. Thus, each imaging system’s X-ray energy spectrum is unique to its tubes. The primary goal of the current study is to develop a clever method for quickly estimating the X-ray energy spectrum for a variety of tube voltages, filter materials, and filter thickness using a small number of unique spectra. In this investigation, two distinct filters made of beryllium and aluminum with thicknesses of 0.4, 0.8, 1.2, 1.6, and 2 mm were employed to obtain certain limited X-ray spectra for tube voltages of 20, 30, 40, 50, 60, 80, 100, 130, and 150 kV. The three inputs of 150 Multilayer Perceptron (MLP) neural networks were tube voltage, filter type, and filter thickness to forecast the X-ray spectra point by point. After training, the MLP neural networks could predict the X-ray spectra for tubes with voltages between 20 and 150 kV and two distinct filters made of aluminum and beryllium with thicknesses between 0 and 2 mm. The presented methodology can be used as a suitable, fast, accurate and reliable alternative method for predicting X-ray spectrum in medical applications. Although a technique was put out in this work for a particular system that was the subject of Monte Carlo simulations, it may be applied to any genuine system.

## 1. Introduction

The X-ray energy spectrum has several practical and medical physics applications. Novel computed tomography (CT) reconstruction approaches based on discrete tomography rely on an understanding of the X-ray energy spectrum in order to mitigate beam hardening effects, calculate scattering photons during X-ray imaging, etc. The medical physics profession makes extensive use of the X-ray energy spectrum for a number of purposes, including numerical evaluation of image quality and x-ray dose computation in procedures including mammography, radiography, fluoroscopy, and computed tomography (CT). Interest in modeling and/or explaining the X-ray energy spectrum of a modern X-ray machine is strongly influenced by its intended function. In X-ray micro-CT applications, knowing the X-ray energy spectrum may be used to correct errors such beam hardening [1] and scatter artifacts [2, 3] that are caused by the interaction of photons with the material being scanned. It has been suggested that knowing the spectrum of an X-ray tube may aid in the elimination of artifacts brought about by flat-field correction [4] and the identification of different types of materials [5]. It is also necessary to know the X-ray source’s spectrum in order to do certain tasks, such as (1) polychromatic CT reconstructions [6, 7], (2) replicating a CT system in a physical simulator to investigate certain elements of photon interactions with the object [8, 9], and (3) estimating the X-ray dose [10]. In diagnostic radiology imaging systems, the computer-simulated x-ray spectrum is one of the most useful instruments for analyzing patient dose and image quality. One of the pioneers in this field was Kramers [11], who attempted to foretell diagnostic x-ray spectra. Numerous researchers have followed this pioneering work, and many groups are presently exploring for an accurate technique to mimic x-ray spectra on computers since their actual measurement needs specialized equipment that is only accessible in a small number of facilities [12–14]. Several x-ray spectra were acquired by Fewell et al. and published [15] using different target/filter combinations. Since measuring x-ray spectra experimentally is tedious and time consuming, many methods have been devised to make educated guesses about them. The three most common varieties are (in order of prevalence) empirical [16], semi-empirical [17], and Monte Carlo [18, 19]. The present models available have drawbacks that prevent their broad use [20], but fully empirical and semi-empirical models are still the quickest approaches for predicting x-ray spectra. Most of these models have fixed target/filter configurations, making it impossible to study how different material compositions might affect subsequent x-ray spectra. Among the packages based on the analytical methods, SpekCalc [21], SPEKTR (TASMICS) [22] are the most powerful and famous ones. However, compared to the proposed method in the present investigation, the aforementioned packages have some disadvantages: A) As mentioned in the abstract of this manuscript, each imaging system’s X-ray energy spectrum is specific and determined by its tubes’ characteristics, while these packages can generate some general spectra. Using the proposed method in the present work, a more precise spectrum can be achieved; B) SpekCalc package produces X-ray spectrum based on the analytical method which are not as accurate as those obtained from Monte Carlo techniques (the authors of SpekCalc package [21] also confirmed that Monte Carlo technique is a gold standard for X-ray generation); C) SpekCalc cannot generate first 10% of energy spectrum. For example, if tube voltage of 140 kV is selected, it can generate spectrum in the energy range of 14 keV-140 keV; D) SpekCalc is not a free source package and is not available to everyone.

Complex Monte Carlo modelling was used as a means of overcoming these limitations. Since their introduction, Monte Carlo radiation transport models have quickly established the gold standard for predicting x-ray spectra, even in highly non-trivial geometries [23]. Monte Carlo simulation has been used by several researchers in recent years to calculate X-ray energy spectra [24–27]. Direct experimental measurements of the X-ray spectrum are difficult and can only be made at a few number of research institutions using expensive and specialized equipment. In addition, calculating the X-ray spectra with great accuracy using the Monte Carlo method is a laborious process. It takes a typical desktop or laptop computer about five days to simulate the generation of a single X-ray spectrum for a given tube voltage, target angle, and metal filter. Naturally, the time of simulation is highly dependent on the tube voltage, and as the tube voltage increases, so does the time needed to simulate. It should be noted that the configuration of the PC used in this research is as follows: Processor intel Corr(TM) i7-10750, RAM16GB,GPU 4GB 1660Ti.

In [28], a humanoid phantom was photographed using a mobile two-dimensional C-arm fluoroscopy system, a robotic ceiling-mounted hybrid OR C-arm cone beam CT (hCBCT), and a mobile O-arm CBCT. Scatter doses were measured using active personal dosimeters and an ionization chamber at several locations in the hybrid operating room. Two distinct RPSs were studied. Compared to oCBCT, the average radiation exposure to workers was decreased by 22% when hCBCT was used. Scatter doses from the hCBCT were 27% lower compared to the oCBCT at a distance of 200 cm from the phantom. One hCBCT or oCBCT rotational acquisition was equivalent to 12 or 16 minutes of C-arm fluoroscopy, respectively. Behind an RPS, the scatter dosage dropped by more than 90%. However, at 60 cm behind the RPS, the protection was marginally diminished owing to tertiary scatter from the environment. The angular distribution of the bremsstrahlung generation has often been assumed to be spherically uniform in theoretical models of the X-ray spectrum. This hypothesis is based on the idea that the inherent bremsstrahlung angular distribution is effectively masked by the rapid acquisition of a diffuse directional distribution by electrons in an x-ray target owing to multiple scattering. In [29], a model was proposed that takes into consideration the bremsstrahlung production’s angular distribution. Incorporating theoretical conclusions for the differential bremsstrahlung cross section, the model combines Monte Carlo-calculated depth, energy, and angular distributions of electrons entering the x-ray target. For tungsten and molybdenum targets in the 20–300 keV energy range, the impacts of employing various simplified model assumptions for the electron penetration and the inherent bremsstrahlung angular distribution were examined. In addition, a full x-ray emission model (including bremsstrahlung and characteristic x-ray emission) over the energy range of 20–300 kV was given and verified by the author in [30]. The objective of the study conducted by [31] was to create and provide x-ray spectra for a selection of commonly used digital mammography (DM), breast tomosynthesis (BT), and breast CT (bCT) systems in the North American region. The study used the Monte Carlo algorithm MCNP6 to conduct simulations of minimally filtered x-ray spectra, specifically using beryllium as the only filtering material. The simulations were conducted at eight different tube potentials ranging from 20 to 49 kV for the DM/BT scenario, and at nine tube potentials ranging from 35 to 70 kV for the bCT scenario. The simulation included the examination of anode compositions particular to vendors, determination of optimal anode angles, assessment of focused spot sizes, analysis of source-to-detector distances, and evaluation of beryllium filtering. at order to get spectra at 1 kV intervals, the fluence for each 0.5 keV energy bin in all simulated spectra was interpolated using cubic splines. This interpolation was performed throughout the range of simulated tube potentials. For the DM/BT spectra, the tube potentials ranged from 20 to 49 kV, while for the bCT spectra, the tube potentials ranged from 35 to 70 kV. Attempts to calculate the X-ray spectrum using an radial basic function network (RBF) network were performed by the authors of [32]. To anticipate the X-ray spectra point by point, they developed many RBF neural networks that all operated concurrently. Some X-ray spectra, such as those produced from an aluminum filter with a tube voltage of 20 kV and from a beryllium filter with a tube voltage of 50 kV, are not predictable, according to their findings utilizing the RBF neural network. Referenced research [33] explored the use of the Monte Carlo N Particle eXtended (MCNPX) algorithm to simulate a whole industrial X-ray digital radiographic system, including an X-ray tube and a computed radiography (CR) picture plate. An industrial X-ray tube operating at 300 kV and 5 mA was modeled as part of their investigation. Moreover, the CR imaging plate was described and modeled as a uniform three-layer plate consisting of a polymer topcoat layer, a phosphor layer, and a polycarbonate backing layer. In order to mimic the process of image generation on the image plate, the absorbed dosage in each pixel within the phosphor layer of the CR imaging plate was first estimated using the mesh tally in the MCNPX algorithm. Two step wedges were fabricated from aluminum and steel, and their pictures were acquired by the tests and compared to the simulations in order to verify the simulation findings. As can be seen from the findings, the suggested approach is capable of replicating an industrial X-ray imaging system, and the simulated pictures are in excellent agreement with the real ones. In [34], the X-ray energy spectrum of FleXCT, a revolutionary prototype industrial micro-CT scanner, was calculated using Monte Carlo simulations to allow for beam hardening and scatter reduction during CT testing. Monte Carlo simulation was used to fully simulate the source and the detector. The Monte Carlo simulation energy spectra were checked against a second set of spectra produced in a similar fashion. Measurements were taken, and then the Maximum Likelihood Expectation Maximization (MLEM) approach was used to derive an energy spectrum. Both methods showed high levels of correlation, proving that FleXCT could be accurately modeled using Monte Carlo analysis. Modeling and calculating the dose to a homogeneous phantom are presented in study [35], which helps to ensure the accuracy of X-ray tube measurement data. As part of the X-ray tube’s quality control, the parameterized X-ray spectrum was utilized to check the air kerma against theoretical predictions. The SPECTRUM X-ray spectrum was compared to two commercially available spectra, SpekCalc and Institute of Physics and Engineering in Medicine (IPEM) 78, to confirm the suggested approach. The PCXMC-calculated imparted fraction was compared to the fraction of energy that was actually transferred to the homogeneous phantom.

The primary goal of the present research is to suggest an intelligent method based on artificial intelligence for more quickly and accurately computing the X-ray energy spectra of each unique medical imaging system based on the restricted set of spectra. Although the suggested approach was used in this research for a particular system, it may be used for any system with its own unique features. The restricted spectrum needed for neural network training could be produced using either experimental techniques or the Monte Carlo simulation methodology, which was applied in the current investigation. This research’s initial step is to show and simulate the X-ray tube’s construction using the Monte Carlo algorithm. This part looked at various tube voltages, the type of filter used, and the thickness of the filter. The result was a collection of 99 X-ray spectra. Although the received spectra have to be determined using lengthy Monte Carlo computations, it is imperative to utilize these spectra to train neural networks to identify X-ray tube spectra from a small set of spectra. The MLP neural network will be thoroughly detailed in the next section. A description of the MLP neural network design procedure for X-ray spectrum prediction will also be provided. The justifications for the proposed approach of forecasting the X-ray spectrum and the new system’s precision are covered in the results stage. The last section contains the conclusion.

## 2. Simulation configuration

**Fig 1:**
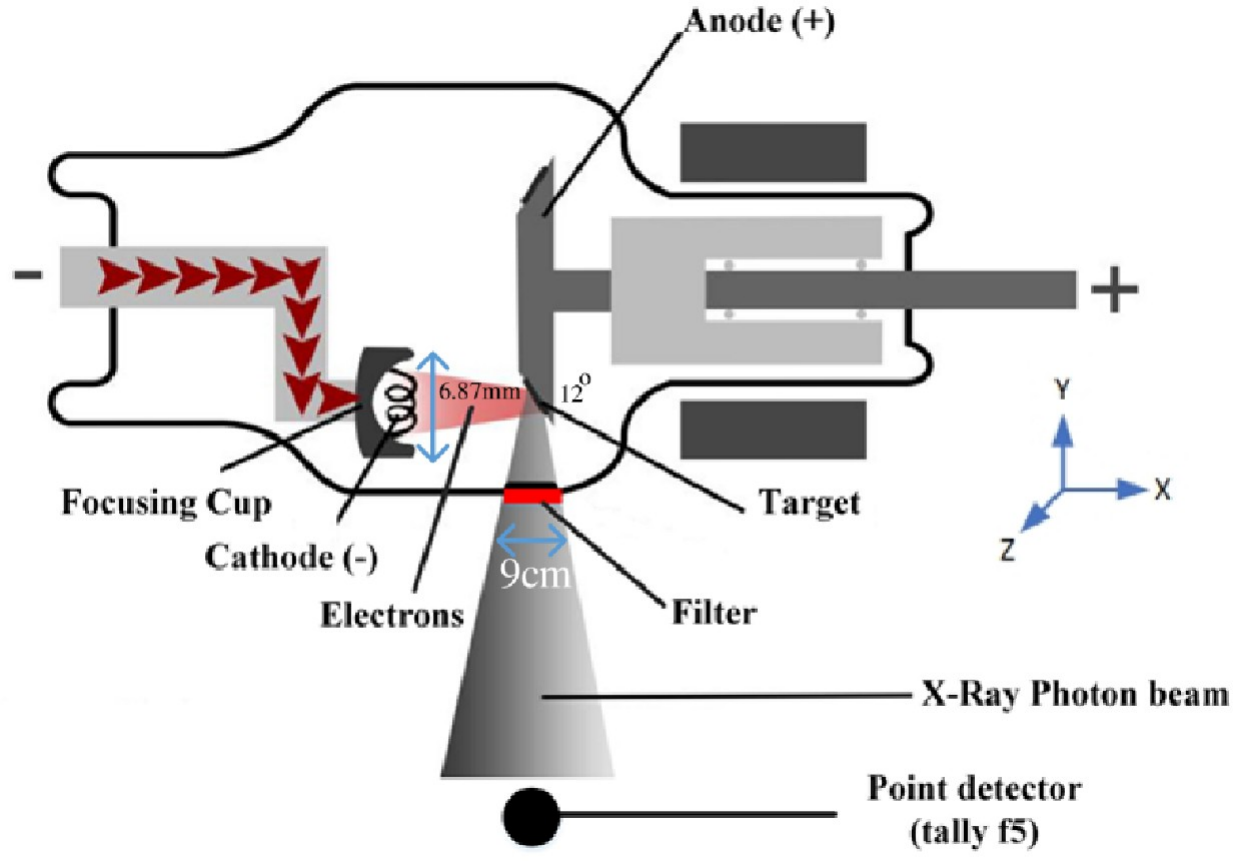
Schematic cross-sectional view of the simulated X-ray tube.

The MCNP algorithm, version X [36], is the basis of the Monte Carlo model used to simulate the interaction of neutrons, photons, electrons, and the combined neutron/photon/electron particles with matter. Radiography, medical physics, radiation protection, dosimetry, radiation shielding, and maybe even nuclear medicine might help all gain from this. The MCNP code is a powerful tool for modelling the medical X-ray imaging machines. The X-ray tube consists of many individual parts, including the electron source, target, output window, shield, and filter. The modelled geometry used in the simulation, can be seen in Fig 1. The angle of the tungsten anode (target) in respect to the vertical axis of the tube, was selected 12 degrees. To model focal spot of X-ray tube (as specified by the IEC 60336 standard), the electron source was defined as a two-dimensional rectangular planar source with uniform weighting in the y-z plane [37]. This rectangular source’s y-axis length was 6.87 mm, while the z-axis width was settled on being 2.5 mm. The focal spot projected by the aforementioned electron source is 1.02mm ×1mm on the target. Accordingly, the effective focal spot is 1.06mm ×1mm on the imaging plane or plane perpendicular to the x-z axis. The emitted electrons have a direction parallel to the x-axis; hence the source is not isotropic. The sphere-shaped output window on the tube shield measures 4.5 centimeters in diameter. A point detector was placed after the X-ray tube’s window to determine the X-ray energy spectrum (tally type F5).

In the present simulations, photon and electron physics cards (PHYS command) were used. In order to reduce the Monte Carlo computation time, the CUTOFF card with energy value of 0.5 keV (cut:e j 0.0005) was used for electrons. The electrons with energy less than 0.5 keV don’t have a significant contribution to the tally results, but tracking these electrons up to energies near 0 keV, would increase the computation time. It worth mentioning that CUTOFF command is usually implemented in MCNP to specify a minimum energy below which the particle is killed. In addition, the statistical uncertainty was kept below 0.5% using the TALYY F5 STOP card (STOP F5 0.005) for all the simulations.

There were 99 computed X-ray spectra used to train the neural network, each calculated under a unique set of parameters such as a distinct tube voltage (20, 30, 40, 50, 60, 80, 100, 130, and 150kV), filter material (no filter, beryllium, and aluminum), and filter thickness (0.4, 0.8, 1.2, 1.6, and 2mm). In the previous research [33], the X-ray spectra obtained from the MCNP code were compared with the X-ray spectra obtained from the experimental structure and also with other software packages. It was observed the results were in good agreement with each other.

## 3. MLP neural network

The human brain is a collection of millions of computing units called neurons, which are associated with one another. Neurons contain branches that are called dendrites, which are the way through which information is sent. Following completion of the processing stages, the information is then sent down the axon to be sent outside of the neuron. The processes that were discussed take place in the physiological and biochemical domains; nevertheless, inspired by this behavior, its mathematical model was extracted. The MLP neural network is one of the models that is used the most often. There are three distinct components that make up the architecture of this network: the output layer component, the hidden layer component, and the input layer component. There may be more than one hidden layer under the surface. Activation functions are the names given to the mathematical operations that are carried out in the layers that are concealed. The kind and level of non-linearity of the data that is supplied determines the number of these layers, as well as the number of neurons in the hidden layer and the type of activation function that is used [38, 39].

The available data are separated into three groups- training, validation, and testing- to address the issues of over-training and under-training. The bulk of the information the neural network needs to recognize and tailor its representations to is found in the training data. Commonly, when people talk about "validation data," they mean a subset of the dataset that is used to check if the training is accurate. These records are used for the validation of the network throughout the training phase. In order to guarantee a precise performance, the test data are applied to the neural network as a last step in the training process. When a neural network is correctly trained and deployed, it excels on all three of the new data categories. MATLAB was used to train and evaluate the MLP neural network used in this investigation. The techniques for training, validating, and testing neural networks have been painstakingly designed in this study to offer for maximum flexibility, even if the software contains several pre-built toolboxes for building certain neural networks. It should be mentioned that the neural network was trained using the MATLAB ’newff’ function. It was discovered that a network structure with one hidden layer and 10 neurons in the hidden layer provided the most accurate estimates of the intensity of the X-ray spectrum in the specified energy after the deployment of several neural networks with varying numbers of hidden layers and neurons in each hidden layer.

## 4. Results and discussion

**Fig 2:**
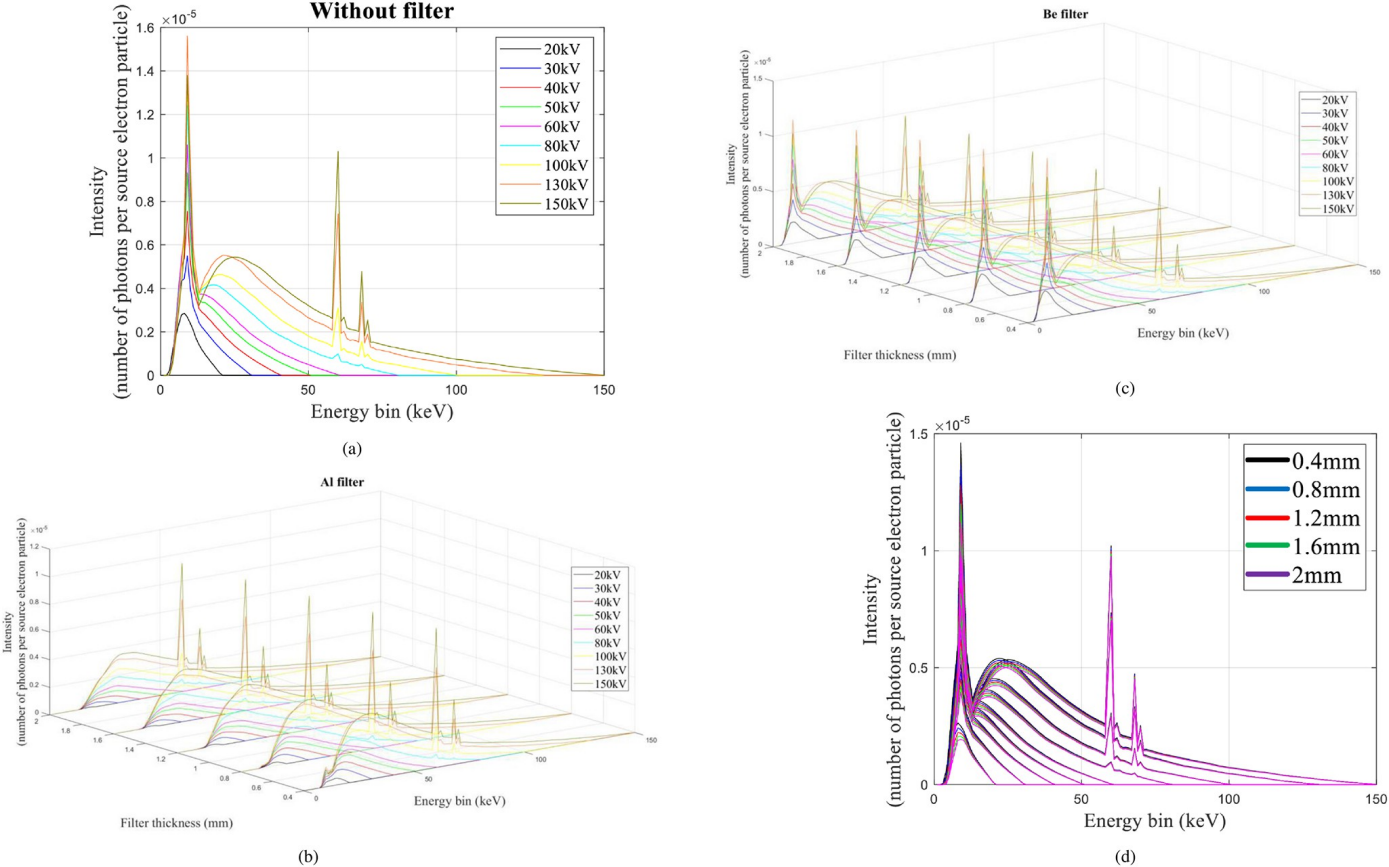
Recorded spectrum of the X-ray tube for three different filter types: a) no filter, b) aluminum filter, c) beryllium filter, and d) different view of beryllium filter.

**Fig 3:**
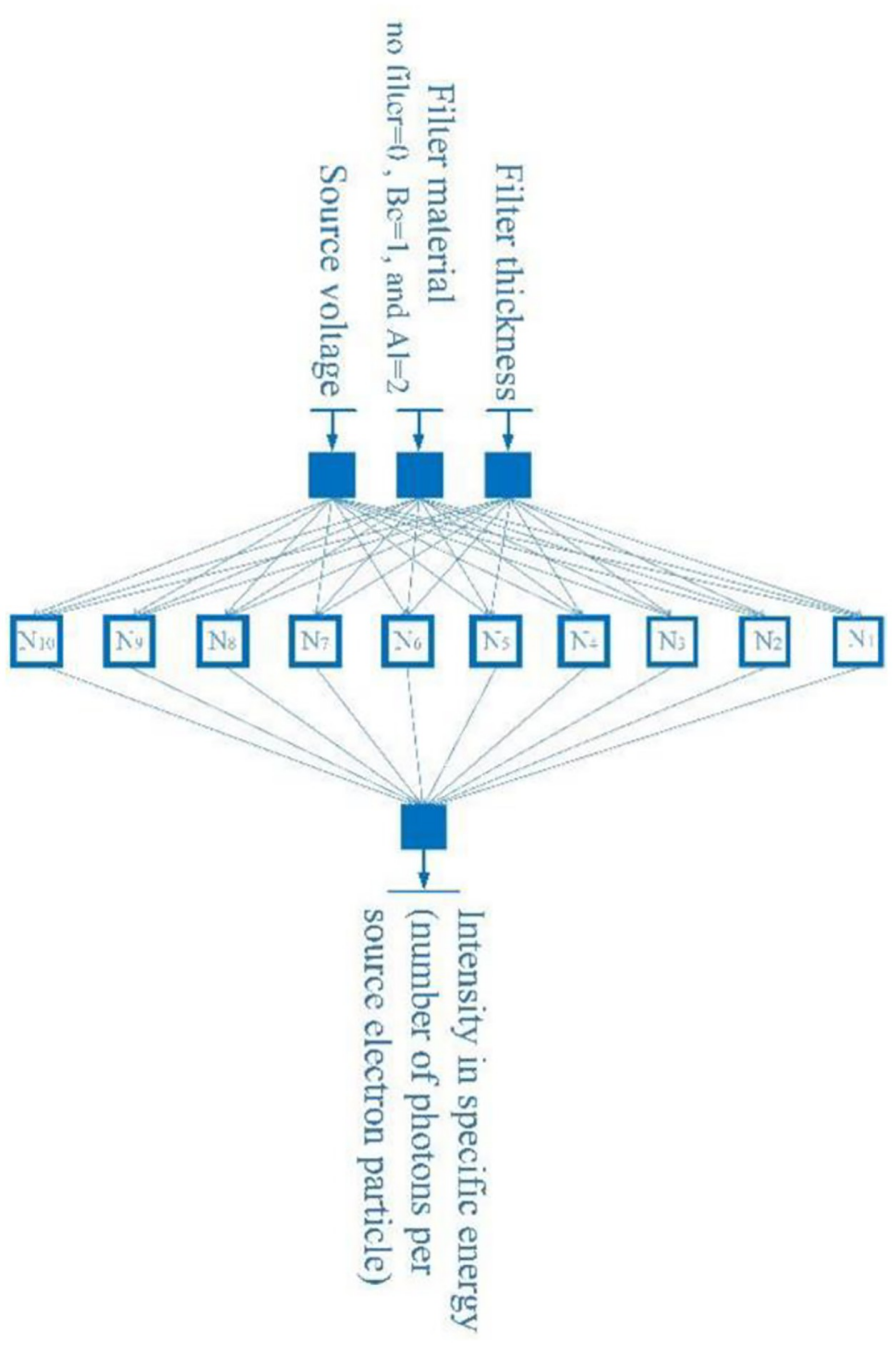
Implemented MLP neural network.

**Fig 4:**
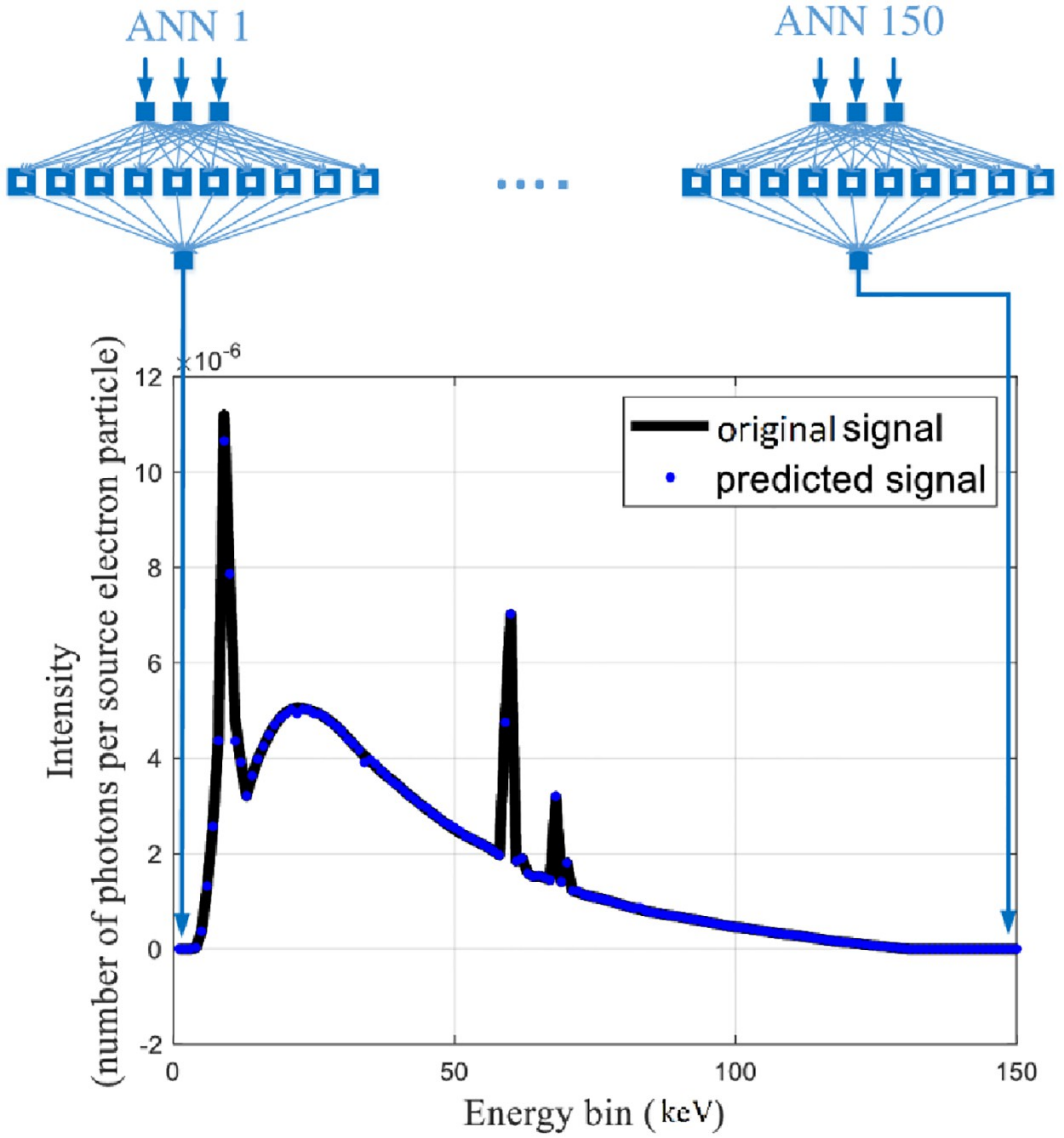
X-ray spectrum prediction process.

**Fig 5:**
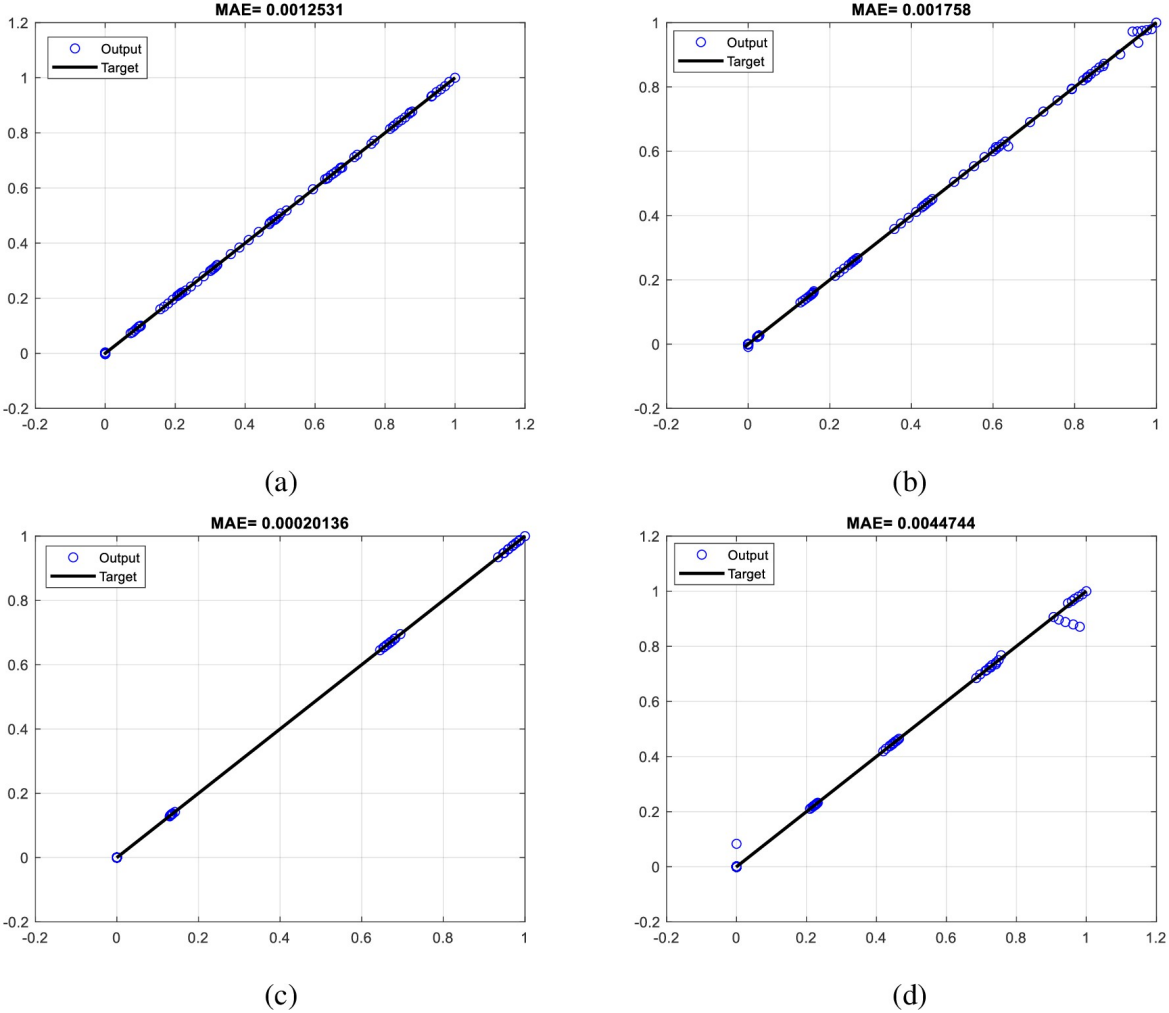
Regression diagrams of network number a) 28, b) 39, c) 62, and d) 95.

**Fig 6:**
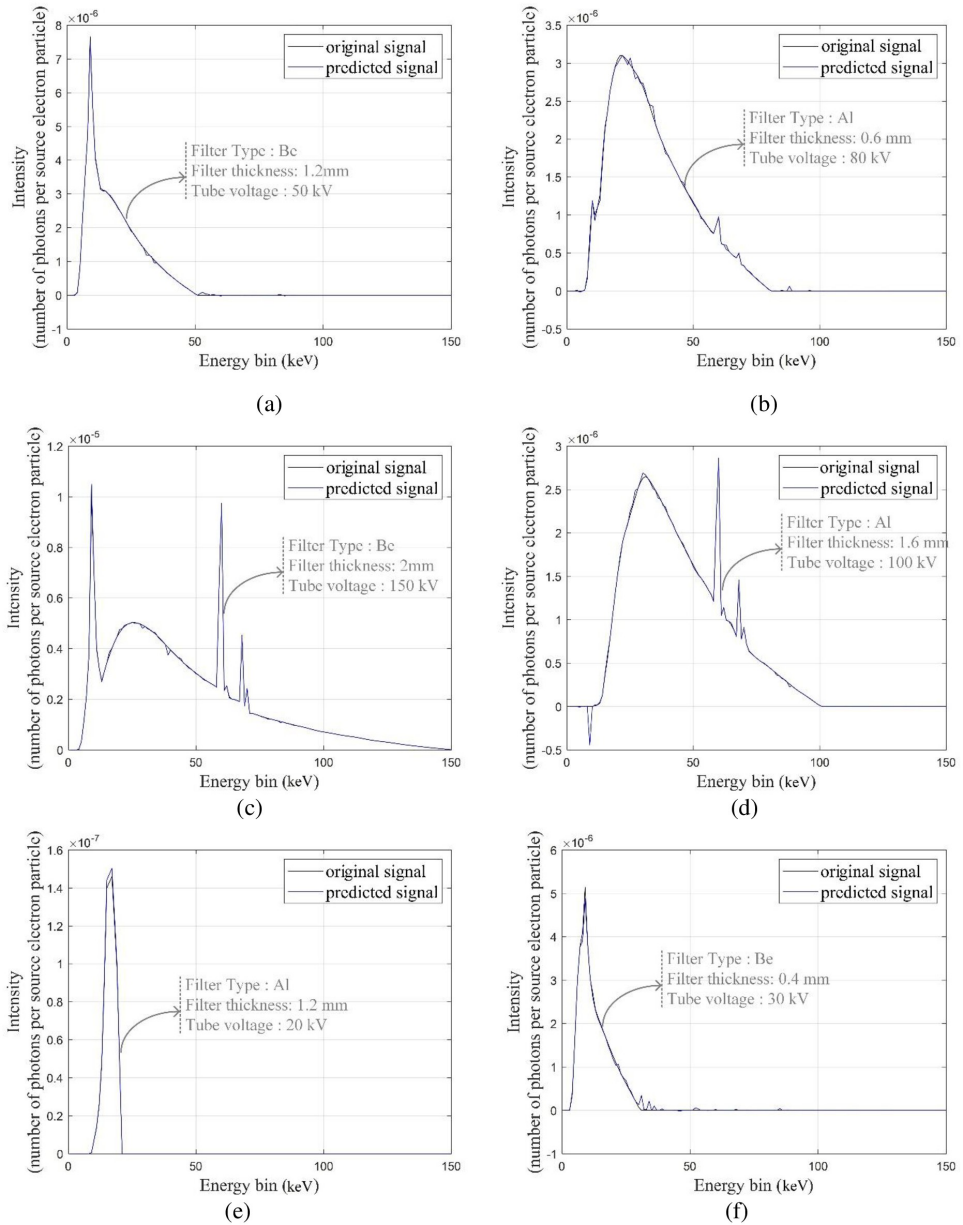
Comparison of spectra reconstructed by MLP neural network and the original spectrum, a) Be filter with thickness of 1.2 mm and tube voltage of 50kV, b) Al filter with thickness of 0.6 mm and tube voltage of 80kV, c) Be filter with thickness of 2 mm and tube voltage of 150kV, d) Al filter with thickness of 1.6 mm and tube voltage of 100kV, e) Al filter with thickness of 1.2 mm and tube voltage of 20kV, and f) Be filter with thickness of 0.4 mm and tube voltage of 30kV.

**Fig 7:**
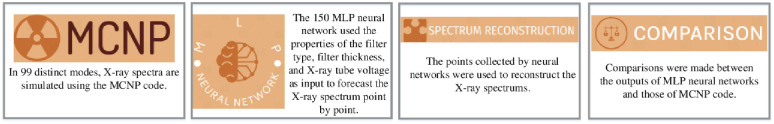
X-ray spectrum prediction steps.

Fig 2 displays the associated X-ray spectrum. Intensity is shown as the number of photons per source electron particle along the z-axis, while the x-axis indicates energy bins in keV. Y-axis shows the thickness of the filter in mm. The figure clearly shows that the attenuation value rises with filter thickness, and that the attenuation value is more pronounced in the low-energy portion of the spectrum than in the high-energy section. The difference between the spectra in Fig 2c is very small. Another view of this figure is shown in Fig 2d. The methodology presented in this research is that every point of the X-ray tube spectrum is predicted using an MLP neural network. What is meant by each point is the intensity value of the spectrum, in terms of counting per source electron particle, at each energy bin. As shown in Fig 2, the received spectra have an energy bin of between 1 and 150; therefore, 150 neural networks have been trained to predict the spectrum. The three characteristics of tube voltage, filter thickness, and filter type were inputs of neural networks. The three tube features described above were taken into consideration as inputs by 150 neural networks from the 99 spectrums obtained from the simulations. About 70% of the data (69 samples) and 15% (15 samples) from this matrix were utilized to create the neural network, with the remaining samples being used for network testing. The structure of trained MLP networks can be seen in Fig 3. This network has one hidden layer and ten neurons in the hidden layer. The activation function used in hidden layer neurons is the Tansig type. The details of the designed networks are tabulated in Table 1. Several neural networks were implemented with different configurations, including varied numbers of hidden layers and neurons inside those hidden layers, with the aim of accurately determining the spectrum of X-rays. In conclusion, empirical evidence has shown that a neural network including just one hidden layer and ten hidden neurons has the capability to effectively forecast the data points inside the X-ray spectrum. Fig 4 depicts the overarching procedure for reconstructing an X-ray spectrum. Mean Absolute Error (MAE) is an error criteria that was used to describe the accuracy of the neural networks; The highest value of MAE among all neural networks and all training, validation and testing groups is equal to 0.044. Fig 5 is a regression diagram showing how well some of these networks function. In this figure, the black line is the data obtained from the MCNP code, and the blue circles are the output of the neural network, which were defined as the target and output data, respectively. Neural networks are correctly trained only when their inputs and outputs are normalized and converted to the range of 0 to 1. It is important to remember that the outputs were reapplied to the seed state after training the neural networks. Following the development and training of the neural networks, the anticipated spectrum was rebuilt and compared to the original. The implemented networks could predict all X-ray spectra correctly and with high accuracy, while in the previous research [32], not all spectra could be predicted. The comparison of some spectra reconstructed by the MLP neural network and the original spectrum is shown in Fig 6. In this figure, the blue spectrum is the spectrum obtained by the MCNP code, and the black spectrum is the spectrum predicted by the neural network. As it is clear from this figure, the artificial neural network has performed very well in determining the X-ray spectrum. The general steps of this research to predict the X-ray spectrum can be seen in Fig 7. Although the accuracy of the system presented in this research is high, using 150 neural networks has imposed a significant computational burden on the detection system. Although the accuracy of the method presented in this research is high, using of 150 neural networks has a large computational load, which is one of the limitations of the method presented in this research. The procedure involves the prediction of X-ray spectra on a point-by-point basis, leading to a significant computational load. Various properties may be extracted from the X-ray spectrum and used to rebuild the X-ray spectrum, hence reducing the computational load. In essence, the utilization of an artificial neural network enables the possibility of predicting the regeneration attributes as opposed to the X-ray spectrum, afterward using these attributes to reconstruct the initial spectrum, which can be a very attractive topic for researchers in this field. In fact, the main goal of this research is to show the ability of artificial neural networks to reproduce the X-ray spectrum. For this purpose, in this research, as an example, we considered the target angle and filter thickness to be constant. As the results of neural networks show, we have succeeded in presenting a method based on artificial neural networks to reproduce the X-ray spectrum. It should be mentioned that different target angles and filters with different thicknesses can be investigated in future research. In future research, it is even possible to train other neural networks to reproduce the x-ray spectrum independently of the target grade and filter thickness.

**Table 1:** The details of trained MLP networks.

Type of ANNs	MLP
**Input layer neurons**	3
**Hidden layer**	1
**The hidden layer neurons**	10
**Output layer neurons**	1
**Epoch range in neural network training phase**	300–600
**Hidden neuron activation function**	Tansig

## 5. Conclusion

The X-ray energy spectrum is essential for evaluating image quality and determining safe X-ray exposure levels in diagnostic imaging modalities such as mammography, radiography, fluoroscopy, and computed tomography (CT). However, it takes much time to determine the X-ray spectrum using experimental techniques and Monte Carlo code. The current study investigated the potential of artificial neural networks for predicting the X-ray spectrum from X-ray tube characteristics. In order to compile a database of X-ray tube spectra, initial spectra were produced from an X-ray tube using the Monte Carlo algorithm with varying parameters, such as tube voltage (20–150 kV), filter type (no filter, Be, and Al), and filter thickness (0.4–2 mm). These features were applied to the input of 150 MLP neural networks to predict the intensity at all energies. The operation of all the neural networks simultaneously makes the spectrum of X-rays can be determined according to the source voltage, filter type, and filter thickness. All spectra collected by MCNP code were reconstructed with high accuracy. Due to this success, the described technique is defined as a rapid and accurate way to recreate an X-ray spectrum. The suggested technology shows great promise for use in various medical applications. Due to the use of 150 neural networks to predict the X-ray spectrum, the determination system has a high computational load. Researchers can obtain characteristics of the X-ray spectrum that can be used to predict the entire X-ray spectrum. For example, by fitting different polynomials and calculating the coefficients of the polynomials, instead of implementing multiple neural networks to predict the X-ray spectrum point by point, they only predict the coefficients of the polynomials. Using those coefficients, and the defined polynomial, the X-ray spectrum may be reconstructed. This method can greatly help to reduce the amount of calculations applied to the system.

## Supporting information

S1 File (DOCX)
