# Supplementary material for: Application of MLP neural network to predict X-ray spectrum from tube voltage, filter material, and filter thickness used in medical imaging systems
Source: PLoS One. 2023 Dec 7;18(12):e0294080. doi: 10.1371/journal.pone.0294080 (PMC10703281; doi:10.1371/journal.pone.0294080)
Supplement: S1 File — (DOCX) [file pone.0294080.s001.docx]

**Application of MLP neural network to predict X-ray spectrum from tube voltage, filter material, and filter thickness used in medical imaging systems**

Cai Zhanjian^1^ , Jiadi Zheng^2^，Mao Shentong^3^ , Mohammad Sh.Daoud^4^, Zhang Hongyu^5*^ , Ehsan Eftekhari-Zadeh 6^,*^ , Xu Guoqiang ^7,*^

^1^ Vasculocardiology Department, The Third People’s Hospital of Hangzhou, Hangzhou 311115, China, [caizj@ahfahp.com](mailto:caizj@ahfahp.com)

^2^ Wenzhou Hospital of Traditional Chinese Medicine Affiliated to Zhejiang University of Chinese Medicine, Wenzhou 325000, China, [zjiadidi@fyhospital.cn](mailto:zjiadidi@fyhospital.cn)

^3^ Wen Zhou Medical University, Wenzhou 325000, China, [shentongm@ahfahp.com](mailto:shentongm@ahfahp.com)

^4^ College of Engineering, Al Ain University, Abu Dhabi 112612, United Arab Emirates; mohammad.daoud@aau.ac.ae

^5^ Shanghai Songjiang District Central Hospital, Shanghai 200000, China, [hongyu_zhang@ahfahp.com](mailto:hongyu_zhang@ahfahp.com)

^6^ Institute of Optics and Quantum Electronics, Abbe Center of Photonics, Friedrich Schiller University Jena, 07743 Jena, Germany; [e.eftekharizadeh@uni-jena.de](mailto:e.eftekharizadeh@uni-jena.de)

^7^ Yongkang First People's Hospital, Department of Neurology, Yongkang 321300, China, guoqiangx89@fyhospital.cn

Tables 1 to 11 are related to the spectra obtained from MCNP code. Tables 12 to 22 show the spectra predicted by the MLP neural network.

Table.1. Spectra obtained by MCNP code: No filter

| energy bin (kev)/voltage(kv) | 20kv | 30kv | 40kv | 50kv | 60kv | 80kv | 100kv | 130kv | 150kv |
| --- | --- | --- | --- | --- | --- | --- | --- | --- | --- |
| 1 | 0.00E+00 | 0.00E+00 | 0.00E+00 | 0.00E+00 | 0.00E+00 | 0.00E+00 | 0.00E+00 | 0.00E+00 | 0.00E+00 |
| 2 | 2.42E-12 | 1.88E-12 | 2.38E-12 | 6.51E-13 | 1.44E-12 | 2.48E-12 | 3.77E-12 | 8.59E-12 | 4.76E-12 |
| 3 | 2.41E-07 | 3.37E-08 | 2.90E-08 | 2.85E-08 | 2.70E-08 | 2.60E-08 | 2.70E-08 | 3.58E-08 | 4.01E-08 |
| 4 | 1.11E-06 | 7.00E-07 | 6.52E-07 | 5.87E-07 | 5.40E-07 | 5.01E-07 | 4.80E-07 | 5.67E-07 | 4.96E-07 |
| 5 | 1.71E-06 | 2.30E-06 | 2.25E-06 | 2.18E-06 | 2.01E-06 | 1.80E-06 | 1.70E-06 | 1.87E-06 | 1.57E-06 |
| 6 | 2.29E-06 | 3.62E-06 | 3.94E-06 | 3.95E-06 | 3.86E-06 | 3.54E-06 | 3.23E-06 | 3.28E-06 | 2.83E-06 |
| 7 | 2.78E-06 | 4.33E-06 | 4.96E-06 | 5.29E-06 | 5.38E-06 | 5.09E-06 | 4.87E-06 | 5.06E-06 | 4.15E-06 |
| 8 | 2.86E-06 | 4.45E-06 | 5.38E-06 | 6.06E-06 | 6.37E-06 | 6.54E-06 | 6.29E-06 | 6.61E-06 | 5.70E-06 |
| 9 | 2.68E-06 | 5.49E-06 | 7.55E-06 | 9.32E-06 | 1.06E-05 | 1.24E-05 | 1.34E-05 | 1.56E-05 | 1.38E-05 |
| 10 | 2.40E-06 | 4.02E-06 | 5.27E-06 | 6.42E-06 | 7.25E-06 | 8.25E-06 | 8.85E-06 | 1.00E-05 | 8.84E-06 |
| 11 | 2.08E-06 | 3.15E-06 | 4.06E-06 | 4.65E-06 | 5.05E-06 | 5.35E-06 | 5.35E-06 | 5.88E-06 | 5.01E-06 |
| 12 | 1.69E-06 | 2.80E-06 | 3.53E-06 | 4.06E-06 | 4.31E-06 | 4.46E-06 | 4.48E-06 | 4.74E-06 | 3.92E-06 |
| 13 | 1.40E-06 | 2.41E-06 | 3.06E-06 | 3.47E-06 | 3.73E-06 | 3.70E-06 | 3.70E-06 | 3.78E-06 | 3.22E-06 |
| 14 | 1.17E-06 | 2.21E-06 | 2.90E-06 | 3.36E-06 | 3.74E-06 | 3.96E-06 | 4.04E-06 | 4.17E-06 | 3.55E-06 |
| 15 | 9.70E-07 | 2.05E-06 | 2.79E-06 | 3.35E-06 | 3.71E-06 | 4.04E-06 | 4.18E-06 | 4.53E-06 | 3.89E-06 |
| 16 | 7.89E-07 | 1.88E-06 | 2.63E-06 | 3.24E-06 | 3.64E-06 | 4.11E-06 | 4.33E-06 | 4.75E-06 | 4.19E-06 |
| 17 | 6.13E-07 | 1.72E-06 | 2.50E-06 | 3.14E-06 | 3.57E-06 | 4.15E-06 | 4.47E-06 | 5.01E-06 | 4.47E-06 |
| 18 | 4.44E-07 | 1.55E-06 | 2.33E-06 | 3.01E-06 | 3.48E-06 | 4.16E-06 | 4.56E-06 | 5.20E-06 | 4.72E-06 |
| 19 | 2.73E-07 | 1.40E-06 | 2.18E-06 | 2.87E-06 | 3.35E-06 | 4.14E-06 | 4.62E-06 | 5.34E-06 | 4.93E-06 |
| 20 | 1.03E-07 | 1.25E-06 | 2.04E-06 | 2.73E-06 | 3.23E-06 | 4.08E-06 | 4.64E-06 | 5.44E-06 | 5.09E-06 |
| 21 | 0.00E+00 | 1.11E-06 | 1.89E-06 | 2.57E-06 | 3.12E-06 | 3.98E-06 | 4.63E-06 | 5.52E-06 | 5.22E-06 |
| 22 | 0.00E+00 | 9.79E-07 | 1.73E-06 | 2.43E-06 | 2.99E-06 | 3.88E-06 | 4.57E-06 | 5.51E-06 | 5.33E-06 |
| 23 | 0.00E+00 | 8.52E-07 | 1.61E-06 | 2.28E-06 | 2.85E-06 | 3.76E-06 | 4.53E-06 | 5.48E-06 | 5.38E-06 |
| 24 | 0.00E+00 | 7.31E-07 | 1.48E-06 | 2.15E-06 | 2.73E-06 | 3.65E-06 | 4.46E-06 | 5.45E-06 | 5.43E-06 |
| 25 | 0.00E+00 | 6.20E-07 | 1.35E-06 | 2.00E-06 | 2.62E-06 | 3.53E-06 | 4.37E-06 | 5.37E-06 | 5.44E-06 |
| 26 | 0.00E+00 | 5.10E-07 | 1.24E-06 | 1.89E-06 | 2.47E-06 | 3.42E-06 | 4.25E-06 | 5.30E-06 | 5.43E-06 |
| 27 | 0.00E+00 | 4.02E-07 | 1.14E-06 | 1.77E-06 | 2.33E-06 | 3.30E-06 | 4.12E-06 | 5.24E-06 | 5.40E-06 |
| 28 | 0.00E+00 | 2.92E-07 | 1.03E-06 | 1.65E-06 | 2.22E-06 | 3.19E-06 | 4.00E-06 | 5.14E-06 | 5.35E-06 |
| 29 | 0.00E+00 | 1.83E-07 | 9.41E-07 | 1.54E-06 | 2.10E-06 | 3.07E-06 | 3.90E-06 | 5.01E-06 | 5.29E-06 |
| 30 | 0.00E+00 | 6.47E-08 | 8.43E-07 | 1.43E-06 | 1.96E-06 | 2.94E-06 | 3.78E-06 | 4.88E-06 | 5.21E-06 |
| 31 | 0.00E+00 | 0.00E+00 | 7.51E-07 | 1.34E-06 | 1.86E-06 | 2.83E-06 | 3.68E-06 | 4.74E-06 | 5.14E-06 |
| 32 | 0.00E+00 | 0.00E+00 | 6.66E-07 | 1.25E-06 | 1.76E-06 | 2.71E-06 | 3.58E-06 | 4.57E-06 | 5.07E-06 |
| 33 | 0.00E+00 | 0.00E+00 | 5.82E-07 | 1.16E-06 | 1.66E-06 | 2.58E-06 | 3.45E-06 | 4.44E-06 | 4.99E-06 |
| 34 | 0.00E+00 | 0.00E+00 | 4.98E-07 | 1.08E-06 | 1.57E-06 | 2.46E-06 | 3.32E-06 | 4.30E-06 | 4.90E-06 |
| 35 | 0.00E+00 | 0.00E+00 | 4.23E-07 | 9.96E-07 | 1.49E-06 | 2.35E-06 | 3.20E-06 | 4.19E-06 | 4.79E-06 |
| 36 | 0.00E+00 | 0.00E+00 | 3.44E-07 | 9.21E-07 | 1.40E-06 | 2.22E-06 | 3.09E-06 | 4.07E-06 | 4.67E-06 |
| 37 | 0.00E+00 | 0.00E+00 | 2.63E-07 | 8.46E-07 | 1.33E-06 | 2.11E-06 | 2.97E-06 | 3.96E-06 | 4.55E-06 |
| 38 | 0.00E+00 | 0.00E+00 | 1.91E-07 | 7.68E-07 | 1.24E-06 | 2.01E-06 | 2.84E-06 | 3.85E-06 | 4.42E-06 |
| 39 | 0.00E+00 | 0.00E+00 | 1.14E-07 | 6.92E-07 | 1.15E-06 | 1.94E-06 | 2.74E-06 | 3.75E-06 | 4.30E-06 |
| 40 | 0.00E+00 | 0.00E+00 | 5.16E-08 | 6.32E-07 | 1.07E-06 | 1.85E-06 | 2.63E-06 | 3.65E-06 | 4.18E-06 |
| 41 | 0.00E+00 | 0.00E+00 | 0.00E+00 | 5.52E-07 | 1.01E-06 | 1.80E-06 | 2.52E-06 | 3.54E-06 | 4.08E-06 |
| 42 | 0.00E+00 | 0.00E+00 | 0.00E+00 | 4.94E-07 | 9.42E-07 | 1.71E-06 | 2.43E-06 | 3.42E-06 | 3.97E-06 |
| 43 | 0.00E+00 | 0.00E+00 | 0.00E+00 | 4.36E-07 | 8.84E-07 | 1.66E-06 | 2.35E-06 | 3.30E-06 | 3.87E-06 |
| 44 | 0.00E+00 | 0.00E+00 | 0.00E+00 | 3.72E-07 | 8.24E-07 | 1.58E-06 | 2.27E-06 | 3.21E-06 | 3.76E-06 |
| 45 | 0.00E+00 | 0.00E+00 | 0.00E+00 | 3.14E-07 | 7.65E-07 | 1.50E-06 | 2.18E-06 | 3.12E-06 | 3.66E-06 |
| 46 | 0.00E+00 | 0.00E+00 | 0.00E+00 | 2.62E-07 | 7.03E-07 | 1.43E-06 | 2.12E-06 | 3.01E-06 | 3.56E-06 |
| 47 | 0.00E+00 | 0.00E+00 | 0.00E+00 | 2.05E-07 | 6.44E-07 | 1.36E-06 | 2.05E-06 | 2.94E-06 | 3.47E-06 |
| 48 | 0.00E+00 | 0.00E+00 | 0.00E+00 | 1.50E-07 | 5.91E-07 | 1.30E-06 | 1.97E-06 | 2.85E-06 | 3.38E-06 |
| 49 | 0.00E+00 | 0.00E+00 | 0.00E+00 | 9.76E-08 | 5.46E-07 | 1.25E-06 | 1.89E-06 | 2.73E-06 | 3.30E-06 |
| 50 | 0.00E+00 | 0.00E+00 | 0.00E+00 | 3.96E-08 | 4.95E-07 | 1.19E-06 | 1.81E-06 | 2.64E-06 | 3.21E-06 |
| 51 | 0.00E+00 | 0.00E+00 | 0.00E+00 | 0.00E+00 | 4.47E-07 | 1.12E-06 | 1.73E-06 | 2.58E-06 | 3.12E-06 |
| 52 | 0.00E+00 | 0.00E+00 | 0.00E+00 | 0.00E+00 | 3.95E-07 | 1.07E-06 | 1.69E-06 | 2.49E-06 | 3.03E-06 |
| 53 | 0.00E+00 | 0.00E+00 | 0.00E+00 | 0.00E+00 | 3.46E-07 | 1.00E-06 | 1.63E-06 | 2.44E-06 | 2.96E-06 |
| 54 | 0.00E+00 | 0.00E+00 | 0.00E+00 | 0.00E+00 | 2.98E-07 | 9.42E-07 | 1.58E-06 | 2.38E-06 | 2.88E-06 |
| 55 | 0.00E+00 | 0.00E+00 | 0.00E+00 | 0.00E+00 | 2.51E-07 | 9.00E-07 | 1.54E-06 | 2.34E-06 | 2.83E-06 |
| 56 | 0.00E+00 | 0.00E+00 | 0.00E+00 | 0.00E+00 | 2.06E-07 | 8.51E-07 | 1.49E-06 | 2.26E-06 | 2.76E-06 |
| 57 | 0.00E+00 | 0.00E+00 | 0.00E+00 | 0.00E+00 | 1.62E-07 | 8.12E-07 | 1.42E-06 | 2.18E-06 | 2.71E-06 |
| 58 | 0.00E+00 | 0.00E+00 | 0.00E+00 | 0.00E+00 | 1.15E-07 | 7.73E-07 | 1.32E-06 | 2.08E-06 | 2.62E-06 |
| 59 | 0.00E+00 | 0.00E+00 | 0.00E+00 | 0.00E+00 | 7.26E-08 | 8.86E-07 | 2.34E-06 | 5.05E-06 | 6.83E-06 |
| 60 | 0.00E+00 | 0.00E+00 | 0.00E+00 | 0.00E+00 | 2.80E-08 | 9.83E-07 | 3.09E-06 | 7.42E-06 | 1.03E-05 |
| 61 | 0.00E+00 | 0.00E+00 | 0.00E+00 | 0.00E+00 | 0.00E+00 | 6.36E-07 | 1.14E-06 | 1.94E-06 | 2.48E-06 |
| 62 | 0.00E+00 | 0.00E+00 | 0.00E+00 | 0.00E+00 | 0.00E+00 | 6.20E-07 | 1.24E-06 | 2.02E-06 | 2.67E-06 |
| 63 | 0.00E+00 | 0.00E+00 | 0.00E+00 | 0.00E+00 | 0.00E+00 | 6.18E-07 | 1.08E-06 | 1.69E-06 | 2.18E-06 |
| 64 | 0.00E+00 | 0.00E+00 | 0.00E+00 | 0.00E+00 | 0.00E+00 | 5.35E-07 | 1.06E-06 | 1.61E-06 | 2.12E-06 |
| 65 | 0.00E+00 | 0.00E+00 | 0.00E+00 | 0.00E+00 | 0.00E+00 | 4.95E-07 | 1.02E-06 | 1.63E-06 | 2.10E-06 |
| 66 | 0.00E+00 | 0.00E+00 | 0.00E+00 | 0.00E+00 | 0.00E+00 | 4.65E-07 | 9.61E-07 | 1.60E-06 | 2.07E-06 |
| 67 | 0.00E+00 | 0.00E+00 | 0.00E+00 | 0.00E+00 | 0.00E+00 | 4.45E-07 | 8.79E-07 | 1.53E-06 | 2.02E-06 |
| 68 | 0.00E+00 | 0.00E+00 | 0.00E+00 | 0.00E+00 | 0.00E+00 | 5.04E-07 | 1.57E-06 | 3.37E-06 | 4.78E-06 |
| 69 | 0.00E+00 | 0.00E+00 | 0.00E+00 | 0.00E+00 | 0.00E+00 | 3.52E-07 | 8.34E-07 | 1.49E-06 | 1.83E-06 |
| 70 | 0.00E+00 | 0.00E+00 | 0.00E+00 | 0.00E+00 | 0.00E+00 | 3.41E-07 | 9.79E-07 | 1.91E-06 | 2.55E-06 |
| 71 | 0.00E+00 | 0.00E+00 | 0.00E+00 | 0.00E+00 | 0.00E+00 | 2.88E-07 | 7.73E-07 | 1.29E-06 | 1.52E-06 |
| 72 | 0.00E+00 | 0.00E+00 | 0.00E+00 | 0.00E+00 | 0.00E+00 | 2.58E-07 | 6.87E-07 | 1.26E-06 | 1.53E-06 |
| 73 | 0.00E+00 | 0.00E+00 | 0.00E+00 | 0.00E+00 | 0.00E+00 | 2.22E-07 | 6.55E-07 | 1.18E-06 | 1.49E-06 |
| 74 | 0.00E+00 | 0.00E+00 | 0.00E+00 | 0.00E+00 | 0.00E+00 | 1.90E-07 | 6.17E-07 | 1.18E-06 | 1.45E-06 |
| 75 | 0.00E+00 | 0.00E+00 | 0.00E+00 | 0.00E+00 | 0.00E+00 | 1.59E-07 | 5.90E-07 | 1.14E-06 | 1.41E-06 |
| 76 | 0.00E+00 | 0.00E+00 | 0.00E+00 | 0.00E+00 | 0.00E+00 | 1.33E-07 | 5.65E-07 | 1.11E-06 | 1.38E-06 |
| 77 | 0.00E+00 | 0.00E+00 | 0.00E+00 | 0.00E+00 | 0.00E+00 | 1.02E-07 | 5.45E-07 | 1.09E-06 | 1.34E-06 |
| 78 | 0.00E+00 | 0.00E+00 | 0.00E+00 | 0.00E+00 | 0.00E+00 | 7.33E-08 | 5.26E-07 | 1.05E-06 | 1.31E-06 |
| 79 | 0.00E+00 | 0.00E+00 | 0.00E+00 | 0.00E+00 | 0.00E+00 | 4.58E-08 | 5.05E-07 | 9.98E-07 | 1.29E-06 |
| 80 | 0.00E+00 | 0.00E+00 | 0.00E+00 | 0.00E+00 | 0.00E+00 | 1.55E-08 | 4.81E-07 | 9.68E-07 | 1.26E-06 |
| 81 | 0.00E+00 | 0.00E+00 | 0.00E+00 | 0.00E+00 | 0.00E+00 | 0.00E+00 | 4.56E-07 | 9.43E-07 | 1.23E-06 |
| 82 | 0.00E+00 | 0.00E+00 | 0.00E+00 | 0.00E+00 | 0.00E+00 | 0.00E+00 | 4.21E-07 | 8.98E-07 | 1.21E-06 |
| 83 | 0.00E+00 | 0.00E+00 | 0.00E+00 | 0.00E+00 | 0.00E+00 | 0.00E+00 | 3.98E-07 | 8.65E-07 | 1.18E-06 |
| 84 | 0.00E+00 | 0.00E+00 | 0.00E+00 | 0.00E+00 | 0.00E+00 | 0.00E+00 | 3.75E-07 | 8.38E-07 | 1.15E-06 |
| 85 | 0.00E+00 | 0.00E+00 | 0.00E+00 | 0.00E+00 | 0.00E+00 | 0.00E+00 | 3.50E-07 | 8.09E-07 | 1.13E-06 |
| 86 | 0.00E+00 | 0.00E+00 | 0.00E+00 | 0.00E+00 | 0.00E+00 | 0.00E+00 | 3.25E-07 | 7.85E-07 | 1.10E-06 |
| 87 | 0.00E+00 | 0.00E+00 | 0.00E+00 | 0.00E+00 | 0.00E+00 | 0.00E+00 | 3.05E-07 | 7.68E-07 | 1.07E-06 |
| 88 | 0.00E+00 | 0.00E+00 | 0.00E+00 | 0.00E+00 | 0.00E+00 | 0.00E+00 | 2.76E-07 | 7.50E-07 | 1.04E-06 |
| 89 | 0.00E+00 | 0.00E+00 | 0.00E+00 | 0.00E+00 | 0.00E+00 | 0.00E+00 | 2.49E-07 | 7.29E-07 | 1.02E-06 |
| 90 | 0.00E+00 | 0.00E+00 | 0.00E+00 | 0.00E+00 | 0.00E+00 | 0.00E+00 | 2.21E-07 | 7.06E-07 | 9.94E-07 |
| 91 | 0.00E+00 | 0.00E+00 | 0.00E+00 | 0.00E+00 | 0.00E+00 | 0.00E+00 | 1.98E-07 | 6.83E-07 | 9.73E-07 |
| 92 | 0.00E+00 | 0.00E+00 | 0.00E+00 | 0.00E+00 | 0.00E+00 | 0.00E+00 | 1.78E-07 | 6.64E-07 | 9.50E-07 |
| 93 | 0.00E+00 | 0.00E+00 | 0.00E+00 | 0.00E+00 | 0.00E+00 | 0.00E+00 | 1.61E-07 | 6.42E-07 | 9.24E-07 |
| 94 | 0.00E+00 | 0.00E+00 | 0.00E+00 | 0.00E+00 | 0.00E+00 | 0.00E+00 | 1.40E-07 | 6.19E-07 | 8.92E-07 |
| 95 | 0.00E+00 | 0.00E+00 | 0.00E+00 | 0.00E+00 | 0.00E+00 | 0.00E+00 | 1.23E-07 | 6.01E-07 | 8.65E-07 |
| 96 | 0.00E+00 | 0.00E+00 | 0.00E+00 | 0.00E+00 | 0.00E+00 | 0.00E+00 | 1.00E-07 | 5.72E-07 | 8.33E-07 |
| 97 | 0.00E+00 | 0.00E+00 | 0.00E+00 | 0.00E+00 | 0.00E+00 | 0.00E+00 | 7.41E-08 | 5.44E-07 | 8.01E-07 |
| 98 | 0.00E+00 | 0.00E+00 | 0.00E+00 | 0.00E+00 | 0.00E+00 | 0.00E+00 | 5.28E-08 | 5.15E-07 | 7.78E-07 |
| 99 | 0.00E+00 | 0.00E+00 | 0.00E+00 | 0.00E+00 | 0.00E+00 | 0.00E+00 | 2.94E-08 | 4.96E-07 | 7.60E-07 |
| 100 | 0.00E+00 | 0.00E+00 | 0.00E+00 | 0.00E+00 | 0.00E+00 | 0.00E+00 | 1.25E-08 | 4.81E-07 | 7.40E-07 |
| 101 | 0.00E+00 | 0.00E+00 | 0.00E+00 | 0.00E+00 | 0.00E+00 | 0.00E+00 | 0.00E+00 | 4.63E-07 | 7.26E-07 |
| 102 | 0.00E+00 | 0.00E+00 | 0.00E+00 | 0.00E+00 | 0.00E+00 | 0.00E+00 | 0.00E+00 | 4.53E-07 | 7.13E-07 |
| 103 | 0.00E+00 | 0.00E+00 | 0.00E+00 | 0.00E+00 | 0.00E+00 | 0.00E+00 | 0.00E+00 | 4.40E-07 | 6.92E-07 |
| 104 | 0.00E+00 | 0.00E+00 | 0.00E+00 | 0.00E+00 | 0.00E+00 | 0.00E+00 | 0.00E+00 | 4.23E-07 | 6.69E-07 |
| 105 | 0.00E+00 | 0.00E+00 | 0.00E+00 | 0.00E+00 | 0.00E+00 | 0.00E+00 | 0.00E+00 | 3.90E-07 | 6.50E-07 |
| 106 | 0.00E+00 | 0.00E+00 | 0.00E+00 | 0.00E+00 | 0.00E+00 | 0.00E+00 | 0.00E+00 | 3.76E-07 | 6.28E-07 |
| 107 | 0.00E+00 | 0.00E+00 | 0.00E+00 | 0.00E+00 | 0.00E+00 | 0.00E+00 | 0.00E+00 | 3.49E-07 | 6.07E-07 |
| 108 | 0.00E+00 | 0.00E+00 | 0.00E+00 | 0.00E+00 | 0.00E+00 | 0.00E+00 | 0.00E+00 | 3.33E-07 | 5.93E-07 |
| 109 | 0.00E+00 | 0.00E+00 | 0.00E+00 | 0.00E+00 | 0.00E+00 | 0.00E+00 | 0.00E+00 | 3.19E-07 | 5.78E-07 |
| 110 | 0.00E+00 | 0.00E+00 | 0.00E+00 | 0.00E+00 | 0.00E+00 | 0.00E+00 | 0.00E+00 | 3.19E-07 | 5.65E-07 |
| 111 | 0.00E+00 | 0.00E+00 | 0.00E+00 | 0.00E+00 | 0.00E+00 | 0.00E+00 | 0.00E+00 | 2.97E-07 | 5.52E-07 |
| 112 | 0.00E+00 | 0.00E+00 | 0.00E+00 | 0.00E+00 | 0.00E+00 | 0.00E+00 | 0.00E+00 | 2.87E-07 | 5.37E-07 |
| 113 | 0.00E+00 | 0.00E+00 | 0.00E+00 | 0.00E+00 | 0.00E+00 | 0.00E+00 | 0.00E+00 | 2.62E-07 | 5.19E-07 |
| 114 | 0.00E+00 | 0.00E+00 | 0.00E+00 | 0.00E+00 | 0.00E+00 | 0.00E+00 | 0.00E+00 | 2.41E-07 | 5.01E-07 |
| 115 | 0.00E+00 | 0.00E+00 | 0.00E+00 | 0.00E+00 | 0.00E+00 | 0.00E+00 | 0.00E+00 | 2.13E-07 | 4.76E-07 |
| 116 | 0.00E+00 | 0.00E+00 | 0.00E+00 | 0.00E+00 | 0.00E+00 | 0.00E+00 | 0.00E+00 | 1.94E-07 | 4.53E-07 |
| 117 | 0.00E+00 | 0.00E+00 | 0.00E+00 | 0.00E+00 | 0.00E+00 | 0.00E+00 | 0.00E+00 | 1.74E-07 | 4.32E-07 |
| 118 | 0.00E+00 | 0.00E+00 | 0.00E+00 | 0.00E+00 | 0.00E+00 | 0.00E+00 | 0.00E+00 | 1.63E-07 | 4.12E-07 |
| 119 | 0.00E+00 | 0.00E+00 | 0.00E+00 | 0.00E+00 | 0.00E+00 | 0.00E+00 | 0.00E+00 | 1.50E-07 | 3.95E-07 |
| 120 | 0.00E+00 | 0.00E+00 | 0.00E+00 | 0.00E+00 | 0.00E+00 | 0.00E+00 | 0.00E+00 | 1.41E-07 | 3.82E-07 |
| 121 | 0.00E+00 | 0.00E+00 | 0.00E+00 | 0.00E+00 | 0.00E+00 | 0.00E+00 | 0.00E+00 | 1.30E-07 | 3.69E-07 |
| 122 | 0.00E+00 | 0.00E+00 | 0.00E+00 | 0.00E+00 | 0.00E+00 | 0.00E+00 | 0.00E+00 | 1.16E-07 | 3.54E-07 |
| 123 | 0.00E+00 | 0.00E+00 | 0.00E+00 | 0.00E+00 | 0.00E+00 | 0.00E+00 | 0.00E+00 | 1.02E-07 | 3.41E-07 |
| 124 | 0.00E+00 | 0.00E+00 | 0.00E+00 | 0.00E+00 | 0.00E+00 | 0.00E+00 | 0.00E+00 | 8.57E-08 | 3.29E-07 |
| 125 | 0.00E+00 | 0.00E+00 | 0.00E+00 | 0.00E+00 | 0.00E+00 | 0.00E+00 | 0.00E+00 | 6.89E-08 | 3.17E-07 |
| 126 | 0.00E+00 | 0.00E+00 | 0.00E+00 | 0.00E+00 | 0.00E+00 | 0.00E+00 | 0.00E+00 | 5.69E-08 | 3.02E-07 |
| 127 | 0.00E+00 | 0.00E+00 | 0.00E+00 | 0.00E+00 | 0.00E+00 | 0.00E+00 | 0.00E+00 | 4.47E-08 | 2.89E-07 |
| 128 | 0.00E+00 | 0.00E+00 | 0.00E+00 | 0.00E+00 | 0.00E+00 | 0.00E+00 | 0.00E+00 | 3.05E-08 | 2.73E-07 |
| 129 | 0.00E+00 | 0.00E+00 | 0.00E+00 | 0.00E+00 | 0.00E+00 | 0.00E+00 | 0.00E+00 | 2.05E-08 | 2.55E-07 |
| 130 | 0.00E+00 | 0.00E+00 | 0.00E+00 | 0.00E+00 | 0.00E+00 | 0.00E+00 | 0.00E+00 | 4.69E-09 | 2.38E-07 |
| 131 | 0.00E+00 | 0.00E+00 | 0.00E+00 | 0.00E+00 | 0.00E+00 | 0.00E+00 | 0.00E+00 | 0.00E+00 | 2.23E-07 |
| 132 | 0.00E+00 | 0.00E+00 | 0.00E+00 | 0.00E+00 | 0.00E+00 | 0.00E+00 | 0.00E+00 | 0.00E+00 | 2.08E-07 |
| 133 | 0.00E+00 | 0.00E+00 | 0.00E+00 | 0.00E+00 | 0.00E+00 | 0.00E+00 | 0.00E+00 | 0.00E+00 | 1.96E-07 |
| 134 | 0.00E+00 | 0.00E+00 | 0.00E+00 | 0.00E+00 | 0.00E+00 | 0.00E+00 | 0.00E+00 | 0.00E+00 | 1.84E-07 |
| 135 | 0.00E+00 | 0.00E+00 | 0.00E+00 | 0.00E+00 | 0.00E+00 | 0.00E+00 | 0.00E+00 | 0.00E+00 | 1.72E-07 |
| 136 | 0.00E+00 | 0.00E+00 | 0.00E+00 | 0.00E+00 | 0.00E+00 | 0.00E+00 | 0.00E+00 | 0.00E+00 | 1.60E-07 |
| 137 | 0.00E+00 | 0.00E+00 | 0.00E+00 | 0.00E+00 | 0.00E+00 | 0.00E+00 | 0.00E+00 | 0.00E+00 | 1.48E-07 |
| 138 | 0.00E+00 | 0.00E+00 | 0.00E+00 | 0.00E+00 | 0.00E+00 | 0.00E+00 | 0.00E+00 | 0.00E+00 | 1.36E-07 |
| 139 | 0.00E+00 | 0.00E+00 | 0.00E+00 | 0.00E+00 | 0.00E+00 | 0.00E+00 | 0.00E+00 | 0.00E+00 | 1.23E-07 |
| 140 | 0.00E+00 | 0.00E+00 | 0.00E+00 | 0.00E+00 | 0.00E+00 | 0.00E+00 | 0.00E+00 | 0.00E+00 | 1.11E-07 |
| 141 | 0.00E+00 | 0.00E+00 | 0.00E+00 | 0.00E+00 | 0.00E+00 | 0.00E+00 | 0.00E+00 | 0.00E+00 | 9.95E-08 |
| 142 | 0.00E+00 | 0.00E+00 | 0.00E+00 | 0.00E+00 | 0.00E+00 | 0.00E+00 | 0.00E+00 | 0.00E+00 | 8.92E-08 |
| 143 | 0.00E+00 | 0.00E+00 | 0.00E+00 | 0.00E+00 | 0.00E+00 | 0.00E+00 | 0.00E+00 | 0.00E+00 | 7.86E-08 |
| 144 | 0.00E+00 | 0.00E+00 | 0.00E+00 | 0.00E+00 | 0.00E+00 | 0.00E+00 | 0.00E+00 | 0.00E+00 | 6.93E-08 |
| 145 | 0.00E+00 | 0.00E+00 | 0.00E+00 | 0.00E+00 | 0.00E+00 | 0.00E+00 | 0.00E+00 | 0.00E+00 | 5.98E-08 |
| 146 | 0.00E+00 | 0.00E+00 | 0.00E+00 | 0.00E+00 | 0.00E+00 | 0.00E+00 | 0.00E+00 | 0.00E+00 | 4.98E-08 |
| 147 | 0.00E+00 | 0.00E+00 | 0.00E+00 | 0.00E+00 | 0.00E+00 | 0.00E+00 | 0.00E+00 | 0.00E+00 | 3.93E-08 |
| 148 | 0.00E+00 | 0.00E+00 | 0.00E+00 | 0.00E+00 | 0.00E+00 | 0.00E+00 | 0.00E+00 | 0.00E+00 | 2.81E-08 |
| 149 | 0.00E+00 | 0.00E+00 | 0.00E+00 | 0.00E+00 | 0.00E+00 | 0.00E+00 | 0.00E+00 | 0.00E+00 | 1.66E-08 |

Table.2. Spectra obtained by MCNP code: Be- 0.4mm

| energy bin (kev)/voltage(kv) | 20kv | 30kv | 40kv | 50kv | 60kv | 80kv | 100kv | 130kv | 150kv |
| --- | --- | --- | --- | --- | --- | --- | --- | --- | --- |
| 1 | 0.00E+00 | 0.00E+00 | 0.00E+00 | 0.00E+00 | 0.00E+00 | 0.00E+00 | 0.00E+00 | 0.00E+00 | 0.00E+00 |
| 2 | 9.62E-15 | 7.47E-15 | 9.46E-15 | 2.59E-15 | 5.72E-15 | 9.86E-15 | 1.50E-14 | 3.41E-14 | 1.89E-14 |
| 3 | 4.98E-08 | 6.97E-09 | 6.00E-09 | 5.89E-09 | 5.58E-09 | 5.38E-09 | 5.58E-09 | 7.40E-09 | 8.29E-09 |
| 4 | 5.84E-07 | 3.68E-07 | 3.43E-07 | 3.09E-07 | 2.84E-07 | 2.63E-07 | 2.52E-07 | 2.98E-07 | 2.61E-07 |
| 5 | 1.24E-06 | 1.66E-06 | 1.63E-06 | 1.58E-06 | 1.45E-06 | 1.30E-06 | 1.23E-06 | 1.35E-06 | 1.14E-06 |
| 6 | 1.90E-06 | 3.00E-06 | 3.27E-06 | 3.28E-06 | 3.20E-06 | 2.94E-06 | 2.68E-06 | 2.72E-06 | 2.35E-06 |
| 7 | 2.43E-06 | 3.78E-06 | 4.33E-06 | 4.62E-06 | 4.70E-06 | 4.45E-06 | 4.25E-06 | 4.42E-06 | 3.62E-06 |
| 8 | 2.63E-06 | 4.10E-06 | 4.95E-06 | 5.58E-06 | 5.86E-06 | 6.02E-06 | 5.79E-06 | 6.08E-06 | 5.25E-06 |
| 9 | 2.51E-06 | 5.14E-06 | 7.07E-06 | 8.73E-06 | 9.93E-06 | 1.16E-05 | 1.26E-05 | 1.46E-05 | 1.29E-05 |
| 10 | 2.29E-06 | 3.83E-06 | 5.02E-06 | 6.12E-06 | 6.91E-06 | 7.86E-06 | 8.44E-06 | 9.53E-06 | 8.43E-06 |
| 11 | 1.99E-06 | 3.02E-06 | 3.89E-06 | 4.45E-06 | 4.84E-06 | 5.13E-06 | 5.13E-06 | 5.63E-06 | 4.80E-06 |
| 12 | 1.63E-06 | 2.70E-06 | 3.40E-06 | 3.91E-06 | 4.15E-06 | 4.29E-06 | 4.31E-06 | 4.56E-06 | 3.77E-06 |
| 13 | 1.35E-06 | 2.33E-06 | 2.96E-06 | 3.36E-06 | 3.61E-06 | 3.58E-06 | 3.58E-06 | 3.66E-06 | 3.12E-06 |
| 14 | 1.14E-06 | 2.15E-06 | 2.82E-06 | 3.27E-06 | 3.64E-06 | 3.85E-06 | 3.93E-06 | 4.06E-06 | 3.45E-06 |
| 15 | 9.53E-07 | 2.00E-06 | 2.71E-06 | 3.24E-06 | 3.61E-06 | 3.94E-06 | 4.08E-06 | 4.38E-06 | 3.79E-06 |
| 16 | 7.79E-07 | 1.84E-06 | 2.57E-06 | 3.15E-06 | 3.55E-06 | 3.99E-06 | 4.22E-06 | 4.63E-06 | 4.07E-06 |
| 17 | 6.05E-07 | 1.69E-06 | 2.44E-06 | 3.06E-06 | 3.48E-06 | 4.04E-06 | 4.34E-06 | 4.87E-06 | 4.35E-06 |
| 18 | 4.36E-07 | 1.53E-06 | 2.29E-06 | 2.94E-06 | 3.39E-06 | 4.05E-06 | 4.44E-06 | 5.05E-06 | 4.59E-06 |
| 19 | 2.68E-07 | 1.38E-06 | 2.15E-06 | 2.81E-06 | 3.29E-06 | 4.03E-06 | 4.50E-06 | 5.21E-06 | 4.80E-06 |
| 20 | 1.01E-07 | 1.24E-06 | 2.00E-06 | 2.68E-06 | 3.18E-06 | 3.98E-06 | 4.53E-06 | 5.31E-06 | 4.97E-06 |
| 21 | 0.00E+00 | 1.10E-06 | 1.86E-06 | 2.53E-06 | 3.06E-06 | 3.90E-06 | 4.52E-06 | 5.37E-06 | 5.11E-06 |
| 22 | 0.00E+00 | 9.69E-07 | 1.72E-06 | 2.39E-06 | 2.94E-06 | 3.81E-06 | 4.49E-06 | 5.39E-06 | 5.21E-06 |
| 23 | 0.00E+00 | 8.45E-07 | 1.59E-06 | 2.25E-06 | 2.82E-06 | 3.70E-06 | 4.44E-06 | 5.38E-06 | 5.28E-06 |
| 24 | 0.00E+00 | 7.27E-07 | 1.46E-06 | 2.12E-06 | 2.69E-06 | 3.59E-06 | 4.37E-06 | 5.34E-06 | 5.32E-06 |
| 25 | 0.00E+00 | 6.14E-07 | 1.34E-06 | 1.99E-06 | 2.56E-06 | 3.48E-06 | 4.28E-06 | 5.29E-06 | 5.34E-06 |
| 26 | 0.00E+00 | 5.04E-07 | 1.23E-06 | 1.86E-06 | 2.44E-06 | 3.37E-06 | 4.18E-06 | 5.22E-06 | 5.33E-06 |
| 27 | 0.00E+00 | 3.96E-07 | 1.12E-06 | 1.74E-06 | 2.31E-06 | 3.26E-06 | 4.07E-06 | 5.14E-06 | 5.31E-06 |
| 28 | 0.00E+00 | 2.86E-07 | 1.02E-06 | 1.63E-06 | 2.19E-06 | 3.14E-06 | 3.95E-06 | 5.04E-06 | 5.26E-06 |
| 29 | 0.00E+00 | 1.77E-07 | 9.28E-07 | 1.52E-06 | 2.07E-06 | 3.02E-06 | 3.84E-06 | 4.93E-06 | 5.21E-06 |
| 30 | 0.00E+00 | 6.38E-08 | 8.35E-07 | 1.42E-06 | 1.95E-06 | 2.91E-06 | 3.74E-06 | 4.80E-06 | 5.14E-06 |
| 31 | 0.00E+00 | 0.00E+00 | 7.47E-07 | 1.33E-06 | 1.84E-06 | 2.79E-06 | 3.63E-06 | 4.67E-06 | 5.07E-06 |
| 32 | 0.00E+00 | 0.00E+00 | 6.59E-07 | 1.24E-06 | 1.74E-06 | 2.67E-06 | 3.52E-06 | 4.53E-06 | 5.00E-06 |
| 33 | 0.00E+00 | 0.00E+00 | 5.76E-07 | 1.15E-06 | 1.65E-06 | 2.55E-06 | 3.40E-06 | 4.39E-06 | 4.91E-06 |
| 34 | 0.00E+00 | 0.00E+00 | 4.96E-07 | 1.07E-06 | 1.56E-06 | 2.43E-06 | 3.29E-06 | 4.26E-06 | 4.82E-06 |
| 35 | 0.00E+00 | 0.00E+00 | 4.17E-07 | 9.88E-07 | 1.47E-06 | 2.31E-06 | 3.17E-06 | 4.14E-06 | 4.72E-06 |
| 36 | 0.00E+00 | 0.00E+00 | 3.39E-07 | 9.11E-07 | 1.39E-06 | 2.20E-06 | 3.05E-06 | 4.02E-06 | 4.61E-06 |
| 37 | 0.00E+00 | 0.00E+00 | 2.64E-07 | 8.34E-07 | 1.31E-06 | 2.10E-06 | 2.93E-06 | 3.91E-06 | 4.49E-06 |
| 38 | 0.00E+00 | 0.00E+00 | 1.90E-07 | 7.62E-07 | 1.22E-06 | 2.00E-06 | 2.82E-06 | 3.81E-06 | 4.37E-06 |
| 39 | 0.00E+00 | 0.00E+00 | 1.17E-07 | 6.89E-07 | 1.15E-06 | 1.92E-06 | 2.71E-06 | 3.70E-06 | 4.25E-06 |
| 40 | 0.00E+00 | 0.00E+00 | 5.10E-08 | 6.20E-07 | 1.07E-06 | 1.84E-06 | 2.60E-06 | 3.60E-06 | 4.14E-06 |
| 41 | 0.00E+00 | 0.00E+00 | 0.00E+00 | 5.54E-07 | 9.99E-07 | 1.77E-06 | 2.50E-06 | 3.49E-06 | 4.03E-06 |
| 42 | 0.00E+00 | 0.00E+00 | 0.00E+00 | 4.91E-07 | 9.35E-07 | 1.70E-06 | 2.41E-06 | 3.38E-06 | 3.92E-06 |
| 43 | 0.00E+00 | 0.00E+00 | 0.00E+00 | 4.28E-07 | 8.74E-07 | 1.63E-06 | 2.32E-06 | 3.28E-06 | 3.82E-06 |
| 44 | 0.00E+00 | 0.00E+00 | 0.00E+00 | 3.71E-07 | 8.14E-07 | 1.56E-06 | 2.24E-06 | 3.17E-06 | 3.72E-06 |
| 45 | 0.00E+00 | 0.00E+00 | 0.00E+00 | 3.14E-07 | 7.55E-07 | 1.49E-06 | 2.17E-06 | 3.08E-06 | 3.62E-06 |
| 46 | 0.00E+00 | 0.00E+00 | 0.00E+00 | 2.58E-07 | 6.97E-07 | 1.42E-06 | 2.09E-06 | 2.99E-06 | 3.52E-06 |
| 47 | 0.00E+00 | 0.00E+00 | 0.00E+00 | 2.03E-07 | 6.42E-07 | 1.35E-06 | 2.02E-06 | 2.90E-06 | 3.43E-06 |
| 48 | 0.00E+00 | 0.00E+00 | 0.00E+00 | 1.49E-07 | 5.89E-07 | 1.29E-06 | 1.95E-06 | 2.80E-06 | 3.34E-06 |
| 49 | 0.00E+00 | 0.00E+00 | 0.00E+00 | 9.46E-08 | 5.38E-07 | 1.23E-06 | 1.87E-06 | 2.72E-06 | 3.26E-06 |
| 50 | 0.00E+00 | 0.00E+00 | 0.00E+00 | 3.91E-08 | 4.89E-07 | 1.17E-06 | 1.80E-06 | 2.63E-06 | 3.17E-06 |
| 51 | 0.00E+00 | 0.00E+00 | 0.00E+00 | 0.00E+00 | 4.41E-07 | 1.11E-06 | 1.73E-06 | 2.55E-06 | 3.09E-06 |
| 52 | 0.00E+00 | 0.00E+00 | 0.00E+00 | 0.00E+00 | 3.92E-07 | 1.05E-06 | 1.67E-06 | 2.48E-06 | 3.01E-06 |
| 53 | 0.00E+00 | 0.00E+00 | 0.00E+00 | 0.00E+00 | 3.43E-07 | 9.95E-07 | 1.62E-06 | 2.42E-06 | 2.93E-06 |
| 54 | 0.00E+00 | 0.00E+00 | 0.00E+00 | 0.00E+00 | 2.96E-07 | 9.42E-07 | 1.57E-06 | 2.36E-06 | 2.86E-06 |
| 55 | 0.00E+00 | 0.00E+00 | 0.00E+00 | 0.00E+00 | 2.50E-07 | 8.91E-07 | 1.51E-06 | 2.29E-06 | 2.80E-06 |
| 56 | 0.00E+00 | 0.00E+00 | 0.00E+00 | 0.00E+00 | 2.04E-07 | 8.46E-07 | 1.45E-06 | 2.22E-06 | 2.73E-06 |
| 57 | 0.00E+00 | 0.00E+00 | 0.00E+00 | 0.00E+00 | 1.59E-07 | 8.03E-07 | 1.39E-06 | 2.15E-06 | 2.67E-06 |
| 58 | 0.00E+00 | 0.00E+00 | 0.00E+00 | 0.00E+00 | 1.14E-07 | 7.64E-07 | 1.31E-06 | 2.06E-06 | 2.59E-06 |
| 59 | 0.00E+00 | 0.00E+00 | 0.00E+00 | 0.00E+00 | 7.18E-08 | 8.76E-07 | 2.31E-06 | 4.99E-06 | 6.75E-06 |
| 60 | 0.00E+00 | 0.00E+00 | 0.00E+00 | 0.00E+00 | 2.77E-08 | 9.72E-07 | 3.06E-06 | 7.34E-06 | 1.02E-05 |
| 61 | 0.00E+00 | 0.00E+00 | 0.00E+00 | 0.00E+00 | 0.00E+00 | 6.29E-07 | 1.13E-06 | 1.92E-06 | 2.45E-06 |
| 62 | 0.00E+00 | 0.00E+00 | 0.00E+00 | 0.00E+00 | 0.00E+00 | 6.13E-07 | 1.23E-06 | 2.00E-06 | 2.64E-06 |
| 63 | 0.00E+00 | 0.00E+00 | 0.00E+00 | 0.00E+00 | 0.00E+00 | 6.11E-07 | 1.07E-06 | 1.67E-06 | 2.16E-06 |
| 64 | 0.00E+00 | 0.00E+00 | 0.00E+00 | 0.00E+00 | 0.00E+00 | 5.29E-07 | 1.05E-06 | 1.59E-06 | 2.10E-06 |
| 65 | 0.00E+00 | 0.00E+00 | 0.00E+00 | 0.00E+00 | 0.00E+00 | 4.93E-07 | 1.00E-06 | 1.60E-06 | 2.07E-06 |
| 66 | 0.00E+00 | 0.00E+00 | 0.00E+00 | 0.00E+00 | 0.00E+00 | 4.63E-07 | 9.43E-07 | 1.57E-06 | 2.04E-06 |
| 67 | 0.00E+00 | 0.00E+00 | 0.00E+00 | 0.00E+00 | 0.00E+00 | 4.40E-07 | 8.70E-07 | 1.51E-06 | 2.00E-06 |
| 68 | 0.00E+00 | 0.00E+00 | 0.00E+00 | 0.00E+00 | 0.00E+00 | 4.99E-07 | 1.55E-06 | 3.33E-06 | 4.73E-06 |
| 69 | 0.00E+00 | 0.00E+00 | 0.00E+00 | 0.00E+00 | 0.00E+00 | 3.48E-07 | 8.25E-07 | 1.47E-06 | 1.81E-06 |
| 70 | 0.00E+00 | 0.00E+00 | 0.00E+00 | 0.00E+00 | 0.00E+00 | 3.37E-07 | 9.69E-07 | 1.89E-06 | 2.52E-06 |
| 71 | 0.00E+00 | 0.00E+00 | 0.00E+00 | 0.00E+00 | 0.00E+00 | 2.85E-07 | 7.65E-07 | 1.28E-06 | 1.50E-06 |
| 72 | 0.00E+00 | 0.00E+00 | 0.00E+00 | 0.00E+00 | 0.00E+00 | 2.55E-07 | 6.80E-07 | 1.25E-06 | 1.51E-06 |
| 73 | 0.00E+00 | 0.00E+00 | 0.00E+00 | 0.00E+00 | 0.00E+00 | 2.21E-07 | 6.46E-07 | 1.19E-06 | 1.47E-06 |
| 74 | 0.00E+00 | 0.00E+00 | 0.00E+00 | 0.00E+00 | 0.00E+00 | 1.90E-07 | 6.16E-07 | 1.16E-06 | 1.44E-06 |
| 75 | 0.00E+00 | 0.00E+00 | 0.00E+00 | 0.00E+00 | 0.00E+00 | 1.60E-07 | 5.88E-07 | 1.13E-06 | 1.40E-06 |
| 76 | 0.00E+00 | 0.00E+00 | 0.00E+00 | 0.00E+00 | 0.00E+00 | 1.30E-07 | 5.63E-07 | 1.10E-06 | 1.36E-06 |
| 77 | 0.00E+00 | 0.00E+00 | 0.00E+00 | 0.00E+00 | 0.00E+00 | 1.02E-07 | 5.41E-07 | 1.07E-06 | 1.33E-06 |
| 78 | 0.00E+00 | 0.00E+00 | 0.00E+00 | 0.00E+00 | 0.00E+00 | 7.32E-08 | 5.19E-07 | 1.03E-06 | 1.30E-06 |
| 79 | 0.00E+00 | 0.00E+00 | 0.00E+00 | 0.00E+00 | 0.00E+00 | 4.44E-08 | 4.97E-07 | 9.99E-07 | 1.27E-06 |
| 80 | 0.00E+00 | 0.00E+00 | 0.00E+00 | 0.00E+00 | 0.00E+00 | 1.53E-08 | 4.73E-07 | 9.61E-07 | 1.25E-06 |
| 81 | 0.00E+00 | 0.00E+00 | 0.00E+00 | 0.00E+00 | 0.00E+00 | 0.00E+00 | 4.48E-07 | 9.25E-07 | 1.22E-06 |
| 82 | 0.00E+00 | 0.00E+00 | 0.00E+00 | 0.00E+00 | 0.00E+00 | 0.00E+00 | 4.22E-07 | 8.93E-07 | 1.19E-06 |
| 83 | 0.00E+00 | 0.00E+00 | 0.00E+00 | 0.00E+00 | 0.00E+00 | 0.00E+00 | 3.96E-07 | 8.62E-07 | 1.17E-06 |
| 84 | 0.00E+00 | 0.00E+00 | 0.00E+00 | 0.00E+00 | 0.00E+00 | 0.00E+00 | 3.70E-07 | 8.30E-07 | 1.14E-06 |
| 85 | 0.00E+00 | 0.00E+00 | 0.00E+00 | 0.00E+00 | 0.00E+00 | 0.00E+00 | 3.47E-07 | 8.05E-07 | 1.11E-06 |
| 86 | 0.00E+00 | 0.00E+00 | 0.00E+00 | 0.00E+00 | 0.00E+00 | 0.00E+00 | 3.23E-07 | 7.82E-07 | 1.09E-06 |
| 87 | 0.00E+00 | 0.00E+00 | 0.00E+00 | 0.00E+00 | 0.00E+00 | 0.00E+00 | 2.98E-07 | 7.60E-07 | 1.06E-06 |
| 88 | 0.00E+00 | 0.00E+00 | 0.00E+00 | 0.00E+00 | 0.00E+00 | 0.00E+00 | 2.72E-07 | 7.40E-07 | 1.03E-06 |
| 89 | 0.00E+00 | 0.00E+00 | 0.00E+00 | 0.00E+00 | 0.00E+00 | 0.00E+00 | 2.47E-07 | 7.20E-07 | 1.01E-06 |
| 90 | 0.00E+00 | 0.00E+00 | 0.00E+00 | 0.00E+00 | 0.00E+00 | 0.00E+00 | 2.22E-07 | 6.99E-07 | 9.85E-07 |
| 91 | 0.00E+00 | 0.00E+00 | 0.00E+00 | 0.00E+00 | 0.00E+00 | 0.00E+00 | 1.99E-07 | 6.78E-07 | 9.62E-07 |
| 92 | 0.00E+00 | 0.00E+00 | 0.00E+00 | 0.00E+00 | 0.00E+00 | 0.00E+00 | 1.78E-07 | 6.56E-07 | 9.37E-07 |
| 93 | 0.00E+00 | 0.00E+00 | 0.00E+00 | 0.00E+00 | 0.00E+00 | 0.00E+00 | 1.58E-07 | 6.35E-07 | 9.12E-07 |
| 94 | 0.00E+00 | 0.00E+00 | 0.00E+00 | 0.00E+00 | 0.00E+00 | 0.00E+00 | 1.39E-07 | 6.13E-07 | 8.84E-07 |
| 95 | 0.00E+00 | 0.00E+00 | 0.00E+00 | 0.00E+00 | 0.00E+00 | 0.00E+00 | 1.18E-07 | 5.90E-07 | 8.54E-07 |
| 96 | 0.00E+00 | 0.00E+00 | 0.00E+00 | 0.00E+00 | 0.00E+00 | 0.00E+00 | 9.70E-08 | 5.65E-07 | 8.26E-07 |
| 97 | 0.00E+00 | 0.00E+00 | 0.00E+00 | 0.00E+00 | 0.00E+00 | 0.00E+00 | 7.51E-08 | 5.40E-07 | 7.99E-07 |
| 98 | 0.00E+00 | 0.00E+00 | 0.00E+00 | 0.00E+00 | 0.00E+00 | 0.00E+00 | 5.32E-08 | 5.16E-07 | 7.75E-07 |
| 99 | 0.00E+00 | 0.00E+00 | 0.00E+00 | 0.00E+00 | 0.00E+00 | 0.00E+00 | 3.13E-08 | 4.95E-07 | 7.54E-07 |
| 100 | 0.00E+00 | 0.00E+00 | 0.00E+00 | 0.00E+00 | 0.00E+00 | 0.00E+00 | 1.24E-08 | 4.77E-07 | 7.36E-07 |
| 101 | 0.00E+00 | 0.00E+00 | 0.00E+00 | 0.00E+00 | 0.00E+00 | 0.00E+00 | 0.00E+00 | 4.62E-07 | 7.19E-07 |
| 102 | 0.00E+00 | 0.00E+00 | 0.00E+00 | 0.00E+00 | 0.00E+00 | 0.00E+00 | 0.00E+00 | 4.48E-07 | 7.01E-07 |
| 103 | 0.00E+00 | 0.00E+00 | 0.00E+00 | 0.00E+00 | 0.00E+00 | 0.00E+00 | 0.00E+00 | 4.30E-07 | 6.83E-07 |
| 104 | 0.00E+00 | 0.00E+00 | 0.00E+00 | 0.00E+00 | 0.00E+00 | 0.00E+00 | 0.00E+00 | 4.12E-07 | 6.64E-07 |
| 105 | 0.00E+00 | 0.00E+00 | 0.00E+00 | 0.00E+00 | 0.00E+00 | 0.00E+00 | 0.00E+00 | 3.92E-07 | 6.43E-07 |
| 106 | 0.00E+00 | 0.00E+00 | 0.00E+00 | 0.00E+00 | 0.00E+00 | 0.00E+00 | 0.00E+00 | 3.71E-07 | 6.23E-07 |
| 107 | 0.00E+00 | 0.00E+00 | 0.00E+00 | 0.00E+00 | 0.00E+00 | 0.00E+00 | 0.00E+00 | 3.50E-07 | 6.05E-07 |
| 108 | 0.00E+00 | 0.00E+00 | 0.00E+00 | 0.00E+00 | 0.00E+00 | 0.00E+00 | 0.00E+00 | 3.36E-07 | 5.88E-07 |
| 109 | 0.00E+00 | 0.00E+00 | 0.00E+00 | 0.00E+00 | 0.00E+00 | 0.00E+00 | 0.00E+00 | 3.20E-07 | 5.73E-07 |
| 110 | 0.00E+00 | 0.00E+00 | 0.00E+00 | 0.00E+00 | 0.00E+00 | 0.00E+00 | 0.00E+00 | 3.08E-07 | 5.60E-07 |
| 111 | 0.00E+00 | 0.00E+00 | 0.00E+00 | 0.00E+00 | 0.00E+00 | 0.00E+00 | 0.00E+00 | 2.94E-07 | 5.45E-07 |
| 112 | 0.00E+00 | 0.00E+00 | 0.00E+00 | 0.00E+00 | 0.00E+00 | 0.00E+00 | 0.00E+00 | 2.79E-07 | 5.30E-07 |
| 113 | 0.00E+00 | 0.00E+00 | 0.00E+00 | 0.00E+00 | 0.00E+00 | 0.00E+00 | 0.00E+00 | 2.58E-07 | 5.12E-07 |
| 114 | 0.00E+00 | 0.00E+00 | 0.00E+00 | 0.00E+00 | 0.00E+00 | 0.00E+00 | 0.00E+00 | 2.37E-07 | 4.92E-07 |
| 115 | 0.00E+00 | 0.00E+00 | 0.00E+00 | 0.00E+00 | 0.00E+00 | 0.00E+00 | 0.00E+00 | 2.15E-07 | 4.72E-07 |
| 116 | 0.00E+00 | 0.00E+00 | 0.00E+00 | 0.00E+00 | 0.00E+00 | 0.00E+00 | 0.00E+00 | 1.95E-07 | 4.50E-07 |
| 117 | 0.00E+00 | 0.00E+00 | 0.00E+00 | 0.00E+00 | 0.00E+00 | 0.00E+00 | 0.00E+00 | 1.77E-07 | 4.30E-07 |
| 118 | 0.00E+00 | 0.00E+00 | 0.00E+00 | 0.00E+00 | 0.00E+00 | 0.00E+00 | 0.00E+00 | 1.63E-07 | 4.11E-07 |
| 119 | 0.00E+00 | 0.00E+00 | 0.00E+00 | 0.00E+00 | 0.00E+00 | 0.00E+00 | 0.00E+00 | 1.50E-07 | 3.94E-07 |
| 120 | 0.00E+00 | 0.00E+00 | 0.00E+00 | 0.00E+00 | 0.00E+00 | 0.00E+00 | 0.00E+00 | 1.39E-07 | 3.79E-07 |
| 121 | 0.00E+00 | 0.00E+00 | 0.00E+00 | 0.00E+00 | 0.00E+00 | 0.00E+00 | 0.00E+00 | 1.27E-07 | 3.65E-07 |
| 122 | 0.00E+00 | 0.00E+00 | 0.00E+00 | 0.00E+00 | 0.00E+00 | 0.00E+00 | 0.00E+00 | 1.14E-07 | 3.52E-07 |
| 123 | 0.00E+00 | 0.00E+00 | 0.00E+00 | 0.00E+00 | 0.00E+00 | 0.00E+00 | 0.00E+00 | 9.96E-08 | 3.39E-07 |
| 124 | 0.00E+00 | 0.00E+00 | 0.00E+00 | 0.00E+00 | 0.00E+00 | 0.00E+00 | 0.00E+00 | 8.51E-08 | 3.26E-07 |
| 125 | 0.00E+00 | 0.00E+00 | 0.00E+00 | 0.00E+00 | 0.00E+00 | 0.00E+00 | 0.00E+00 | 7.10E-08 | 3.13E-07 |
| 126 | 0.00E+00 | 0.00E+00 | 0.00E+00 | 0.00E+00 | 0.00E+00 | 0.00E+00 | 0.00E+00 | 5.68E-08 | 2.99E-07 |
| 127 | 0.00E+00 | 0.00E+00 | 0.00E+00 | 0.00E+00 | 0.00E+00 | 0.00E+00 | 0.00E+00 | 4.39E-08 | 2.85E-07 |
| 128 | 0.00E+00 | 0.00E+00 | 0.00E+00 | 0.00E+00 | 0.00E+00 | 0.00E+00 | 0.00E+00 | 3.12E-08 | 2.69E-07 |
| 129 | 0.00E+00 | 0.00E+00 | 0.00E+00 | 0.00E+00 | 0.00E+00 | 0.00E+00 | 0.00E+00 | 1.99E-08 | 2.53E-07 |
| 130 | 0.00E+00 | 0.00E+00 | 0.00E+00 | 0.00E+00 | 0.00E+00 | 0.00E+00 | 0.00E+00 | 1.10E-08 | 2.37E-07 |
| 131 | 0.00E+00 | 0.00E+00 | 0.00E+00 | 0.00E+00 | 0.00E+00 | 0.00E+00 | 0.00E+00 | 0.00E+00 | 2.22E-07 |
| 132 | 0.00E+00 | 0.00E+00 | 0.00E+00 | 0.00E+00 | 0.00E+00 | 0.00E+00 | 0.00E+00 | 0.00E+00 | 2.08E-07 |
| 133 | 0.00E+00 | 0.00E+00 | 0.00E+00 | 0.00E+00 | 0.00E+00 | 0.00E+00 | 0.00E+00 | 0.00E+00 | 1.95E-07 |
| 134 | 0.00E+00 | 0.00E+00 | 0.00E+00 | 0.00E+00 | 0.00E+00 | 0.00E+00 | 0.00E+00 | 0.00E+00 | 1.82E-07 |
| 135 | 0.00E+00 | 0.00E+00 | 0.00E+00 | 0.00E+00 | 0.00E+00 | 0.00E+00 | 0.00E+00 | 0.00E+00 | 1.70E-07 |
| 136 | 0.00E+00 | 0.00E+00 | 0.00E+00 | 0.00E+00 | 0.00E+00 | 0.00E+00 | 0.00E+00 | 0.00E+00 | 1.59E-07 |
| 137 | 0.00E+00 | 0.00E+00 | 0.00E+00 | 0.00E+00 | 0.00E+00 | 0.00E+00 | 0.00E+00 | 0.00E+00 | 1.46E-07 |
| 138 | 0.00E+00 | 0.00E+00 | 0.00E+00 | 0.00E+00 | 0.00E+00 | 0.00E+00 | 0.00E+00 | 0.00E+00 | 1.34E-07 |
| 139 | 0.00E+00 | 0.00E+00 | 0.00E+00 | 0.00E+00 | 0.00E+00 | 0.00E+00 | 0.00E+00 | 0.00E+00 | 1.22E-07 |
| 140 | 0.00E+00 | 0.00E+00 | 0.00E+00 | 0.00E+00 | 0.00E+00 | 0.00E+00 | 0.00E+00 | 0.00E+00 | 1.11E-07 |
| 141 | 0.00E+00 | 0.00E+00 | 0.00E+00 | 0.00E+00 | 0.00E+00 | 0.00E+00 | 0.00E+00 | 0.00E+00 | 9.94E-08 |
| 142 | 0.00E+00 | 0.00E+00 | 0.00E+00 | 0.00E+00 | 0.00E+00 | 0.00E+00 | 0.00E+00 | 0.00E+00 | 8.87E-08 |
| 143 | 0.00E+00 | 0.00E+00 | 0.00E+00 | 0.00E+00 | 0.00E+00 | 0.00E+00 | 0.00E+00 | 0.00E+00 | 7.86E-08 |
| 144 | 0.00E+00 | 0.00E+00 | 0.00E+00 | 0.00E+00 | 0.00E+00 | 0.00E+00 | 0.00E+00 | 0.00E+00 | 6.87E-08 |
| 145 | 0.00E+00 | 0.00E+00 | 0.00E+00 | 0.00E+00 | 0.00E+00 | 0.00E+00 | 0.00E+00 | 0.00E+00 | 5.88E-08 |
| 146 | 0.00E+00 | 0.00E+00 | 0.00E+00 | 0.00E+00 | 0.00E+00 | 0.00E+00 | 0.00E+00 | 0.00E+00 | 4.88E-08 |
| 147 | 0.00E+00 | 0.00E+00 | 0.00E+00 | 0.00E+00 | 0.00E+00 | 0.00E+00 | 0.00E+00 | 0.00E+00 | 3.84E-08 |
| 148 | 0.00E+00 | 0.00E+00 | 0.00E+00 | 0.00E+00 | 0.00E+00 | 0.00E+00 | 0.00E+00 | 0.00E+00 | 2.73E-08 |
| 149 | 0.00E+00 | 0.00E+00 | 0.00E+00 | 0.00E+00 | 0.00E+00 | 0.00E+00 | 0.00E+00 | 0.00E+00 | 1.60E-08 |
| 150 | 0.00E+00 | 0.00E+00 | 0.00E+00 | 0.00E+00 | 0.00E+00 | 0.00E+00 | 0.00E+00 | 0.00E+00 | 3.80E-09 |

Table.3. Spectra obtained by MCNP code: Be- 0.8mm

| energy bin (kev)/voltage(kv) | 20kv | 30kv | 40kv | 50kv | 60kv | 80kv | 100kv | 130kv | 150kv |
| --- | --- | --- | --- | --- | --- | --- | --- | --- | --- |
| 1 | 0.00E+00 | 0.00E+00 | 0.00E+00 | 0.00E+00 | 0.00E+00 | 0.00E+00 | 0.00E+00 | 0.00E+00 | 0.00E+00 |
| 2 | 3.82E-17 | 2.97E-17 | 3.76E-17 | 1.03E-17 | 2.27E-17 | 3.92E-17 | 5.96E-17 | 1.36E-16 | 7.52E-17 |
| 3 | 1.03E-08 | 1.44E-09 | 1.24E-09 | 1.22E-09 | 1.15E-09 | 1.11E-09 | 1.15E-09 | 1.53E-09 | 1.71E-09 |
| 4 | 3.07E-07 | 1.93E-07 | 1.80E-07 | 1.62E-07 | 1.49E-07 | 1.38E-07 | 1.33E-07 | 1.57E-07 | 1.37E-07 |
| 5 | 8.96E-07 | 1.20E-06 | 1.18E-06 | 1.14E-06 | 1.05E-06 | 9.43E-07 | 8.90E-07 | 9.79E-07 | 8.22E-07 |
| 6 | 1.57E-06 | 2.49E-06 | 2.71E-06 | 2.72E-06 | 2.65E-06 | 2.43E-06 | 2.22E-06 | 2.26E-06 | 1.95E-06 |
| 7 | 2.12E-06 | 3.30E-06 | 3.78E-06 | 4.03E-06 | 4.10E-06 | 3.88E-06 | 3.71E-06 | 3.86E-06 | 3.17E-06 |
| 8 | 2.42E-06 | 3.77E-06 | 4.56E-06 | 5.13E-06 | 5.40E-06 | 5.54E-06 | 5.33E-06 | 5.60E-06 | 4.83E-06 |
| 9 | 2.35E-06 | 4.82E-06 | 6.62E-06 | 8.18E-06 | 9.30E-06 | 1.09E-05 | 1.18E-05 | 1.37E-05 | 1.21E-05 |
| 10 | 2.18E-06 | 3.65E-06 | 4.79E-06 | 5.83E-06 | 6.59E-06 | 7.50E-06 | 8.04E-06 | 9.09E-06 | 8.03E-06 |
| 11 | 1.91E-06 | 2.89E-06 | 3.73E-06 | 4.27E-06 | 4.64E-06 | 4.91E-06 | 4.91E-06 | 5.40E-06 | 4.60E-06 |
| 12 | 1.57E-06 | 2.60E-06 | 3.27E-06 | 3.76E-06 | 4.00E-06 | 4.14E-06 | 4.15E-06 | 4.39E-06 | 3.63E-06 |
| 13 | 1.31E-06 | 2.26E-06 | 2.87E-06 | 3.25E-06 | 3.49E-06 | 3.47E-06 | 3.47E-06 | 3.54E-06 | 3.02E-06 |
| 14 | 1.11E-06 | 2.09E-06 | 2.74E-06 | 3.18E-06 | 3.54E-06 | 3.75E-06 | 3.82E-06 | 3.94E-06 | 3.36E-06 |
| 15 | 9.30E-07 | 1.95E-06 | 2.64E-06 | 3.16E-06 | 3.52E-06 | 3.85E-06 | 3.99E-06 | 4.27E-06 | 3.70E-06 |
| 16 | 7.61E-07 | 1.80E-06 | 2.51E-06 | 3.08E-06 | 3.47E-06 | 3.91E-06 | 4.13E-06 | 4.53E-06 | 3.98E-06 |
| 17 | 5.92E-07 | 1.65E-06 | 2.39E-06 | 3.00E-06 | 3.41E-06 | 3.96E-06 | 4.26E-06 | 4.77E-06 | 4.26E-06 |
| 18 | 4.27E-07 | 1.50E-06 | 2.25E-06 | 2.89E-06 | 3.32E-06 | 3.97E-06 | 4.36E-06 | 4.96E-06 | 4.51E-06 |
| 19 | 2.63E-07 | 1.36E-06 | 2.11E-06 | 2.76E-06 | 3.23E-06 | 3.96E-06 | 4.42E-06 | 5.11E-06 | 4.71E-06 |
| 20 | 9.96E-08 | 1.21E-06 | 1.96E-06 | 2.63E-06 | 3.12E-06 | 3.91E-06 | 4.45E-06 | 5.22E-06 | 4.89E-06 |
| 21 | 0.00E+00 | 1.08E-06 | 1.83E-06 | 2.49E-06 | 3.01E-06 | 3.84E-06 | 4.45E-06 | 5.28E-06 | 5.02E-06 |
| 22 | 0.00E+00 | 9.53E-07 | 1.69E-06 | 2.36E-06 | 2.89E-06 | 3.75E-06 | 4.42E-06 | 5.31E-06 | 5.12E-06 |
| 23 | 0.00E+00 | 8.32E-07 | 1.56E-06 | 2.22E-06 | 2.77E-06 | 3.64E-06 | 4.37E-06 | 5.30E-06 | 5.19E-06 |
| 24 | 0.00E+00 | 7.16E-07 | 1.44E-06 | 2.09E-06 | 2.65E-06 | 3.54E-06 | 4.30E-06 | 5.26E-06 | 5.24E-06 |
| 25 | 0.00E+00 | 6.04E-07 | 1.32E-06 | 1.96E-06 | 2.52E-06 | 3.43E-06 | 4.22E-06 | 5.21E-06 | 5.26E-06 |
| 26 | 0.00E+00 | 4.96E-07 | 1.21E-06 | 1.84E-06 | 2.40E-06 | 3.32E-06 | 4.12E-06 | 5.15E-06 | 5.25E-06 |
| 27 | 0.00E+00 | 3.90E-07 | 1.11E-06 | 1.72E-06 | 2.28E-06 | 3.21E-06 | 4.01E-06 | 5.07E-06 | 5.23E-06 |
| 28 | 0.00E+00 | 2.82E-07 | 1.01E-06 | 1.61E-06 | 2.15E-06 | 3.10E-06 | 3.90E-06 | 4.97E-06 | 5.19E-06 |
| 29 | 0.00E+00 | 1.75E-07 | 9.16E-07 | 1.50E-06 | 2.04E-06 | 2.98E-06 | 3.79E-06 | 4.87E-06 | 5.14E-06 |
| 30 | 0.00E+00 | 6.30E-08 | 8.24E-07 | 1.40E-06 | 1.93E-06 | 2.87E-06 | 3.69E-06 | 4.74E-06 | 5.07E-06 |
| 31 | 0.00E+00 | 0.00E+00 | 7.37E-07 | 1.31E-06 | 1.82E-06 | 2.75E-06 | 3.58E-06 | 4.60E-06 | 5.01E-06 |
| 32 | 0.00E+00 | 0.00E+00 | 6.51E-07 | 1.22E-06 | 1.72E-06 | 2.63E-06 | 3.47E-06 | 4.47E-06 | 4.93E-06 |
| 33 | 0.00E+00 | 0.00E+00 | 5.69E-07 | 1.14E-06 | 1.63E-06 | 2.52E-06 | 3.36E-06 | 4.33E-06 | 4.85E-06 |
| 34 | 0.00E+00 | 0.00E+00 | 4.90E-07 | 1.05E-06 | 1.54E-06 | 2.40E-06 | 3.24E-06 | 4.20E-06 | 4.76E-06 |
| 35 | 0.00E+00 | 0.00E+00 | 4.11E-07 | 9.75E-07 | 1.45E-06 | 2.29E-06 | 3.13E-06 | 4.09E-06 | 4.66E-06 |
| 36 | 0.00E+00 | 0.00E+00 | 3.35E-07 | 8.99E-07 | 1.37E-06 | 2.17E-06 | 3.01E-06 | 3.97E-06 | 4.55E-06 |
| 37 | 0.00E+00 | 0.00E+00 | 2.60E-07 | 8.24E-07 | 1.29E-06 | 2.07E-06 | 2.89E-06 | 3.87E-06 | 4.43E-06 |
| 38 | 0.00E+00 | 0.00E+00 | 1.88E-07 | 7.53E-07 | 1.21E-06 | 1.98E-06 | 2.78E-06 | 3.76E-06 | 4.32E-06 |
| 39 | 0.00E+00 | 0.00E+00 | 1.16E-07 | 6.81E-07 | 1.13E-06 | 1.89E-06 | 2.67E-06 | 3.66E-06 | 4.20E-06 |
| 40 | 0.00E+00 | 0.00E+00 | 5.04E-08 | 6.12E-07 | 1.06E-06 | 1.82E-06 | 2.57E-06 | 3.55E-06 | 4.09E-06 |
| 41 | 0.00E+00 | 0.00E+00 | 0.00E+00 | 5.48E-07 | 9.87E-07 | 1.75E-06 | 2.47E-06 | 3.45E-06 | 3.98E-06 |
| 42 | 0.00E+00 | 0.00E+00 | 0.00E+00 | 4.85E-07 | 9.24E-07 | 1.68E-06 | 2.38E-06 | 3.34E-06 | 3.88E-06 |
| 43 | 0.00E+00 | 0.00E+00 | 0.00E+00 | 4.23E-07 | 8.64E-07 | 1.61E-06 | 2.29E-06 | 3.24E-06 | 3.78E-06 |
| 44 | 0.00E+00 | 0.00E+00 | 0.00E+00 | 3.67E-07 | 8.04E-07 | 1.54E-06 | 2.22E-06 | 3.14E-06 | 3.68E-06 |
| 45 | 0.00E+00 | 0.00E+00 | 0.00E+00 | 3.10E-07 | 7.46E-07 | 1.47E-06 | 2.14E-06 | 3.04E-06 | 3.58E-06 |
| 46 | 0.00E+00 | 0.00E+00 | 0.00E+00 | 2.55E-07 | 6.89E-07 | 1.40E-06 | 2.07E-06 | 2.96E-06 | 3.48E-06 |
| 47 | 0.00E+00 | 0.00E+00 | 0.00E+00 | 2.01E-07 | 6.35E-07 | 1.34E-06 | 1.99E-06 | 2.86E-06 | 3.39E-06 |
| 48 | 0.00E+00 | 0.00E+00 | 0.00E+00 | 1.47E-07 | 5.82E-07 | 1.28E-06 | 1.92E-06 | 2.77E-06 | 3.31E-06 |
| 49 | 0.00E+00 | 0.00E+00 | 0.00E+00 | 9.35E-08 | 5.32E-07 | 1.22E-06 | 1.85E-06 | 2.69E-06 | 3.22E-06 |
| 50 | 0.00E+00 | 0.00E+00 | 0.00E+00 | 3.87E-08 | 4.84E-07 | 1.16E-06 | 1.78E-06 | 2.60E-06 | 3.14E-06 |
| 51 | 0.00E+00 | 0.00E+00 | 0.00E+00 | 0.00E+00 | 4.36E-07 | 1.10E-06 | 1.71E-06 | 2.52E-06 | 3.05E-06 |
| 52 | 0.00E+00 | 0.00E+00 | 0.00E+00 | 0.00E+00 | 3.87E-07 | 1.04E-06 | 1.65E-06 | 2.45E-06 | 2.97E-06 |
| 53 | 0.00E+00 | 0.00E+00 | 0.00E+00 | 0.00E+00 | 3.40E-07 | 9.84E-07 | 1.60E-06 | 2.39E-06 | 2.90E-06 |
| 54 | 0.00E+00 | 0.00E+00 | 0.00E+00 | 0.00E+00 | 2.92E-07 | 9.31E-07 | 1.55E-06 | 2.33E-06 | 2.83E-06 |
| 55 | 0.00E+00 | 0.00E+00 | 0.00E+00 | 0.00E+00 | 2.47E-07 | 8.81E-07 | 1.50E-06 | 2.27E-06 | 2.76E-06 |
| 56 | 0.00E+00 | 0.00E+00 | 0.00E+00 | 0.00E+00 | 2.02E-07 | 8.37E-07 | 1.44E-06 | 2.20E-06 | 2.70E-06 |
| 57 | 0.00E+00 | 0.00E+00 | 0.00E+00 | 0.00E+00 | 1.57E-07 | 7.94E-07 | 1.38E-06 | 2.13E-06 | 2.64E-06 |
| 58 | 0.00E+00 | 0.00E+00 | 0.00E+00 | 0.00E+00 | 1.12E-07 | 7.56E-07 | 1.29E-06 | 2.03E-06 | 2.56E-06 |
| 59 | 0.00E+00 | 0.00E+00 | 0.00E+00 | 0.00E+00 | 7.10E-08 | 8.67E-07 | 2.29E-06 | 4.94E-06 | 6.68E-06 |
| 60 | 0.00E+00 | 0.00E+00 | 0.00E+00 | 0.00E+00 | 2.74E-08 | 9.62E-07 | 3.02E-06 | 7.26E-06 | 1.01E-05 |
| 61 | 0.00E+00 | 0.00E+00 | 0.00E+00 | 0.00E+00 | 0.00E+00 | 6.22E-07 | 1.12E-06 | 1.90E-06 | 2.43E-06 |
| 62 | 0.00E+00 | 0.00E+00 | 0.00E+00 | 0.00E+00 | 0.00E+00 | 6.07E-07 | 1.21E-06 | 1.98E-06 | 2.61E-06 |
| 63 | 0.00E+00 | 0.00E+00 | 0.00E+00 | 0.00E+00 | 0.00E+00 | 6.05E-07 | 1.06E-06 | 1.65E-06 | 2.13E-06 |
| 64 | 0.00E+00 | 0.00E+00 | 0.00E+00 | 0.00E+00 | 0.00E+00 | 5.23E-07 | 1.04E-06 | 1.58E-06 | 2.07E-06 |
| 65 | 0.00E+00 | 0.00E+00 | 0.00E+00 | 0.00E+00 | 0.00E+00 | 4.88E-07 | 9.92E-07 | 1.58E-06 | 2.05E-06 |
| 66 | 0.00E+00 | 0.00E+00 | 0.00E+00 | 0.00E+00 | 0.00E+00 | 4.58E-07 | 9.33E-07 | 1.55E-06 | 2.02E-06 |
| 67 | 0.00E+00 | 0.00E+00 | 0.00E+00 | 0.00E+00 | 0.00E+00 | 4.35E-07 | 8.60E-07 | 1.50E-06 | 1.98E-06 |
| 68 | 0.00E+00 | 0.00E+00 | 0.00E+00 | 0.00E+00 | 0.00E+00 | 4.93E-07 | 1.54E-06 | 3.30E-06 | 4.68E-06 |
| 69 | 0.00E+00 | 0.00E+00 | 0.00E+00 | 0.00E+00 | 0.00E+00 | 3.45E-07 | 8.16E-07 | 1.46E-06 | 1.79E-06 |
| 70 | 0.00E+00 | 0.00E+00 | 0.00E+00 | 0.00E+00 | 0.00E+00 | 3.34E-07 | 9.58E-07 | 1.87E-06 | 2.50E-06 |
| 71 | 0.00E+00 | 0.00E+00 | 0.00E+00 | 0.00E+00 | 0.00E+00 | 2.82E-07 | 7.57E-07 | 1.26E-06 | 1.49E-06 |
| 72 | 0.00E+00 | 0.00E+00 | 0.00E+00 | 0.00E+00 | 0.00E+00 | 2.53E-07 | 6.73E-07 | 1.23E-06 | 1.50E-06 |
| 73 | 0.00E+00 | 0.00E+00 | 0.00E+00 | 0.00E+00 | 0.00E+00 | 2.19E-07 | 6.39E-07 | 1.18E-06 | 1.46E-06 |
| 74 | 0.00E+00 | 0.00E+00 | 0.00E+00 | 0.00E+00 | 0.00E+00 | 1.88E-07 | 6.10E-07 | 1.15E-06 | 1.42E-06 |
| 75 | 0.00E+00 | 0.00E+00 | 0.00E+00 | 0.00E+00 | 0.00E+00 | 1.58E-07 | 5.82E-07 | 1.12E-06 | 1.38E-06 |
| 76 | 0.00E+00 | 0.00E+00 | 0.00E+00 | 0.00E+00 | 0.00E+00 | 1.29E-07 | 5.57E-07 | 1.09E-06 | 1.35E-06 |
| 77 | 0.00E+00 | 0.00E+00 | 0.00E+00 | 0.00E+00 | 0.00E+00 | 1.00E-07 | 5.35E-07 | 1.06E-06 | 1.32E-06 |
| 78 | 0.00E+00 | 0.00E+00 | 0.00E+00 | 0.00E+00 | 0.00E+00 | 7.24E-08 | 5.14E-07 | 1.02E-06 | 1.29E-06 |
| 79 | 0.00E+00 | 0.00E+00 | 0.00E+00 | 0.00E+00 | 0.00E+00 | 4.39E-08 | 4.92E-07 | 9.89E-07 | 1.26E-06 |
| 80 | 0.00E+00 | 0.00E+00 | 0.00E+00 | 0.00E+00 | 0.00E+00 | 1.52E-08 | 4.68E-07 | 9.51E-07 | 1.23E-06 |
| 81 | 0.00E+00 | 0.00E+00 | 0.00E+00 | 0.00E+00 | 0.00E+00 | 0.00E+00 | 4.43E-07 | 9.15E-07 | 1.21E-06 |
| 82 | 0.00E+00 | 0.00E+00 | 0.00E+00 | 0.00E+00 | 0.00E+00 | 0.00E+00 | 4.17E-07 | 8.84E-07 | 1.18E-06 |
| 83 | 0.00E+00 | 0.00E+00 | 0.00E+00 | 0.00E+00 | 0.00E+00 | 0.00E+00 | 3.92E-07 | 8.53E-07 | 1.16E-06 |
| 84 | 0.00E+00 | 0.00E+00 | 0.00E+00 | 0.00E+00 | 0.00E+00 | 0.00E+00 | 3.66E-07 | 8.22E-07 | 1.13E-06 |
| 85 | 0.00E+00 | 0.00E+00 | 0.00E+00 | 0.00E+00 | 0.00E+00 | 0.00E+00 | 3.43E-07 | 7.97E-07 | 1.10E-06 |
| 86 | 0.00E+00 | 0.00E+00 | 0.00E+00 | 0.00E+00 | 0.00E+00 | 0.00E+00 | 3.20E-07 | 7.74E-07 | 1.08E-06 |
| 87 | 0.00E+00 | 0.00E+00 | 0.00E+00 | 0.00E+00 | 0.00E+00 | 0.00E+00 | 2.95E-07 | 7.53E-07 | 1.05E-06 |
| 88 | 0.00E+00 | 0.00E+00 | 0.00E+00 | 0.00E+00 | 0.00E+00 | 0.00E+00 | 2.70E-07 | 7.33E-07 | 1.02E-06 |
| 89 | 0.00E+00 | 0.00E+00 | 0.00E+00 | 0.00E+00 | 0.00E+00 | 0.00E+00 | 2.45E-07 | 7.13E-07 | 9.99E-07 |
| 90 | 0.00E+00 | 0.00E+00 | 0.00E+00 | 0.00E+00 | 0.00E+00 | 0.00E+00 | 2.20E-07 | 6.92E-07 | 9.76E-07 |
| 91 | 0.00E+00 | 0.00E+00 | 0.00E+00 | 0.00E+00 | 0.00E+00 | 0.00E+00 | 1.97E-07 | 6.71E-07 | 9.53E-07 |
| 92 | 0.00E+00 | 0.00E+00 | 0.00E+00 | 0.00E+00 | 0.00E+00 | 0.00E+00 | 1.76E-07 | 6.50E-07 | 9.28E-07 |
| 93 | 0.00E+00 | 0.00E+00 | 0.00E+00 | 0.00E+00 | 0.00E+00 | 0.00E+00 | 1.57E-07 | 6.29E-07 | 9.03E-07 |
| 94 | 0.00E+00 | 0.00E+00 | 0.00E+00 | 0.00E+00 | 0.00E+00 | 0.00E+00 | 1.38E-07 | 6.07E-07 | 8.75E-07 |
| 95 | 0.00E+00 | 0.00E+00 | 0.00E+00 | 0.00E+00 | 0.00E+00 | 0.00E+00 | 1.17E-07 | 5.84E-07 | 8.46E-07 |
| 96 | 0.00E+00 | 0.00E+00 | 0.00E+00 | 0.00E+00 | 0.00E+00 | 0.00E+00 | 9.60E-08 | 5.59E-07 | 8.17E-07 |
| 97 | 0.00E+00 | 0.00E+00 | 0.00E+00 | 0.00E+00 | 0.00E+00 | 0.00E+00 | 7.44E-08 | 5.35E-07 | 7.92E-07 |
| 98 | 0.00E+00 | 0.00E+00 | 0.00E+00 | 0.00E+00 | 0.00E+00 | 0.00E+00 | 5.27E-08 | 5.11E-07 | 7.67E-07 |
| 99 | 0.00E+00 | 0.00E+00 | 0.00E+00 | 0.00E+00 | 0.00E+00 | 0.00E+00 | 3.09E-08 | 4.90E-07 | 7.46E-07 |
| 100 | 0.00E+00 | 0.00E+00 | 0.00E+00 | 0.00E+00 | 0.00E+00 | 0.00E+00 | 1.23E-08 | 4.72E-07 | 7.29E-07 |
| 101 | 0.00E+00 | 0.00E+00 | 0.00E+00 | 0.00E+00 | 0.00E+00 | 0.00E+00 | 0.00E+00 | 4.58E-07 | 7.12E-07 |
| 102 | 0.00E+00 | 0.00E+00 | 0.00E+00 | 0.00E+00 | 0.00E+00 | 0.00E+00 | 0.00E+00 | 4.43E-07 | 6.94E-07 |
| 103 | 0.00E+00 | 0.00E+00 | 0.00E+00 | 0.00E+00 | 0.00E+00 | 0.00E+00 | 0.00E+00 | 4.25E-07 | 6.77E-07 |
| 104 | 0.00E+00 | 0.00E+00 | 0.00E+00 | 0.00E+00 | 0.00E+00 | 0.00E+00 | 0.00E+00 | 4.08E-07 | 6.57E-07 |
| 105 | 0.00E+00 | 0.00E+00 | 0.00E+00 | 0.00E+00 | 0.00E+00 | 0.00E+00 | 0.00E+00 | 3.88E-07 | 6.37E-07 |
| 106 | 0.00E+00 | 0.00E+00 | 0.00E+00 | 0.00E+00 | 0.00E+00 | 0.00E+00 | 0.00E+00 | 3.67E-07 | 6.17E-07 |
| 107 | 0.00E+00 | 0.00E+00 | 0.00E+00 | 0.00E+00 | 0.00E+00 | 0.00E+00 | 0.00E+00 | 3.47E-07 | 5.99E-07 |
| 108 | 0.00E+00 | 0.00E+00 | 0.00E+00 | 0.00E+00 | 0.00E+00 | 0.00E+00 | 0.00E+00 | 3.33E-07 | 5.83E-07 |
| 109 | 0.00E+00 | 0.00E+00 | 0.00E+00 | 0.00E+00 | 0.00E+00 | 0.00E+00 | 0.00E+00 | 3.17E-07 | 5.68E-07 |
| 110 | 0.00E+00 | 0.00E+00 | 0.00E+00 | 0.00E+00 | 0.00E+00 | 0.00E+00 | 0.00E+00 | 3.05E-07 | 5.54E-07 |
| 111 | 0.00E+00 | 0.00E+00 | 0.00E+00 | 0.00E+00 | 0.00E+00 | 0.00E+00 | 0.00E+00 | 2.91E-07 | 5.40E-07 |
| 112 | 0.00E+00 | 0.00E+00 | 0.00E+00 | 0.00E+00 | 0.00E+00 | 0.00E+00 | 0.00E+00 | 2.76E-07 | 5.25E-07 |
| 113 | 0.00E+00 | 0.00E+00 | 0.00E+00 | 0.00E+00 | 0.00E+00 | 0.00E+00 | 0.00E+00 | 2.55E-07 | 5.07E-07 |
| 114 | 0.00E+00 | 0.00E+00 | 0.00E+00 | 0.00E+00 | 0.00E+00 | 0.00E+00 | 0.00E+00 | 2.35E-07 | 4.88E-07 |
| 115 | 0.00E+00 | 0.00E+00 | 0.00E+00 | 0.00E+00 | 0.00E+00 | 0.00E+00 | 0.00E+00 | 2.13E-07 | 4.67E-07 |
| 116 | 0.00E+00 | 0.00E+00 | 0.00E+00 | 0.00E+00 | 0.00E+00 | 0.00E+00 | 0.00E+00 | 1.93E-07 | 4.46E-07 |
| 117 | 0.00E+00 | 0.00E+00 | 0.00E+00 | 0.00E+00 | 0.00E+00 | 0.00E+00 | 0.00E+00 | 1.75E-07 | 4.25E-07 |
| 118 | 0.00E+00 | 0.00E+00 | 0.00E+00 | 0.00E+00 | 0.00E+00 | 0.00E+00 | 0.00E+00 | 1.61E-07 | 4.07E-07 |
| 119 | 0.00E+00 | 0.00E+00 | 0.00E+00 | 0.00E+00 | 0.00E+00 | 0.00E+00 | 0.00E+00 | 1.49E-07 | 3.91E-07 |
| 120 | 0.00E+00 | 0.00E+00 | 0.00E+00 | 0.00E+00 | 0.00E+00 | 0.00E+00 | 0.00E+00 | 1.37E-07 | 3.75E-07 |
| 121 | 0.00E+00 | 0.00E+00 | 0.00E+00 | 0.00E+00 | 0.00E+00 | 0.00E+00 | 0.00E+00 | 1.25E-07 | 3.61E-07 |
| 122 | 0.00E+00 | 0.00E+00 | 0.00E+00 | 0.00E+00 | 0.00E+00 | 0.00E+00 | 0.00E+00 | 1.13E-07 | 3.48E-07 |
| 123 | 0.00E+00 | 0.00E+00 | 0.00E+00 | 0.00E+00 | 0.00E+00 | 0.00E+00 | 0.00E+00 | 9.87E-08 | 3.36E-07 |
| 124 | 0.00E+00 | 0.00E+00 | 0.00E+00 | 0.00E+00 | 0.00E+00 | 0.00E+00 | 0.00E+00 | 8.43E-08 | 3.23E-07 |
| 125 | 0.00E+00 | 0.00E+00 | 0.00E+00 | 0.00E+00 | 0.00E+00 | 0.00E+00 | 0.00E+00 | 7.03E-08 | 3.10E-07 |
| 126 | 0.00E+00 | 0.00E+00 | 0.00E+00 | 0.00E+00 | 0.00E+00 | 0.00E+00 | 0.00E+00 | 5.63E-08 | 2.96E-07 |
| 127 | 0.00E+00 | 0.00E+00 | 0.00E+00 | 0.00E+00 | 0.00E+00 | 0.00E+00 | 0.00E+00 | 4.35E-08 | 2.82E-07 |
| 128 | 0.00E+00 | 0.00E+00 | 0.00E+00 | 0.00E+00 | 0.00E+00 | 0.00E+00 | 0.00E+00 | 3.09E-08 | 2.66E-07 |
| 129 | 0.00E+00 | 0.00E+00 | 0.00E+00 | 0.00E+00 | 0.00E+00 | 0.00E+00 | 0.00E+00 | 1.97E-08 | 2.51E-07 |
| 130 | 0.00E+00 | 0.00E+00 | 0.00E+00 | 0.00E+00 | 0.00E+00 | 0.00E+00 | 0.00E+00 | 1.09E-08 | 2.35E-07 |
| 131 | 0.00E+00 | 0.00E+00 | 0.00E+00 | 0.00E+00 | 0.00E+00 | 0.00E+00 | 0.00E+00 | 0.00E+00 | 2.20E-07 |
| 132 | 0.00E+00 | 0.00E+00 | 0.00E+00 | 0.00E+00 | 0.00E+00 | 0.00E+00 | 0.00E+00 | 0.00E+00 | 2.06E-07 |
| 133 | 0.00E+00 | 0.00E+00 | 0.00E+00 | 0.00E+00 | 0.00E+00 | 0.00E+00 | 0.00E+00 | 0.00E+00 | 1.93E-07 |
| 134 | 0.00E+00 | 0.00E+00 | 0.00E+00 | 0.00E+00 | 0.00E+00 | 0.00E+00 | 0.00E+00 | 0.00E+00 | 1.81E-07 |
| 135 | 0.00E+00 | 0.00E+00 | 0.00E+00 | 0.00E+00 | 0.00E+00 | 0.00E+00 | 0.00E+00 | 0.00E+00 | 1.69E-07 |
| 136 | 0.00E+00 | 0.00E+00 | 0.00E+00 | 0.00E+00 | 0.00E+00 | 0.00E+00 | 0.00E+00 | 0.00E+00 | 1.57E-07 |
| 137 | 0.00E+00 | 0.00E+00 | 0.00E+00 | 0.00E+00 | 0.00E+00 | 0.00E+00 | 0.00E+00 | 0.00E+00 | 1.45E-07 |
| 138 | 0.00E+00 | 0.00E+00 | 0.00E+00 | 0.00E+00 | 0.00E+00 | 0.00E+00 | 0.00E+00 | 0.00E+00 | 1.33E-07 |
| 139 | 0.00E+00 | 0.00E+00 | 0.00E+00 | 0.00E+00 | 0.00E+00 | 0.00E+00 | 0.00E+00 | 0.00E+00 | 1.21E-07 |
| 140 | 0.00E+00 | 0.00E+00 | 0.00E+00 | 0.00E+00 | 0.00E+00 | 0.00E+00 | 0.00E+00 | 0.00E+00 | 1.10E-07 |
| 141 | 0.00E+00 | 0.00E+00 | 0.00E+00 | 0.00E+00 | 0.00E+00 | 0.00E+00 | 0.00E+00 | 0.00E+00 | 9.85E-08 |
| 142 | 0.00E+00 | 0.00E+00 | 0.00E+00 | 0.00E+00 | 0.00E+00 | 0.00E+00 | 0.00E+00 | 0.00E+00 | 8.79E-08 |
| 143 | 0.00E+00 | 0.00E+00 | 0.00E+00 | 0.00E+00 | 0.00E+00 | 0.00E+00 | 0.00E+00 | 0.00E+00 | 7.79E-08 |
| 144 | 0.00E+00 | 0.00E+00 | 0.00E+00 | 0.00E+00 | 0.00E+00 | 0.00E+00 | 0.00E+00 | 0.00E+00 | 6.81E-08 |
| 145 | 0.00E+00 | 0.00E+00 | 0.00E+00 | 0.00E+00 | 0.00E+00 | 0.00E+00 | 0.00E+00 | 0.00E+00 | 5.83E-08 |
| 146 | 0.00E+00 | 0.00E+00 | 0.00E+00 | 0.00E+00 | 0.00E+00 | 0.00E+00 | 0.00E+00 | 0.00E+00 | 4.84E-08 |
| 147 | 0.00E+00 | 0.00E+00 | 0.00E+00 | 0.00E+00 | 0.00E+00 | 0.00E+00 | 0.00E+00 | 0.00E+00 | 3.80E-08 |
| 148 | 0.00E+00 | 0.00E+00 | 0.00E+00 | 0.00E+00 | 0.00E+00 | 0.00E+00 | 0.00E+00 | 0.00E+00 | 2.70E-08 |
| 149 | 0.00E+00 | 0.00E+00 | 0.00E+00 | 0.00E+00 | 0.00E+00 | 0.00E+00 | 0.00E+00 | 0.00E+00 | 1.59E-08 |
| 150 | 0.00E+00 | 0.00E+00 | 0.00E+00 | 0.00E+00 | 0.00E+00 | 0.00E+00 | 0.00E+00 | 0.00E+00 | 3.76E-09 |

Table.4. Spectra obtained by MCNP code: Be- 1.2mm

| energy bin (kev)/voltage(kv) | 20kv | 30kv | 40kv | 50kv | 60kv | 80kv | 100kv | 130kv | 150kv |
| --- | --- | --- | --- | --- | --- | --- | --- | --- | --- |
| 1 | 0.00E+00 | 0.00E+00 | 0.00E+00 | 0.00E+00 | 0.00E+00 | 0.00E+00 | 0.00E+00 | 0.00E+00 | 0.00E+00 |
| 2 | 1.52E-19 | 1.18E-19 | 1.49E-19 | 4.09E-20 | 9.04E-20 | 1.56E-19 | 2.37E-19 | 5.39E-19 | 2.99E-19 |
| 3 | 2.13E-09 | 2.98E-10 | 2.56E-10 | 2.52E-10 | 2.39E-10 | 2.30E-10 | 2.39E-10 | 3.16E-10 | 3.54E-10 |
| 4 | 1.61E-07 | 1.02E-07 | 9.47E-08 | 8.53E-08 | 7.84E-08 | 7.28E-08 | 6.97E-08 | 8.24E-08 | 7.21E-08 |
| 5 | 6.48E-07 | 8.72E-07 | 8.53E-07 | 8.26E-07 | 7.62E-07 | 6.82E-07 | 6.44E-07 | 7.09E-07 | 5.95E-07 |
| 6 | 1.31E-06 | 2.06E-06 | 2.25E-06 | 2.25E-06 | 2.20E-06 | 2.02E-06 | 1.84E-06 | 1.87E-06 | 1.61E-06 |
| 7 | 1.85E-06 | 2.88E-06 | 3.30E-06 | 3.52E-06 | 3.58E-06 | 3.39E-06 | 3.24E-06 | 3.37E-06 | 2.76E-06 |
| 8 | 2.23E-06 | 3.47E-06 | 4.20E-06 | 4.73E-06 | 4.97E-06 | 5.10E-06 | 4.91E-06 | 5.15E-06 | 4.45E-06 |
| 9 | 2.20E-06 | 4.51E-06 | 6.20E-06 | 7.66E-06 | 8.71E-06 | 1.02E-05 | 1.10E-05 | 1.28E-05 | 1.13E-05 |
| 10 | 2.08E-06 | 3.48E-06 | 4.56E-06 | 5.56E-06 | 6.28E-06 | 7.15E-06 | 7.67E-06 | 8.66E-06 | 7.66E-06 |
| 11 | 1.83E-06 | 2.77E-06 | 3.57E-06 | 4.09E-06 | 4.44E-06 | 4.70E-06 | 4.70E-06 | 5.17E-06 | 4.41E-06 |
| 12 | 1.51E-06 | 2.50E-06 | 3.15E-06 | 3.62E-06 | 3.85E-06 | 3.98E-06 | 4.00E-06 | 4.23E-06 | 3.50E-06 |
| 13 | 1.27E-06 | 2.18E-06 | 2.77E-06 | 3.14E-06 | 3.38E-06 | 3.35E-06 | 3.35E-06 | 3.43E-06 | 2.92E-06 |
| 14 | 1.08E-06 | 2.03E-06 | 2.67E-06 | 3.09E-06 | 3.44E-06 | 3.64E-06 | 3.72E-06 | 3.84E-06 | 3.27E-06 |
| 15 | 9.07E-07 | 1.90E-06 | 2.58E-06 | 3.09E-06 | 3.44E-06 | 3.76E-06 | 3.89E-06 | 4.17E-06 | 3.61E-06 |
| 16 | 7.44E-07 | 1.76E-06 | 2.46E-06 | 3.01E-06 | 3.39E-06 | 3.82E-06 | 4.04E-06 | 4.43E-06 | 3.90E-06 |
| 17 | 5.80E-07 | 1.62E-06 | 2.34E-06 | 2.94E-06 | 3.34E-06 | 3.88E-06 | 4.17E-06 | 4.67E-06 | 4.18E-06 |
| 18 | 4.19E-07 | 1.47E-06 | 2.21E-06 | 2.83E-06 | 3.26E-06 | 3.90E-06 | 4.27E-06 | 4.86E-06 | 4.42E-06 |
| 19 | 2.59E-07 | 1.33E-06 | 2.07E-06 | 2.71E-06 | 3.17E-06 | 3.89E-06 | 4.34E-06 | 5.02E-06 | 4.63E-06 |
| 20 | 9.80E-08 | 1.19E-06 | 1.93E-06 | 2.58E-06 | 3.07E-06 | 3.84E-06 | 4.37E-06 | 5.13E-06 | 4.80E-06 |
| 21 | 0.00E+00 | 1.06E-06 | 1.80E-06 | 2.45E-06 | 2.96E-06 | 3.78E-06 | 4.38E-06 | 5.19E-06 | 4.94E-06 |
| 22 | 0.00E+00 | 9.38E-07 | 1.67E-06 | 2.32E-06 | 2.84E-06 | 3.69E-06 | 4.35E-06 | 5.22E-06 | 5.04E-06 |
| 23 | 0.00E+00 | 8.19E-07 | 1.54E-06 | 2.18E-06 | 2.73E-06 | 3.59E-06 | 4.31E-06 | 5.22E-06 | 5.11E-06 |
| 24 | 0.00E+00 | 7.05E-07 | 1.42E-06 | 2.05E-06 | 2.61E-06 | 3.48E-06 | 4.24E-06 | 5.18E-06 | 5.16E-06 |
| 25 | 0.00E+00 | 5.95E-07 | 1.30E-06 | 1.93E-06 | 2.49E-06 | 3.38E-06 | 4.16E-06 | 5.13E-06 | 5.18E-06 |
| 26 | 0.00E+00 | 4.89E-07 | 1.19E-06 | 1.81E-06 | 2.37E-06 | 3.27E-06 | 4.06E-06 | 5.07E-06 | 5.18E-06 |
| 27 | 0.00E+00 | 3.84E-07 | 1.09E-06 | 1.70E-06 | 2.25E-06 | 3.16E-06 | 3.95E-06 | 4.99E-06 | 5.16E-06 |
| 28 | 0.00E+00 | 2.78E-07 | 9.96E-07 | 1.59E-06 | 2.12E-06 | 3.05E-06 | 3.85E-06 | 4.90E-06 | 5.12E-06 |
| 29 | 0.00E+00 | 1.73E-07 | 9.03E-07 | 1.48E-06 | 2.01E-06 | 2.94E-06 | 3.74E-06 | 4.80E-06 | 5.07E-06 |
| 30 | 0.00E+00 | 6.22E-08 | 8.13E-07 | 1.39E-06 | 1.90E-06 | 2.83E-06 | 3.64E-06 | 4.68E-06 | 5.01E-06 |
| 31 | 0.00E+00 | 0.00E+00 | 7.27E-07 | 1.29E-06 | 1.80E-06 | 2.72E-06 | 3.54E-06 | 4.54E-06 | 4.94E-06 |
| 32 | 0.00E+00 | 0.00E+00 | 6.42E-07 | 1.20E-06 | 1.69E-06 | 2.60E-06 | 3.43E-06 | 4.41E-06 | 4.87E-06 |
| 33 | 0.00E+00 | 0.00E+00 | 5.62E-07 | 1.12E-06 | 1.60E-06 | 2.49E-06 | 3.31E-06 | 4.28E-06 | 4.79E-06 |
| 34 | 0.00E+00 | 0.00E+00 | 4.84E-07 | 1.04E-06 | 1.52E-06 | 2.37E-06 | 3.20E-06 | 4.15E-06 | 4.70E-06 |
| 35 | 0.00E+00 | 0.00E+00 | 4.06E-07 | 9.63E-07 | 1.43E-06 | 2.26E-06 | 3.09E-06 | 4.04E-06 | 4.60E-06 |
| 36 | 0.00E+00 | 0.00E+00 | 3.31E-07 | 8.88E-07 | 1.35E-06 | 2.15E-06 | 2.97E-06 | 3.92E-06 | 4.49E-06 |
| 37 | 0.00E+00 | 0.00E+00 | 2.57E-07 | 8.13E-07 | 1.27E-06 | 2.05E-06 | 2.86E-06 | 3.82E-06 | 4.38E-06 |
| 38 | 0.00E+00 | 0.00E+00 | 1.86E-07 | 7.44E-07 | 1.19E-06 | 1.95E-06 | 2.75E-06 | 3.72E-06 | 4.26E-06 |
| 39 | 0.00E+00 | 0.00E+00 | 1.15E-07 | 6.73E-07 | 1.12E-06 | 1.87E-06 | 2.64E-06 | 3.61E-06 | 4.15E-06 |
| 40 | 0.00E+00 | 0.00E+00 | 4.98E-08 | 6.05E-07 | 1.04E-06 | 1.80E-06 | 2.54E-06 | 3.51E-06 | 4.04E-06 |
| 41 | 0.00E+00 | 0.00E+00 | 0.00E+00 | 5.41E-07 | 9.75E-07 | 1.73E-06 | 2.44E-06 | 3.41E-06 | 3.93E-06 |
| 42 | 0.00E+00 | 0.00E+00 | 0.00E+00 | 4.80E-07 | 9.13E-07 | 1.66E-06 | 2.35E-06 | 3.30E-06 | 3.83E-06 |
| 43 | 0.00E+00 | 0.00E+00 | 0.00E+00 | 4.18E-07 | 8.54E-07 | 1.59E-06 | 2.27E-06 | 3.20E-06 | 3.73E-06 |
| 44 | 0.00E+00 | 0.00E+00 | 0.00E+00 | 3.62E-07 | 7.95E-07 | 1.52E-06 | 2.19E-06 | 3.10E-06 | 3.63E-06 |
| 45 | 0.00E+00 | 0.00E+00 | 0.00E+00 | 3.07E-07 | 7.37E-07 | 1.45E-06 | 2.12E-06 | 3.01E-06 | 3.54E-06 |
| 46 | 0.00E+00 | 0.00E+00 | 0.00E+00 | 2.52E-07 | 6.81E-07 | 1.38E-06 | 2.04E-06 | 2.92E-06 | 3.44E-06 |
| 47 | 0.00E+00 | 0.00E+00 | 0.00E+00 | 1.99E-07 | 6.27E-07 | 1.32E-06 | 1.97E-06 | 2.83E-06 | 3.35E-06 |
| 48 | 0.00E+00 | 0.00E+00 | 0.00E+00 | 1.46E-07 | 5.75E-07 | 1.26E-06 | 1.90E-06 | 2.74E-06 | 3.27E-06 |
| 49 | 0.00E+00 | 0.00E+00 | 0.00E+00 | 9.25E-08 | 5.26E-07 | 1.20E-06 | 1.83E-06 | 2.65E-06 | 3.18E-06 |
| 50 | 0.00E+00 | 0.00E+00 | 0.00E+00 | 3.83E-08 | 4.78E-07 | 1.15E-06 | 1.76E-06 | 2.57E-06 | 3.10E-06 |
| 51 | 0.00E+00 | 0.00E+00 | 0.00E+00 | 0.00E+00 | 4.31E-07 | 1.09E-06 | 1.69E-06 | 2.49E-06 | 3.02E-06 |
| 52 | 0.00E+00 | 0.00E+00 | 0.00E+00 | 0.00E+00 | 3.83E-07 | 1.03E-06 | 1.63E-06 | 2.42E-06 | 2.94E-06 |
| 53 | 0.00E+00 | 0.00E+00 | 0.00E+00 | 0.00E+00 | 3.36E-07 | 9.73E-07 | 1.58E-06 | 2.36E-06 | 2.86E-06 |
| 54 | 0.00E+00 | 0.00E+00 | 0.00E+00 | 0.00E+00 | 2.89E-07 | 9.21E-07 | 1.53E-06 | 2.30E-06 | 2.80E-06 |
| 55 | 0.00E+00 | 0.00E+00 | 0.00E+00 | 0.00E+00 | 2.44E-07 | 8.71E-07 | 1.48E-06 | 2.24E-06 | 2.73E-06 |
| 56 | 0.00E+00 | 0.00E+00 | 0.00E+00 | 0.00E+00 | 2.00E-07 | 8.27E-07 | 1.42E-06 | 2.17E-06 | 2.67E-06 |
| 57 | 0.00E+00 | 0.00E+00 | 0.00E+00 | 0.00E+00 | 1.56E-07 | 7.85E-07 | 1.36E-06 | 2.10E-06 | 2.61E-06 |
| 58 | 0.00E+00 | 0.00E+00 | 0.00E+00 | 0.00E+00 | 1.11E-07 | 7.48E-07 | 1.28E-06 | 2.01E-06 | 2.53E-06 |
| 59 | 0.00E+00 | 0.00E+00 | 0.00E+00 | 0.00E+00 | 7.02E-08 | 8.57E-07 | 2.26E-06 | 4.88E-06 | 6.61E-06 |
| 60 | 0.00E+00 | 0.00E+00 | 0.00E+00 | 0.00E+00 | 2.71E-08 | 9.51E-07 | 2.99E-06 | 7.18E-06 | 9.96E-06 |
| 61 | 0.00E+00 | 0.00E+00 | 0.00E+00 | 0.00E+00 | 0.00E+00 | 6.15E-07 | 1.10E-06 | 1.88E-06 | 2.40E-06 |
| 62 | 0.00E+00 | 0.00E+00 | 0.00E+00 | 0.00E+00 | 0.00E+00 | 6.00E-07 | 1.20E-06 | 1.95E-06 | 2.58E-06 |
| 63 | 0.00E+00 | 0.00E+00 | 0.00E+00 | 0.00E+00 | 0.00E+00 | 5.98E-07 | 1.05E-06 | 1.64E-06 | 2.11E-06 |
| 64 | 0.00E+00 | 0.00E+00 | 0.00E+00 | 0.00E+00 | 0.00E+00 | 5.18E-07 | 1.03E-06 | 1.56E-06 | 2.05E-06 |
| 65 | 0.00E+00 | 0.00E+00 | 0.00E+00 | 0.00E+00 | 0.00E+00 | 4.82E-07 | 9.81E-07 | 1.56E-06 | 2.03E-06 |
| 66 | 0.00E+00 | 0.00E+00 | 0.00E+00 | 0.00E+00 | 0.00E+00 | 4.53E-07 | 9.23E-07 | 1.54E-06 | 2.00E-06 |
| 67 | 0.00E+00 | 0.00E+00 | 0.00E+00 | 0.00E+00 | 0.00E+00 | 4.31E-07 | 8.51E-07 | 1.48E-06 | 1.96E-06 |
| 68 | 0.00E+00 | 0.00E+00 | 0.00E+00 | 0.00E+00 | 0.00E+00 | 4.88E-07 | 1.52E-06 | 3.26E-06 | 4.63E-06 |
| 69 | 0.00E+00 | 0.00E+00 | 0.00E+00 | 0.00E+00 | 0.00E+00 | 3.41E-07 | 8.08E-07 | 1.44E-06 | 1.77E-06 |
| 70 | 0.00E+00 | 0.00E+00 | 0.00E+00 | 0.00E+00 | 0.00E+00 | 3.30E-07 | 9.48E-07 | 1.85E-06 | 2.47E-06 |
| 71 | 0.00E+00 | 0.00E+00 | 0.00E+00 | 0.00E+00 | 0.00E+00 | 2.79E-07 | 7.49E-07 | 1.25E-06 | 1.47E-06 |
| 72 | 0.00E+00 | 0.00E+00 | 0.00E+00 | 0.00E+00 | 0.00E+00 | 2.50E-07 | 6.65E-07 | 1.22E-06 | 1.48E-06 |
| 73 | 0.00E+00 | 0.00E+00 | 0.00E+00 | 0.00E+00 | 0.00E+00 | 2.16E-07 | 6.33E-07 | 1.17E-06 | 1.44E-06 |
| 74 | 0.00E+00 | 0.00E+00 | 0.00E+00 | 0.00E+00 | 0.00E+00 | 1.86E-07 | 6.03E-07 | 1.14E-06 | 1.41E-06 |
| 75 | 0.00E+00 | 0.00E+00 | 0.00E+00 | 0.00E+00 | 0.00E+00 | 1.56E-07 | 5.76E-07 | 1.10E-06 | 1.37E-06 |
| 76 | 0.00E+00 | 0.00E+00 | 0.00E+00 | 0.00E+00 | 0.00E+00 | 1.27E-07 | 5.51E-07 | 1.08E-06 | 1.34E-06 |
| 77 | 0.00E+00 | 0.00E+00 | 0.00E+00 | 0.00E+00 | 0.00E+00 | 9.94E-08 | 5.29E-07 | 1.04E-06 | 1.30E-06 |
| 78 | 0.00E+00 | 0.00E+00 | 0.00E+00 | 0.00E+00 | 0.00E+00 | 7.16E-08 | 5.08E-07 | 1.01E-06 | 1.28E-06 |
| 79 | 0.00E+00 | 0.00E+00 | 0.00E+00 | 0.00E+00 | 0.00E+00 | 4.35E-08 | 4.87E-07 | 9.79E-07 | 1.25E-06 |
| 80 | 0.00E+00 | 0.00E+00 | 0.00E+00 | 0.00E+00 | 0.00E+00 | 1.50E-08 | 4.63E-07 | 9.42E-07 | 1.22E-06 |
| 81 | 0.00E+00 | 0.00E+00 | 0.00E+00 | 0.00E+00 | 0.00E+00 | 0.00E+00 | 4.38E-07 | 9.06E-07 | 1.20E-06 |
| 82 | 0.00E+00 | 0.00E+00 | 0.00E+00 | 0.00E+00 | 0.00E+00 | 0.00E+00 | 4.13E-07 | 8.75E-07 | 1.17E-06 |
| 83 | 0.00E+00 | 0.00E+00 | 0.00E+00 | 0.00E+00 | 0.00E+00 | 0.00E+00 | 3.88E-07 | 8.44E-07 | 1.14E-06 |
| 84 | 0.00E+00 | 0.00E+00 | 0.00E+00 | 0.00E+00 | 0.00E+00 | 0.00E+00 | 3.62E-07 | 8.14E-07 | 1.12E-06 |
| 85 | 0.00E+00 | 0.00E+00 | 0.00E+00 | 0.00E+00 | 0.00E+00 | 0.00E+00 | 3.40E-07 | 7.88E-07 | 1.09E-06 |
| 86 | 0.00E+00 | 0.00E+00 | 0.00E+00 | 0.00E+00 | 0.00E+00 | 0.00E+00 | 3.16E-07 | 7.66E-07 | 1.06E-06 |
| 87 | 0.00E+00 | 0.00E+00 | 0.00E+00 | 0.00E+00 | 0.00E+00 | 0.00E+00 | 2.92E-07 | 7.45E-07 | 1.04E-06 |
| 88 | 0.00E+00 | 0.00E+00 | 0.00E+00 | 0.00E+00 | 0.00E+00 | 0.00E+00 | 2.67E-07 | 7.25E-07 | 1.01E-06 |
| 89 | 0.00E+00 | 0.00E+00 | 0.00E+00 | 0.00E+00 | 0.00E+00 | 0.00E+00 | 2.42E-07 | 7.05E-07 | 9.89E-07 |
| 90 | 0.00E+00 | 0.00E+00 | 0.00E+00 | 0.00E+00 | 0.00E+00 | 0.00E+00 | 2.18E-07 | 6.85E-07 | 9.66E-07 |
| 91 | 0.00E+00 | 0.00E+00 | 0.00E+00 | 0.00E+00 | 0.00E+00 | 0.00E+00 | 1.95E-07 | 6.64E-07 | 9.43E-07 |
| 92 | 0.00E+00 | 0.00E+00 | 0.00E+00 | 0.00E+00 | 0.00E+00 | 0.00E+00 | 1.74E-07 | 6.43E-07 | 9.19E-07 |
| 93 | 0.00E+00 | 0.00E+00 | 0.00E+00 | 0.00E+00 | 0.00E+00 | 0.00E+00 | 1.55E-07 | 6.23E-07 | 8.94E-07 |
| 94 | 0.00E+00 | 0.00E+00 | 0.00E+00 | 0.00E+00 | 0.00E+00 | 0.00E+00 | 1.36E-07 | 6.01E-07 | 8.66E-07 |
| 95 | 0.00E+00 | 0.00E+00 | 0.00E+00 | 0.00E+00 | 0.00E+00 | 0.00E+00 | 1.16E-07 | 5.78E-07 | 8.38E-07 |
| 96 | 0.00E+00 | 0.00E+00 | 0.00E+00 | 0.00E+00 | 0.00E+00 | 0.00E+00 | 9.51E-08 | 5.53E-07 | 8.09E-07 |
| 97 | 0.00E+00 | 0.00E+00 | 0.00E+00 | 0.00E+00 | 0.00E+00 | 0.00E+00 | 7.36E-08 | 5.30E-07 | 7.84E-07 |
| 98 | 0.00E+00 | 0.00E+00 | 0.00E+00 | 0.00E+00 | 0.00E+00 | 0.00E+00 | 5.22E-08 | 5.06E-07 | 7.60E-07 |
| 99 | 0.00E+00 | 0.00E+00 | 0.00E+00 | 0.00E+00 | 0.00E+00 | 0.00E+00 | 3.06E-08 | 4.85E-07 | 7.39E-07 |
| 100 | 0.00E+00 | 0.00E+00 | 0.00E+00 | 0.00E+00 | 0.00E+00 | 0.00E+00 | 1.21E-08 | 4.68E-07 | 7.22E-07 |
| 101 | 0.00E+00 | 0.00E+00 | 0.00E+00 | 0.00E+00 | 0.00E+00 | 0.00E+00 | 0.00E+00 | 4.53E-07 | 7.05E-07 |
| 102 | 0.00E+00 | 0.00E+00 | 0.00E+00 | 0.00E+00 | 0.00E+00 | 0.00E+00 | 0.00E+00 | 4.39E-07 | 6.87E-07 |
| 103 | 0.00E+00 | 0.00E+00 | 0.00E+00 | 0.00E+00 | 0.00E+00 | 0.00E+00 | 0.00E+00 | 4.21E-07 | 6.70E-07 |
| 104 | 0.00E+00 | 0.00E+00 | 0.00E+00 | 0.00E+00 | 0.00E+00 | 0.00E+00 | 0.00E+00 | 4.04E-07 | 6.51E-07 |
| 105 | 0.00E+00 | 0.00E+00 | 0.00E+00 | 0.00E+00 | 0.00E+00 | 0.00E+00 | 0.00E+00 | 3.84E-07 | 6.31E-07 |
| 106 | 0.00E+00 | 0.00E+00 | 0.00E+00 | 0.00E+00 | 0.00E+00 | 0.00E+00 | 0.00E+00 | 3.63E-07 | 6.11E-07 |
| 107 | 0.00E+00 | 0.00E+00 | 0.00E+00 | 0.00E+00 | 0.00E+00 | 0.00E+00 | 0.00E+00 | 3.43E-07 | 5.94E-07 |
| 108 | 0.00E+00 | 0.00E+00 | 0.00E+00 | 0.00E+00 | 0.00E+00 | 0.00E+00 | 0.00E+00 | 3.30E-07 | 5.77E-07 |
| 109 | 0.00E+00 | 0.00E+00 | 0.00E+00 | 0.00E+00 | 0.00E+00 | 0.00E+00 | 0.00E+00 | 3.14E-07 | 5.62E-07 |
| 110 | 0.00E+00 | 0.00E+00 | 0.00E+00 | 0.00E+00 | 0.00E+00 | 0.00E+00 | 0.00E+00 | 3.02E-07 | 5.49E-07 |
| 111 | 0.00E+00 | 0.00E+00 | 0.00E+00 | 0.00E+00 | 0.00E+00 | 0.00E+00 | 0.00E+00 | 2.88E-07 | 5.35E-07 |
| 112 | 0.00E+00 | 0.00E+00 | 0.00E+00 | 0.00E+00 | 0.00E+00 | 0.00E+00 | 0.00E+00 | 2.73E-07 | 5.20E-07 |
| 113 | 0.00E+00 | 0.00E+00 | 0.00E+00 | 0.00E+00 | 0.00E+00 | 0.00E+00 | 0.00E+00 | 2.53E-07 | 5.02E-07 |
| 114 | 0.00E+00 | 0.00E+00 | 0.00E+00 | 0.00E+00 | 0.00E+00 | 0.00E+00 | 0.00E+00 | 2.33E-07 | 4.83E-07 |
| 115 | 0.00E+00 | 0.00E+00 | 0.00E+00 | 0.00E+00 | 0.00E+00 | 0.00E+00 | 0.00E+00 | 2.11E-07 | 4.63E-07 |
| 116 | 0.00E+00 | 0.00E+00 | 0.00E+00 | 0.00E+00 | 0.00E+00 | 0.00E+00 | 0.00E+00 | 1.91E-07 | 4.42E-07 |
| 117 | 0.00E+00 | 0.00E+00 | 0.00E+00 | 0.00E+00 | 0.00E+00 | 0.00E+00 | 0.00E+00 | 1.74E-07 | 4.21E-07 |
| 118 | 0.00E+00 | 0.00E+00 | 0.00E+00 | 0.00E+00 | 0.00E+00 | 0.00E+00 | 0.00E+00 | 1.60E-07 | 4.03E-07 |
| 119 | 0.00E+00 | 0.00E+00 | 0.00E+00 | 0.00E+00 | 0.00E+00 | 0.00E+00 | 0.00E+00 | 1.47E-07 | 3.87E-07 |
| 120 | 0.00E+00 | 0.00E+00 | 0.00E+00 | 0.00E+00 | 0.00E+00 | 0.00E+00 | 0.00E+00 | 1.36E-07 | 3.72E-07 |
| 121 | 0.00E+00 | 0.00E+00 | 0.00E+00 | 0.00E+00 | 0.00E+00 | 0.00E+00 | 0.00E+00 | 1.24E-07 | 3.58E-07 |
| 122 | 0.00E+00 | 0.00E+00 | 0.00E+00 | 0.00E+00 | 0.00E+00 | 0.00E+00 | 0.00E+00 | 1.12E-07 | 3.45E-07 |
| 123 | 0.00E+00 | 0.00E+00 | 0.00E+00 | 0.00E+00 | 0.00E+00 | 0.00E+00 | 0.00E+00 | 9.77E-08 | 3.33E-07 |
| 124 | 0.00E+00 | 0.00E+00 | 0.00E+00 | 0.00E+00 | 0.00E+00 | 0.00E+00 | 0.00E+00 | 8.35E-08 | 3.20E-07 |
| 125 | 0.00E+00 | 0.00E+00 | 0.00E+00 | 0.00E+00 | 0.00E+00 | 0.00E+00 | 0.00E+00 | 6.97E-08 | 3.07E-07 |
| 126 | 0.00E+00 | 0.00E+00 | 0.00E+00 | 0.00E+00 | 0.00E+00 | 0.00E+00 | 0.00E+00 | 5.58E-08 | 2.94E-07 |
| 127 | 0.00E+00 | 0.00E+00 | 0.00E+00 | 0.00E+00 | 0.00E+00 | 0.00E+00 | 0.00E+00 | 4.31E-08 | 2.79E-07 |
| 128 | 0.00E+00 | 0.00E+00 | 0.00E+00 | 0.00E+00 | 0.00E+00 | 0.00E+00 | 0.00E+00 | 3.06E-08 | 2.64E-07 |
| 129 | 0.00E+00 | 0.00E+00 | 0.00E+00 | 0.00E+00 | 0.00E+00 | 0.00E+00 | 0.00E+00 | 1.95E-08 | 2.49E-07 |
| 130 | 0.00E+00 | 0.00E+00 | 0.00E+00 | 0.00E+00 | 0.00E+00 | 0.00E+00 | 0.00E+00 | 1.08E-08 | 2.33E-07 |
| 131 | 0.00E+00 | 0.00E+00 | 0.00E+00 | 0.00E+00 | 0.00E+00 | 0.00E+00 | 0.00E+00 | 0.00E+00 | 2.18E-07 |
| 132 | 0.00E+00 | 0.00E+00 | 0.00E+00 | 0.00E+00 | 0.00E+00 | 0.00E+00 | 0.00E+00 | 0.00E+00 | 2.04E-07 |
| 133 | 0.00E+00 | 0.00E+00 | 0.00E+00 | 0.00E+00 | 0.00E+00 | 0.00E+00 | 0.00E+00 | 0.00E+00 | 1.91E-07 |
| 134 | 0.00E+00 | 0.00E+00 | 0.00E+00 | 0.00E+00 | 0.00E+00 | 0.00E+00 | 0.00E+00 | 0.00E+00 | 1.79E-07 |
| 135 | 0.00E+00 | 0.00E+00 | 0.00E+00 | 0.00E+00 | 0.00E+00 | 0.00E+00 | 0.00E+00 | 0.00E+00 | 1.67E-07 |
| 136 | 0.00E+00 | 0.00E+00 | 0.00E+00 | 0.00E+00 | 0.00E+00 | 0.00E+00 | 0.00E+00 | 0.00E+00 | 1.56E-07 |
| 137 | 0.00E+00 | 0.00E+00 | 0.00E+00 | 0.00E+00 | 0.00E+00 | 0.00E+00 | 0.00E+00 | 0.00E+00 | 1.44E-07 |
| 138 | 0.00E+00 | 0.00E+00 | 0.00E+00 | 0.00E+00 | 0.00E+00 | 0.00E+00 | 0.00E+00 | 0.00E+00 | 1.32E-07 |
| 139 | 0.00E+00 | 0.00E+00 | 0.00E+00 | 0.00E+00 | 0.00E+00 | 0.00E+00 | 0.00E+00 | 0.00E+00 | 1.20E-07 |
| 140 | 0.00E+00 | 0.00E+00 | 0.00E+00 | 0.00E+00 | 0.00E+00 | 0.00E+00 | 0.00E+00 | 0.00E+00 | 1.09E-07 |
| 141 | 0.00E+00 | 0.00E+00 | 0.00E+00 | 0.00E+00 | 0.00E+00 | 0.00E+00 | 0.00E+00 | 0.00E+00 | 9.76E-08 |
| 142 | 0.00E+00 | 0.00E+00 | 0.00E+00 | 0.00E+00 | 0.00E+00 | 0.00E+00 | 0.00E+00 | 0.00E+00 | 8.71E-08 |
| 143 | 0.00E+00 | 0.00E+00 | 0.00E+00 | 0.00E+00 | 0.00E+00 | 0.00E+00 | 0.00E+00 | 0.00E+00 | 7.72E-08 |
| 144 | 0.00E+00 | 0.00E+00 | 0.00E+00 | 0.00E+00 | 0.00E+00 | 0.00E+00 | 0.00E+00 | 0.00E+00 | 6.75E-08 |
| 145 | 0.00E+00 | 0.00E+00 | 0.00E+00 | 0.00E+00 | 0.00E+00 | 0.00E+00 | 0.00E+00 | 0.00E+00 | 5.78E-08 |
| 146 | 0.00E+00 | 0.00E+00 | 0.00E+00 | 0.00E+00 | 0.00E+00 | 0.00E+00 | 0.00E+00 | 0.00E+00 | 4.80E-08 |
| 147 | 0.00E+00 | 0.00E+00 | 0.00E+00 | 0.00E+00 | 0.00E+00 | 0.00E+00 | 0.00E+00 | 0.00E+00 | 3.77E-08 |
| 148 | 0.00E+00 | 0.00E+00 | 0.00E+00 | 0.00E+00 | 0.00E+00 | 0.00E+00 | 0.00E+00 | 0.00E+00 | 2.68E-08 |
| 149 | 0.00E+00 | 0.00E+00 | 0.00E+00 | 0.00E+00 | 0.00E+00 | 0.00E+00 | 0.00E+00 | 0.00E+00 | 1.58E-08 |
| 150 | 0.00E+00 | 0.00E+00 | 0.00E+00 | 0.00E+00 | 0.00E+00 | 0.00E+00 | 0.00E+00 | 0.00E+00 | 3.73E-09 |

Table.5. Spectra obtained by MCNP code: Be- 1.6mm

| energy bin (kev)/voltage(kv) | 20kv | 30kv | 40kv | 50kv | 60kv | 80kv | 100kv | 130kv | 150kv |
| --- | --- | --- | --- | --- | --- | --- | --- | --- | --- |
| 1 | 0.00E+00 | 0.00E+00 | 0.00E+00 | 0.00E+00 | 0.00E+00 | 0.00E+00 | 0.00E+00 | 0.00E+00 | 0.00E+00 |
| 2 | 6.04E-22 | 4.69E-22 | 5.94E-22 | 1.62E-22 | 3.59E-22 | 6.19E-22 | 9.41E-22 | 2.14E-21 | 1.19E-21 |
| 3 | 4.40E-10 | 6.16E-11 | 5.30E-11 | 5.21E-11 | 4.93E-11 | 4.75E-11 | 4.93E-11 | 6.54E-11 | 7.33E-11 |
| 4 | 8.48E-08 | 5.35E-08 | 4.98E-08 | 4.48E-08 | 4.12E-08 | 3.83E-08 | 3.67E-08 | 4.33E-08 | 3.79E-08 |
| 5 | 4.69E-07 | 6.31E-07 | 6.17E-07 | 5.98E-07 | 5.51E-07 | 4.94E-07 | 4.66E-07 | 5.13E-07 | 4.31E-07 |
| 6 | 1.08E-06 | 1.71E-06 | 1.86E-06 | 1.87E-06 | 1.83E-06 | 1.67E-06 | 1.53E-06 | 1.55E-06 | 1.34E-06 |
| 7 | 1.62E-06 | 2.52E-06 | 2.89E-06 | 3.08E-06 | 3.13E-06 | 2.96E-06 | 2.83E-06 | 2.94E-06 | 2.41E-06 |
| 8 | 2.05E-06 | 3.19E-06 | 3.86E-06 | 4.35E-06 | 4.57E-06 | 4.69E-06 | 4.52E-06 | 4.74E-06 | 4.09E-06 |
| 9 | 2.06E-06 | 4.22E-06 | 5.81E-06 | 7.17E-06 | 8.16E-06 | 9.54E-06 | 1.03E-05 | 1.20E-05 | 1.06E-05 |
| 10 | 1.98E-06 | 3.32E-06 | 4.35E-06 | 5.30E-06 | 5.99E-06 | 6.81E-06 | 7.31E-06 | 8.26E-06 | 7.30E-06 |
| 11 | 1.75E-06 | 2.65E-06 | 3.42E-06 | 3.92E-06 | 4.25E-06 | 4.51E-06 | 4.51E-06 | 4.95E-06 | 4.22E-06 |
| 12 | 1.45E-06 | 2.41E-06 | 3.03E-06 | 3.49E-06 | 3.70E-06 | 3.83E-06 | 3.85E-06 | 4.07E-06 | 3.37E-06 |
| 13 | 1.23E-06 | 2.11E-06 | 2.68E-06 | 3.04E-06 | 3.27E-06 | 3.25E-06 | 3.25E-06 | 3.32E-06 | 2.82E-06 |
| 14 | 1.05E-06 | 1.98E-06 | 2.60E-06 | 3.01E-06 | 3.35E-06 | 3.54E-06 | 3.62E-06 | 3.73E-06 | 3.18E-06 |
| 15 | 8.86E-07 | 1.86E-06 | 2.52E-06 | 3.01E-06 | 3.36E-06 | 3.67E-06 | 3.80E-06 | 4.08E-06 | 3.52E-06 |
| 16 | 7.27E-07 | 1.72E-06 | 2.40E-06 | 2.94E-06 | 3.32E-06 | 3.74E-06 | 3.95E-06 | 4.33E-06 | 3.81E-06 |
| 17 | 5.68E-07 | 1.58E-06 | 2.29E-06 | 2.88E-06 | 3.27E-06 | 3.80E-06 | 4.09E-06 | 4.58E-06 | 4.10E-06 |
| 18 | 4.10E-07 | 1.44E-06 | 2.16E-06 | 2.78E-06 | 3.20E-06 | 3.82E-06 | 4.19E-06 | 4.77E-06 | 4.34E-06 |
| 19 | 2.54E-07 | 1.31E-06 | 2.03E-06 | 2.66E-06 | 3.12E-06 | 3.82E-06 | 4.27E-06 | 4.93E-06 | 4.55E-06 |
| 20 | 9.64E-08 | 1.17E-06 | 1.90E-06 | 2.54E-06 | 3.02E-06 | 3.78E-06 | 4.30E-06 | 5.04E-06 | 4.72E-06 |
| 21 | 0.00E+00 | 1.05E-06 | 1.77E-06 | 2.41E-06 | 2.91E-06 | 3.71E-06 | 4.30E-06 | 5.11E-06 | 4.86E-06 |
| 22 | 0.00E+00 | 9.23E-07 | 1.64E-06 | 2.28E-06 | 2.80E-06 | 3.63E-06 | 4.28E-06 | 5.14E-06 | 4.96E-06 |
| 23 | 0.00E+00 | 8.06E-07 | 1.51E-06 | 2.15E-06 | 2.69E-06 | 3.53E-06 | 4.24E-06 | 5.13E-06 | 5.04E-06 |
| 24 | 0.00E+00 | 6.94E-07 | 1.39E-06 | 2.02E-06 | 2.57E-06 | 3.43E-06 | 4.17E-06 | 5.10E-06 | 5.08E-06 |
| 25 | 0.00E+00 | 5.87E-07 | 1.28E-06 | 1.90E-06 | 2.45E-06 | 3.33E-06 | 4.09E-06 | 5.06E-06 | 5.10E-06 |
| 26 | 0.00E+00 | 4.82E-07 | 1.18E-06 | 1.78E-06 | 2.33E-06 | 3.22E-06 | 4.00E-06 | 5.00E-06 | 5.10E-06 |
| 27 | 0.00E+00 | 3.79E-07 | 1.08E-06 | 1.67E-06 | 2.22E-06 | 3.12E-06 | 3.90E-06 | 4.92E-06 | 5.08E-06 |
| 28 | 0.00E+00 | 2.74E-07 | 9.82E-07 | 1.57E-06 | 2.10E-06 | 3.01E-06 | 3.79E-06 | 4.84E-06 | 5.05E-06 |
| 29 | 0.00E+00 | 1.70E-07 | 8.91E-07 | 1.46E-06 | 1.98E-06 | 2.90E-06 | 3.69E-06 | 4.74E-06 | 5.00E-06 |
| 30 | 0.00E+00 | 6.14E-08 | 8.02E-07 | 1.37E-06 | 1.88E-06 | 2.79E-06 | 3.59E-06 | 4.61E-06 | 4.94E-06 |
| 31 | 0.00E+00 | 0.00E+00 | 7.18E-07 | 1.27E-06 | 1.77E-06 | 2.68E-06 | 3.49E-06 | 4.48E-06 | 4.88E-06 |
| 32 | 0.00E+00 | 0.00E+00 | 6.34E-07 | 1.19E-06 | 1.67E-06 | 2.57E-06 | 3.38E-06 | 4.35E-06 | 4.80E-06 |
| 33 | 0.00E+00 | 0.00E+00 | 5.54E-07 | 1.11E-06 | 1.58E-06 | 2.46E-06 | 3.27E-06 | 4.22E-06 | 4.73E-06 |
| 34 | 0.00E+00 | 0.00E+00 | 4.77E-07 | 1.03E-06 | 1.50E-06 | 2.34E-06 | 3.16E-06 | 4.10E-06 | 4.64E-06 |
| 35 | 0.00E+00 | 0.00E+00 | 4.01E-07 | 9.51E-07 | 1.42E-06 | 2.23E-06 | 3.05E-06 | 3.98E-06 | 4.54E-06 |
| 36 | 0.00E+00 | 0.00E+00 | 3.27E-07 | 8.77E-07 | 1.34E-06 | 2.12E-06 | 2.93E-06 | 3.87E-06 | 4.44E-06 |
| 37 | 0.00E+00 | 0.00E+00 | 2.54E-07 | 8.03E-07 | 1.26E-06 | 2.02E-06 | 2.82E-06 | 3.77E-06 | 4.32E-06 |
| 38 | 0.00E+00 | 0.00E+00 | 1.83E-07 | 7.34E-07 | 1.18E-06 | 1.93E-06 | 2.72E-06 | 3.67E-06 | 4.21E-06 |
| 39 | 0.00E+00 | 0.00E+00 | 1.13E-07 | 6.65E-07 | 1.10E-06 | 1.85E-06 | 2.61E-06 | 3.57E-06 | 4.10E-06 |
| 40 | 0.00E+00 | 0.00E+00 | 4.92E-08 | 5.98E-07 | 1.03E-06 | 1.77E-06 | 2.51E-06 | 3.47E-06 | 3.99E-06 |
| 41 | 0.00E+00 | 0.00E+00 | 0.00E+00 | 5.35E-07 | 9.63E-07 | 1.71E-06 | 2.41E-06 | 3.37E-06 | 3.89E-06 |
| 42 | 0.00E+00 | 0.00E+00 | 0.00E+00 | 4.74E-07 | 9.02E-07 | 1.64E-06 | 2.33E-06 | 3.26E-06 | 3.79E-06 |
| 43 | 0.00E+00 | 0.00E+00 | 0.00E+00 | 4.13E-07 | 8.44E-07 | 1.57E-06 | 2.24E-06 | 3.16E-06 | 3.69E-06 |
| 44 | 0.00E+00 | 0.00E+00 | 0.00E+00 | 3.58E-07 | 7.85E-07 | 1.50E-06 | 2.16E-06 | 3.06E-06 | 3.59E-06 |
| 45 | 0.00E+00 | 0.00E+00 | 0.00E+00 | 3.03E-07 | 7.29E-07 | 1.44E-06 | 2.09E-06 | 2.97E-06 | 3.49E-06 |
| 46 | 0.00E+00 | 0.00E+00 | 0.00E+00 | 2.49E-07 | 6.73E-07 | 1.37E-06 | 2.02E-06 | 2.89E-06 | 3.40E-06 |
| 47 | 0.00E+00 | 0.00E+00 | 0.00E+00 | 1.96E-07 | 6.20E-07 | 1.31E-06 | 1.95E-06 | 2.80E-06 | 3.32E-06 |
| 48 | 0.00E+00 | 0.00E+00 | 0.00E+00 | 1.44E-07 | 5.69E-07 | 1.25E-06 | 1.88E-06 | 2.71E-06 | 3.23E-06 |
| 49 | 0.00E+00 | 0.00E+00 | 0.00E+00 | 9.14E-08 | 5.20E-07 | 1.19E-06 | 1.80E-06 | 2.62E-06 | 3.15E-06 |
| 50 | 0.00E+00 | 0.00E+00 | 0.00E+00 | 3.78E-08 | 4.73E-07 | 1.13E-06 | 1.74E-06 | 2.54E-06 | 3.06E-06 |
| 51 | 0.00E+00 | 0.00E+00 | 0.00E+00 | 0.00E+00 | 4.26E-07 | 1.08E-06 | 1.67E-06 | 2.46E-06 | 2.98E-06 |
| 52 | 0.00E+00 | 0.00E+00 | 0.00E+00 | 0.00E+00 | 3.79E-07 | 1.02E-06 | 1.61E-06 | 2.39E-06 | 2.90E-06 |
| 53 | 0.00E+00 | 0.00E+00 | 0.00E+00 | 0.00E+00 | 3.32E-07 | 9.62E-07 | 1.56E-06 | 2.34E-06 | 2.83E-06 |
| 54 | 0.00E+00 | 0.00E+00 | 0.00E+00 | 0.00E+00 | 2.86E-07 | 9.10E-07 | 1.52E-06 | 2.28E-06 | 2.76E-06 |
| 55 | 0.00E+00 | 0.00E+00 | 0.00E+00 | 0.00E+00 | 2.41E-07 | 8.61E-07 | 1.46E-06 | 2.22E-06 | 2.70E-06 |
| 56 | 0.00E+00 | 0.00E+00 | 0.00E+00 | 0.00E+00 | 1.97E-07 | 8.18E-07 | 1.41E-06 | 2.15E-06 | 2.64E-06 |
| 57 | 0.00E+00 | 0.00E+00 | 0.00E+00 | 0.00E+00 | 1.54E-07 | 7.76E-07 | 1.35E-06 | 2.08E-06 | 2.58E-06 |
| 58 | 0.00E+00 | 0.00E+00 | 0.00E+00 | 0.00E+00 | 1.10E-07 | 7.39E-07 | 1.26E-06 | 1.99E-06 | 2.51E-06 |
| 59 | 0.00E+00 | 0.00E+00 | 0.00E+00 | 0.00E+00 | 6.94E-08 | 8.48E-07 | 2.24E-06 | 4.83E-06 | 6.53E-06 |
| 60 | 0.00E+00 | 0.00E+00 | 0.00E+00 | 0.00E+00 | 2.68E-08 | 9.41E-07 | 2.96E-06 | 7.10E-06 | 9.86E-06 |
| 61 | 0.00E+00 | 0.00E+00 | 0.00E+00 | 0.00E+00 | 0.00E+00 | 6.09E-07 | 1.09E-06 | 1.86E-06 | 2.37E-06 |
| 62 | 0.00E+00 | 0.00E+00 | 0.00E+00 | 0.00E+00 | 0.00E+00 | 5.93E-07 | 1.19E-06 | 1.93E-06 | 2.56E-06 |
| 63 | 0.00E+00 | 0.00E+00 | 0.00E+00 | 0.00E+00 | 0.00E+00 | 5.92E-07 | 1.03E-06 | 1.62E-06 | 2.09E-06 |
| 64 | 0.00E+00 | 0.00E+00 | 0.00E+00 | 0.00E+00 | 0.00E+00 | 5.12E-07 | 1.01E-06 | 1.54E-06 | 2.03E-06 |
| 65 | 0.00E+00 | 0.00E+00 | 0.00E+00 | 0.00E+00 | 0.00E+00 | 4.77E-07 | 9.71E-07 | 1.54E-06 | 2.01E-06 |
| 66 | 0.00E+00 | 0.00E+00 | 0.00E+00 | 0.00E+00 | 0.00E+00 | 4.48E-07 | 9.13E-07 | 1.52E-06 | 1.98E-06 |
| 67 | 0.00E+00 | 0.00E+00 | 0.00E+00 | 0.00E+00 | 0.00E+00 | 4.26E-07 | 8.42E-07 | 1.47E-06 | 1.93E-06 |
| 68 | 0.00E+00 | 0.00E+00 | 0.00E+00 | 0.00E+00 | 0.00E+00 | 4.83E-07 | 1.50E-06 | 3.23E-06 | 4.58E-06 |
| 69 | 0.00E+00 | 0.00E+00 | 0.00E+00 | 0.00E+00 | 0.00E+00 | 3.37E-07 | 7.99E-07 | 1.43E-06 | 1.75E-06 |
| 70 | 0.00E+00 | 0.00E+00 | 0.00E+00 | 0.00E+00 | 0.00E+00 | 3.27E-07 | 9.38E-07 | 1.83E-06 | 2.44E-06 |
| 71 | 0.00E+00 | 0.00E+00 | 0.00E+00 | 0.00E+00 | 0.00E+00 | 2.76E-07 | 7.41E-07 | 1.24E-06 | 1.46E-06 |
| 72 | 0.00E+00 | 0.00E+00 | 0.00E+00 | 0.00E+00 | 0.00E+00 | 2.47E-07 | 6.58E-07 | 1.21E-06 | 1.47E-06 |
| 73 | 0.00E+00 | 0.00E+00 | 0.00E+00 | 0.00E+00 | 0.00E+00 | 2.14E-07 | 6.26E-07 | 1.16E-06 | 1.43E-06 |
| 74 | 0.00E+00 | 0.00E+00 | 0.00E+00 | 0.00E+00 | 0.00E+00 | 1.84E-07 | 5.97E-07 | 1.13E-06 | 1.39E-06 |
| 75 | 0.00E+00 | 0.00E+00 | 0.00E+00 | 0.00E+00 | 0.00E+00 | 1.55E-07 | 5.70E-07 | 1.09E-06 | 1.36E-06 |
| 76 | 0.00E+00 | 0.00E+00 | 0.00E+00 | 0.00E+00 | 0.00E+00 | 1.26E-07 | 5.45E-07 | 1.07E-06 | 1.32E-06 |
| 77 | 0.00E+00 | 0.00E+00 | 0.00E+00 | 0.00E+00 | 0.00E+00 | 9.84E-08 | 5.24E-07 | 1.03E-06 | 1.29E-06 |
| 78 | 0.00E+00 | 0.00E+00 | 0.00E+00 | 0.00E+00 | 0.00E+00 | 7.09E-08 | 5.03E-07 | 1.00E-06 | 1.26E-06 |
| 79 | 0.00E+00 | 0.00E+00 | 0.00E+00 | 0.00E+00 | 0.00E+00 | 4.30E-08 | 4.82E-07 | 9.69E-07 | 1.23E-06 |
| 80 | 0.00E+00 | 0.00E+00 | 0.00E+00 | 0.00E+00 | 0.00E+00 | 1.49E-08 | 4.58E-07 | 9.32E-07 | 1.21E-06 |
| 81 | 0.00E+00 | 0.00E+00 | 0.00E+00 | 0.00E+00 | 0.00E+00 | 0.00E+00 | 4.34E-07 | 8.97E-07 | 1.18E-06 |
| 82 | 0.00E+00 | 0.00E+00 | 0.00E+00 | 0.00E+00 | 0.00E+00 | 0.00E+00 | 4.09E-07 | 8.66E-07 | 1.16E-06 |
| 83 | 0.00E+00 | 0.00E+00 | 0.00E+00 | 0.00E+00 | 0.00E+00 | 0.00E+00 | 3.84E-07 | 8.35E-07 | 1.13E-06 |
| 84 | 0.00E+00 | 0.00E+00 | 0.00E+00 | 0.00E+00 | 0.00E+00 | 0.00E+00 | 3.59E-07 | 8.05E-07 | 1.11E-06 |
| 85 | 0.00E+00 | 0.00E+00 | 0.00E+00 | 0.00E+00 | 0.00E+00 | 0.00E+00 | 3.37E-07 | 7.80E-07 | 1.08E-06 |
| 86 | 0.00E+00 | 0.00E+00 | 0.00E+00 | 0.00E+00 | 0.00E+00 | 0.00E+00 | 3.13E-07 | 7.58E-07 | 1.05E-06 |
| 87 | 0.00E+00 | 0.00E+00 | 0.00E+00 | 0.00E+00 | 0.00E+00 | 0.00E+00 | 2.89E-07 | 7.38E-07 | 1.03E-06 |
| 88 | 0.00E+00 | 0.00E+00 | 0.00E+00 | 0.00E+00 | 0.00E+00 | 0.00E+00 | 2.64E-07 | 7.18E-07 | 1.00E-06 |
| 89 | 0.00E+00 | 0.00E+00 | 0.00E+00 | 0.00E+00 | 0.00E+00 | 0.00E+00 | 2.40E-07 | 6.98E-07 | 9.79E-07 |
| 90 | 0.00E+00 | 0.00E+00 | 0.00E+00 | 0.00E+00 | 0.00E+00 | 0.00E+00 | 2.16E-07 | 6.78E-07 | 9.56E-07 |
| 91 | 0.00E+00 | 0.00E+00 | 0.00E+00 | 0.00E+00 | 0.00E+00 | 0.00E+00 | 1.93E-07 | 6.58E-07 | 9.34E-07 |
| 92 | 0.00E+00 | 0.00E+00 | 0.00E+00 | 0.00E+00 | 0.00E+00 | 0.00E+00 | 1.73E-07 | 6.37E-07 | 9.09E-07 |
| 93 | 0.00E+00 | 0.00E+00 | 0.00E+00 | 0.00E+00 | 0.00E+00 | 0.00E+00 | 1.54E-07 | 6.17E-07 | 8.85E-07 |
| 94 | 0.00E+00 | 0.00E+00 | 0.00E+00 | 0.00E+00 | 0.00E+00 | 0.00E+00 | 1.35E-07 | 5.95E-07 | 8.58E-07 |
| 95 | 0.00E+00 | 0.00E+00 | 0.00E+00 | 0.00E+00 | 0.00E+00 | 0.00E+00 | 1.15E-07 | 5.72E-07 | 8.29E-07 |
| 96 | 0.00E+00 | 0.00E+00 | 0.00E+00 | 0.00E+00 | 0.00E+00 | 0.00E+00 | 9.42E-08 | 5.48E-07 | 8.01E-07 |
| 97 | 0.00E+00 | 0.00E+00 | 0.00E+00 | 0.00E+00 | 0.00E+00 | 0.00E+00 | 7.29E-08 | 5.24E-07 | 7.76E-07 |
| 98 | 0.00E+00 | 0.00E+00 | 0.00E+00 | 0.00E+00 | 0.00E+00 | 0.00E+00 | 5.17E-08 | 5.01E-07 | 7.52E-07 |
| 99 | 0.00E+00 | 0.00E+00 | 0.00E+00 | 0.00E+00 | 0.00E+00 | 0.00E+00 | 3.03E-08 | 4.80E-07 | 7.32E-07 |
| 100 | 0.00E+00 | 0.00E+00 | 0.00E+00 | 0.00E+00 | 0.00E+00 | 0.00E+00 | 1.20E-08 | 4.63E-07 | 7.15E-07 |
| 101 | 0.00E+00 | 0.00E+00 | 0.00E+00 | 0.00E+00 | 0.00E+00 | 0.00E+00 | 0.00E+00 | 4.49E-07 | 6.98E-07 |
| 102 | 0.00E+00 | 0.00E+00 | 0.00E+00 | 0.00E+00 | 0.00E+00 | 0.00E+00 | 0.00E+00 | 4.35E-07 | 6.81E-07 |
| 103 | 0.00E+00 | 0.00E+00 | 0.00E+00 | 0.00E+00 | 0.00E+00 | 0.00E+00 | 0.00E+00 | 4.17E-07 | 6.64E-07 |
| 104 | 0.00E+00 | 0.00E+00 | 0.00E+00 | 0.00E+00 | 0.00E+00 | 0.00E+00 | 0.00E+00 | 4.00E-07 | 6.45E-07 |
| 105 | 0.00E+00 | 0.00E+00 | 0.00E+00 | 0.00E+00 | 0.00E+00 | 0.00E+00 | 0.00E+00 | 3.81E-07 | 6.24E-07 |
| 106 | 0.00E+00 | 0.00E+00 | 0.00E+00 | 0.00E+00 | 0.00E+00 | 0.00E+00 | 0.00E+00 | 3.60E-07 | 6.05E-07 |
| 107 | 0.00E+00 | 0.00E+00 | 0.00E+00 | 0.00E+00 | 0.00E+00 | 0.00E+00 | 0.00E+00 | 3.40E-07 | 5.88E-07 |
| 108 | 0.00E+00 | 0.00E+00 | 0.00E+00 | 0.00E+00 | 0.00E+00 | 0.00E+00 | 0.00E+00 | 3.26E-07 | 5.72E-07 |
| 109 | 0.00E+00 | 0.00E+00 | 0.00E+00 | 0.00E+00 | 0.00E+00 | 0.00E+00 | 0.00E+00 | 3.11E-07 | 5.57E-07 |
| 110 | 0.00E+00 | 0.00E+00 | 0.00E+00 | 0.00E+00 | 0.00E+00 | 0.00E+00 | 0.00E+00 | 2.99E-07 | 5.44E-07 |
| 111 | 0.00E+00 | 0.00E+00 | 0.00E+00 | 0.00E+00 | 0.00E+00 | 0.00E+00 | 0.00E+00 | 2.86E-07 | 5.29E-07 |
| 112 | 0.00E+00 | 0.00E+00 | 0.00E+00 | 0.00E+00 | 0.00E+00 | 0.00E+00 | 0.00E+00 | 2.71E-07 | 5.15E-07 |
| 113 | 0.00E+00 | 0.00E+00 | 0.00E+00 | 0.00E+00 | 0.00E+00 | 0.00E+00 | 0.00E+00 | 2.50E-07 | 4.98E-07 |
| 114 | 0.00E+00 | 0.00E+00 | 0.00E+00 | 0.00E+00 | 0.00E+00 | 0.00E+00 | 0.00E+00 | 2.30E-07 | 4.79E-07 |
| 115 | 0.00E+00 | 0.00E+00 | 0.00E+00 | 0.00E+00 | 0.00E+00 | 0.00E+00 | 0.00E+00 | 2.09E-07 | 4.58E-07 |
| 116 | 0.00E+00 | 0.00E+00 | 0.00E+00 | 0.00E+00 | 0.00E+00 | 0.00E+00 | 0.00E+00 | 1.90E-07 | 4.38E-07 |
| 117 | 0.00E+00 | 0.00E+00 | 0.00E+00 | 0.00E+00 | 0.00E+00 | 0.00E+00 | 0.00E+00 | 1.72E-07 | 4.17E-07 |
| 118 | 0.00E+00 | 0.00E+00 | 0.00E+00 | 0.00E+00 | 0.00E+00 | 0.00E+00 | 0.00E+00 | 1.58E-07 | 3.99E-07 |
| 119 | 0.00E+00 | 0.00E+00 | 0.00E+00 | 0.00E+00 | 0.00E+00 | 0.00E+00 | 0.00E+00 | 1.46E-07 | 3.83E-07 |
| 120 | 0.00E+00 | 0.00E+00 | 0.00E+00 | 0.00E+00 | 0.00E+00 | 0.00E+00 | 0.00E+00 | 1.35E-07 | 3.68E-07 |
| 121 | 0.00E+00 | 0.00E+00 | 0.00E+00 | 0.00E+00 | 0.00E+00 | 0.00E+00 | 0.00E+00 | 1.23E-07 | 3.55E-07 |
| 122 | 0.00E+00 | 0.00E+00 | 0.00E+00 | 0.00E+00 | 0.00E+00 | 0.00E+00 | 0.00E+00 | 1.11E-07 | 3.42E-07 |
| 123 | 0.00E+00 | 0.00E+00 | 0.00E+00 | 0.00E+00 | 0.00E+00 | 0.00E+00 | 0.00E+00 | 9.68E-08 | 3.29E-07 |
| 124 | 0.00E+00 | 0.00E+00 | 0.00E+00 | 0.00E+00 | 0.00E+00 | 0.00E+00 | 0.00E+00 | 8.27E-08 | 3.17E-07 |
| 125 | 0.00E+00 | 0.00E+00 | 0.00E+00 | 0.00E+00 | 0.00E+00 | 0.00E+00 | 0.00E+00 | 6.90E-08 | 3.04E-07 |
| 126 | 0.00E+00 | 0.00E+00 | 0.00E+00 | 0.00E+00 | 0.00E+00 | 0.00E+00 | 0.00E+00 | 5.52E-08 | 2.91E-07 |
| 127 | 0.00E+00 | 0.00E+00 | 0.00E+00 | 0.00E+00 | 0.00E+00 | 0.00E+00 | 0.00E+00 | 4.27E-08 | 2.77E-07 |
| 128 | 0.00E+00 | 0.00E+00 | 0.00E+00 | 0.00E+00 | 0.00E+00 | 0.00E+00 | 0.00E+00 | 3.03E-08 | 2.62E-07 |
| 129 | 0.00E+00 | 0.00E+00 | 0.00E+00 | 0.00E+00 | 0.00E+00 | 0.00E+00 | 0.00E+00 | 1.93E-08 | 2.46E-07 |
| 130 | 0.00E+00 | 0.00E+00 | 0.00E+00 | 0.00E+00 | 0.00E+00 | 0.00E+00 | 0.00E+00 | 1.07E-08 | 2.31E-07 |
| 131 | 0.00E+00 | 0.00E+00 | 0.00E+00 | 0.00E+00 | 0.00E+00 | 0.00E+00 | 0.00E+00 | 0.00E+00 | 2.16E-07 |
| 132 | 0.00E+00 | 0.00E+00 | 0.00E+00 | 0.00E+00 | 0.00E+00 | 0.00E+00 | 0.00E+00 | 0.00E+00 | 2.02E-07 |
| 133 | 0.00E+00 | 0.00E+00 | 0.00E+00 | 0.00E+00 | 0.00E+00 | 0.00E+00 | 0.00E+00 | 0.00E+00 | 1.90E-07 |
| 134 | 0.00E+00 | 0.00E+00 | 0.00E+00 | 0.00E+00 | 0.00E+00 | 0.00E+00 | 0.00E+00 | 0.00E+00 | 1.77E-07 |
| 135 | 0.00E+00 | 0.00E+00 | 0.00E+00 | 0.00E+00 | 0.00E+00 | 0.00E+00 | 0.00E+00 | 0.00E+00 | 1.66E-07 |
| 136 | 0.00E+00 | 0.00E+00 | 0.00E+00 | 0.00E+00 | 0.00E+00 | 0.00E+00 | 0.00E+00 | 0.00E+00 | 1.54E-07 |
| 137 | 0.00E+00 | 0.00E+00 | 0.00E+00 | 0.00E+00 | 0.00E+00 | 0.00E+00 | 0.00E+00 | 0.00E+00 | 1.43E-07 |
| 138 | 0.00E+00 | 0.00E+00 | 0.00E+00 | 0.00E+00 | 0.00E+00 | 0.00E+00 | 0.00E+00 | 0.00E+00 | 1.31E-07 |
| 139 | 0.00E+00 | 0.00E+00 | 0.00E+00 | 0.00E+00 | 0.00E+00 | 0.00E+00 | 0.00E+00 | 0.00E+00 | 1.19E-07 |
| 140 | 0.00E+00 | 0.00E+00 | 0.00E+00 | 0.00E+00 | 0.00E+00 | 0.00E+00 | 0.00E+00 | 0.00E+00 | 1.08E-07 |
| 141 | 0.00E+00 | 0.00E+00 | 0.00E+00 | 0.00E+00 | 0.00E+00 | 0.00E+00 | 0.00E+00 | 0.00E+00 | 9.67E-08 |
| 142 | 0.00E+00 | 0.00E+00 | 0.00E+00 | 0.00E+00 | 0.00E+00 | 0.00E+00 | 0.00E+00 | 0.00E+00 | 8.64E-08 |
| 143 | 0.00E+00 | 0.00E+00 | 0.00E+00 | 0.00E+00 | 0.00E+00 | 0.00E+00 | 0.00E+00 | 0.00E+00 | 7.65E-08 |
| 144 | 0.00E+00 | 0.00E+00 | 0.00E+00 | 0.00E+00 | 0.00E+00 | 0.00E+00 | 0.00E+00 | 0.00E+00 | 6.69E-08 |
| 145 | 0.00E+00 | 0.00E+00 | 0.00E+00 | 0.00E+00 | 0.00E+00 | 0.00E+00 | 0.00E+00 | 0.00E+00 | 5.73E-08 |
| 146 | 0.00E+00 | 0.00E+00 | 0.00E+00 | 0.00E+00 | 0.00E+00 | 0.00E+00 | 0.00E+00 | 0.00E+00 | 4.75E-08 |
| 147 | 0.00E+00 | 0.00E+00 | 0.00E+00 | 0.00E+00 | 0.00E+00 | 0.00E+00 | 0.00E+00 | 0.00E+00 | 3.74E-08 |
| 148 | 0.00E+00 | 0.00E+00 | 0.00E+00 | 0.00E+00 | 0.00E+00 | 0.00E+00 | 0.00E+00 | 0.00E+00 | 2.66E-08 |
| 149 | 0.00E+00 | 0.00E+00 | 0.00E+00 | 0.00E+00 | 0.00E+00 | 0.00E+00 | 0.00E+00 | 0.00E+00 | 1.56E-08 |
| 150 | 0.00E+00 | 0.00E+00 | 0.00E+00 | 0.00E+00 | 0.00E+00 | 0.00E+00 | 0.00E+00 | 0.00E+00 | 3.70E-09 |

Table.6. Spectra obtained by MCNP code: Be- 2mm

| energy bin (kev)/voltage(kv) | 20kv | 30kv | 40kv | 50kv | 60kv | 80kv | 100kv | 130kv | 150kv |
| --- | --- | --- | --- | --- | --- | --- | --- | --- | --- |
| 1 | 0.00E+00 | 0.00E+00 | 0.00E+00 | 0.00E+00 | 0.00E+00 | 0.00E+00 | 0.00E+00 | 0.00E+00 | 0.00E+00 |
| 2 | 2.40E-24 | 1.87E-24 | 2.36E-24 | 6.46E-25 | 1.43E-24 | 2.46E-24 | 3.74E-24 | 8.52E-24 | 4.72E-24 |
| 3 | 9.11E-11 | 1.27E-11 | 1.10E-11 | 1.08E-11 | 1.02E-11 | 9.82E-12 | 1.02E-11 | 1.35E-11 | 1.52E-11 |
| 4 | 4.46E-08 | 2.81E-08 | 2.62E-08 | 2.36E-08 | 2.17E-08 | 2.01E-08 | 1.93E-08 | 2.28E-08 | 1.99E-08 |
| 5 | 3.39E-07 | 4.57E-07 | 4.47E-07 | 4.33E-07 | 3.99E-07 | 3.57E-07 | 3.37E-07 | 3.71E-07 | 3.12E-07 |
| 6 | 8.98E-07 | 1.42E-06 | 1.55E-06 | 1.55E-06 | 1.51E-06 | 1.39E-06 | 1.27E-06 | 1.29E-06 | 1.11E-06 |
| 7 | 1.41E-06 | 2.20E-06 | 2.52E-06 | 2.69E-06 | 2.73E-06 | 2.59E-06 | 2.47E-06 | 2.57E-06 | 2.11E-06 |
| 8 | 1.89E-06 | 2.94E-06 | 3.55E-06 | 4.00E-06 | 4.21E-06 | 4.32E-06 | 4.16E-06 | 4.37E-06 | 3.77E-06 |
| 9 | 1.93E-06 | 3.96E-06 | 5.44E-06 | 6.72E-06 | 7.64E-06 | 8.94E-06 | 9.66E-06 | 1.12E-05 | 9.95E-06 |
| 10 | 1.89E-06 | 3.16E-06 | 4.15E-06 | 5.05E-06 | 5.71E-06 | 6.49E-06 | 6.97E-06 | 7.87E-06 | 6.96E-06 |
| 11 | 1.68E-06 | 2.54E-06 | 3.28E-06 | 3.75E-06 | 4.08E-06 | 4.32E-06 | 4.32E-06 | 4.75E-06 | 4.04E-06 |
| 12 | 1.40E-06 | 2.32E-06 | 2.92E-06 | 3.36E-06 | 3.57E-06 | 3.69E-06 | 3.71E-06 | 3.92E-06 | 3.24E-06 |
| 13 | 1.19E-06 | 2.05E-06 | 2.60E-06 | 2.95E-06 | 3.17E-06 | 3.14E-06 | 3.14E-06 | 3.21E-06 | 2.73E-06 |
| 14 | 1.02E-06 | 1.92E-06 | 2.52E-06 | 2.92E-06 | 3.26E-06 | 3.45E-06 | 3.52E-06 | 3.63E-06 | 3.09E-06 |
| 15 | 8.64E-07 | 1.81E-06 | 2.46E-06 | 2.94E-06 | 3.28E-06 | 3.58E-06 | 3.71E-06 | 3.98E-06 | 3.44E-06 |
| 16 | 7.10E-07 | 1.68E-06 | 2.35E-06 | 2.88E-06 | 3.24E-06 | 3.65E-06 | 3.86E-06 | 4.24E-06 | 3.73E-06 |
| 17 | 5.56E-07 | 1.55E-06 | 2.24E-06 | 2.82E-06 | 3.21E-06 | 3.72E-06 | 4.01E-06 | 4.49E-06 | 4.01E-06 |
| 18 | 4.02E-07 | 1.42E-06 | 2.12E-06 | 2.72E-06 | 3.14E-06 | 3.75E-06 | 4.11E-06 | 4.68E-06 | 4.26E-06 |
| 19 | 2.49E-07 | 1.28E-06 | 2.00E-06 | 2.62E-06 | 3.06E-06 | 3.75E-06 | 4.19E-06 | 4.85E-06 | 4.47E-06 |
| 20 | 9.48E-08 | 1.15E-06 | 1.87E-06 | 2.50E-06 | 2.97E-06 | 3.71E-06 | 4.23E-06 | 4.96E-06 | 4.64E-06 |
| 21 | 0.00E+00 | 1.03E-06 | 1.74E-06 | 2.37E-06 | 2.86E-06 | 3.65E-06 | 4.23E-06 | 5.03E-06 | 4.78E-06 |
| 22 | 0.00E+00 | 9.08E-07 | 1.62E-06 | 2.24E-06 | 2.75E-06 | 3.57E-06 | 4.22E-06 | 5.06E-06 | 4.88E-06 |
| 23 | 0.00E+00 | 7.93E-07 | 1.49E-06 | 2.11E-06 | 2.65E-06 | 3.48E-06 | 4.17E-06 | 5.05E-06 | 4.96E-06 |
| 24 | 0.00E+00 | 6.84E-07 | 1.37E-06 | 1.99E-06 | 2.53E-06 | 3.38E-06 | 4.11E-06 | 5.02E-06 | 5.00E-06 |
| 25 | 0.00E+00 | 5.78E-07 | 1.27E-06 | 1.87E-06 | 2.41E-06 | 3.28E-06 | 4.03E-06 | 4.98E-06 | 5.03E-06 |
| 26 | 0.00E+00 | 4.75E-07 | 1.16E-06 | 1.76E-06 | 2.30E-06 | 3.18E-06 | 3.94E-06 | 4.93E-06 | 5.03E-06 |
| 27 | 0.00E+00 | 3.73E-07 | 1.06E-06 | 1.65E-06 | 2.19E-06 | 3.07E-06 | 3.84E-06 | 4.85E-06 | 5.01E-06 |
| 28 | 0.00E+00 | 2.70E-07 | 9.69E-07 | 1.54E-06 | 2.07E-06 | 2.97E-06 | 3.74E-06 | 4.77E-06 | 4.98E-06 |
| 29 | 0.00E+00 | 1.68E-07 | 8.79E-07 | 1.44E-06 | 1.96E-06 | 2.86E-06 | 3.64E-06 | 4.67E-06 | 4.93E-06 |
| 30 | 0.00E+00 | 6.06E-08 | 7.91E-07 | 1.35E-06 | 1.85E-06 | 2.76E-06 | 3.54E-06 | 4.55E-06 | 4.87E-06 |
| 31 | 0.00E+00 | 0.00E+00 | 7.08E-07 | 1.26E-06 | 1.75E-06 | 2.65E-06 | 3.44E-06 | 4.43E-06 | 4.81E-06 |
| 32 | 0.00E+00 | 0.00E+00 | 6.26E-07 | 1.17E-06 | 1.65E-06 | 2.53E-06 | 3.34E-06 | 4.30E-06 | 4.74E-06 |
| 33 | 0.00E+00 | 0.00E+00 | 5.47E-07 | 1.09E-06 | 1.56E-06 | 2.42E-06 | 3.23E-06 | 4.17E-06 | 4.67E-06 |
| 34 | 0.00E+00 | 0.00E+00 | 4.71E-07 | 1.01E-06 | 1.48E-06 | 2.31E-06 | 3.12E-06 | 4.05E-06 | 4.58E-06 |
| 35 | 0.00E+00 | 0.00E+00 | 3.96E-07 | 9.39E-07 | 1.40E-06 | 2.20E-06 | 3.01E-06 | 3.93E-06 | 4.49E-06 |
| 36 | 0.00E+00 | 0.00E+00 | 3.23E-07 | 8.66E-07 | 1.32E-06 | 2.09E-06 | 2.90E-06 | 3.83E-06 | 4.38E-06 |
| 37 | 0.00E+00 | 0.00E+00 | 2.51E-07 | 7.93E-07 | 1.24E-06 | 2.00E-06 | 2.79E-06 | 3.72E-06 | 4.27E-06 |
| 38 | 0.00E+00 | 0.00E+00 | 1.81E-07 | 7.25E-07 | 1.16E-06 | 1.90E-06 | 2.68E-06 | 3.62E-06 | 4.16E-06 |
| 39 | 0.00E+00 | 0.00E+00 | 1.12E-07 | 6.56E-07 | 1.09E-06 | 1.83E-06 | 2.58E-06 | 3.53E-06 | 4.05E-06 |
| 40 | 0.00E+00 | 0.00E+00 | 4.86E-08 | 5.91E-07 | 1.02E-06 | 1.75E-06 | 2.48E-06 | 3.43E-06 | 3.94E-06 |
| 41 | 0.00E+00 | 0.00E+00 | 0.00E+00 | 5.28E-07 | 9.52E-07 | 1.69E-06 | 2.39E-06 | 3.32E-06 | 3.84E-06 |
| 42 | 0.00E+00 | 0.00E+00 | 0.00E+00 | 4.68E-07 | 8.91E-07 | 1.62E-06 | 2.30E-06 | 3.22E-06 | 3.74E-06 |
| 43 | 0.00E+00 | 0.00E+00 | 0.00E+00 | 4.08E-07 | 8.34E-07 | 1.55E-06 | 2.21E-06 | 3.13E-06 | 3.64E-06 |
| 44 | 0.00E+00 | 0.00E+00 | 0.00E+00 | 3.54E-07 | 7.76E-07 | 1.49E-06 | 2.14E-06 | 3.03E-06 | 3.55E-06 |
| 45 | 0.00E+00 | 0.00E+00 | 0.00E+00 | 3.00E-07 | 7.20E-07 | 1.42E-06 | 2.07E-06 | 2.94E-06 | 3.45E-06 |
| 46 | 0.00E+00 | 0.00E+00 | 0.00E+00 | 2.46E-07 | 6.65E-07 | 1.35E-06 | 2.00E-06 | 2.85E-06 | 3.36E-06 |
| 47 | 0.00E+00 | 0.00E+00 | 0.00E+00 | 1.94E-07 | 6.13E-07 | 1.29E-06 | 1.93E-06 | 2.76E-06 | 3.28E-06 |
| 48 | 0.00E+00 | 0.00E+00 | 0.00E+00 | 1.42E-07 | 5.62E-07 | 1.23E-06 | 1.86E-06 | 2.67E-06 | 3.19E-06 |
| 49 | 0.00E+00 | 0.00E+00 | 0.00E+00 | 9.04E-08 | 5.14E-07 | 1.17E-06 | 1.78E-06 | 2.59E-06 | 3.11E-06 |
| 50 | 0.00E+00 | 0.00E+00 | 0.00E+00 | 3.74E-08 | 4.67E-07 | 1.12E-06 | 1.72E-06 | 2.51E-06 | 3.03E-06 |
| 51 | 0.00E+00 | 0.00E+00 | 0.00E+00 | 0.00E+00 | 4.21E-07 | 1.06E-06 | 1.65E-06 | 2.43E-06 | 2.95E-06 |
| 52 | 0.00E+00 | 0.00E+00 | 0.00E+00 | 0.00E+00 | 3.74E-07 | 1.01E-06 | 1.59E-06 | 2.37E-06 | 2.87E-06 |
| 53 | 0.00E+00 | 0.00E+00 | 0.00E+00 | 0.00E+00 | 3.28E-07 | 9.51E-07 | 1.54E-06 | 2.31E-06 | 2.80E-06 |
| 54 | 0.00E+00 | 0.00E+00 | 0.00E+00 | 0.00E+00 | 2.83E-07 | 9.00E-07 | 1.50E-06 | 2.25E-06 | 2.73E-06 |
| 55 | 0.00E+00 | 0.00E+00 | 0.00E+00 | 0.00E+00 | 2.39E-07 | 8.52E-07 | 1.45E-06 | 2.19E-06 | 2.67E-06 |
| 56 | 0.00E+00 | 0.00E+00 | 0.00E+00 | 0.00E+00 | 1.95E-07 | 8.09E-07 | 1.39E-06 | 2.13E-06 | 2.61E-06 |
| 57 | 0.00E+00 | 0.00E+00 | 0.00E+00 | 0.00E+00 | 1.52E-07 | 7.68E-07 | 1.33E-06 | 2.05E-06 | 2.55E-06 |
| 58 | 0.00E+00 | 0.00E+00 | 0.00E+00 | 0.00E+00 | 1.09E-07 | 7.31E-07 | 1.25E-06 | 1.97E-06 | 2.48E-06 |
| 59 | 0.00E+00 | 0.00E+00 | 0.00E+00 | 0.00E+00 | 6.87E-08 | 8.38E-07 | 2.21E-06 | 4.78E-06 | 6.46E-06 |
| 60 | 0.00E+00 | 0.00E+00 | 0.00E+00 | 0.00E+00 | 2.65E-08 | 9.30E-07 | 2.92E-06 | 7.02E-06 | 9.75E-06 |
| 61 | 0.00E+00 | 0.00E+00 | 0.00E+00 | 0.00E+00 | 0.00E+00 | 6.02E-07 | 1.08E-06 | 1.84E-06 | 2.35E-06 |
| 62 | 0.00E+00 | 0.00E+00 | 0.00E+00 | 0.00E+00 | 0.00E+00 | 5.87E-07 | 1.17E-06 | 1.91E-06 | 2.53E-06 |
| 63 | 0.00E+00 | 0.00E+00 | 0.00E+00 | 0.00E+00 | 0.00E+00 | 5.85E-07 | 1.02E-06 | 1.60E-06 | 2.06E-06 |
| 64 | 0.00E+00 | 0.00E+00 | 0.00E+00 | 0.00E+00 | 0.00E+00 | 5.07E-07 | 1.00E-06 | 1.52E-06 | 2.01E-06 |
| 65 | 0.00E+00 | 0.00E+00 | 0.00E+00 | 0.00E+00 | 0.00E+00 | 4.72E-07 | 9.60E-07 | 1.53E-06 | 1.99E-06 |
| 66 | 0.00E+00 | 0.00E+00 | 0.00E+00 | 0.00E+00 | 0.00E+00 | 4.44E-07 | 9.03E-07 | 1.50E-06 | 1.95E-06 |
| 67 | 0.00E+00 | 0.00E+00 | 0.00E+00 | 0.00E+00 | 0.00E+00 | 4.22E-07 | 8.33E-07 | 1.45E-06 | 1.91E-06 |
| 68 | 0.00E+00 | 0.00E+00 | 0.00E+00 | 0.00E+00 | 0.00E+00 | 4.77E-07 | 1.49E-06 | 3.19E-06 | 4.53E-06 |
| 69 | 0.00E+00 | 0.00E+00 | 0.00E+00 | 0.00E+00 | 0.00E+00 | 3.34E-07 | 7.90E-07 | 1.41E-06 | 1.73E-06 |
| 70 | 0.00E+00 | 0.00E+00 | 0.00E+00 | 0.00E+00 | 0.00E+00 | 3.23E-07 | 9.28E-07 | 1.81E-06 | 2.42E-06 |
| 71 | 0.00E+00 | 0.00E+00 | 0.00E+00 | 0.00E+00 | 0.00E+00 | 2.73E-07 | 7.33E-07 | 1.22E-06 | 1.44E-06 |
| 72 | 0.00E+00 | 0.00E+00 | 0.00E+00 | 0.00E+00 | 0.00E+00 | 2.45E-07 | 6.51E-07 | 1.19E-06 | 1.45E-06 |
| 73 | 0.00E+00 | 0.00E+00 | 0.00E+00 | 0.00E+00 | 0.00E+00 | 2.12E-07 | 6.19E-07 | 1.14E-06 | 1.41E-06 |
| 74 | 0.00E+00 | 0.00E+00 | 0.00E+00 | 0.00E+00 | 0.00E+00 | 1.82E-07 | 5.91E-07 | 1.11E-06 | 1.38E-06 |
| 75 | 0.00E+00 | 0.00E+00 | 0.00E+00 | 0.00E+00 | 0.00E+00 | 1.53E-07 | 5.64E-07 | 1.08E-06 | 1.34E-06 |
| 76 | 0.00E+00 | 0.00E+00 | 0.00E+00 | 0.00E+00 | 0.00E+00 | 1.25E-07 | 5.40E-07 | 1.06E-06 | 1.31E-06 |
| 77 | 0.00E+00 | 0.00E+00 | 0.00E+00 | 0.00E+00 | 0.00E+00 | 9.74E-08 | 5.18E-07 | 1.02E-06 | 1.28E-06 |
| 78 | 0.00E+00 | 0.00E+00 | 0.00E+00 | 0.00E+00 | 0.00E+00 | 7.02E-08 | 4.98E-07 | 9.90E-07 | 1.25E-06 |
| 79 | 0.00E+00 | 0.00E+00 | 0.00E+00 | 0.00E+00 | 0.00E+00 | 4.26E-08 | 4.77E-07 | 9.59E-07 | 1.22E-06 |
| 80 | 0.00E+00 | 0.00E+00 | 0.00E+00 | 0.00E+00 | 0.00E+00 | 1.47E-08 | 4.54E-07 | 9.22E-07 | 1.20E-06 |
| 81 | 0.00E+00 | 0.00E+00 | 0.00E+00 | 0.00E+00 | 0.00E+00 | 0.00E+00 | 4.29E-07 | 8.87E-07 | 1.17E-06 |
| 82 | 0.00E+00 | 0.00E+00 | 0.00E+00 | 0.00E+00 | 0.00E+00 | 0.00E+00 | 4.05E-07 | 8.57E-07 | 1.15E-06 |
| 83 | 0.00E+00 | 0.00E+00 | 0.00E+00 | 0.00E+00 | 0.00E+00 | 0.00E+00 | 3.80E-07 | 8.27E-07 | 1.12E-06 |
| 84 | 0.00E+00 | 0.00E+00 | 0.00E+00 | 0.00E+00 | 0.00E+00 | 0.00E+00 | 3.55E-07 | 7.97E-07 | 1.10E-06 |
| 85 | 0.00E+00 | 0.00E+00 | 0.00E+00 | 0.00E+00 | 0.00E+00 | 0.00E+00 | 3.33E-07 | 7.72E-07 | 1.07E-06 |
| 86 | 0.00E+00 | 0.00E+00 | 0.00E+00 | 0.00E+00 | 0.00E+00 | 0.00E+00 | 3.10E-07 | 7.51E-07 | 1.04E-06 |
| 87 | 0.00E+00 | 0.00E+00 | 0.00E+00 | 0.00E+00 | 0.00E+00 | 0.00E+00 | 2.86E-07 | 7.30E-07 | 1.02E-06 |
| 88 | 0.00E+00 | 0.00E+00 | 0.00E+00 | 0.00E+00 | 0.00E+00 | 0.00E+00 | 2.62E-07 | 7.11E-07 | 9.93E-07 |
| 89 | 0.00E+00 | 0.00E+00 | 0.00E+00 | 0.00E+00 | 0.00E+00 | 0.00E+00 | 2.37E-07 | 6.91E-07 | 9.69E-07 |
| 90 | 0.00E+00 | 0.00E+00 | 0.00E+00 | 0.00E+00 | 0.00E+00 | 0.00E+00 | 2.13E-07 | 6.72E-07 | 9.46E-07 |
| 91 | 0.00E+00 | 0.00E+00 | 0.00E+00 | 0.00E+00 | 0.00E+00 | 0.00E+00 | 1.92E-07 | 6.51E-07 | 9.24E-07 |
| 92 | 0.00E+00 | 0.00E+00 | 0.00E+00 | 0.00E+00 | 0.00E+00 | 0.00E+00 | 1.71E-07 | 6.30E-07 | 9.00E-07 |
| 93 | 0.00E+00 | 0.00E+00 | 0.00E+00 | 0.00E+00 | 0.00E+00 | 0.00E+00 | 1.52E-07 | 6.10E-07 | 8.76E-07 |
| 94 | 0.00E+00 | 0.00E+00 | 0.00E+00 | 0.00E+00 | 0.00E+00 | 0.00E+00 | 1.34E-07 | 5.89E-07 | 8.49E-07 |
| 95 | 0.00E+00 | 0.00E+00 | 0.00E+00 | 0.00E+00 | 0.00E+00 | 0.00E+00 | 1.14E-07 | 5.67E-07 | 8.21E-07 |
| 96 | 0.00E+00 | 0.00E+00 | 0.00E+00 | 0.00E+00 | 0.00E+00 | 0.00E+00 | 9.32E-08 | 5.43E-07 | 7.93E-07 |
| 97 | 0.00E+00 | 0.00E+00 | 0.00E+00 | 0.00E+00 | 0.00E+00 | 0.00E+00 | 7.22E-08 | 5.19E-07 | 7.68E-07 |
| 98 | 0.00E+00 | 0.00E+00 | 0.00E+00 | 0.00E+00 | 0.00E+00 | 0.00E+00 | 5.12E-08 | 4.96E-07 | 7.45E-07 |
| 99 | 0.00E+00 | 0.00E+00 | 0.00E+00 | 0.00E+00 | 0.00E+00 | 0.00E+00 | 3.00E-08 | 4.76E-07 | 7.24E-07 |
| 100 | 0.00E+00 | 0.00E+00 | 0.00E+00 | 0.00E+00 | 0.00E+00 | 0.00E+00 | 1.19E-08 | 4.58E-07 | 7.08E-07 |
| 101 | 0.00E+00 | 0.00E+00 | 0.00E+00 | 0.00E+00 | 0.00E+00 | 0.00E+00 | 0.00E+00 | 4.44E-07 | 6.91E-07 |
| 102 | 0.00E+00 | 0.00E+00 | 0.00E+00 | 0.00E+00 | 0.00E+00 | 0.00E+00 | 0.00E+00 | 4.30E-07 | 6.74E-07 |
| 103 | 0.00E+00 | 0.00E+00 | 0.00E+00 | 0.00E+00 | 0.00E+00 | 0.00E+00 | 0.00E+00 | 4.13E-07 | 6.57E-07 |
| 104 | 0.00E+00 | 0.00E+00 | 0.00E+00 | 0.00E+00 | 0.00E+00 | 0.00E+00 | 0.00E+00 | 3.97E-07 | 6.39E-07 |
| 105 | 0.00E+00 | 0.00E+00 | 0.00E+00 | 0.00E+00 | 0.00E+00 | 0.00E+00 | 0.00E+00 | 3.77E-07 | 6.18E-07 |
| 106 | 0.00E+00 | 0.00E+00 | 0.00E+00 | 0.00E+00 | 0.00E+00 | 0.00E+00 | 0.00E+00 | 3.56E-07 | 6.00E-07 |
| 107 | 0.00E+00 | 0.00E+00 | 0.00E+00 | 0.00E+00 | 0.00E+00 | 0.00E+00 | 0.00E+00 | 3.37E-07 | 5.82E-07 |
| 108 | 0.00E+00 | 0.00E+00 | 0.00E+00 | 0.00E+00 | 0.00E+00 | 0.00E+00 | 0.00E+00 | 3.23E-07 | 5.66E-07 |
| 109 | 0.00E+00 | 0.00E+00 | 0.00E+00 | 0.00E+00 | 0.00E+00 | 0.00E+00 | 0.00E+00 | 3.08E-07 | 5.52E-07 |
| 110 | 0.00E+00 | 0.00E+00 | 0.00E+00 | 0.00E+00 | 0.00E+00 | 0.00E+00 | 0.00E+00 | 2.96E-07 | 5.38E-07 |
| 111 | 0.00E+00 | 0.00E+00 | 0.00E+00 | 0.00E+00 | 0.00E+00 | 0.00E+00 | 0.00E+00 | 2.83E-07 | 5.24E-07 |
| 112 | 0.00E+00 | 0.00E+00 | 0.00E+00 | 0.00E+00 | 0.00E+00 | 0.00E+00 | 0.00E+00 | 2.68E-07 | 5.10E-07 |
| 113 | 0.00E+00 | 0.00E+00 | 0.00E+00 | 0.00E+00 | 0.00E+00 | 0.00E+00 | 0.00E+00 | 2.48E-07 | 4.93E-07 |
| 114 | 0.00E+00 | 0.00E+00 | 0.00E+00 | 0.00E+00 | 0.00E+00 | 0.00E+00 | 0.00E+00 | 2.28E-07 | 4.74E-07 |
| 115 | 0.00E+00 | 0.00E+00 | 0.00E+00 | 0.00E+00 | 0.00E+00 | 0.00E+00 | 0.00E+00 | 2.07E-07 | 4.54E-07 |
| 116 | 0.00E+00 | 0.00E+00 | 0.00E+00 | 0.00E+00 | 0.00E+00 | 0.00E+00 | 0.00E+00 | 1.88E-07 | 4.34E-07 |
| 117 | 0.00E+00 | 0.00E+00 | 0.00E+00 | 0.00E+00 | 0.00E+00 | 0.00E+00 | 0.00E+00 | 1.71E-07 | 4.14E-07 |
| 118 | 0.00E+00 | 0.00E+00 | 0.00E+00 | 0.00E+00 | 0.00E+00 | 0.00E+00 | 0.00E+00 | 1.57E-07 | 3.96E-07 |
| 119 | 0.00E+00 | 0.00E+00 | 0.00E+00 | 0.00E+00 | 0.00E+00 | 0.00E+00 | 0.00E+00 | 1.45E-07 | 3.80E-07 |
| 120 | 0.00E+00 | 0.00E+00 | 0.00E+00 | 0.00E+00 | 0.00E+00 | 0.00E+00 | 0.00E+00 | 1.34E-07 | 3.65E-07 |
| 121 | 0.00E+00 | 0.00E+00 | 0.00E+00 | 0.00E+00 | 0.00E+00 | 0.00E+00 | 0.00E+00 | 1.22E-07 | 3.51E-07 |
| 122 | 0.00E+00 | 0.00E+00 | 0.00E+00 | 0.00E+00 | 0.00E+00 | 0.00E+00 | 0.00E+00 | 1.10E-07 | 3.39E-07 |
| 123 | 0.00E+00 | 0.00E+00 | 0.00E+00 | 0.00E+00 | 0.00E+00 | 0.00E+00 | 0.00E+00 | 9.59E-08 | 3.26E-07 |
| 124 | 0.00E+00 | 0.00E+00 | 0.00E+00 | 0.00E+00 | 0.00E+00 | 0.00E+00 | 0.00E+00 | 8.20E-08 | 3.14E-07 |
| 125 | 0.00E+00 | 0.00E+00 | 0.00E+00 | 0.00E+00 | 0.00E+00 | 0.00E+00 | 0.00E+00 | 6.84E-08 | 3.01E-07 |
| 126 | 0.00E+00 | 0.00E+00 | 0.00E+00 | 0.00E+00 | 0.00E+00 | 0.00E+00 | 0.00E+00 | 5.47E-08 | 2.88E-07 |
| 127 | 0.00E+00 | 0.00E+00 | 0.00E+00 | 0.00E+00 | 0.00E+00 | 0.00E+00 | 0.00E+00 | 4.23E-08 | 2.74E-07 |
| 128 | 0.00E+00 | 0.00E+00 | 0.00E+00 | 0.00E+00 | 0.00E+00 | 0.00E+00 | 0.00E+00 | 3.00E-08 | 2.59E-07 |
| 129 | 0.00E+00 | 0.00E+00 | 0.00E+00 | 0.00E+00 | 0.00E+00 | 0.00E+00 | 0.00E+00 | 1.92E-08 | 2.44E-07 |
| 130 | 0.00E+00 | 0.00E+00 | 0.00E+00 | 0.00E+00 | 0.00E+00 | 0.00E+00 | 0.00E+00 | 1.06E-08 | 2.29E-07 |
| 131 | 0.00E+00 | 0.00E+00 | 0.00E+00 | 0.00E+00 | 0.00E+00 | 0.00E+00 | 0.00E+00 | 0.00E+00 | 2.14E-07 |
| 132 | 0.00E+00 | 0.00E+00 | 0.00E+00 | 0.00E+00 | 0.00E+00 | 0.00E+00 | 0.00E+00 | 0.00E+00 | 2.00E-07 |
| 133 | 0.00E+00 | 0.00E+00 | 0.00E+00 | 0.00E+00 | 0.00E+00 | 0.00E+00 | 0.00E+00 | 0.00E+00 | 1.88E-07 |
| 134 | 0.00E+00 | 0.00E+00 | 0.00E+00 | 0.00E+00 | 0.00E+00 | 0.00E+00 | 0.00E+00 | 0.00E+00 | 1.76E-07 |
| 135 | 0.00E+00 | 0.00E+00 | 0.00E+00 | 0.00E+00 | 0.00E+00 | 0.00E+00 | 0.00E+00 | 0.00E+00 | 1.64E-07 |
| 136 | 0.00E+00 | 0.00E+00 | 0.00E+00 | 0.00E+00 | 0.00E+00 | 0.00E+00 | 0.00E+00 | 0.00E+00 | 1.53E-07 |
| 137 | 0.00E+00 | 0.00E+00 | 0.00E+00 | 0.00E+00 | 0.00E+00 | 0.00E+00 | 0.00E+00 | 0.00E+00 | 1.41E-07 |
| 138 | 0.00E+00 | 0.00E+00 | 0.00E+00 | 0.00E+00 | 0.00E+00 | 0.00E+00 | 0.00E+00 | 0.00E+00 | 1.30E-07 |
| 139 | 0.00E+00 | 0.00E+00 | 0.00E+00 | 0.00E+00 | 0.00E+00 | 0.00E+00 | 0.00E+00 | 0.00E+00 | 1.18E-07 |
| 140 | 0.00E+00 | 0.00E+00 | 0.00E+00 | 0.00E+00 | 0.00E+00 | 0.00E+00 | 0.00E+00 | 0.00E+00 | 1.07E-07 |
| 141 | 0.00E+00 | 0.00E+00 | 0.00E+00 | 0.00E+00 | 0.00E+00 | 0.00E+00 | 0.00E+00 | 0.00E+00 | 9.59E-08 |
| 142 | 0.00E+00 | 0.00E+00 | 0.00E+00 | 0.00E+00 | 0.00E+00 | 0.00E+00 | 0.00E+00 | 0.00E+00 | 8.56E-08 |
| 143 | 0.00E+00 | 0.00E+00 | 0.00E+00 | 0.00E+00 | 0.00E+00 | 0.00E+00 | 0.00E+00 | 0.00E+00 | 7.58E-08 |
| 144 | 0.00E+00 | 0.00E+00 | 0.00E+00 | 0.00E+00 | 0.00E+00 | 0.00E+00 | 0.00E+00 | 0.00E+00 | 6.63E-08 |
| 145 | 0.00E+00 | 0.00E+00 | 0.00E+00 | 0.00E+00 | 0.00E+00 | 0.00E+00 | 0.00E+00 | 0.00E+00 | 5.68E-08 |
| 146 | 0.00E+00 | 0.00E+00 | 0.00E+00 | 0.00E+00 | 0.00E+00 | 0.00E+00 | 0.00E+00 | 0.00E+00 | 4.71E-08 |
| 147 | 0.00E+00 | 0.00E+00 | 0.00E+00 | 0.00E+00 | 0.00E+00 | 0.00E+00 | 0.00E+00 | 0.00E+00 | 3.70E-08 |
| 148 | 0.00E+00 | 0.00E+00 | 0.00E+00 | 0.00E+00 | 0.00E+00 | 0.00E+00 | 0.00E+00 | 0.00E+00 | 2.63E-08 |
| 149 | 0.00E+00 | 0.00E+00 | 0.00E+00 | 0.00E+00 | 0.00E+00 | 0.00E+00 | 0.00E+00 | 0.00E+00 | 1.55E-08 |
| 150 | 0.00E+00 | 0.00E+00 | 0.00E+00 | 0.00E+00 | 0.00E+00 | 0.00E+00 | 0.00E+00 | 0.00E+00 | 3.67E-09 |

Table.7. Spectra obtained by MCNP code: Al- 0.4mm

| energy bin (kev)/voltage(kv) | 20kv | 30kv | 40kv | 50kv | 60kv | 80kv | 100kv | 130kv | 150kv |
| --- | --- | --- | --- | --- | --- | --- | --- | --- | --- |
| 1 | 0.00E+00 | 0.00E+00 | 0.00E+00 | 0.00E+00 | 0.00E+00 | 0.00E+00 | 0.00E+00 | 0.00E+00 | 0.00E+00 |
| 2 | 5.65E-85 | 4.39E-85 | 5.56E-85 | 1.52E-85 | 3.36E-85 | 5.79E-85 | 8.81E-85 | 2.01E-84 | 1.11E-84 |
| 3 | 1.14E-32 | 1.60E-33 | 1.37E-33 | 1.35E-33 | 1.28E-33 | 1.23E-33 | 1.28E-33 | 1.70E-33 | 1.90E-33 |
| 4 | 2.78E-18 | 1.75E-18 | 1.63E-18 | 1.47E-18 | 1.35E-18 | 1.25E-18 | 1.20E-18 | 1.42E-18 | 1.24E-18 |
| 5 | 1.07E-12 | 1.44E-12 | 1.41E-12 | 1.37E-12 | 1.26E-12 | 1.13E-12 | 1.07E-12 | 1.17E-12 | 9.85E-13 |
| 6 | 4.61E-10 | 7.29E-10 | 7.94E-10 | 7.96E-10 | 7.78E-10 | 7.13E-10 | 6.51E-10 | 6.61E-10 | 5.70E-10 |
| 7 | 6.07E-09 | 9.45E-09 | 1.08E-08 | 1.15E-08 | 1.17E-08 | 1.11E-08 | 1.06E-08 | 1.10E-08 | 9.06E-09 |
| 8 | 6.92E-08 | 1.08E-07 | 1.30E-07 | 1.47E-07 | 1.54E-07 | 1.58E-07 | 1.52E-07 | 1.60E-07 | 1.38E-07 |
| 9 | 1.57E-07 | 3.23E-07 | 4.44E-07 | 5.48E-07 | 6.23E-07 | 7.29E-07 | 7.87E-07 | 9.17E-07 | 8.11E-07 |
| 10 | 3.45E-07 | 5.78E-07 | 7.58E-07 | 9.24E-07 | 1.04E-06 | 1.19E-06 | 1.27E-06 | 1.44E-06 | 1.27E-06 |
| 11 | 3.91E-07 | 5.92E-07 | 7.62E-07 | 8.73E-07 | 9.48E-07 | 1.00E-06 | 1.00E-06 | 1.10E-06 | 9.41E-07 |
| 12 | 4.17E-07 | 6.91E-07 | 8.72E-07 | 1.00E-06 | 1.06E-06 | 1.10E-06 | 1.11E-06 | 1.17E-06 | 9.68E-07 |
| 13 | 4.51E-07 | 7.77E-07 | 9.86E-07 | 1.12E-06 | 1.20E-06 | 1.19E-06 | 1.19E-06 | 1.22E-06 | 1.04E-06 |
| 14 | 4.96E-07 | 9.37E-07 | 1.23E-06 | 1.42E-06 | 1.59E-06 | 1.68E-06 | 1.71E-06 | 1.77E-06 | 1.50E-06 |
| 15 | 5.01E-07 | 1.06E-06 | 1.45E-06 | 1.74E-06 | 1.93E-06 | 2.12E-06 | 2.20E-06 | 2.37E-06 | 2.05E-06 |
| 16 | 4.38E-07 | 1.07E-06 | 1.50E-06 | 1.85E-06 | 2.09E-06 | 2.36E-06 | 2.51E-06 | 2.76E-06 | 2.44E-06 |
| 17 | 3.79E-07 | 1.08E-06 | 1.57E-06 | 1.98E-06 | 2.25E-06 | 2.63E-06 | 2.83E-06 | 3.18E-06 | 2.85E-06 |
| 18 | 2.87E-07 | 1.05E-06 | 1.58E-06 | 2.03E-06 | 2.34E-06 | 2.81E-06 | 3.09E-06 | 3.52E-06 | 3.21E-06 |
| 19 | 1.93E-07 | 1.00E-06 | 1.56E-06 | 2.05E-06 | 2.40E-06 | 2.95E-06 | 3.31E-06 | 3.83E-06 | 3.53E-06 |
| 20 | 7.99E-08 | 9.39E-07 | 1.52E-06 | 2.04E-06 | 2.43E-06 | 3.05E-06 | 3.47E-06 | 4.08E-06 | 3.82E-06 |
| 21 | 0.00E+00 | 8.68E-07 | 1.47E-06 | 2.01E-06 | 2.42E-06 | 3.10E-06 | 3.59E-06 | 4.27E-06 | 4.06E-06 |
| 22 | 0.00E+00 | 7.86E-07 | 1.40E-06 | 1.95E-06 | 2.39E-06 | 3.10E-06 | 3.66E-06 | 4.40E-06 | 4.25E-06 |
| 23 | 0.00E+00 | 6.97E-07 | 1.31E-06 | 1.86E-06 | 2.33E-06 | 3.07E-06 | 3.68E-06 | 4.46E-06 | 4.38E-06 |
| 24 | 0.00E+00 | 6.10E-07 | 1.23E-06 | 1.78E-06 | 2.26E-06 | 3.03E-06 | 3.68E-06 | 4.50E-06 | 4.49E-06 |
| 25 | 0.00E+00 | 5.23E-07 | 1.15E-06 | 1.70E-06 | 2.19E-06 | 2.98E-06 | 3.67E-06 | 4.53E-06 | 4.58E-06 |
| 26 | 0.00E+00 | 4.36E-07 | 1.07E-06 | 1.62E-06 | 2.12E-06 | 2.93E-06 | 3.64E-06 | 4.55E-06 | 4.65E-06 |
| 27 | 0.00E+00 | 3.48E-07 | 9.94E-07 | 1.54E-06 | 2.05E-06 | 2.88E-06 | 3.61E-06 | 4.55E-06 | 4.70E-06 |
| 28 | 0.00E+00 | 2.55E-07 | 9.21E-07 | 1.47E-06 | 1.97E-06 | 2.83E-06 | 3.56E-06 | 4.54E-06 | 4.74E-06 |
| 29 | 0.00E+00 | 1.62E-07 | 8.46E-07 | 1.39E-06 | 1.89E-06 | 2.76E-06 | 3.51E-06 | 4.51E-06 | 4.76E-06 |
| 30 | 0.00E+00 | 5.95E-08 | 7.71E-07 | 1.31E-06 | 1.81E-06 | 2.69E-06 | 3.46E-06 | 4.44E-06 | 4.76E-06 |
| 31 | 0.00E+00 | 0.00E+00 | 6.96E-07 | 1.24E-06 | 1.72E-06 | 2.60E-06 | 3.39E-06 | 4.35E-06 | 4.73E-06 |
| 32 | 0.00E+00 | 0.00E+00 | 6.19E-07 | 1.16E-06 | 1.63E-06 | 2.51E-06 | 3.30E-06 | 4.25E-06 | 4.69E-06 |
| 33 | 0.00E+00 | 0.00E+00 | 5.43E-07 | 1.08E-06 | 1.55E-06 | 2.41E-06 | 3.21E-06 | 4.14E-06 | 4.64E-06 |
| 34 | 0.00E+00 | 0.00E+00 | 4.69E-07 | 1.01E-06 | 1.47E-06 | 2.30E-06 | 3.11E-06 | 4.03E-06 | 4.57E-06 |
| 35 | 0.00E+00 | 0.00E+00 | 3.96E-07 | 9.39E-07 | 1.40E-06 | 2.20E-06 | 3.01E-06 | 3.94E-06 | 4.49E-06 |
| 36 | 0.00E+00 | 0.00E+00 | 3.24E-07 | 8.69E-07 | 1.33E-06 | 2.10E-06 | 2.91E-06 | 3.84E-06 | 4.40E-06 |
| 37 | 0.00E+00 | 0.00E+00 | 2.52E-07 | 7.99E-07 | 1.25E-06 | 2.01E-06 | 2.81E-06 | 3.75E-06 | 4.30E-06 |
| 38 | 0.00E+00 | 0.00E+00 | 1.83E-07 | 7.33E-07 | 1.18E-06 | 1.93E-06 | 2.71E-06 | 3.67E-06 | 4.21E-06 |
| 39 | 0.00E+00 | 0.00E+00 | 1.13E-07 | 6.66E-07 | 1.11E-06 | 1.85E-06 | 2.61E-06 | 3.58E-06 | 4.11E-06 |
| 40 | 0.00E+00 | 0.00E+00 | 4.95E-08 | 6.00E-07 | 1.04E-06 | 1.78E-06 | 2.52E-06 | 3.49E-06 | 4.01E-06 |
| 41 | 0.00E+00 | 0.00E+00 | 0.00E+00 | 5.38E-07 | 9.70E-07 | 1.72E-06 | 2.43E-06 | 3.39E-06 | 3.92E-06 |
| 42 | 0.00E+00 | 0.00E+00 | 0.00E+00 | 4.78E-07 | 9.10E-07 | 1.65E-06 | 2.35E-06 | 3.29E-06 | 3.82E-06 |
| 43 | 0.00E+00 | 0.00E+00 | 0.00E+00 | 4.17E-07 | 8.52E-07 | 1.59E-06 | 2.26E-06 | 3.20E-06 | 3.73E-06 |
| 44 | 0.00E+00 | 0.00E+00 | 0.00E+00 | 3.62E-07 | 7.94E-07 | 1.52E-06 | 2.19E-06 | 3.10E-06 | 3.63E-06 |
| 45 | 0.00E+00 | 0.00E+00 | 0.00E+00 | 3.07E-07 | 7.38E-07 | 1.45E-06 | 2.12E-06 | 3.01E-06 | 3.54E-06 |
| 46 | 0.00E+00 | 0.00E+00 | 0.00E+00 | 2.52E-07 | 6.82E-07 | 1.39E-06 | 2.05E-06 | 2.93E-06 | 3.45E-06 |
| 47 | 0.00E+00 | 0.00E+00 | 0.00E+00 | 1.99E-07 | 6.29E-07 | 1.33E-06 | 1.98E-06 | 2.84E-06 | 3.37E-06 |
| 48 | 0.00E+00 | 0.00E+00 | 0.00E+00 | 1.46E-07 | 5.78E-07 | 1.27E-06 | 1.91E-06 | 2.75E-06 | 3.28E-06 |
| 49 | 0.00E+00 | 0.00E+00 | 0.00E+00 | 9.30E-08 | 5.29E-07 | 1.21E-06 | 1.84E-06 | 2.67E-06 | 3.20E-06 |
| 50 | 0.00E+00 | 0.00E+00 | 0.00E+00 | 3.85E-08 | 4.81E-07 | 1.15E-06 | 1.77E-06 | 2.59E-06 | 3.12E-06 |
| 51 | 0.00E+00 | 0.00E+00 | 0.00E+00 | 0.00E+00 | 4.34E-07 | 1.10E-06 | 1.70E-06 | 2.51E-06 | 3.04E-06 |
| 52 | 0.00E+00 | 0.00E+00 | 0.00E+00 | 0.00E+00 | 3.86E-07 | 1.04E-06 | 1.64E-06 | 2.44E-06 | 2.96E-06 |
| 53 | 0.00E+00 | 0.00E+00 | 0.00E+00 | 0.00E+00 | 3.39E-07 | 9.81E-07 | 1.59E-06 | 2.38E-06 | 2.89E-06 |
| 54 | 0.00E+00 | 0.00E+00 | 0.00E+00 | 0.00E+00 | 2.92E-07 | 9.29E-07 | 1.55E-06 | 2.32E-06 | 2.82E-06 |
| 55 | 0.00E+00 | 0.00E+00 | 0.00E+00 | 0.00E+00 | 2.47E-07 | 8.80E-07 | 1.50E-06 | 2.27E-06 | 2.76E-06 |
| 56 | 0.00E+00 | 0.00E+00 | 0.00E+00 | 0.00E+00 | 2.02E-07 | 8.36E-07 | 1.44E-06 | 2.20E-06 | 2.70E-06 |
| 57 | 0.00E+00 | 0.00E+00 | 0.00E+00 | 0.00E+00 | 1.57E-07 | 7.94E-07 | 1.38E-06 | 2.12E-06 | 2.64E-06 |
| 58 | 0.00E+00 | 0.00E+00 | 0.00E+00 | 0.00E+00 | 1.13E-07 | 7.56E-07 | 1.29E-06 | 2.03E-06 | 2.56E-06 |
| 59 | 0.00E+00 | 0.00E+00 | 0.00E+00 | 0.00E+00 | 7.11E-08 | 8.67E-07 | 2.29E-06 | 4.94E-06 | 6.69E-06 |
| 60 | 0.00E+00 | 0.00E+00 | 0.00E+00 | 0.00E+00 | 2.74E-08 | 9.63E-07 | 3.03E-06 | 7.27E-06 | 1.01E-05 |
| 61 | 0.00E+00 | 0.00E+00 | 0.00E+00 | 0.00E+00 | 0.00E+00 | 6.23E-07 | 1.12E-06 | 1.90E-06 | 2.43E-06 |
| 62 | 0.00E+00 | 0.00E+00 | 0.00E+00 | 0.00E+00 | 0.00E+00 | 6.08E-07 | 1.22E-06 | 1.98E-06 | 2.62E-06 |
| 63 | 0.00E+00 | 0.00E+00 | 0.00E+00 | 0.00E+00 | 0.00E+00 | 6.06E-07 | 1.06E-06 | 1.66E-06 | 2.14E-06 |
| 64 | 0.00E+00 | 0.00E+00 | 0.00E+00 | 0.00E+00 | 0.00E+00 | 5.25E-07 | 1.04E-06 | 1.58E-06 | 2.08E-06 |
| 65 | 0.00E+00 | 0.00E+00 | 0.00E+00 | 0.00E+00 | 0.00E+00 | 4.89E-07 | 9.94E-07 | 1.58E-06 | 2.06E-06 |
| 66 | 0.00E+00 | 0.00E+00 | 0.00E+00 | 0.00E+00 | 0.00E+00 | 4.60E-07 | 9.35E-07 | 1.56E-06 | 2.02E-06 |
| 67 | 0.00E+00 | 0.00E+00 | 0.00E+00 | 0.00E+00 | 0.00E+00 | 4.37E-07 | 8.63E-07 | 1.50E-06 | 1.98E-06 |
| 68 | 0.00E+00 | 0.00E+00 | 0.00E+00 | 0.00E+00 | 0.00E+00 | 4.95E-07 | 1.54E-06 | 3.31E-06 | 4.69E-06 |
| 69 | 0.00E+00 | 0.00E+00 | 0.00E+00 | 0.00E+00 | 0.00E+00 | 3.46E-07 | 8.19E-07 | 1.46E-06 | 1.80E-06 |
| 70 | 0.00E+00 | 0.00E+00 | 0.00E+00 | 0.00E+00 | 0.00E+00 | 3.35E-07 | 9.62E-07 | 1.88E-06 | 2.51E-06 |
| 71 | 0.00E+00 | 0.00E+00 | 0.00E+00 | 0.00E+00 | 0.00E+00 | 2.83E-07 | 7.60E-07 | 1.27E-06 | 1.49E-06 |
| 72 | 0.00E+00 | 0.00E+00 | 0.00E+00 | 0.00E+00 | 0.00E+00 | 2.54E-07 | 6.75E-07 | 1.24E-06 | 1.50E-06 |
| 73 | 0.00E+00 | 0.00E+00 | 0.00E+00 | 0.00E+00 | 0.00E+00 | 2.20E-07 | 6.42E-07 | 1.19E-06 | 1.47E-06 |
| 74 | 0.00E+00 | 0.00E+00 | 0.00E+00 | 0.00E+00 | 0.00E+00 | 1.89E-07 | 6.13E-07 | 1.15E-06 | 1.43E-06 |
| 75 | 0.00E+00 | 0.00E+00 | 0.00E+00 | 0.00E+00 | 0.00E+00 | 1.59E-07 | 5.85E-07 | 1.12E-06 | 1.39E-06 |
| 76 | 0.00E+00 | 0.00E+00 | 0.00E+00 | 0.00E+00 | 0.00E+00 | 1.29E-07 | 5.60E-07 | 1.10E-06 | 1.36E-06 |
| 77 | 0.00E+00 | 0.00E+00 | 0.00E+00 | 0.00E+00 | 0.00E+00 | 1.01E-07 | 5.38E-07 | 1.06E-06 | 1.32E-06 |
| 78 | 0.00E+00 | 0.00E+00 | 0.00E+00 | 0.00E+00 | 0.00E+00 | 7.28E-08 | 5.16E-07 | 1.03E-06 | 1.30E-06 |
| 79 | 0.00E+00 | 0.00E+00 | 0.00E+00 | 0.00E+00 | 0.00E+00 | 4.42E-08 | 4.95E-07 | 9.95E-07 | 1.27E-06 |
| 80 | 0.00E+00 | 0.00E+00 | 0.00E+00 | 0.00E+00 | 0.00E+00 | 1.53E-08 | 4.71E-07 | 9.57E-07 | 1.24E-06 |
| 81 | 0.00E+00 | 0.00E+00 | 0.00E+00 | 0.00E+00 | 0.00E+00 | 0.00E+00 | 4.46E-07 | 9.21E-07 | 1.22E-06 |
| 82 | 0.00E+00 | 0.00E+00 | 0.00E+00 | 0.00E+00 | 0.00E+00 | 0.00E+00 | 4.20E-07 | 8.89E-07 | 1.19E-06 |
| 83 | 0.00E+00 | 0.00E+00 | 0.00E+00 | 0.00E+00 | 0.00E+00 | 0.00E+00 | 3.94E-07 | 8.58E-07 | 1.16E-06 |
| 84 | 0.00E+00 | 0.00E+00 | 0.00E+00 | 0.00E+00 | 0.00E+00 | 0.00E+00 | 3.68E-07 | 8.27E-07 | 1.14E-06 |
| 85 | 0.00E+00 | 0.00E+00 | 0.00E+00 | 0.00E+00 | 0.00E+00 | 0.00E+00 | 3.46E-07 | 8.01E-07 | 1.11E-06 |
| 86 | 0.00E+00 | 0.00E+00 | 0.00E+00 | 0.00E+00 | 0.00E+00 | 0.00E+00 | 3.22E-07 | 7.79E-07 | 1.08E-06 |
| 87 | 0.00E+00 | 0.00E+00 | 0.00E+00 | 0.00E+00 | 0.00E+00 | 0.00E+00 | 2.97E-07 | 7.57E-07 | 1.06E-06 |
| 88 | 0.00E+00 | 0.00E+00 | 0.00E+00 | 0.00E+00 | 0.00E+00 | 0.00E+00 | 2.71E-07 | 7.37E-07 | 1.03E-06 |
| 89 | 0.00E+00 | 0.00E+00 | 0.00E+00 | 0.00E+00 | 0.00E+00 | 0.00E+00 | 2.46E-07 | 7.17E-07 | 1.01E-06 |
| 90 | 0.00E+00 | 0.00E+00 | 0.00E+00 | 0.00E+00 | 0.00E+00 | 0.00E+00 | 2.21E-07 | 6.97E-07 | 9.82E-07 |
| 91 | 0.00E+00 | 0.00E+00 | 0.00E+00 | 0.00E+00 | 0.00E+00 | 0.00E+00 | 1.99E-07 | 6.76E-07 | 9.59E-07 |
| 92 | 0.00E+00 | 0.00E+00 | 0.00E+00 | 0.00E+00 | 0.00E+00 | 0.00E+00 | 1.77E-07 | 6.54E-07 | 9.34E-07 |
| 93 | 0.00E+00 | 0.00E+00 | 0.00E+00 | 0.00E+00 | 0.00E+00 | 0.00E+00 | 1.58E-07 | 6.33E-07 | 9.09E-07 |
| 94 | 0.00E+00 | 0.00E+00 | 0.00E+00 | 0.00E+00 | 0.00E+00 | 0.00E+00 | 1.39E-07 | 6.11E-07 | 8.81E-07 |
| 95 | 0.00E+00 | 0.00E+00 | 0.00E+00 | 0.00E+00 | 0.00E+00 | 0.00E+00 | 1.18E-07 | 5.88E-07 | 8.52E-07 |
| 96 | 0.00E+00 | 0.00E+00 | 0.00E+00 | 0.00E+00 | 0.00E+00 | 0.00E+00 | 9.67E-08 | 5.63E-07 | 8.23E-07 |
| 97 | 0.00E+00 | 0.00E+00 | 0.00E+00 | 0.00E+00 | 0.00E+00 | 0.00E+00 | 7.49E-08 | 5.39E-07 | 7.97E-07 |
| 98 | 0.00E+00 | 0.00E+00 | 0.00E+00 | 0.00E+00 | 0.00E+00 | 0.00E+00 | 5.31E-08 | 5.15E-07 | 7.72E-07 |
| 99 | 0.00E+00 | 0.00E+00 | 0.00E+00 | 0.00E+00 | 0.00E+00 | 0.00E+00 | 3.12E-08 | 4.93E-07 | 7.51E-07 |
| 100 | 0.00E+00 | 0.00E+00 | 0.00E+00 | 0.00E+00 | 0.00E+00 | 0.00E+00 | 1.23E-08 | 4.76E-07 | 7.34E-07 |
| 101 | 0.00E+00 | 0.00E+00 | 0.00E+00 | 0.00E+00 | 0.00E+00 | 0.00E+00 | 0.00E+00 | 4.61E-07 | 7.17E-07 |
| 102 | 0.00E+00 | 0.00E+00 | 0.00E+00 | 0.00E+00 | 0.00E+00 | 0.00E+00 | 0.00E+00 | 4.46E-07 | 6.99E-07 |
| 103 | 0.00E+00 | 0.00E+00 | 0.00E+00 | 0.00E+00 | 0.00E+00 | 0.00E+00 | 0.00E+00 | 4.28E-07 | 6.81E-07 |
| 104 | 0.00E+00 | 0.00E+00 | 0.00E+00 | 0.00E+00 | 0.00E+00 | 0.00E+00 | 0.00E+00 | 4.11E-07 | 6.62E-07 |
| 105 | 0.00E+00 | 0.00E+00 | 0.00E+00 | 0.00E+00 | 0.00E+00 | 0.00E+00 | 0.00E+00 | 3.91E-07 | 6.41E-07 |
| 106 | 0.00E+00 | 0.00E+00 | 0.00E+00 | 0.00E+00 | 0.00E+00 | 0.00E+00 | 0.00E+00 | 3.70E-07 | 6.22E-07 |
| 107 | 0.00E+00 | 0.00E+00 | 0.00E+00 | 0.00E+00 | 0.00E+00 | 0.00E+00 | 0.00E+00 | 3.49E-07 | 6.04E-07 |
| 108 | 0.00E+00 | 0.00E+00 | 0.00E+00 | 0.00E+00 | 0.00E+00 | 0.00E+00 | 0.00E+00 | 3.35E-07 | 5.87E-07 |
| 109 | 0.00E+00 | 0.00E+00 | 0.00E+00 | 0.00E+00 | 0.00E+00 | 0.00E+00 | 0.00E+00 | 3.19E-07 | 5.72E-07 |
| 110 | 0.00E+00 | 0.00E+00 | 0.00E+00 | 0.00E+00 | 0.00E+00 | 0.00E+00 | 0.00E+00 | 3.07E-07 | 5.58E-07 |
| 111 | 0.00E+00 | 0.00E+00 | 0.00E+00 | 0.00E+00 | 0.00E+00 | 0.00E+00 | 0.00E+00 | 2.93E-07 | 5.44E-07 |
| 112 | 0.00E+00 | 0.00E+00 | 0.00E+00 | 0.00E+00 | 0.00E+00 | 0.00E+00 | 0.00E+00 | 2.78E-07 | 5.28E-07 |
| 113 | 0.00E+00 | 0.00E+00 | 0.00E+00 | 0.00E+00 | 0.00E+00 | 0.00E+00 | 0.00E+00 | 2.57E-07 | 5.11E-07 |
| 114 | 0.00E+00 | 0.00E+00 | 0.00E+00 | 0.00E+00 | 0.00E+00 | 0.00E+00 | 0.00E+00 | 2.37E-07 | 4.91E-07 |
| 115 | 0.00E+00 | 0.00E+00 | 0.00E+00 | 0.00E+00 | 0.00E+00 | 0.00E+00 | 0.00E+00 | 2.14E-07 | 4.71E-07 |
| 116 | 0.00E+00 | 0.00E+00 | 0.00E+00 | 0.00E+00 | 0.00E+00 | 0.00E+00 | 0.00E+00 | 1.95E-07 | 4.49E-07 |
| 117 | 0.00E+00 | 0.00E+00 | 0.00E+00 | 0.00E+00 | 0.00E+00 | 0.00E+00 | 0.00E+00 | 1.77E-07 | 4.29E-07 |
| 118 | 0.00E+00 | 0.00E+00 | 0.00E+00 | 0.00E+00 | 0.00E+00 | 0.00E+00 | 0.00E+00 | 1.62E-07 | 4.10E-07 |
| 119 | 0.00E+00 | 0.00E+00 | 0.00E+00 | 0.00E+00 | 0.00E+00 | 0.00E+00 | 0.00E+00 | 1.50E-07 | 3.93E-07 |
| 120 | 0.00E+00 | 0.00E+00 | 0.00E+00 | 0.00E+00 | 0.00E+00 | 0.00E+00 | 0.00E+00 | 1.38E-07 | 3.78E-07 |
| 121 | 0.00E+00 | 0.00E+00 | 0.00E+00 | 0.00E+00 | 0.00E+00 | 0.00E+00 | 0.00E+00 | 1.26E-07 | 3.64E-07 |
| 122 | 0.00E+00 | 0.00E+00 | 0.00E+00 | 0.00E+00 | 0.00E+00 | 0.00E+00 | 0.00E+00 | 1.14E-07 | 3.51E-07 |
| 123 | 0.00E+00 | 0.00E+00 | 0.00E+00 | 0.00E+00 | 0.00E+00 | 0.00E+00 | 0.00E+00 | 9.94E-08 | 3.38E-07 |
| 124 | 0.00E+00 | 0.00E+00 | 0.00E+00 | 0.00E+00 | 0.00E+00 | 0.00E+00 | 0.00E+00 | 8.49E-08 | 3.25E-07 |
| 125 | 0.00E+00 | 0.00E+00 | 0.00E+00 | 0.00E+00 | 0.00E+00 | 0.00E+00 | 0.00E+00 | 7.08E-08 | 3.12E-07 |
| 126 | 0.00E+00 | 0.00E+00 | 0.00E+00 | 0.00E+00 | 0.00E+00 | 0.00E+00 | 0.00E+00 | 5.67E-08 | 2.99E-07 |
| 127 | 0.00E+00 | 0.00E+00 | 0.00E+00 | 0.00E+00 | 0.00E+00 | 0.00E+00 | 0.00E+00 | 4.38E-08 | 2.84E-07 |
| 128 | 0.00E+00 | 0.00E+00 | 0.00E+00 | 0.00E+00 | 0.00E+00 | 0.00E+00 | 0.00E+00 | 3.11E-08 | 2.68E-07 |
| 129 | 0.00E+00 | 0.00E+00 | 0.00E+00 | 0.00E+00 | 0.00E+00 | 0.00E+00 | 0.00E+00 | 1.84E-08 | 2.53E-07 |
| 130 | 0.00E+00 | 0.00E+00 | 0.00E+00 | 0.00E+00 | 0.00E+00 | 0.00E+00 | 0.00E+00 | 4.64E-09 | 2.37E-07 |
| 131 | 0.00E+00 | 0.00E+00 | 0.00E+00 | 0.00E+00 | 0.00E+00 | 0.00E+00 | 0.00E+00 | 0.00E+00 | 2.22E-07 |
| 132 | 0.00E+00 | 0.00E+00 | 0.00E+00 | 0.00E+00 | 0.00E+00 | 0.00E+00 | 0.00E+00 | 0.00E+00 | 2.07E-07 |
| 133 | 0.00E+00 | 0.00E+00 | 0.00E+00 | 0.00E+00 | 0.00E+00 | 0.00E+00 | 0.00E+00 | 0.00E+00 | 1.94E-07 |
| 134 | 0.00E+00 | 0.00E+00 | 0.00E+00 | 0.00E+00 | 0.00E+00 | 0.00E+00 | 0.00E+00 | 0.00E+00 | 1.82E-07 |
| 135 | 0.00E+00 | 0.00E+00 | 0.00E+00 | 0.00E+00 | 0.00E+00 | 0.00E+00 | 0.00E+00 | 0.00E+00 | 1.70E-07 |
| 136 | 0.00E+00 | 0.00E+00 | 0.00E+00 | 0.00E+00 | 0.00E+00 | 0.00E+00 | 0.00E+00 | 0.00E+00 | 1.58E-07 |
| 137 | 0.00E+00 | 0.00E+00 | 0.00E+00 | 0.00E+00 | 0.00E+00 | 0.00E+00 | 0.00E+00 | 0.00E+00 | 1.46E-07 |
| 138 | 0.00E+00 | 0.00E+00 | 0.00E+00 | 0.00E+00 | 0.00E+00 | 0.00E+00 | 0.00E+00 | 0.00E+00 | 1.34E-07 |
| 139 | 0.00E+00 | 0.00E+00 | 0.00E+00 | 0.00E+00 | 0.00E+00 | 0.00E+00 | 0.00E+00 | 0.00E+00 | 1.22E-07 |
| 140 | 0.00E+00 | 0.00E+00 | 0.00E+00 | 0.00E+00 | 0.00E+00 | 0.00E+00 | 0.00E+00 | 0.00E+00 | 1.11E-07 |
| 141 | 0.00E+00 | 0.00E+00 | 0.00E+00 | 0.00E+00 | 0.00E+00 | 0.00E+00 | 0.00E+00 | 0.00E+00 | 9.92E-08 |
| 142 | 0.00E+00 | 0.00E+00 | 0.00E+00 | 0.00E+00 | 0.00E+00 | 0.00E+00 | 0.00E+00 | 0.00E+00 | 8.86E-08 |
| 143 | 0.00E+00 | 0.00E+00 | 0.00E+00 | 0.00E+00 | 0.00E+00 | 0.00E+00 | 0.00E+00 | 0.00E+00 | 7.84E-08 |
| 144 | 0.00E+00 | 0.00E+00 | 0.00E+00 | 0.00E+00 | 0.00E+00 | 0.00E+00 | 0.00E+00 | 0.00E+00 | 6.86E-08 |
| 145 | 0.00E+00 | 0.00E+00 | 0.00E+00 | 0.00E+00 | 0.00E+00 | 0.00E+00 | 0.00E+00 | 0.00E+00 | 5.87E-08 |
| 146 | 0.00E+00 | 0.00E+00 | 0.00E+00 | 0.00E+00 | 0.00E+00 | 0.00E+00 | 0.00E+00 | 0.00E+00 | 4.87E-08 |
| 147 | 0.00E+00 | 0.00E+00 | 0.00E+00 | 0.00E+00 | 0.00E+00 | 0.00E+00 | 0.00E+00 | 0.00E+00 | 3.83E-08 |
| 148 | 0.00E+00 | 0.00E+00 | 0.00E+00 | 0.00E+00 | 0.00E+00 | 0.00E+00 | 0.00E+00 | 0.00E+00 | 2.72E-08 |
| 149 | 0.00E+00 | 0.00E+00 | 0.00E+00 | 0.00E+00 | 0.00E+00 | 0.00E+00 | 0.00E+00 | 0.00E+00 | 1.60E-08 |
| 150 | 0.00E+00 | 0.00E+00 | 0.00E+00 | 0.00E+00 | 0.00E+00 | 0.00E+00 | 0.00E+00 | 0.00E+00 | 3.79E-09 |

Table.8. Spectra obtained by MCNP code: Al- 0.8mm

| energy bin (kev)/voltage(kv) | 20kv | 30kv | 40kv | 50kv | 60kv | 80kv | 100kv | 130kv | 150kv |
| --- | --- | --- | --- | --- | --- | --- | --- | --- | --- |
| 1 | 0.00E+00 | 0.00E+00 | 0.00E+00 | 0.00E+00 | 0.00E+00 | 0.00E+00 | 0.00E+00 | 0.00E+00 | 0.00E+00 |
| 2 | ######## | ######## | ######## | ######## | ######## | ######## | ######## | ######## | ######## |
| 3 | 5.41E-58 | 7.56E-59 | 6.50E-59 | 6.39E-59 | 6.06E-59 | 5.83E-59 | 6.06E-59 | 8.03E-59 | 8.99E-59 |
| 4 | 6.95E-30 | 4.38E-30 | 4.08E-30 | 3.67E-30 | 3.38E-30 | 3.14E-30 | 3.00E-30 | 3.55E-30 | 3.10E-30 |
| 5 | 6.73E-19 | 9.05E-19 | 8.85E-19 | 8.58E-19 | 7.91E-19 | 7.08E-19 | 6.69E-19 | 7.36E-19 | 6.18E-19 |
| 6 | 9.29E-14 | 1.47E-13 | 1.60E-13 | 1.60E-13 | 1.57E-13 | 1.44E-13 | 1.31E-13 | 1.33E-13 | 1.15E-13 |
| 7 | 1.32E-11 | 2.06E-11 | 2.36E-11 | 2.52E-11 | 2.56E-11 | 2.42E-11 | 2.32E-11 | 2.41E-11 | 1.98E-11 |
| 8 | 1.67E-09 | 2.60E-09 | 3.15E-09 | 3.54E-09 | 3.72E-09 | 3.82E-09 | 3.68E-09 | 3.86E-09 | 3.33E-09 |
| 9 | 9.26E-09 | 1.90E-08 | 2.61E-08 | 3.22E-08 | 3.66E-08 | 4.28E-08 | 4.63E-08 | 5.39E-08 | 4.77E-08 |
| 10 | 4.97E-08 | 8.32E-08 | 1.09E-07 | 1.33E-07 | 1.50E-07 | 1.71E-07 | 1.83E-07 | 2.07E-07 | 1.83E-07 |
| 11 | 7.34E-08 | 1.11E-07 | 1.43E-07 | 1.64E-07 | 1.78E-07 | 1.89E-07 | 1.89E-07 | 2.07E-07 | 1.77E-07 |
| 12 | 1.03E-07 | 1.71E-07 | 2.15E-07 | 2.48E-07 | 2.63E-07 | 2.72E-07 | 2.73E-07 | 2.89E-07 | 2.39E-07 |
| 13 | 1.45E-07 | 2.50E-07 | 3.18E-07 | 3.61E-07 | 3.88E-07 | 3.84E-07 | 3.84E-07 | 3.93E-07 | 3.35E-07 |
| 14 | 2.10E-07 | 3.97E-07 | 5.21E-07 | 6.04E-07 | 6.72E-07 | 7.11E-07 | 7.26E-07 | 7.49E-07 | 6.38E-07 |
| 15 | 2.62E-07 | 5.63E-07 | 7.69E-07 | 9.25E-07 | 1.03E-06 | 1.13E-06 | 1.18E-06 | 1.27E-06 | 1.10E-06 |
| 16 | 2.48E-07 | 6.19E-07 | 8.77E-07 | 1.08E-06 | 1.23E-06 | 1.40E-06 | 1.49E-06 | 1.64E-06 | 1.46E-06 |
| 17 | 2.34E-07 | 6.87E-07 | 1.00E-06 | 1.27E-06 | 1.44E-06 | 1.69E-06 | 1.83E-06 | 2.06E-06 | 1.85E-06 |
| 18 | 1.87E-07 | 7.11E-07 | 1.08E-06 | 1.39E-06 | 1.60E-06 | 1.93E-06 | 2.13E-06 | 2.43E-06 | 2.22E-06 |
| 19 | 1.37E-07 | 7.17E-07 | 1.13E-06 | 1.48E-06 | 1.74E-06 | 2.14E-06 | 2.40E-06 | 2.78E-06 | 2.57E-06 |
| 20 | 6.19E-08 | 7.04E-07 | 1.15E-06 | 1.54E-06 | 1.83E-06 | 2.30E-06 | 2.63E-06 | 3.09E-06 | 2.90E-06 |
| 21 | 0.00E+00 | 6.75E-07 | 1.15E-06 | 1.57E-06 | 1.89E-06 | 2.42E-06 | 2.81E-06 | 3.34E-06 | 3.18E-06 |
| 22 | 0.00E+00 | 6.28E-07 | 1.12E-06 | 1.56E-06 | 1.92E-06 | 2.49E-06 | 2.94E-06 | 3.53E-06 | 3.41E-06 |
| 23 | 0.00E+00 | 5.67E-07 | 1.07E-06 | 1.52E-06 | 1.90E-06 | 2.50E-06 | 3.00E-06 | 3.64E-06 | 3.58E-06 |
| 24 | 0.00E+00 | 5.04E-07 | 1.02E-06 | 1.48E-06 | 1.88E-06 | 2.51E-06 | 3.06E-06 | 3.74E-06 | 3.73E-06 |
| 25 | 0.00E+00 | 4.39E-07 | 9.68E-07 | 1.43E-06 | 1.85E-06 | 2.52E-06 | 3.10E-06 | 3.83E-06 | 3.87E-06 |
| 26 | 0.00E+00 | 3.72E-07 | 9.17E-07 | 1.39E-06 | 1.82E-06 | 2.52E-06 | 3.13E-06 | 3.91E-06 | 4.00E-06 |
| 27 | 0.00E+00 | 3.01E-07 | 8.67E-07 | 1.35E-06 | 1.79E-06 | 2.52E-06 | 3.15E-06 | 3.98E-06 | 4.11E-06 |
| 28 | 0.00E+00 | 2.24E-07 | 8.17E-07 | 1.30E-06 | 1.75E-06 | 2.51E-06 | 3.17E-06 | 4.04E-06 | 4.22E-06 |
| 29 | 0.00E+00 | 1.45E-07 | 7.62E-07 | 1.25E-06 | 1.70E-06 | 2.49E-06 | 3.17E-06 | 4.07E-06 | 4.29E-06 |
| 30 | 0.00E+00 | 5.47E-08 | 7.02E-07 | 1.20E-06 | 1.65E-06 | 2.45E-06 | 3.15E-06 | 4.05E-06 | 4.34E-06 |
| 31 | 0.00E+00 | 0.00E+00 | 6.40E-07 | 1.14E-06 | 1.58E-06 | 2.40E-06 | 3.12E-06 | 4.01E-06 | 4.36E-06 |
| 32 | 0.00E+00 | 0.00E+00 | 5.73E-07 | 1.08E-06 | 1.51E-06 | 2.32E-06 | 3.06E-06 | 3.94E-06 | 4.35E-06 |
| 33 | 0.00E+00 | 0.00E+00 | 5.05E-07 | 1.01E-06 | 1.45E-06 | 2.24E-06 | 2.99E-06 | 3.86E-06 | 4.32E-06 |
| 34 | 0.00E+00 | 0.00E+00 | 4.38E-07 | 9.45E-07 | 1.38E-06 | 2.15E-06 | 2.91E-06 | 3.77E-06 | 4.27E-06 |
| 35 | 0.00E+00 | 0.00E+00 | 3.71E-07 | 8.81E-07 | 1.31E-06 | 2.07E-06 | 2.83E-06 | 3.70E-06 | 4.21E-06 |
| 36 | 0.00E+00 | 0.00E+00 | 3.05E-07 | 8.19E-07 | 1.25E-06 | 1.98E-06 | 2.74E-06 | 3.62E-06 | 4.15E-06 |
| 37 | 0.00E+00 | 0.00E+00 | 2.38E-07 | 7.56E-07 | 1.18E-06 | 1.91E-06 | 2.66E-06 | 3.55E-06 | 4.08E-06 |
| 38 | 0.00E+00 | 0.00E+00 | 1.73E-07 | 6.97E-07 | 1.12E-06 | 1.83E-06 | 2.58E-06 | 3.49E-06 | 4.00E-06 |
| 39 | 0.00E+00 | 0.00E+00 | 1.08E-07 | 6.35E-07 | 1.06E-06 | 1.77E-06 | 2.49E-06 | 3.41E-06 | 3.92E-06 |
| 40 | 0.00E+00 | 0.00E+00 | 4.74E-08 | 5.74E-07 | 9.91E-07 | 1.71E-06 | 2.41E-06 | 3.34E-06 | 3.84E-06 |
| 41 | 0.00E+00 | 0.00E+00 | 0.00E+00 | 5.16E-07 | 9.31E-07 | 1.65E-06 | 2.33E-06 | 3.25E-06 | 3.76E-06 |
| 42 | 0.00E+00 | 0.00E+00 | 0.00E+00 | 4.59E-07 | 8.75E-07 | 1.59E-06 | 2.26E-06 | 3.17E-06 | 3.67E-06 |
| 43 | 0.00E+00 | 0.00E+00 | 0.00E+00 | 4.02E-07 | 8.21E-07 | 1.53E-06 | 2.18E-06 | 3.08E-06 | 3.59E-06 |
| 44 | 0.00E+00 | 0.00E+00 | 0.00E+00 | 3.49E-07 | 7.66E-07 | 1.47E-06 | 2.11E-06 | 2.99E-06 | 3.50E-06 |
| 45 | 0.00E+00 | 0.00E+00 | 0.00E+00 | 2.96E-07 | 7.13E-07 | 1.40E-06 | 2.05E-06 | 2.91E-06 | 3.42E-06 |
| 46 | 0.00E+00 | 0.00E+00 | 0.00E+00 | 2.44E-07 | 6.60E-07 | 1.34E-06 | 1.98E-06 | 2.83E-06 | 3.34E-06 |
| 47 | 0.00E+00 | 0.00E+00 | 0.00E+00 | 1.93E-07 | 6.10E-07 | 1.28E-06 | 1.92E-06 | 2.75E-06 | 3.26E-06 |
| 48 | 0.00E+00 | 0.00E+00 | 0.00E+00 | 1.42E-07 | 5.61E-07 | 1.23E-06 | 1.85E-06 | 2.67E-06 | 3.19E-06 |
| 49 | 0.00E+00 | 0.00E+00 | 0.00E+00 | 9.03E-08 | 5.14E-07 | 1.17E-06 | 1.78E-06 | 2.59E-06 | 3.11E-06 |
| 50 | 0.00E+00 | 0.00E+00 | 0.00E+00 | 3.75E-08 | 4.68E-07 | 1.12E-06 | 1.72E-06 | 2.51E-06 | 3.03E-06 |
| 51 | 0.00E+00 | 0.00E+00 | 0.00E+00 | 0.00E+00 | 4.22E-07 | 1.07E-06 | 1.66E-06 | 2.44E-06 | 2.96E-06 |
| 52 | 0.00E+00 | 0.00E+00 | 0.00E+00 | 0.00E+00 | 3.76E-07 | 1.01E-06 | 1.60E-06 | 2.38E-06 | 2.89E-06 |
| 53 | 0.00E+00 | 0.00E+00 | 0.00E+00 | 0.00E+00 | 3.30E-07 | 9.57E-07 | 1.55E-06 | 2.33E-06 | 2.82E-06 |
| 54 | 0.00E+00 | 0.00E+00 | 0.00E+00 | 0.00E+00 | 2.85E-07 | 9.07E-07 | 1.51E-06 | 2.27E-06 | 2.75E-06 |
| 55 | 0.00E+00 | 0.00E+00 | 0.00E+00 | 0.00E+00 | 2.41E-07 | 8.59E-07 | 1.46E-06 | 2.21E-06 | 2.70E-06 |
| 56 | 0.00E+00 | 0.00E+00 | 0.00E+00 | 0.00E+00 | 1.97E-07 | 8.17E-07 | 1.40E-06 | 2.15E-06 | 2.63E-06 |
| 57 | 0.00E+00 | 0.00E+00 | 0.00E+00 | 0.00E+00 | 1.54E-07 | 7.76E-07 | 1.35E-06 | 2.08E-06 | 2.58E-06 |
| 58 | 0.00E+00 | 0.00E+00 | 0.00E+00 | 0.00E+00 | 1.10E-07 | 7.40E-07 | 1.26E-06 | 1.99E-06 | 2.51E-06 |
| 59 | 0.00E+00 | 0.00E+00 | 0.00E+00 | 0.00E+00 | 6.96E-08 | 8.49E-07 | 2.24E-06 | 4.84E-06 | 6.55E-06 |
| 60 | 0.00E+00 | 0.00E+00 | 0.00E+00 | 0.00E+00 | 2.69E-08 | 9.43E-07 | 2.97E-06 | 7.12E-06 | 9.88E-06 |
| 61 | 0.00E+00 | 0.00E+00 | 0.00E+00 | 0.00E+00 | 0.00E+00 | 6.11E-07 | 1.09E-06 | 1.86E-06 | 2.38E-06 |
| 62 | 0.00E+00 | 0.00E+00 | 0.00E+00 | 0.00E+00 | 0.00E+00 | 5.96E-07 | 1.19E-06 | 1.94E-06 | 2.57E-06 |
| 63 | 0.00E+00 | 0.00E+00 | 0.00E+00 | 0.00E+00 | 0.00E+00 | 5.94E-07 | 1.04E-06 | 1.62E-06 | 2.10E-06 |
| 64 | 0.00E+00 | 0.00E+00 | 0.00E+00 | 0.00E+00 | 0.00E+00 | 5.15E-07 | 1.02E-06 | 1.55E-06 | 2.04E-06 |
| 65 | 0.00E+00 | 0.00E+00 | 0.00E+00 | 0.00E+00 | 0.00E+00 | 4.80E-07 | 9.76E-07 | 1.55E-06 | 2.02E-06 |
| 66 | 0.00E+00 | 0.00E+00 | 0.00E+00 | 0.00E+00 | 0.00E+00 | 4.51E-07 | 9.18E-07 | 1.53E-06 | 1.99E-06 |
| 67 | 0.00E+00 | 0.00E+00 | 0.00E+00 | 0.00E+00 | 0.00E+00 | 4.29E-07 | 8.47E-07 | 1.47E-06 | 1.95E-06 |
| 68 | 0.00E+00 | 0.00E+00 | 0.00E+00 | 0.00E+00 | 0.00E+00 | 4.86E-07 | 1.51E-06 | 3.25E-06 | 4.61E-06 |
| 69 | 0.00E+00 | 0.00E+00 | 0.00E+00 | 0.00E+00 | 0.00E+00 | 3.40E-07 | 8.04E-07 | 1.44E-06 | 1.77E-06 |
| 70 | 0.00E+00 | 0.00E+00 | 0.00E+00 | 0.00E+00 | 0.00E+00 | 3.29E-07 | 9.45E-07 | 1.84E-06 | 2.46E-06 |
| 71 | 0.00E+00 | 0.00E+00 | 0.00E+00 | 0.00E+00 | 0.00E+00 | 2.78E-07 | 7.46E-07 | 1.25E-06 | 1.47E-06 |
| 72 | 0.00E+00 | 0.00E+00 | 0.00E+00 | 0.00E+00 | 0.00E+00 | 2.49E-07 | 6.64E-07 | 1.22E-06 | 1.48E-06 |
| 73 | 0.00E+00 | 0.00E+00 | 0.00E+00 | 0.00E+00 | 0.00E+00 | 2.16E-07 | 6.31E-07 | 1.17E-06 | 1.44E-06 |
| 74 | 0.00E+00 | 0.00E+00 | 0.00E+00 | 0.00E+00 | 0.00E+00 | 1.86E-07 | 6.02E-07 | 1.14E-06 | 1.40E-06 |
| 75 | 0.00E+00 | 0.00E+00 | 0.00E+00 | 0.00E+00 | 0.00E+00 | 1.56E-07 | 5.75E-07 | 1.10E-06 | 1.37E-06 |
| 76 | 0.00E+00 | 0.00E+00 | 0.00E+00 | 0.00E+00 | 0.00E+00 | 1.27E-07 | 5.51E-07 | 1.08E-06 | 1.33E-06 |
| 77 | 0.00E+00 | 0.00E+00 | 0.00E+00 | 0.00E+00 | 0.00E+00 | 9.94E-08 | 5.29E-07 | 1.04E-06 | 1.30E-06 |
| 78 | 0.00E+00 | 0.00E+00 | 0.00E+00 | 0.00E+00 | 0.00E+00 | 7.16E-08 | 5.08E-07 | 1.01E-06 | 1.28E-06 |
| 79 | 0.00E+00 | 0.00E+00 | 0.00E+00 | 0.00E+00 | 0.00E+00 | 4.35E-08 | 4.87E-07 | 9.79E-07 | 1.25E-06 |
| 80 | 0.00E+00 | 0.00E+00 | 0.00E+00 | 0.00E+00 | 0.00E+00 | 1.50E-08 | 4.64E-07 | 9.43E-07 | 1.22E-06 |
| 81 | 0.00E+00 | 0.00E+00 | 0.00E+00 | 0.00E+00 | 0.00E+00 | 0.00E+00 | 4.39E-07 | 9.07E-07 | 1.20E-06 |
| 82 | 0.00E+00 | 0.00E+00 | 0.00E+00 | 0.00E+00 | 0.00E+00 | 0.00E+00 | 4.14E-07 | 8.76E-07 | 1.17E-06 |
| 83 | 0.00E+00 | 0.00E+00 | 0.00E+00 | 0.00E+00 | 0.00E+00 | 0.00E+00 | 3.88E-07 | 8.46E-07 | 1.15E-06 |
| 84 | 0.00E+00 | 0.00E+00 | 0.00E+00 | 0.00E+00 | 0.00E+00 | 0.00E+00 | 3.63E-07 | 8.15E-07 | 1.12E-06 |
| 85 | 0.00E+00 | 0.00E+00 | 0.00E+00 | 0.00E+00 | 0.00E+00 | 0.00E+00 | 3.41E-07 | 7.90E-07 | 1.09E-06 |
| 86 | 0.00E+00 | 0.00E+00 | 0.00E+00 | 0.00E+00 | 0.00E+00 | 0.00E+00 | 3.17E-07 | 7.68E-07 | 1.07E-06 |
| 87 | 0.00E+00 | 0.00E+00 | 0.00E+00 | 0.00E+00 | 0.00E+00 | 0.00E+00 | 2.93E-07 | 7.47E-07 | 1.04E-06 |
| 88 | 0.00E+00 | 0.00E+00 | 0.00E+00 | 0.00E+00 | 0.00E+00 | 0.00E+00 | 2.68E-07 | 7.27E-07 | 1.02E-06 |
| 89 | 0.00E+00 | 0.00E+00 | 0.00E+00 | 0.00E+00 | 0.00E+00 | 0.00E+00 | 2.43E-07 | 7.07E-07 | 9.91E-07 |
| 90 | 0.00E+00 | 0.00E+00 | 0.00E+00 | 0.00E+00 | 0.00E+00 | 0.00E+00 | 2.18E-07 | 6.87E-07 | 9.68E-07 |
| 91 | 0.00E+00 | 0.00E+00 | 0.00E+00 | 0.00E+00 | 0.00E+00 | 0.00E+00 | 1.96E-07 | 6.66E-07 | 9.46E-07 |
| 92 | 0.00E+00 | 0.00E+00 | 0.00E+00 | 0.00E+00 | 0.00E+00 | 0.00E+00 | 1.75E-07 | 6.45E-07 | 9.21E-07 |
| 93 | 0.00E+00 | 0.00E+00 | 0.00E+00 | 0.00E+00 | 0.00E+00 | 0.00E+00 | 1.56E-07 | 6.25E-07 | 8.96E-07 |
| 94 | 0.00E+00 | 0.00E+00 | 0.00E+00 | 0.00E+00 | 0.00E+00 | 0.00E+00 | 1.37E-07 | 6.03E-07 | 8.69E-07 |
| 95 | 0.00E+00 | 0.00E+00 | 0.00E+00 | 0.00E+00 | 0.00E+00 | 0.00E+00 | 1.16E-07 | 5.80E-07 | 8.41E-07 |
| 96 | 0.00E+00 | 0.00E+00 | 0.00E+00 | 0.00E+00 | 0.00E+00 | 0.00E+00 | 9.54E-08 | 5.55E-07 | 8.12E-07 |
| 97 | 0.00E+00 | 0.00E+00 | 0.00E+00 | 0.00E+00 | 0.00E+00 | 0.00E+00 | 7.39E-08 | 5.32E-07 | 7.87E-07 |
| 98 | 0.00E+00 | 0.00E+00 | 0.00E+00 | 0.00E+00 | 0.00E+00 | 0.00E+00 | 5.24E-08 | 5.08E-07 | 7.63E-07 |
| 99 | 0.00E+00 | 0.00E+00 | 0.00E+00 | 0.00E+00 | 0.00E+00 | 0.00E+00 | 3.08E-08 | 4.87E-07 | 7.42E-07 |
| 100 | 0.00E+00 | 0.00E+00 | 0.00E+00 | 0.00E+00 | 0.00E+00 | 0.00E+00 | 1.22E-08 | 4.70E-07 | 7.25E-07 |
| 101 | 0.00E+00 | 0.00E+00 | 0.00E+00 | 0.00E+00 | 0.00E+00 | 0.00E+00 | 0.00E+00 | 4.55E-07 | 7.08E-07 |
| 102 | 0.00E+00 | 0.00E+00 | 0.00E+00 | 0.00E+00 | 0.00E+00 | 0.00E+00 | 0.00E+00 | 4.41E-07 | 6.91E-07 |
| 103 | 0.00E+00 | 0.00E+00 | 0.00E+00 | 0.00E+00 | 0.00E+00 | 0.00E+00 | 0.00E+00 | 4.23E-07 | 6.73E-07 |
| 104 | 0.00E+00 | 0.00E+00 | 0.00E+00 | 0.00E+00 | 0.00E+00 | 0.00E+00 | 0.00E+00 | 4.06E-07 | 6.54E-07 |
| 105 | 0.00E+00 | 0.00E+00 | 0.00E+00 | 0.00E+00 | 0.00E+00 | 0.00E+00 | 0.00E+00 | 3.86E-07 | 6.33E-07 |
| 106 | 0.00E+00 | 0.00E+00 | 0.00E+00 | 0.00E+00 | 0.00E+00 | 0.00E+00 | 0.00E+00 | 3.65E-07 | 6.14E-07 |
| 107 | 0.00E+00 | 0.00E+00 | 0.00E+00 | 0.00E+00 | 0.00E+00 | 0.00E+00 | 0.00E+00 | 3.45E-07 | 5.96E-07 |
| 108 | 0.00E+00 | 0.00E+00 | 0.00E+00 | 0.00E+00 | 0.00E+00 | 0.00E+00 | 0.00E+00 | 3.31E-07 | 5.80E-07 |
| 109 | 0.00E+00 | 0.00E+00 | 0.00E+00 | 0.00E+00 | 0.00E+00 | 0.00E+00 | 0.00E+00 | 3.16E-07 | 5.65E-07 |
| 110 | 0.00E+00 | 0.00E+00 | 0.00E+00 | 0.00E+00 | 0.00E+00 | 0.00E+00 | 0.00E+00 | 3.04E-07 | 5.51E-07 |
| 111 | 0.00E+00 | 0.00E+00 | 0.00E+00 | 0.00E+00 | 0.00E+00 | 0.00E+00 | 0.00E+00 | 2.90E-07 | 5.37E-07 |
| 112 | 0.00E+00 | 0.00E+00 | 0.00E+00 | 0.00E+00 | 0.00E+00 | 0.00E+00 | 0.00E+00 | 2.75E-07 | 5.22E-07 |
| 113 | 0.00E+00 | 0.00E+00 | 0.00E+00 | 0.00E+00 | 0.00E+00 | 0.00E+00 | 0.00E+00 | 2.54E-07 | 5.05E-07 |
| 114 | 0.00E+00 | 0.00E+00 | 0.00E+00 | 0.00E+00 | 0.00E+00 | 0.00E+00 | 0.00E+00 | 2.34E-07 | 4.85E-07 |
| 115 | 0.00E+00 | 0.00E+00 | 0.00E+00 | 0.00E+00 | 0.00E+00 | 0.00E+00 | 0.00E+00 | 2.12E-07 | 4.65E-07 |
| 116 | 0.00E+00 | 0.00E+00 | 0.00E+00 | 0.00E+00 | 0.00E+00 | 0.00E+00 | 0.00E+00 | 1.92E-07 | 4.44E-07 |
| 117 | 0.00E+00 | 0.00E+00 | 0.00E+00 | 0.00E+00 | 0.00E+00 | 0.00E+00 | 0.00E+00 | 1.75E-07 | 4.23E-07 |
| 118 | 0.00E+00 | 0.00E+00 | 0.00E+00 | 0.00E+00 | 0.00E+00 | 0.00E+00 | 0.00E+00 | 1.61E-07 | 4.05E-07 |
| 119 | 0.00E+00 | 0.00E+00 | 0.00E+00 | 0.00E+00 | 0.00E+00 | 0.00E+00 | 0.00E+00 | 1.48E-07 | 3.89E-07 |
| 120 | 0.00E+00 | 0.00E+00 | 0.00E+00 | 0.00E+00 | 0.00E+00 | 0.00E+00 | 0.00E+00 | 1.37E-07 | 3.74E-07 |
| 121 | 0.00E+00 | 0.00E+00 | 0.00E+00 | 0.00E+00 | 0.00E+00 | 0.00E+00 | 0.00E+00 | 1.25E-07 | 3.60E-07 |
| 122 | 0.00E+00 | 0.00E+00 | 0.00E+00 | 0.00E+00 | 0.00E+00 | 0.00E+00 | 0.00E+00 | 1.12E-07 | 3.47E-07 |
| 123 | 0.00E+00 | 0.00E+00 | 0.00E+00 | 0.00E+00 | 0.00E+00 | 0.00E+00 | 0.00E+00 | 9.82E-08 | 3.34E-07 |
| 124 | 0.00E+00 | 0.00E+00 | 0.00E+00 | 0.00E+00 | 0.00E+00 | 0.00E+00 | 0.00E+00 | 8.40E-08 | 3.21E-07 |
| 125 | 0.00E+00 | 0.00E+00 | 0.00E+00 | 0.00E+00 | 0.00E+00 | 0.00E+00 | 0.00E+00 | 7.00E-08 | 3.08E-07 |
| 126 | 0.00E+00 | 0.00E+00 | 0.00E+00 | 0.00E+00 | 0.00E+00 | 0.00E+00 | 0.00E+00 | 5.61E-08 | 2.95E-07 |
| 127 | 0.00E+00 | 0.00E+00 | 0.00E+00 | 0.00E+00 | 0.00E+00 | 0.00E+00 | 0.00E+00 | 4.33E-08 | 2.81E-07 |
| 128 | 0.00E+00 | 0.00E+00 | 0.00E+00 | 0.00E+00 | 0.00E+00 | 0.00E+00 | 0.00E+00 | 3.08E-08 | 2.65E-07 |
| 129 | 0.00E+00 | 0.00E+00 | 0.00E+00 | 0.00E+00 | 0.00E+00 | 0.00E+00 | 0.00E+00 | 1.82E-08 | 2.50E-07 |
| 130 | 0.00E+00 | 0.00E+00 | 0.00E+00 | 0.00E+00 | 0.00E+00 | 0.00E+00 | 0.00E+00 | 4.59E-09 | 2.34E-07 |
| 131 | 0.00E+00 | 0.00E+00 | 0.00E+00 | 0.00E+00 | 0.00E+00 | 0.00E+00 | 0.00E+00 | 0.00E+00 | 2.19E-07 |
| 132 | 0.00E+00 | 0.00E+00 | 0.00E+00 | 0.00E+00 | 0.00E+00 | 0.00E+00 | 0.00E+00 | 0.00E+00 | 2.05E-07 |
| 133 | 0.00E+00 | 0.00E+00 | 0.00E+00 | 0.00E+00 | 0.00E+00 | 0.00E+00 | 0.00E+00 | 0.00E+00 | 1.92E-07 |
| 134 | 0.00E+00 | 0.00E+00 | 0.00E+00 | 0.00E+00 | 0.00E+00 | 0.00E+00 | 0.00E+00 | 0.00E+00 | 1.80E-07 |
| 135 | 0.00E+00 | 0.00E+00 | 0.00E+00 | 0.00E+00 | 0.00E+00 | 0.00E+00 | 0.00E+00 | 0.00E+00 | 1.68E-07 |
| 136 | 0.00E+00 | 0.00E+00 | 0.00E+00 | 0.00E+00 | 0.00E+00 | 0.00E+00 | 0.00E+00 | 0.00E+00 | 1.57E-07 |
| 137 | 0.00E+00 | 0.00E+00 | 0.00E+00 | 0.00E+00 | 0.00E+00 | 0.00E+00 | 0.00E+00 | 0.00E+00 | 1.45E-07 |
| 138 | 0.00E+00 | 0.00E+00 | 0.00E+00 | 0.00E+00 | 0.00E+00 | 0.00E+00 | 0.00E+00 | 0.00E+00 | 1.33E-07 |
| 139 | 0.00E+00 | 0.00E+00 | 0.00E+00 | 0.00E+00 | 0.00E+00 | 0.00E+00 | 0.00E+00 | 0.00E+00 | 1.21E-07 |
| 140 | 0.00E+00 | 0.00E+00 | 0.00E+00 | 0.00E+00 | 0.00E+00 | 0.00E+00 | 0.00E+00 | 0.00E+00 | 1.09E-07 |
| 141 | 0.00E+00 | 0.00E+00 | 0.00E+00 | 0.00E+00 | 0.00E+00 | 0.00E+00 | 0.00E+00 | 0.00E+00 | 9.81E-08 |
| 142 | 0.00E+00 | 0.00E+00 | 0.00E+00 | 0.00E+00 | 0.00E+00 | 0.00E+00 | 0.00E+00 | 0.00E+00 | 8.76E-08 |
| 143 | 0.00E+00 | 0.00E+00 | 0.00E+00 | 0.00E+00 | 0.00E+00 | 0.00E+00 | 0.00E+00 | 0.00E+00 | 7.76E-08 |
| 144 | 0.00E+00 | 0.00E+00 | 0.00E+00 | 0.00E+00 | 0.00E+00 | 0.00E+00 | 0.00E+00 | 0.00E+00 | 6.79E-08 |
| 145 | 0.00E+00 | 0.00E+00 | 0.00E+00 | 0.00E+00 | 0.00E+00 | 0.00E+00 | 0.00E+00 | 0.00E+00 | 5.81E-08 |
| 146 | 0.00E+00 | 0.00E+00 | 0.00E+00 | 0.00E+00 | 0.00E+00 | 0.00E+00 | 0.00E+00 | 0.00E+00 | 4.82E-08 |
| 147 | 0.00E+00 | 0.00E+00 | 0.00E+00 | 0.00E+00 | 0.00E+00 | 0.00E+00 | 0.00E+00 | 0.00E+00 | 3.79E-08 |
| 148 | 0.00E+00 | 0.00E+00 | 0.00E+00 | 0.00E+00 | 0.00E+00 | 0.00E+00 | 0.00E+00 | 0.00E+00 | 2.70E-08 |
| 149 | 0.00E+00 | 0.00E+00 | 0.00E+00 | 0.00E+00 | 0.00E+00 | 0.00E+00 | 0.00E+00 | 0.00E+00 | 1.58E-08 |
| 150 | 0.00E+00 | 0.00E+00 | 0.00E+00 | 0.00E+00 | 0.00E+00 | 0.00E+00 | 0.00E+00 | 0.00E+00 | 3.75E-09 |

Table.9. Spectra obtained by MCNP code: Al- 1.2mm

| energy bin (kev)/voltage(kv) | 20kv | 30kv | 40kv | 50kv | 60kv | 80kv | 100kv | 130kv | 150kv |
| --- | --- | --- | --- | --- | --- | --- | --- | --- | --- |
| 1 | 0.00E+00 | 0.00E+00 | 0.00E+00 | 0.00E+00 | 0.00E+00 | 0.00E+00 | 0.00E+00 | 0.00E+00 | 0.00E+00 |
| 2 | ######## | ######## | ######## | ######## | ######## | ######## | ######## | ######## | ######## |
| 3 | 2.56E-83 | 3.58E-84 | 3.08E-84 | 3.03E-84 | 2.87E-84 | 2.76E-84 | 2.87E-84 | 3.80E-84 | 4.26E-84 |
| 4 | 1.74E-41 | 1.10E-41 | 1.02E-41 | 9.19E-42 | 8.46E-42 | 7.85E-42 | 7.52E-42 | 8.88E-42 | 7.77E-42 |
| 5 | 4.22E-25 | 5.67E-25 | 5.55E-25 | 5.38E-25 | 4.96E-25 | 4.44E-25 | 4.19E-25 | 4.61E-25 | 3.87E-25 |
| 6 | 1.87E-17 | 2.96E-17 | 3.22E-17 | 3.23E-17 | 3.16E-17 | 2.89E-17 | 2.64E-17 | 2.68E-17 | 2.31E-17 |
| 7 | 2.89E-14 | 4.50E-14 | 5.16E-14 | 5.50E-14 | 5.59E-14 | 5.29E-14 | 5.06E-14 | 5.26E-14 | 4.32E-14 |
| 8 | 4.04E-11 | 6.29E-11 | 7.61E-11 | 8.57E-11 | 9.01E-11 | 9.25E-11 | 8.89E-11 | 9.35E-11 | 8.06E-11 |
| 9 | 5.44E-10 | 1.11E-09 | 1.53E-09 | 1.89E-09 | 2.15E-09 | 2.52E-09 | 2.72E-09 | 3.17E-09 | 2.80E-09 |
| 10 | 7.15E-09 | 1.20E-08 | 1.57E-08 | 1.91E-08 | 2.16E-08 | 2.46E-08 | 2.64E-08 | 2.98E-08 | 2.63E-08 |
| 11 | 1.38E-08 | 2.09E-08 | 2.69E-08 | 3.08E-08 | 3.34E-08 | 3.54E-08 | 3.54E-08 | 3.89E-08 | 3.32E-08 |
| 12 | 2.54E-08 | 4.22E-08 | 5.32E-08 | 6.11E-08 | 6.49E-08 | 6.72E-08 | 6.75E-08 | 7.14E-08 | 5.90E-08 |
| 13 | 4.69E-08 | 8.07E-08 | 1.02E-07 | 1.16E-07 | 1.25E-07 | 1.24E-07 | 1.24E-07 | 1.27E-07 | 1.08E-07 |
| 14 | 8.91E-08 | 1.68E-07 | 2.21E-07 | 2.56E-07 | 2.85E-07 | 3.02E-07 | 3.08E-07 | 3.17E-07 | 2.70E-07 |
| 15 | 1.40E-07 | 3.04E-07 | 4.16E-07 | 5.02E-07 | 5.60E-07 | 6.17E-07 | 6.42E-07 | 6.95E-07 | 6.04E-07 |
| 16 | 1.43E-07 | 3.67E-07 | 5.22E-07 | 6.49E-07 | 7.35E-07 | 8.41E-07 | 8.98E-07 | 9.97E-07 | 8.85E-07 |
| 17 | 1.46E-07 | 4.40E-07 | 6.45E-07 | 8.17E-07 | 9.34E-07 | 1.10E-06 | 1.19E-06 | 1.34E-06 | 1.21E-06 |
| 18 | 1.22E-07 | 4.86E-07 | 7.39E-07 | 9.57E-07 | 1.11E-06 | 1.34E-06 | 1.48E-06 | 1.69E-06 | 1.55E-06 |
| 19 | 9.69E-08 | 5.17E-07 | 8.15E-07 | 1.07E-06 | 1.26E-06 | 1.56E-06 | 1.75E-06 | 2.04E-06 | 1.89E-06 |
| 20 | 4.80E-08 | 5.30E-07 | 8.66E-07 | 1.16E-06 | 1.39E-06 | 1.75E-06 | 2.00E-06 | 2.35E-06 | 2.21E-06 |
| 21 | 0.00E+00 | 5.26E-07 | 8.95E-07 | 1.22E-06 | 1.48E-06 | 1.90E-06 | 2.20E-06 | 2.62E-06 | 2.50E-06 |
| 22 | 0.00E+00 | 5.02E-07 | 8.98E-07 | 1.25E-06 | 1.54E-06 | 2.00E-06 | 2.36E-06 | 2.84E-06 | 2.74E-06 |
| 23 | 0.00E+00 | 4.61E-07 | 8.71E-07 | 1.24E-06 | 1.55E-06 | 2.04E-06 | 2.45E-06 | 2.98E-06 | 2.92E-06 |
| 24 | 0.00E+00 | 4.17E-07 | 8.43E-07 | 1.23E-06 | 1.56E-06 | 2.09E-06 | 2.54E-06 | 3.11E-06 | 3.10E-06 |
| 25 | 0.00E+00 | 3.69E-07 | 8.16E-07 | 1.21E-06 | 1.56E-06 | 2.13E-06 | 2.62E-06 | 3.24E-06 | 3.27E-06 |
| 26 | 0.00E+00 | 3.18E-07 | 7.86E-07 | 1.19E-06 | 1.56E-06 | 2.17E-06 | 2.69E-06 | 3.36E-06 | 3.44E-06 |
| 27 | 0.00E+00 | 2.61E-07 | 7.56E-07 | 1.18E-06 | 1.56E-06 | 2.20E-06 | 2.75E-06 | 3.48E-06 | 3.60E-06 |
| 28 | 0.00E+00 | 1.97E-07 | 7.25E-07 | 1.16E-06 | 1.55E-06 | 2.23E-06 | 2.82E-06 | 3.59E-06 | 3.75E-06 |
| 29 | 0.00E+00 | 1.30E-07 | 6.86E-07 | 1.13E-06 | 1.53E-06 | 2.25E-06 | 2.86E-06 | 3.67E-06 | 3.88E-06 |
| 30 | 0.00E+00 | 5.03E-08 | 6.40E-07 | 1.09E-06 | 1.50E-06 | 2.24E-06 | 2.88E-06 | 3.70E-06 | 3.96E-06 |
| 31 | 0.00E+00 | 0.00E+00 | 5.89E-07 | 1.05E-06 | 1.46E-06 | 2.21E-06 | 2.87E-06 | 3.69E-06 | 4.02E-06 |
| 32 | 0.00E+00 | 0.00E+00 | 5.31E-07 | 9.97E-07 | 1.40E-06 | 2.16E-06 | 2.84E-06 | 3.66E-06 | 4.04E-06 |
| 33 | 0.00E+00 | 0.00E+00 | 4.70E-07 | 9.40E-07 | 1.35E-06 | 2.09E-06 | 2.78E-06 | 3.59E-06 | 4.02E-06 |
| 34 | 0.00E+00 | 0.00E+00 | 4.10E-07 | 8.83E-07 | 1.29E-06 | 2.01E-06 | 2.72E-06 | 3.53E-06 | 3.99E-06 |
| 35 | 0.00E+00 | 0.00E+00 | 3.48E-07 | 8.27E-07 | 1.23E-06 | 1.94E-06 | 2.65E-06 | 3.47E-06 | 3.96E-06 |
| 36 | 0.00E+00 | 0.00E+00 | 2.87E-07 | 7.72E-07 | 1.18E-06 | 1.87E-06 | 2.58E-06 | 3.42E-06 | 3.91E-06 |
| 37 | 0.00E+00 | 0.00E+00 | 2.25E-07 | 7.16E-07 | 1.12E-06 | 1.80E-06 | 2.52E-06 | 3.36E-06 | 3.86E-06 |
| 38 | 0.00E+00 | 0.00E+00 | 1.64E-07 | 6.62E-07 | 1.06E-06 | 1.74E-06 | 2.45E-06 | 3.31E-06 | 3.80E-06 |
| 39 | 0.00E+00 | 0.00E+00 | 1.03E-07 | 6.05E-07 | 1.01E-06 | 1.69E-06 | 2.38E-06 | 3.26E-06 | 3.74E-06 |
| 40 | 0.00E+00 | 0.00E+00 | 4.55E-08 | 5.50E-07 | 9.49E-07 | 1.63E-06 | 2.31E-06 | 3.19E-06 | 3.67E-06 |
| 41 | 0.00E+00 | 0.00E+00 | 0.00E+00 | 4.95E-07 | 8.93E-07 | 1.58E-06 | 2.24E-06 | 3.12E-06 | 3.61E-06 |
| 42 | 0.00E+00 | 0.00E+00 | 0.00E+00 | 4.42E-07 | 8.41E-07 | 1.53E-06 | 2.17E-06 | 3.04E-06 | 3.53E-06 |
| 43 | 0.00E+00 | 0.00E+00 | 0.00E+00 | 3.87E-07 | 7.90E-07 | 1.47E-06 | 2.10E-06 | 2.96E-06 | 3.45E-06 |
| 44 | 0.00E+00 | 0.00E+00 | 0.00E+00 | 3.37E-07 | 7.39E-07 | 1.41E-06 | 2.04E-06 | 2.88E-06 | 3.38E-06 |
| 45 | 0.00E+00 | 0.00E+00 | 0.00E+00 | 2.86E-07 | 6.88E-07 | 1.36E-06 | 1.98E-06 | 2.81E-06 | 3.30E-06 |
| 46 | 0.00E+00 | 0.00E+00 | 0.00E+00 | 2.35E-07 | 6.38E-07 | 1.30E-06 | 1.92E-06 | 2.74E-06 | 3.23E-06 |
| 47 | 0.00E+00 | 0.00E+00 | 0.00E+00 | 1.87E-07 | 5.90E-07 | 1.24E-06 | 1.86E-06 | 2.66E-06 | 3.16E-06 |
| 48 | 0.00E+00 | 0.00E+00 | 0.00E+00 | 1.37E-07 | 5.44E-07 | 1.19E-06 | 1.80E-06 | 2.59E-06 | 3.09E-06 |
| 49 | 0.00E+00 | 0.00E+00 | 0.00E+00 | 8.77E-08 | 4.99E-07 | 1.14E-06 | 1.73E-06 | 2.52E-06 | 3.02E-06 |
| 50 | 0.00E+00 | 0.00E+00 | 0.00E+00 | 3.65E-08 | 4.55E-07 | 1.09E-06 | 1.67E-06 | 2.45E-06 | 2.95E-06 |
| 51 | 0.00E+00 | 0.00E+00 | 0.00E+00 | 0.00E+00 | 4.11E-07 | 1.04E-06 | 1.61E-06 | 2.38E-06 | 2.88E-06 |
| 52 | 0.00E+00 | 0.00E+00 | 0.00E+00 | 0.00E+00 | 3.66E-07 | 9.85E-07 | 1.56E-06 | 2.32E-06 | 2.81E-06 |
| 53 | 0.00E+00 | 0.00E+00 | 0.00E+00 | 0.00E+00 | 3.22E-07 | 9.33E-07 | 1.51E-06 | 2.27E-06 | 2.75E-06 |
| 54 | 0.00E+00 | 0.00E+00 | 0.00E+00 | 0.00E+00 | 2.78E-07 | 8.85E-07 | 1.47E-06 | 2.21E-06 | 2.69E-06 |
| 55 | 0.00E+00 | 0.00E+00 | 0.00E+00 | 0.00E+00 | 2.35E-07 | 8.38E-07 | 1.43E-06 | 2.16E-06 | 2.63E-06 |
| 56 | 0.00E+00 | 0.00E+00 | 0.00E+00 | 0.00E+00 | 1.92E-07 | 7.98E-07 | 1.37E-06 | 2.10E-06 | 2.57E-06 |
| 57 | 0.00E+00 | 0.00E+00 | 0.00E+00 | 0.00E+00 | 1.50E-07 | 7.59E-07 | 1.32E-06 | 2.03E-06 | 2.52E-06 |
| 58 | 0.00E+00 | 0.00E+00 | 0.00E+00 | 0.00E+00 | 1.08E-07 | 7.24E-07 | 1.24E-06 | 1.95E-06 | 2.45E-06 |
| 59 | 0.00E+00 | 0.00E+00 | 0.00E+00 | 0.00E+00 | 6.81E-08 | 8.31E-07 | 2.20E-06 | 4.74E-06 | 6.41E-06 |
| 60 | 0.00E+00 | 0.00E+00 | 0.00E+00 | 0.00E+00 | 2.63E-08 | 9.24E-07 | 2.91E-06 | 6.98E-06 | 9.68E-06 |
| 61 | 0.00E+00 | 0.00E+00 | 0.00E+00 | 0.00E+00 | 0.00E+00 | 5.98E-07 | 1.07E-06 | 1.83E-06 | 2.33E-06 |
| 62 | 0.00E+00 | 0.00E+00 | 0.00E+00 | 0.00E+00 | 0.00E+00 | 5.84E-07 | 1.17E-06 | 1.90E-06 | 2.51E-06 |
| 63 | 0.00E+00 | 0.00E+00 | 0.00E+00 | 0.00E+00 | 0.00E+00 | 5.83E-07 | 1.02E-06 | 1.59E-06 | 2.05E-06 |
| 64 | 0.00E+00 | 0.00E+00 | 0.00E+00 | 0.00E+00 | 0.00E+00 | 5.05E-07 | 1.00E-06 | 1.52E-06 | 2.00E-06 |
| 65 | 0.00E+00 | 0.00E+00 | 0.00E+00 | 0.00E+00 | 0.00E+00 | 4.70E-07 | 9.57E-07 | 1.52E-06 | 1.98E-06 |
| 66 | 0.00E+00 | 0.00E+00 | 0.00E+00 | 0.00E+00 | 0.00E+00 | 4.43E-07 | 9.01E-07 | 1.50E-06 | 1.95E-06 |
| 67 | 0.00E+00 | 0.00E+00 | 0.00E+00 | 0.00E+00 | 0.00E+00 | 4.21E-07 | 8.31E-07 | 1.45E-06 | 1.91E-06 |
| 68 | 0.00E+00 | 0.00E+00 | 0.00E+00 | 0.00E+00 | 0.00E+00 | 4.77E-07 | 1.49E-06 | 3.19E-06 | 4.52E-06 |
| 69 | 0.00E+00 | 0.00E+00 | 0.00E+00 | 0.00E+00 | 0.00E+00 | 3.33E-07 | 7.90E-07 | 1.41E-06 | 1.73E-06 |
| 70 | 0.00E+00 | 0.00E+00 | 0.00E+00 | 0.00E+00 | 0.00E+00 | 3.23E-07 | 9.28E-07 | 1.81E-06 | 2.42E-06 |
| 71 | 0.00E+00 | 0.00E+00 | 0.00E+00 | 0.00E+00 | 0.00E+00 | 2.73E-07 | 7.34E-07 | 1.22E-06 | 1.44E-06 |
| 72 | 0.00E+00 | 0.00E+00 | 0.00E+00 | 0.00E+00 | 0.00E+00 | 2.45E-07 | 6.53E-07 | 1.20E-06 | 1.45E-06 |
| 73 | 0.00E+00 | 0.00E+00 | 0.00E+00 | 0.00E+00 | 0.00E+00 | 2.12E-07 | 6.21E-07 | 1.15E-06 | 1.42E-06 |
| 74 | 0.00E+00 | 0.00E+00 | 0.00E+00 | 0.00E+00 | 0.00E+00 | 1.83E-07 | 5.92E-07 | 1.12E-06 | 1.38E-06 |
| 75 | 0.00E+00 | 0.00E+00 | 0.00E+00 | 0.00E+00 | 0.00E+00 | 1.53E-07 | 5.66E-07 | 1.09E-06 | 1.35E-06 |
| 76 | 0.00E+00 | 0.00E+00 | 0.00E+00 | 0.00E+00 | 0.00E+00 | 1.25E-07 | 5.42E-07 | 1.06E-06 | 1.31E-06 |
| 77 | 0.00E+00 | 0.00E+00 | 0.00E+00 | 0.00E+00 | 0.00E+00 | 9.78E-08 | 5.21E-07 | 1.03E-06 | 1.28E-06 |
| 78 | 0.00E+00 | 0.00E+00 | 0.00E+00 | 0.00E+00 | 0.00E+00 | 7.05E-08 | 5.01E-07 | 9.96E-07 | 1.26E-06 |
| 79 | 0.00E+00 | 0.00E+00 | 0.00E+00 | 0.00E+00 | 0.00E+00 | 4.28E-08 | 4.80E-07 | 9.65E-07 | 1.23E-06 |
| 80 | 0.00E+00 | 0.00E+00 | 0.00E+00 | 0.00E+00 | 0.00E+00 | 1.48E-08 | 4.57E-07 | 9.29E-07 | 1.20E-06 |
| 81 | 0.00E+00 | 0.00E+00 | 0.00E+00 | 0.00E+00 | 0.00E+00 | 0.00E+00 | 4.32E-07 | 8.94E-07 | 1.18E-06 |
| 82 | 0.00E+00 | 0.00E+00 | 0.00E+00 | 0.00E+00 | 0.00E+00 | 0.00E+00 | 4.08E-07 | 8.63E-07 | 1.15E-06 |
| 83 | 0.00E+00 | 0.00E+00 | 0.00E+00 | 0.00E+00 | 0.00E+00 | 0.00E+00 | 3.83E-07 | 8.33E-07 | 1.13E-06 |
| 84 | 0.00E+00 | 0.00E+00 | 0.00E+00 | 0.00E+00 | 0.00E+00 | 0.00E+00 | 3.58E-07 | 8.03E-07 | 1.10E-06 |
| 85 | 0.00E+00 | 0.00E+00 | 0.00E+00 | 0.00E+00 | 0.00E+00 | 0.00E+00 | 3.36E-07 | 7.79E-07 | 1.08E-06 |
| 86 | 0.00E+00 | 0.00E+00 | 0.00E+00 | 0.00E+00 | 0.00E+00 | 0.00E+00 | 3.13E-07 | 7.57E-07 | 1.05E-06 |
| 87 | 0.00E+00 | 0.00E+00 | 0.00E+00 | 0.00E+00 | 0.00E+00 | 0.00E+00 | 2.89E-07 | 7.36E-07 | 1.03E-06 |
| 88 | 0.00E+00 | 0.00E+00 | 0.00E+00 | 0.00E+00 | 0.00E+00 | 0.00E+00 | 2.64E-07 | 7.17E-07 | 1.00E-06 |
| 89 | 0.00E+00 | 0.00E+00 | 0.00E+00 | 0.00E+00 | 0.00E+00 | 0.00E+00 | 2.40E-07 | 6.97E-07 | 9.78E-07 |
| 90 | 0.00E+00 | 0.00E+00 | 0.00E+00 | 0.00E+00 | 0.00E+00 | 0.00E+00 | 2.15E-07 | 6.78E-07 | 9.55E-07 |
| 91 | 0.00E+00 | 0.00E+00 | 0.00E+00 | 0.00E+00 | 0.00E+00 | 0.00E+00 | 1.93E-07 | 6.57E-07 | 9.33E-07 |
| 92 | 0.00E+00 | 0.00E+00 | 0.00E+00 | 0.00E+00 | 0.00E+00 | 0.00E+00 | 1.72E-07 | 6.36E-07 | 9.09E-07 |
| 93 | 0.00E+00 | 0.00E+00 | 0.00E+00 | 0.00E+00 | 0.00E+00 | 0.00E+00 | 1.54E-07 | 6.16E-07 | 8.84E-07 |
| 94 | 0.00E+00 | 0.00E+00 | 0.00E+00 | 0.00E+00 | 0.00E+00 | 0.00E+00 | 1.35E-07 | 5.95E-07 | 8.58E-07 |
| 95 | 0.00E+00 | 0.00E+00 | 0.00E+00 | 0.00E+00 | 0.00E+00 | 0.00E+00 | 1.15E-07 | 5.72E-07 | 8.30E-07 |
| 96 | 0.00E+00 | 0.00E+00 | 0.00E+00 | 0.00E+00 | 0.00E+00 | 0.00E+00 | 9.42E-08 | 5.48E-07 | 8.02E-07 |
| 97 | 0.00E+00 | 0.00E+00 | 0.00E+00 | 0.00E+00 | 0.00E+00 | 0.00E+00 | 7.30E-08 | 5.25E-07 | 7.77E-07 |
| 98 | 0.00E+00 | 0.00E+00 | 0.00E+00 | 0.00E+00 | 0.00E+00 | 0.00E+00 | 5.17E-08 | 5.02E-07 | 7.53E-07 |
| 99 | 0.00E+00 | 0.00E+00 | 0.00E+00 | 0.00E+00 | 0.00E+00 | 0.00E+00 | 3.04E-08 | 4.81E-07 | 7.32E-07 |
| 100 | 0.00E+00 | 0.00E+00 | 0.00E+00 | 0.00E+00 | 0.00E+00 | 0.00E+00 | 1.20E-08 | 4.64E-07 | 7.16E-07 |
| 101 | 0.00E+00 | 0.00E+00 | 0.00E+00 | 0.00E+00 | 0.00E+00 | 0.00E+00 | 0.00E+00 | 4.49E-07 | 6.99E-07 |
| 102 | 0.00E+00 | 0.00E+00 | 0.00E+00 | 0.00E+00 | 0.00E+00 | 0.00E+00 | 0.00E+00 | 4.35E-07 | 6.82E-07 |
| 103 | 0.00E+00 | 0.00E+00 | 0.00E+00 | 0.00E+00 | 0.00E+00 | 0.00E+00 | 0.00E+00 | 4.18E-07 | 6.65E-07 |
| 104 | 0.00E+00 | 0.00E+00 | 0.00E+00 | 0.00E+00 | 0.00E+00 | 0.00E+00 | 0.00E+00 | 4.01E-07 | 6.46E-07 |
| 105 | 0.00E+00 | 0.00E+00 | 0.00E+00 | 0.00E+00 | 0.00E+00 | 0.00E+00 | 0.00E+00 | 3.81E-07 | 6.26E-07 |
| 106 | 0.00E+00 | 0.00E+00 | 0.00E+00 | 0.00E+00 | 0.00E+00 | 0.00E+00 | 0.00E+00 | 3.61E-07 | 6.07E-07 |
| 107 | 0.00E+00 | 0.00E+00 | 0.00E+00 | 0.00E+00 | 0.00E+00 | 0.00E+00 | 0.00E+00 | 3.41E-07 | 5.89E-07 |
| 108 | 0.00E+00 | 0.00E+00 | 0.00E+00 | 0.00E+00 | 0.00E+00 | 0.00E+00 | 0.00E+00 | 3.27E-07 | 5.73E-07 |
| 109 | 0.00E+00 | 0.00E+00 | 0.00E+00 | 0.00E+00 | 0.00E+00 | 0.00E+00 | 0.00E+00 | 3.12E-07 | 5.58E-07 |
| 110 | 0.00E+00 | 0.00E+00 | 0.00E+00 | 0.00E+00 | 0.00E+00 | 0.00E+00 | 0.00E+00 | 3.00E-07 | 5.45E-07 |
| 111 | 0.00E+00 | 0.00E+00 | 0.00E+00 | 0.00E+00 | 0.00E+00 | 0.00E+00 | 0.00E+00 | 2.86E-07 | 5.31E-07 |
| 112 | 0.00E+00 | 0.00E+00 | 0.00E+00 | 0.00E+00 | 0.00E+00 | 0.00E+00 | 0.00E+00 | 2.71E-07 | 5.16E-07 |
| 113 | 0.00E+00 | 0.00E+00 | 0.00E+00 | 0.00E+00 | 0.00E+00 | 0.00E+00 | 0.00E+00 | 2.51E-07 | 4.99E-07 |
| 114 | 0.00E+00 | 0.00E+00 | 0.00E+00 | 0.00E+00 | 0.00E+00 | 0.00E+00 | 0.00E+00 | 2.31E-07 | 4.80E-07 |
| 115 | 0.00E+00 | 0.00E+00 | 0.00E+00 | 0.00E+00 | 0.00E+00 | 0.00E+00 | 0.00E+00 | 2.09E-07 | 4.60E-07 |
| 116 | 0.00E+00 | 0.00E+00 | 0.00E+00 | 0.00E+00 | 0.00E+00 | 0.00E+00 | 0.00E+00 | 1.90E-07 | 4.39E-07 |
| 117 | 0.00E+00 | 0.00E+00 | 0.00E+00 | 0.00E+00 | 0.00E+00 | 0.00E+00 | 0.00E+00 | 1.73E-07 | 4.19E-07 |
| 118 | 0.00E+00 | 0.00E+00 | 0.00E+00 | 0.00E+00 | 0.00E+00 | 0.00E+00 | 0.00E+00 | 1.59E-07 | 4.00E-07 |
| 119 | 0.00E+00 | 0.00E+00 | 0.00E+00 | 0.00E+00 | 0.00E+00 | 0.00E+00 | 0.00E+00 | 1.46E-07 | 3.84E-07 |
| 120 | 0.00E+00 | 0.00E+00 | 0.00E+00 | 0.00E+00 | 0.00E+00 | 0.00E+00 | 0.00E+00 | 1.35E-07 | 3.69E-07 |
| 121 | 0.00E+00 | 0.00E+00 | 0.00E+00 | 0.00E+00 | 0.00E+00 | 0.00E+00 | 0.00E+00 | 1.23E-07 | 3.56E-07 |
| 122 | 0.00E+00 | 0.00E+00 | 0.00E+00 | 0.00E+00 | 0.00E+00 | 0.00E+00 | 0.00E+00 | 1.11E-07 | 3.43E-07 |
| 123 | 0.00E+00 | 0.00E+00 | 0.00E+00 | 0.00E+00 | 0.00E+00 | 0.00E+00 | 0.00E+00 | 9.71E-08 | 3.30E-07 |
| 124 | 0.00E+00 | 0.00E+00 | 0.00E+00 | 0.00E+00 | 0.00E+00 | 0.00E+00 | 0.00E+00 | 8.30E-08 | 3.18E-07 |
| 125 | 0.00E+00 | 0.00E+00 | 0.00E+00 | 0.00E+00 | 0.00E+00 | 0.00E+00 | 0.00E+00 | 6.92E-08 | 3.05E-07 |
| 126 | 0.00E+00 | 0.00E+00 | 0.00E+00 | 0.00E+00 | 0.00E+00 | 0.00E+00 | 0.00E+00 | 5.54E-08 | 2.92E-07 |
| 127 | 0.00E+00 | 0.00E+00 | 0.00E+00 | 0.00E+00 | 0.00E+00 | 0.00E+00 | 0.00E+00 | 4.28E-08 | 2.78E-07 |
| 128 | 0.00E+00 | 0.00E+00 | 0.00E+00 | 0.00E+00 | 0.00E+00 | 0.00E+00 | 0.00E+00 | 3.04E-08 | 2.62E-07 |
| 129 | 0.00E+00 | 0.00E+00 | 0.00E+00 | 0.00E+00 | 0.00E+00 | 0.00E+00 | 0.00E+00 | 1.79E-08 | 2.47E-07 |
| 130 | 0.00E+00 | 0.00E+00 | 0.00E+00 | 0.00E+00 | 0.00E+00 | 0.00E+00 | 0.00E+00 | 4.54E-09 | 2.32E-07 |
| 131 | 0.00E+00 | 0.00E+00 | 0.00E+00 | 0.00E+00 | 0.00E+00 | 0.00E+00 | 0.00E+00 | 0.00E+00 | 2.17E-07 |
| 132 | 0.00E+00 | 0.00E+00 | 0.00E+00 | 0.00E+00 | 0.00E+00 | 0.00E+00 | 0.00E+00 | 0.00E+00 | 2.03E-07 |
| 133 | 0.00E+00 | 0.00E+00 | 0.00E+00 | 0.00E+00 | 0.00E+00 | 0.00E+00 | 0.00E+00 | 0.00E+00 | 1.90E-07 |
| 134 | 0.00E+00 | 0.00E+00 | 0.00E+00 | 0.00E+00 | 0.00E+00 | 0.00E+00 | 0.00E+00 | 0.00E+00 | 1.78E-07 |
| 135 | 0.00E+00 | 0.00E+00 | 0.00E+00 | 0.00E+00 | 0.00E+00 | 0.00E+00 | 0.00E+00 | 0.00E+00 | 1.66E-07 |
| 136 | 0.00E+00 | 0.00E+00 | 0.00E+00 | 0.00E+00 | 0.00E+00 | 0.00E+00 | 0.00E+00 | 0.00E+00 | 1.55E-07 |
| 137 | 0.00E+00 | 0.00E+00 | 0.00E+00 | 0.00E+00 | 0.00E+00 | 0.00E+00 | 0.00E+00 | 0.00E+00 | 1.43E-07 |
| 138 | 0.00E+00 | 0.00E+00 | 0.00E+00 | 0.00E+00 | 0.00E+00 | 0.00E+00 | 0.00E+00 | 0.00E+00 | 1.31E-07 |
| 139 | 0.00E+00 | 0.00E+00 | 0.00E+00 | 0.00E+00 | 0.00E+00 | 0.00E+00 | 0.00E+00 | 0.00E+00 | 1.20E-07 |
| 140 | 0.00E+00 | 0.00E+00 | 0.00E+00 | 0.00E+00 | 0.00E+00 | 0.00E+00 | 0.00E+00 | 0.00E+00 | 1.08E-07 |
| 141 | 0.00E+00 | 0.00E+00 | 0.00E+00 | 0.00E+00 | 0.00E+00 | 0.00E+00 | 0.00E+00 | 0.00E+00 | 9.71E-08 |
| 142 | 0.00E+00 | 0.00E+00 | 0.00E+00 | 0.00E+00 | 0.00E+00 | 0.00E+00 | 0.00E+00 | 0.00E+00 | 8.67E-08 |
| 143 | 0.00E+00 | 0.00E+00 | 0.00E+00 | 0.00E+00 | 0.00E+00 | 0.00E+00 | 0.00E+00 | 0.00E+00 | 7.68E-08 |
| 144 | 0.00E+00 | 0.00E+00 | 0.00E+00 | 0.00E+00 | 0.00E+00 | 0.00E+00 | 0.00E+00 | 0.00E+00 | 6.72E-08 |
| 145 | 0.00E+00 | 0.00E+00 | 0.00E+00 | 0.00E+00 | 0.00E+00 | 0.00E+00 | 0.00E+00 | 0.00E+00 | 5.75E-08 |
| 146 | 0.00E+00 | 0.00E+00 | 0.00E+00 | 0.00E+00 | 0.00E+00 | 0.00E+00 | 0.00E+00 | 0.00E+00 | 4.77E-08 |
| 147 | 0.00E+00 | 0.00E+00 | 0.00E+00 | 0.00E+00 | 0.00E+00 | 0.00E+00 | 0.00E+00 | 0.00E+00 | 3.75E-08 |
| 148 | 0.00E+00 | 0.00E+00 | 0.00E+00 | 0.00E+00 | 0.00E+00 | 0.00E+00 | 0.00E+00 | 0.00E+00 | 2.67E-08 |
| 149 | 0.00E+00 | 0.00E+00 | 0.00E+00 | 0.00E+00 | 0.00E+00 | 0.00E+00 | 0.00E+00 | 0.00E+00 | 1.57E-08 |
| 150 | 0.00E+00 | 0.00E+00 | 0.00E+00 | 0.00E+00 | 0.00E+00 | 0.00E+00 | 0.00E+00 | 0.00E+00 | 3.71E-09 |

Table.10. Spectra obtained by MCNP code: Al- 1.6mm

| energy bin (kev)/voltage(kv) | 20kv | 30kv | 40kv | 50kv | 60kv | 80kv | 100kv | 130kv | 150kv |
| --- | --- | --- | --- | --- | --- | --- | --- | --- | --- |
| 1 | 0.00E+00 | 0.00E+00 | 0.00E+00 | 0.00E+00 | 0.00E+00 | 0.00E+00 | 0.00E+00 | 0.00E+00 | 0.00E+00 |
| 2 | ######## | ######## | ######## | ######## | ######## | ######## | ######## | ######## | ######## |
| 3 | ######## | ######## | ######## | ######## | ######## | ######## | ######## | ######## | ######## |
| 4 | 4.35E-53 | 2.74E-53 | 2.55E-53 | 2.30E-53 | 2.12E-53 | 1.96E-53 | 1.88E-53 | 2.22E-53 | 1.94E-53 |
| 5 | 2.65E-31 | 3.56E-31 | 3.48E-31 | 3.37E-31 | 3.11E-31 | 2.79E-31 | 2.63E-31 | 2.89E-31 | 2.43E-31 |
| 6 | 3.77E-21 | 5.96E-21 | 6.49E-21 | 6.50E-21 | 6.36E-21 | 5.83E-21 | 5.32E-21 | 5.40E-21 | 4.66E-21 |
| 7 | 6.31E-17 | 9.83E-17 | 1.13E-16 | 1.20E-16 | 1.22E-16 | 1.16E-16 | 1.11E-16 | 1.15E-16 | 9.42E-17 |
| 8 | 9.78E-13 | 1.52E-12 | 1.84E-12 | 2.07E-12 | 2.18E-12 | 2.24E-12 | 2.15E-12 | 2.26E-12 | 1.95E-12 |
| 9 | 3.20E-11 | 6.55E-11 | 9.00E-11 | 1.11E-10 | 1.26E-10 | 1.48E-10 | 1.60E-10 | 1.86E-10 | 1.65E-10 |
| 10 | 1.03E-09 | 1.72E-09 | 2.26E-09 | 2.75E-09 | 3.11E-09 | 3.54E-09 | 3.79E-09 | 4.29E-09 | 3.79E-09 |
| 11 | 2.59E-09 | 3.92E-09 | 5.05E-09 | 5.78E-09 | 6.28E-09 | 6.65E-09 | 6.65E-09 | 7.31E-09 | 6.23E-09 |
| 12 | 6.28E-09 | 1.04E-08 | 1.31E-08 | 1.51E-08 | 1.60E-08 | 1.66E-08 | 1.67E-08 | 1.76E-08 | 1.46E-08 |
| 13 | 1.51E-08 | 2.60E-08 | 3.30E-08 | 3.75E-08 | 4.03E-08 | 3.99E-08 | 3.99E-08 | 4.08E-08 | 3.48E-08 |
| 14 | 3.78E-08 | 7.13E-08 | 9.36E-08 | 1.08E-07 | 1.21E-07 | 1.28E-07 | 1.30E-07 | 1.35E-07 | 1.15E-07 |
| 15 | 7.59E-08 | 1.66E-07 | 2.28E-07 | 2.76E-07 | 3.08E-07 | 3.40E-07 | 3.55E-07 | 3.84E-07 | 3.34E-07 |
| 16 | 8.41E-08 | 2.21E-07 | 3.16E-07 | 3.95E-07 | 4.48E-07 | 5.14E-07 | 5.50E-07 | 6.13E-07 | 5.45E-07 |
| 17 | 9.17E-08 | 2.84E-07 | 4.18E-07 | 5.32E-07 | 6.09E-07 | 7.18E-07 | 7.80E-07 | 8.81E-07 | 7.95E-07 |
| 18 | 8.08E-08 | 3.36E-07 | 5.13E-07 | 6.65E-07 | 7.72E-07 | 9.36E-07 | 1.04E-06 | 1.19E-06 | 1.09E-06 |
| 19 | 6.89E-08 | 3.75E-07 | 5.94E-07 | 7.84E-07 | 9.23E-07 | 1.14E-06 | 1.29E-06 | 1.50E-06 | 1.39E-06 |
| 20 | 3.72E-08 | 4.00E-07 | 6.56E-07 | 8.83E-07 | 1.05E-06 | 1.33E-06 | 1.52E-06 | 1.79E-06 | 1.69E-06 |
| 21 | 0.00E+00 | 4.10E-07 | 7.00E-07 | 9.58E-07 | 1.16E-06 | 1.49E-06 | 1.73E-06 | 2.06E-06 | 1.96E-06 |
| 22 | 0.00E+00 | 4.02E-07 | 7.20E-07 | 1.00E-06 | 1.23E-06 | 1.60E-06 | 1.90E-06 | 2.28E-06 | 2.20E-06 |
| 23 | 0.00E+00 | 3.75E-07 | 7.10E-07 | 1.01E-06 | 1.27E-06 | 1.67E-06 | 2.01E-06 | 2.43E-06 | 2.39E-06 |
| 24 | 0.00E+00 | 3.45E-07 | 6.99E-07 | 1.02E-06 | 1.30E-06 | 1.73E-06 | 2.11E-06 | 2.58E-06 | 2.58E-06 |
| 25 | 0.00E+00 | 3.10E-07 | 6.89E-07 | 1.02E-06 | 1.32E-06 | 1.80E-06 | 2.21E-06 | 2.74E-06 | 2.77E-06 |
| 26 | 0.00E+00 | 2.71E-07 | 6.75E-07 | 1.03E-06 | 1.34E-06 | 1.86E-06 | 2.31E-06 | 2.90E-06 | 2.96E-06 |
| 27 | 0.00E+00 | 2.27E-07 | 6.60E-07 | 1.03E-06 | 1.37E-06 | 1.93E-06 | 2.41E-06 | 3.05E-06 | 3.15E-06 |
| 28 | 0.00E+00 | 1.73E-07 | 6.43E-07 | 1.03E-06 | 1.38E-06 | 1.99E-06 | 2.51E-06 | 3.20E-06 | 3.34E-06 |
| 29 | 0.00E+00 | 1.17E-07 | 6.18E-07 | 1.02E-06 | 1.38E-06 | 2.03E-06 | 2.58E-06 | 3.31E-06 | 3.50E-06 |
| 30 | 0.00E+00 | 4.63E-08 | 5.83E-07 | 9.97E-07 | 1.37E-06 | 2.04E-06 | 2.63E-06 | 3.38E-06 | 3.62E-06 |
| 31 | 0.00E+00 | 0.00E+00 | 5.42E-07 | 9.65E-07 | 1.34E-06 | 2.03E-06 | 2.65E-06 | 3.40E-06 | 3.70E-06 |
| 32 | 0.00E+00 | 0.00E+00 | 4.92E-07 | 9.25E-07 | 1.30E-06 | 2.00E-06 | 2.63E-06 | 3.39E-06 | 3.75E-06 |
| 33 | 0.00E+00 | 0.00E+00 | 4.38E-07 | 8.75E-07 | 1.25E-06 | 1.94E-06 | 2.59E-06 | 3.34E-06 | 3.75E-06 |
| 34 | 0.00E+00 | 0.00E+00 | 3.83E-07 | 8.25E-07 | 1.20E-06 | 1.88E-06 | 2.54E-06 | 3.30E-06 | 3.73E-06 |
| 35 | 0.00E+00 | 0.00E+00 | 3.26E-07 | 7.77E-07 | 1.16E-06 | 1.82E-06 | 2.49E-06 | 3.26E-06 | 3.72E-06 |
| 36 | 0.00E+00 | 0.00E+00 | 2.70E-07 | 7.28E-07 | 1.11E-06 | 1.76E-06 | 2.44E-06 | 3.22E-06 | 3.69E-06 |
| 37 | 0.00E+00 | 0.00E+00 | 2.13E-07 | 6.77E-07 | 1.06E-06 | 1.71E-06 | 2.38E-06 | 3.19E-06 | 3.65E-06 |
| 38 | 0.00E+00 | 0.00E+00 | 1.56E-07 | 6.29E-07 | 1.01E-06 | 1.65E-06 | 2.33E-06 | 3.15E-06 | 3.61E-06 |
| 39 | 0.00E+00 | 0.00E+00 | 9.82E-08 | 5.77E-07 | 9.61E-07 | 1.61E-06 | 2.27E-06 | 3.11E-06 | 3.57E-06 |
| 40 | 0.00E+00 | 0.00E+00 | 4.36E-08 | 5.26E-07 | 9.08E-07 | 1.56E-06 | 2.21E-06 | 3.06E-06 | 3.52E-06 |
| 41 | 0.00E+00 | 0.00E+00 | 0.00E+00 | 4.75E-07 | 8.57E-07 | 1.52E-06 | 2.15E-06 | 3.00E-06 | 3.46E-06 |
| 42 | 0.00E+00 | 0.00E+00 | 0.00E+00 | 4.25E-07 | 8.08E-07 | 1.47E-06 | 2.09E-06 | 2.93E-06 | 3.40E-06 |
| 43 | 0.00E+00 | 0.00E+00 | 0.00E+00 | 3.72E-07 | 7.61E-07 | 1.42E-06 | 2.02E-06 | 2.85E-06 | 3.33E-06 |
| 44 | 0.00E+00 | 0.00E+00 | 0.00E+00 | 3.24E-07 | 7.12E-07 | 1.36E-06 | 1.96E-06 | 2.78E-06 | 3.26E-06 |
| 45 | 0.00E+00 | 0.00E+00 | 0.00E+00 | 2.76E-07 | 6.65E-07 | 1.31E-06 | 1.91E-06 | 2.71E-06 | 3.19E-06 |
| 46 | 0.00E+00 | 0.00E+00 | 0.00E+00 | 2.28E-07 | 6.17E-07 | 1.26E-06 | 1.85E-06 | 2.65E-06 | 3.12E-06 |
| 47 | 0.00E+00 | 0.00E+00 | 0.00E+00 | 1.81E-07 | 5.72E-07 | 1.20E-06 | 1.80E-06 | 2.58E-06 | 3.06E-06 |
| 48 | 0.00E+00 | 0.00E+00 | 0.00E+00 | 1.33E-07 | 5.28E-07 | 1.16E-06 | 1.74E-06 | 2.51E-06 | 3.00E-06 |
| 49 | 0.00E+00 | 0.00E+00 | 0.00E+00 | 8.52E-08 | 4.85E-07 | 1.11E-06 | 1.68E-06 | 2.45E-06 | 2.94E-06 |
| 50 | 0.00E+00 | 0.00E+00 | 0.00E+00 | 3.55E-08 | 4.43E-07 | 1.06E-06 | 1.63E-06 | 2.38E-06 | 2.87E-06 |
| 51 | 0.00E+00 | 0.00E+00 | 0.00E+00 | 0.00E+00 | 4.00E-07 | 1.01E-06 | 1.57E-06 | 2.31E-06 | 2.81E-06 |
| 52 | 0.00E+00 | 0.00E+00 | 0.00E+00 | 0.00E+00 | 3.57E-07 | 9.59E-07 | 1.52E-06 | 2.26E-06 | 2.74E-06 |
| 53 | 0.00E+00 | 0.00E+00 | 0.00E+00 | 0.00E+00 | 3.14E-07 | 9.10E-07 | 1.48E-06 | 2.21E-06 | 2.68E-06 |
| 54 | 0.00E+00 | 0.00E+00 | 0.00E+00 | 0.00E+00 | 2.71E-07 | 8.63E-07 | 1.44E-06 | 2.16E-06 | 2.62E-06 |
| 55 | 0.00E+00 | 0.00E+00 | 0.00E+00 | 0.00E+00 | 2.29E-07 | 8.19E-07 | 1.39E-06 | 2.11E-06 | 2.57E-06 |
| 56 | 0.00E+00 | 0.00E+00 | 0.00E+00 | 0.00E+00 | 1.88E-07 | 7.79E-07 | 1.34E-06 | 2.05E-06 | 2.51E-06 |
| 57 | 0.00E+00 | 0.00E+00 | 0.00E+00 | 0.00E+00 | 1.47E-07 | 7.42E-07 | 1.29E-06 | 1.99E-06 | 2.46E-06 |
| 58 | 0.00E+00 | 0.00E+00 | 0.00E+00 | 0.00E+00 | 1.05E-07 | 7.08E-07 | 1.21E-06 | 1.91E-06 | 2.40E-06 |
| 59 | 0.00E+00 | 0.00E+00 | 0.00E+00 | 0.00E+00 | 6.67E-08 | 8.14E-07 | 2.15E-06 | 4.64E-06 | 6.27E-06 |
| 60 | 0.00E+00 | 0.00E+00 | 0.00E+00 | 0.00E+00 | 2.58E-08 | 9.05E-07 | 2.85E-06 | 6.83E-06 | 9.49E-06 |
| 61 | 0.00E+00 | 0.00E+00 | 0.00E+00 | 0.00E+00 | 0.00E+00 | 5.86E-07 | 1.05E-06 | 1.79E-06 | 2.29E-06 |
| 62 | 0.00E+00 | 0.00E+00 | 0.00E+00 | 0.00E+00 | 0.00E+00 | 5.72E-07 | 1.14E-06 | 1.86E-06 | 2.46E-06 |
| 63 | 0.00E+00 | 0.00E+00 | 0.00E+00 | 0.00E+00 | 0.00E+00 | 5.71E-07 | 9.98E-07 | 1.56E-06 | 2.01E-06 |
| 64 | 0.00E+00 | 0.00E+00 | 0.00E+00 | 0.00E+00 | 0.00E+00 | 4.95E-07 | 9.81E-07 | 1.49E-06 | 1.96E-06 |
| 65 | 0.00E+00 | 0.00E+00 | 0.00E+00 | 0.00E+00 | 0.00E+00 | 4.62E-07 | 9.39E-07 | 1.49E-06 | 1.94E-06 |
| 66 | 0.00E+00 | 0.00E+00 | 0.00E+00 | 0.00E+00 | 0.00E+00 | 4.34E-07 | 8.84E-07 | 1.47E-06 | 1.91E-06 |
| 67 | 0.00E+00 | 0.00E+00 | 0.00E+00 | 0.00E+00 | 0.00E+00 | 4.13E-07 | 8.16E-07 | 1.42E-06 | 1.88E-06 |
| 68 | 0.00E+00 | 0.00E+00 | 0.00E+00 | 0.00E+00 | 0.00E+00 | 4.68E-07 | 1.46E-06 | 3.13E-06 | 4.44E-06 |
| 69 | 0.00E+00 | 0.00E+00 | 0.00E+00 | 0.00E+00 | 0.00E+00 | 3.27E-07 | 7.76E-07 | 1.39E-06 | 1.70E-06 |
| 70 | 0.00E+00 | 0.00E+00 | 0.00E+00 | 0.00E+00 | 0.00E+00 | 3.18E-07 | 9.12E-07 | 1.78E-06 | 2.38E-06 |
| 71 | 0.00E+00 | 0.00E+00 | 0.00E+00 | 0.00E+00 | 0.00E+00 | 2.69E-07 | 7.21E-07 | 1.20E-06 | 1.42E-06 |
| 72 | 0.00E+00 | 0.00E+00 | 0.00E+00 | 0.00E+00 | 0.00E+00 | 2.41E-07 | 6.41E-07 | 1.18E-06 | 1.43E-06 |
| 73 | 0.00E+00 | 0.00E+00 | 0.00E+00 | 0.00E+00 | 0.00E+00 | 2.09E-07 | 6.10E-07 | 1.13E-06 | 1.39E-06 |
| 74 | 0.00E+00 | 0.00E+00 | 0.00E+00 | 0.00E+00 | 0.00E+00 | 1.80E-07 | 5.83E-07 | 1.10E-06 | 1.36E-06 |
| 75 | 0.00E+00 | 0.00E+00 | 0.00E+00 | 0.00E+00 | 0.00E+00 | 1.51E-07 | 5.57E-07 | 1.07E-06 | 1.32E-06 |
| 76 | 0.00E+00 | 0.00E+00 | 0.00E+00 | 0.00E+00 | 0.00E+00 | 1.23E-07 | 5.33E-07 | 1.04E-06 | 1.29E-06 |
| 77 | 0.00E+00 | 0.00E+00 | 0.00E+00 | 0.00E+00 | 0.00E+00 | 9.63E-08 | 5.13E-07 | 1.01E-06 | 1.26E-06 |
| 78 | 0.00E+00 | 0.00E+00 | 0.00E+00 | 0.00E+00 | 0.00E+00 | 6.94E-08 | 4.93E-07 | 9.80E-07 | 1.24E-06 |
| 79 | 0.00E+00 | 0.00E+00 | 0.00E+00 | 0.00E+00 | 0.00E+00 | 4.22E-08 | 4.73E-07 | 9.50E-07 | 1.21E-06 |
| 80 | 0.00E+00 | 0.00E+00 | 0.00E+00 | 0.00E+00 | 0.00E+00 | 1.46E-08 | 4.50E-07 | 9.15E-07 | 1.19E-06 |
| 81 | 0.00E+00 | 0.00E+00 | 0.00E+00 | 0.00E+00 | 0.00E+00 | 0.00E+00 | 4.26E-07 | 8.80E-07 | 1.16E-06 |
| 82 | 0.00E+00 | 0.00E+00 | 0.00E+00 | 0.00E+00 | 0.00E+00 | 0.00E+00 | 4.02E-07 | 8.51E-07 | 1.14E-06 |
| 83 | 0.00E+00 | 0.00E+00 | 0.00E+00 | 0.00E+00 | 0.00E+00 | 0.00E+00 | 3.77E-07 | 8.21E-07 | 1.11E-06 |
| 84 | 0.00E+00 | 0.00E+00 | 0.00E+00 | 0.00E+00 | 0.00E+00 | 0.00E+00 | 3.53E-07 | 7.92E-07 | 1.09E-06 |
| 85 | 0.00E+00 | 0.00E+00 | 0.00E+00 | 0.00E+00 | 0.00E+00 | 0.00E+00 | 3.31E-07 | 7.68E-07 | 1.06E-06 |
| 86 | 0.00E+00 | 0.00E+00 | 0.00E+00 | 0.00E+00 | 0.00E+00 | 0.00E+00 | 3.08E-07 | 7.46E-07 | 1.04E-06 |
| 87 | 0.00E+00 | 0.00E+00 | 0.00E+00 | 0.00E+00 | 0.00E+00 | 0.00E+00 | 2.84E-07 | 7.26E-07 | 1.01E-06 |
| 88 | 0.00E+00 | 0.00E+00 | 0.00E+00 | 0.00E+00 | 0.00E+00 | 0.00E+00 | 2.60E-07 | 7.07E-07 | 9.88E-07 |
| 89 | 0.00E+00 | 0.00E+00 | 0.00E+00 | 0.00E+00 | 0.00E+00 | 0.00E+00 | 2.36E-07 | 6.88E-07 | 9.64E-07 |
| 90 | 0.00E+00 | 0.00E+00 | 0.00E+00 | 0.00E+00 | 0.00E+00 | 0.00E+00 | 2.12E-07 | 6.69E-07 | 9.42E-07 |
| 91 | 0.00E+00 | 0.00E+00 | 0.00E+00 | 0.00E+00 | 0.00E+00 | 0.00E+00 | 1.91E-07 | 6.48E-07 | 9.20E-07 |
| 92 | 0.00E+00 | 0.00E+00 | 0.00E+00 | 0.00E+00 | 0.00E+00 | 0.00E+00 | 1.70E-07 | 6.28E-07 | 8.97E-07 |
| 93 | 0.00E+00 | 0.00E+00 | 0.00E+00 | 0.00E+00 | 0.00E+00 | 0.00E+00 | 1.52E-07 | 6.08E-07 | 8.73E-07 |
| 94 | 0.00E+00 | 0.00E+00 | 0.00E+00 | 0.00E+00 | 0.00E+00 | 0.00E+00 | 1.33E-07 | 5.87E-07 | 8.47E-07 |
| 95 | 0.00E+00 | 0.00E+00 | 0.00E+00 | 0.00E+00 | 0.00E+00 | 0.00E+00 | 1.13E-07 | 5.65E-07 | 8.19E-07 |
| 96 | 0.00E+00 | 0.00E+00 | 0.00E+00 | 0.00E+00 | 0.00E+00 | 0.00E+00 | 9.30E-08 | 5.41E-07 | 7.91E-07 |
| 97 | 0.00E+00 | 0.00E+00 | 0.00E+00 | 0.00E+00 | 0.00E+00 | 0.00E+00 | 7.20E-08 | 5.18E-07 | 7.67E-07 |
| 98 | 0.00E+00 | 0.00E+00 | 0.00E+00 | 0.00E+00 | 0.00E+00 | 0.00E+00 | 5.10E-08 | 4.95E-07 | 7.43E-07 |
| 99 | 0.00E+00 | 0.00E+00 | 0.00E+00 | 0.00E+00 | 0.00E+00 | 0.00E+00 | 3.00E-08 | 4.75E-07 | 7.23E-07 |
| 100 | 0.00E+00 | 0.00E+00 | 0.00E+00 | 0.00E+00 | 0.00E+00 | 0.00E+00 | 1.19E-08 | 4.58E-07 | 7.07E-07 |
| 101 | 0.00E+00 | 0.00E+00 | 0.00E+00 | 0.00E+00 | 0.00E+00 | 0.00E+00 | 0.00E+00 | 4.44E-07 | 6.91E-07 |
| 102 | 0.00E+00 | 0.00E+00 | 0.00E+00 | 0.00E+00 | 0.00E+00 | 0.00E+00 | 0.00E+00 | 4.30E-07 | 6.73E-07 |
| 103 | 0.00E+00 | 0.00E+00 | 0.00E+00 | 0.00E+00 | 0.00E+00 | 0.00E+00 | 0.00E+00 | 4.13E-07 | 6.56E-07 |
| 104 | 0.00E+00 | 0.00E+00 | 0.00E+00 | 0.00E+00 | 0.00E+00 | 0.00E+00 | 0.00E+00 | 3.96E-07 | 6.38E-07 |
| 105 | 0.00E+00 | 0.00E+00 | 0.00E+00 | 0.00E+00 | 0.00E+00 | 0.00E+00 | 0.00E+00 | 3.77E-07 | 6.18E-07 |
| 106 | 0.00E+00 | 0.00E+00 | 0.00E+00 | 0.00E+00 | 0.00E+00 | 0.00E+00 | 0.00E+00 | 3.56E-07 | 5.99E-07 |
| 107 | 0.00E+00 | 0.00E+00 | 0.00E+00 | 0.00E+00 | 0.00E+00 | 0.00E+00 | 0.00E+00 | 3.36E-07 | 5.82E-07 |
| 108 | 0.00E+00 | 0.00E+00 | 0.00E+00 | 0.00E+00 | 0.00E+00 | 0.00E+00 | 0.00E+00 | 3.23E-07 | 5.66E-07 |
| 109 | 0.00E+00 | 0.00E+00 | 0.00E+00 | 0.00E+00 | 0.00E+00 | 0.00E+00 | 0.00E+00 | 3.08E-07 | 5.51E-07 |
| 110 | 0.00E+00 | 0.00E+00 | 0.00E+00 | 0.00E+00 | 0.00E+00 | 0.00E+00 | 0.00E+00 | 2.96E-07 | 5.38E-07 |
| 111 | 0.00E+00 | 0.00E+00 | 0.00E+00 | 0.00E+00 | 0.00E+00 | 0.00E+00 | 0.00E+00 | 2.83E-07 | 5.24E-07 |
| 112 | 0.00E+00 | 0.00E+00 | 0.00E+00 | 0.00E+00 | 0.00E+00 | 0.00E+00 | 0.00E+00 | 2.68E-07 | 5.10E-07 |
| 113 | 0.00E+00 | 0.00E+00 | 0.00E+00 | 0.00E+00 | 0.00E+00 | 0.00E+00 | 0.00E+00 | 2.48E-07 | 4.93E-07 |
| 114 | 0.00E+00 | 0.00E+00 | 0.00E+00 | 0.00E+00 | 0.00E+00 | 0.00E+00 | 0.00E+00 | 2.28E-07 | 4.74E-07 |
| 115 | 0.00E+00 | 0.00E+00 | 0.00E+00 | 0.00E+00 | 0.00E+00 | 0.00E+00 | 0.00E+00 | 2.07E-07 | 4.54E-07 |
| 116 | 0.00E+00 | 0.00E+00 | 0.00E+00 | 0.00E+00 | 0.00E+00 | 0.00E+00 | 0.00E+00 | 1.88E-07 | 4.34E-07 |
| 117 | 0.00E+00 | 0.00E+00 | 0.00E+00 | 0.00E+00 | 0.00E+00 | 0.00E+00 | 0.00E+00 | 1.71E-07 | 4.14E-07 |
| 118 | 0.00E+00 | 0.00E+00 | 0.00E+00 | 0.00E+00 | 0.00E+00 | 0.00E+00 | 0.00E+00 | 1.57E-07 | 3.96E-07 |
| 119 | 0.00E+00 | 0.00E+00 | 0.00E+00 | 0.00E+00 | 0.00E+00 | 0.00E+00 | 0.00E+00 | 1.45E-07 | 3.80E-07 |
| 120 | 0.00E+00 | 0.00E+00 | 0.00E+00 | 0.00E+00 | 0.00E+00 | 0.00E+00 | 0.00E+00 | 1.34E-07 | 3.65E-07 |
| 121 | 0.00E+00 | 0.00E+00 | 0.00E+00 | 0.00E+00 | 0.00E+00 | 0.00E+00 | 0.00E+00 | 1.22E-07 | 3.52E-07 |
| 122 | 0.00E+00 | 0.00E+00 | 0.00E+00 | 0.00E+00 | 0.00E+00 | 0.00E+00 | 0.00E+00 | 1.10E-07 | 3.39E-07 |
| 123 | 0.00E+00 | 0.00E+00 | 0.00E+00 | 0.00E+00 | 0.00E+00 | 0.00E+00 | 0.00E+00 | 9.60E-08 | 3.27E-07 |
| 124 | 0.00E+00 | 0.00E+00 | 0.00E+00 | 0.00E+00 | 0.00E+00 | 0.00E+00 | 0.00E+00 | 8.21E-08 | 3.14E-07 |
| 125 | 0.00E+00 | 0.00E+00 | 0.00E+00 | 0.00E+00 | 0.00E+00 | 0.00E+00 | 0.00E+00 | 6.84E-08 | 3.02E-07 |
| 126 | 0.00E+00 | 0.00E+00 | 0.00E+00 | 0.00E+00 | 0.00E+00 | 0.00E+00 | 0.00E+00 | 5.48E-08 | 2.89E-07 |
| 127 | 0.00E+00 | 0.00E+00 | 0.00E+00 | 0.00E+00 | 0.00E+00 | 0.00E+00 | 0.00E+00 | 4.23E-08 | 2.75E-07 |
| 128 | 0.00E+00 | 0.00E+00 | 0.00E+00 | 0.00E+00 | 0.00E+00 | 0.00E+00 | 0.00E+00 | 3.01E-08 | 2.59E-07 |
| 129 | 0.00E+00 | 0.00E+00 | 0.00E+00 | 0.00E+00 | 0.00E+00 | 0.00E+00 | 0.00E+00 | 1.77E-08 | 2.44E-07 |
| 130 | 0.00E+00 | 0.00E+00 | 0.00E+00 | 0.00E+00 | 0.00E+00 | 0.00E+00 | 0.00E+00 | 4.48E-09 | 2.29E-07 |
| 131 | 0.00E+00 | 0.00E+00 | 0.00E+00 | 0.00E+00 | 0.00E+00 | 0.00E+00 | 0.00E+00 | 0.00E+00 | 2.14E-07 |
| 132 | 0.00E+00 | 0.00E+00 | 0.00E+00 | 0.00E+00 | 0.00E+00 | 0.00E+00 | 0.00E+00 | 0.00E+00 | 2.01E-07 |
| 133 | 0.00E+00 | 0.00E+00 | 0.00E+00 | 0.00E+00 | 0.00E+00 | 0.00E+00 | 0.00E+00 | 0.00E+00 | 1.88E-07 |
| 134 | 0.00E+00 | 0.00E+00 | 0.00E+00 | 0.00E+00 | 0.00E+00 | 0.00E+00 | 0.00E+00 | 0.00E+00 | 1.76E-07 |
| 135 | 0.00E+00 | 0.00E+00 | 0.00E+00 | 0.00E+00 | 0.00E+00 | 0.00E+00 | 0.00E+00 | 0.00E+00 | 1.65E-07 |
| 136 | 0.00E+00 | 0.00E+00 | 0.00E+00 | 0.00E+00 | 0.00E+00 | 0.00E+00 | 0.00E+00 | 0.00E+00 | 1.53E-07 |
| 137 | 0.00E+00 | 0.00E+00 | 0.00E+00 | 0.00E+00 | 0.00E+00 | 0.00E+00 | 0.00E+00 | 0.00E+00 | 1.42E-07 |
| 138 | 0.00E+00 | 0.00E+00 | 0.00E+00 | 0.00E+00 | 0.00E+00 | 0.00E+00 | 0.00E+00 | 0.00E+00 | 1.30E-07 |
| 139 | 0.00E+00 | 0.00E+00 | 0.00E+00 | 0.00E+00 | 0.00E+00 | 0.00E+00 | 0.00E+00 | 0.00E+00 | 1.18E-07 |
| 140 | 0.00E+00 | 0.00E+00 | 0.00E+00 | 0.00E+00 | 0.00E+00 | 0.00E+00 | 0.00E+00 | 0.00E+00 | 1.07E-07 |
| 141 | 0.00E+00 | 0.00E+00 | 0.00E+00 | 0.00E+00 | 0.00E+00 | 0.00E+00 | 0.00E+00 | 0.00E+00 | 9.61E-08 |
| 142 | 0.00E+00 | 0.00E+00 | 0.00E+00 | 0.00E+00 | 0.00E+00 | 0.00E+00 | 0.00E+00 | 0.00E+00 | 8.58E-08 |
| 143 | 0.00E+00 | 0.00E+00 | 0.00E+00 | 0.00E+00 | 0.00E+00 | 0.00E+00 | 0.00E+00 | 0.00E+00 | 7.60E-08 |
| 144 | 0.00E+00 | 0.00E+00 | 0.00E+00 | 0.00E+00 | 0.00E+00 | 0.00E+00 | 0.00E+00 | 0.00E+00 | 6.65E-08 |
| 145 | 0.00E+00 | 0.00E+00 | 0.00E+00 | 0.00E+00 | 0.00E+00 | 0.00E+00 | 0.00E+00 | 0.00E+00 | 5.69E-08 |
| 146 | 0.00E+00 | 0.00E+00 | 0.00E+00 | 0.00E+00 | 0.00E+00 | 0.00E+00 | 0.00E+00 | 0.00E+00 | 4.73E-08 |
| 147 | 0.00E+00 | 0.00E+00 | 0.00E+00 | 0.00E+00 | 0.00E+00 | 0.00E+00 | 0.00E+00 | 0.00E+00 | 3.71E-08 |
| 148 | 0.00E+00 | 0.00E+00 | 0.00E+00 | 0.00E+00 | 0.00E+00 | 0.00E+00 | 0.00E+00 | 0.00E+00 | 2.64E-08 |
| 149 | 0.00E+00 | 0.00E+00 | 0.00E+00 | 0.00E+00 | 0.00E+00 | 0.00E+00 | 0.00E+00 | 0.00E+00 | 1.55E-08 |
| 150 | 0.00E+00 | 0.00E+00 | 0.00E+00 | 0.00E+00 | 0.00E+00 | 0.00E+00 | 0.00E+00 | 0.00E+00 | 3.68E-09 |

Table.11. Spectra obtained by MCNP code: Al- 2mm

| energy bin (kev)/voltage(kv) | 20kv | 30kv | 40kv | 50kv | 60kv | 80kv | 100kv | 130kv | 150kv |
| --- | --- | --- | --- | --- | --- | --- | --- | --- | --- |
| 1 | 0.00E+00 | 0.00E+00 | 0.00E+00 | 0.00E+00 | 0.00E+00 | 0.00E+00 | 0.00E+00 | 0.00E+00 | 0.00E+00 |
| 2 | 0.00E+00 | 0.00E+00 | 0.00E+00 | 0.00E+00 | 0.00E+00 | 0.00E+00 | 0.00E+00 | 0.00E+00 | 0.00E+00 |
| 3 | ######## | ######## | ######## | ######## | ######## | ######## | ######## | ######## | ######## |
| 4 | 1.09E-64 | 6.86E-65 | 6.39E-65 | 5.75E-65 | 5.29E-65 | 4.91E-65 | 4.70E-65 | 5.56E-65 | 4.86E-65 |
| 5 | 1.66E-37 | 2.23E-37 | 2.18E-37 | 2.12E-37 | 1.95E-37 | 1.75E-37 | 1.65E-37 | 1.81E-37 | 1.52E-37 |
| 6 | 7.60E-25 | 1.20E-24 | 1.31E-24 | 1.31E-24 | 1.28E-24 | 1.17E-24 | 1.07E-24 | 1.09E-24 | 9.39E-25 |
| 7 | 1.38E-19 | 2.15E-19 | 2.46E-19 | 2.62E-19 | 2.67E-19 | 2.52E-19 | 2.41E-19 | 2.51E-19 | 2.06E-19 |
| 8 | 2.36E-14 | 3.68E-14 | 4.45E-14 | 5.01E-14 | 5.27E-14 | 5.41E-14 | 5.20E-14 | 5.46E-14 | 4.71E-14 |
| 9 | 1.88E-12 | 3.85E-12 | 5.29E-12 | 6.53E-12 | 7.43E-12 | 8.69E-12 | 9.39E-12 | 1.09E-11 | 9.67E-12 |
| 10 | 1.48E-10 | 2.48E-10 | 3.25E-10 | 3.96E-10 | 4.47E-10 | 5.09E-10 | 5.46E-10 | 6.17E-10 | 5.45E-10 |
| 11 | 4.86E-10 | 7.36E-10 | 9.48E-10 | 1.09E-09 | 1.18E-09 | 1.25E-09 | 1.25E-09 | 1.37E-09 | 1.17E-09 |
| 12 | 1.55E-09 | 2.57E-09 | 3.24E-09 | 3.73E-09 | 3.96E-09 | 4.10E-09 | 4.11E-09 | 4.35E-09 | 3.60E-09 |
| 13 | 4.87E-09 | 8.38E-09 | 1.06E-08 | 1.21E-08 | 1.30E-08 | 1.29E-08 | 1.29E-08 | 1.32E-08 | 1.12E-08 |
| 14 | 1.60E-08 | 3.02E-08 | 3.97E-08 | 4.60E-08 | 5.12E-08 | 5.42E-08 | 5.53E-08 | 5.70E-08 | 4.86E-08 |
| 15 | 4.17E-08 | 9.22E-08 | 1.27E-07 | 1.54E-07 | 1.71E-07 | 1.90E-07 | 1.98E-07 | 2.15E-07 | 1.87E-07 |
| 16 | 5.04E-08 | 1.35E-07 | 1.94E-07 | 2.43E-07 | 2.76E-07 | 3.18E-07 | 3.41E-07 | 3.81E-07 | 3.40E-07 |
| 17 | 5.81E-08 | 1.85E-07 | 2.74E-07 | 3.49E-07 | 4.00E-07 | 4.74E-07 | 5.16E-07 | 5.84E-07 | 5.28E-07 |
| 18 | 5.37E-08 | 2.34E-07 | 3.59E-07 | 4.67E-07 | 5.42E-07 | 6.60E-07 | 7.32E-07 | 8.41E-07 | 7.72E-07 |
| 19 | 4.91E-08 | 2.74E-07 | 4.35E-07 | 5.76E-07 | 6.79E-07 | 8.42E-07 | 9.50E-07 | 1.11E-06 | 1.03E-06 |
| 20 | 2.88E-08 | 3.03E-07 | 4.99E-07 | 6.73E-07 | 8.04E-07 | 1.01E-06 | 1.16E-06 | 1.37E-06 | 1.29E-06 |
| 21 | 0.00E+00 | 3.21E-07 | 5.48E-07 | 7.51E-07 | 9.10E-07 | 1.17E-06 | 1.36E-06 | 1.62E-06 | 1.54E-06 |
| 22 | 0.00E+00 | 3.22E-07 | 5.77E-07 | 8.05E-07 | 9.91E-07 | 1.29E-06 | 1.52E-06 | 1.83E-06 | 1.77E-06 |
| 23 | 0.00E+00 | 3.05E-07 | 5.79E-07 | 8.24E-07 | 1.04E-06 | 1.36E-06 | 1.64E-06 | 1.99E-06 | 1.96E-06 |
| 24 | 0.00E+00 | 2.85E-07 | 5.80E-07 | 8.45E-07 | 1.08E-06 | 1.44E-06 | 1.76E-06 | 2.15E-06 | 2.15E-06 |
| 25 | 0.00E+00 | 2.61E-07 | 5.81E-07 | 8.64E-07 | 1.12E-06 | 1.52E-06 | 1.87E-06 | 2.32E-06 | 2.34E-06 |
| 26 | 0.00E+00 | 2.32E-07 | 5.79E-07 | 8.82E-07 | 1.16E-06 | 1.60E-06 | 1.99E-06 | 2.49E-06 | 2.55E-06 |
| 27 | 0.00E+00 | 1.97E-07 | 5.77E-07 | 8.99E-07 | 1.19E-06 | 1.69E-06 | 2.11E-06 | 2.67E-06 | 2.76E-06 |
| 28 | 0.00E+00 | 1.52E-07 | 5.72E-07 | 9.15E-07 | 1.23E-06 | 1.77E-06 | 2.23E-06 | 2.85E-06 | 2.98E-06 |
| 29 | 0.00E+00 | 1.05E-07 | 5.57E-07 | 9.19E-07 | 1.25E-06 | 1.83E-06 | 2.33E-06 | 2.99E-06 | 3.16E-06 |
| 30 | 0.00E+00 | 4.26E-08 | 5.32E-07 | 9.10E-07 | 1.25E-06 | 1.87E-06 | 2.40E-06 | 3.08E-06 | 3.31E-06 |
| 31 | 0.00E+00 | 0.00E+00 | 4.99E-07 | 8.89E-07 | 1.24E-06 | 1.87E-06 | 2.44E-06 | 3.14E-06 | 3.41E-06 |
| 32 | 0.00E+00 | 0.00E+00 | 4.56E-07 | 8.57E-07 | 1.21E-06 | 1.85E-06 | 2.44E-06 | 3.15E-06 | 3.47E-06 |
| 33 | 0.00E+00 | 0.00E+00 | 4.07E-07 | 8.15E-07 | 1.17E-06 | 1.81E-06 | 2.41E-06 | 3.12E-06 | 3.49E-06 |
| 34 | 0.00E+00 | 0.00E+00 | 3.57E-07 | 7.72E-07 | 1.13E-06 | 1.76E-06 | 2.38E-06 | 3.08E-06 | 3.49E-06 |
| 35 | 0.00E+00 | 0.00E+00 | 3.06E-07 | 7.29E-07 | 1.09E-06 | 1.71E-06 | 2.34E-06 | 3.06E-06 | 3.49E-06 |
| 36 | 0.00E+00 | 0.00E+00 | 2.54E-07 | 6.86E-07 | 1.05E-06 | 1.66E-06 | 2.30E-06 | 3.04E-06 | 3.48E-06 |
| 37 | 0.00E+00 | 0.00E+00 | 2.01E-07 | 6.41E-07 | 1.00E-06 | 1.62E-06 | 2.26E-06 | 3.02E-06 | 3.46E-06 |
| 38 | 0.00E+00 | 0.00E+00 | 1.48E-07 | 5.98E-07 | 9.60E-07 | 1.57E-06 | 2.22E-06 | 3.00E-06 | 3.44E-06 |
| 39 | 0.00E+00 | 0.00E+00 | 9.36E-08 | 5.51E-07 | 9.16E-07 | 1.54E-06 | 2.17E-06 | 2.97E-06 | 3.41E-06 |
| 40 | 0.00E+00 | 0.00E+00 | 4.18E-08 | 5.03E-07 | 8.69E-07 | 1.50E-06 | 2.11E-06 | 2.93E-06 | 3.37E-06 |
| 41 | 0.00E+00 | 0.00E+00 | 0.00E+00 | 4.56E-07 | 8.22E-07 | 1.46E-06 | 2.06E-06 | 2.87E-06 | 3.32E-06 |
| 42 | 0.00E+00 | 0.00E+00 | 0.00E+00 | 4.08E-07 | 7.77E-07 | 1.41E-06 | 2.01E-06 | 2.81E-06 | 3.27E-06 |
| 43 | 0.00E+00 | 0.00E+00 | 0.00E+00 | 3.59E-07 | 7.33E-07 | 1.37E-06 | 1.95E-06 | 2.75E-06 | 3.20E-06 |
| 44 | 0.00E+00 | 0.00E+00 | 0.00E+00 | 3.13E-07 | 6.87E-07 | 1.31E-06 | 1.89E-06 | 2.68E-06 | 3.14E-06 |
| 45 | 0.00E+00 | 0.00E+00 | 0.00E+00 | 2.67E-07 | 6.42E-07 | 1.27E-06 | 1.84E-06 | 2.62E-06 | 3.08E-06 |
| 46 | 0.00E+00 | 0.00E+00 | 0.00E+00 | 2.20E-07 | 5.97E-07 | 1.21E-06 | 1.79E-06 | 2.56E-06 | 3.02E-06 |
| 47 | 0.00E+00 | 0.00E+00 | 0.00E+00 | 1.75E-07 | 5.54E-07 | 1.17E-06 | 1.74E-06 | 2.50E-06 | 2.96E-06 |
| 48 | 0.00E+00 | 0.00E+00 | 0.00E+00 | 1.29E-07 | 5.12E-07 | 1.12E-06 | 1.69E-06 | 2.44E-06 | 2.91E-06 |
| 49 | 0.00E+00 | 0.00E+00 | 0.00E+00 | 8.27E-08 | 4.71E-07 | 1.08E-06 | 1.64E-06 | 2.38E-06 | 2.85E-06 |
| 50 | 0.00E+00 | 0.00E+00 | 0.00E+00 | 3.46E-08 | 4.30E-07 | 1.03E-06 | 1.58E-06 | 2.31E-06 | 2.79E-06 |
| 51 | 0.00E+00 | 0.00E+00 | 0.00E+00 | 0.00E+00 | 3.90E-07 | 9.85E-07 | 1.53E-06 | 2.25E-06 | 2.73E-06 |
| 52 | 0.00E+00 | 0.00E+00 | 0.00E+00 | 0.00E+00 | 3.48E-07 | 9.35E-07 | 1.48E-06 | 2.20E-06 | 2.67E-06 |
| 53 | 0.00E+00 | 0.00E+00 | 0.00E+00 | 0.00E+00 | 3.06E-07 | 8.87E-07 | 1.44E-06 | 2.16E-06 | 2.61E-06 |
| 54 | 0.00E+00 | 0.00E+00 | 0.00E+00 | 0.00E+00 | 2.64E-07 | 8.42E-07 | 1.40E-06 | 2.11E-06 | 2.56E-06 |
| 55 | 0.00E+00 | 0.00E+00 | 0.00E+00 | 0.00E+00 | 2.24E-07 | 7.99E-07 | 1.36E-06 | 2.06E-06 | 2.51E-06 |
| 56 | 0.00E+00 | 0.00E+00 | 0.00E+00 | 0.00E+00 | 1.83E-07 | 7.62E-07 | 1.31E-06 | 2.00E-06 | 2.46E-06 |
| 57 | 0.00E+00 | 0.00E+00 | 0.00E+00 | 0.00E+00 | 1.44E-07 | 7.25E-07 | 1.26E-06 | 1.94E-06 | 2.41E-06 |
| 58 | 0.00E+00 | 0.00E+00 | 0.00E+00 | 0.00E+00 | 1.03E-07 | 6.93E-07 | 1.18E-06 | 1.86E-06 | 2.35E-06 |
| 59 | 0.00E+00 | 0.00E+00 | 0.00E+00 | 0.00E+00 | 6.53E-08 | 7.97E-07 | 2.10E-06 | 4.54E-06 | 6.14E-06 |
| 60 | 0.00E+00 | 0.00E+00 | 0.00E+00 | 0.00E+00 | 2.53E-08 | 8.87E-07 | 2.79E-06 | 6.69E-06 | 9.29E-06 |
| 61 | 0.00E+00 | 0.00E+00 | 0.00E+00 | 0.00E+00 | 0.00E+00 | 5.75E-07 | 1.03E-06 | 1.75E-06 | 2.24E-06 |
| 62 | 0.00E+00 | 0.00E+00 | 0.00E+00 | 0.00E+00 | 0.00E+00 | 5.61E-07 | 1.12E-06 | 1.83E-06 | 2.42E-06 |
| 63 | 0.00E+00 | 0.00E+00 | 0.00E+00 | 0.00E+00 | 0.00E+00 | 5.60E-07 | 9.79E-07 | 1.53E-06 | 1.98E-06 |
| 64 | 0.00E+00 | 0.00E+00 | 0.00E+00 | 0.00E+00 | 0.00E+00 | 4.85E-07 | 9.62E-07 | 1.46E-06 | 1.92E-06 |
| 65 | 0.00E+00 | 0.00E+00 | 0.00E+00 | 0.00E+00 | 0.00E+00 | 4.53E-07 | 9.21E-07 | 1.47E-06 | 1.91E-06 |
| 66 | 0.00E+00 | 0.00E+00 | 0.00E+00 | 0.00E+00 | 0.00E+00 | 4.26E-07 | 8.67E-07 | 1.44E-06 | 1.88E-06 |
| 67 | 0.00E+00 | 0.00E+00 | 0.00E+00 | 0.00E+00 | 0.00E+00 | 4.06E-07 | 8.01E-07 | 1.39E-06 | 1.84E-06 |
| 68 | 0.00E+00 | 0.00E+00 | 0.00E+00 | 0.00E+00 | 0.00E+00 | 4.60E-07 | 1.43E-06 | 3.08E-06 | 4.36E-06 |
| 69 | 0.00E+00 | 0.00E+00 | 0.00E+00 | 0.00E+00 | 0.00E+00 | 3.22E-07 | 7.62E-07 | 1.36E-06 | 1.67E-06 |
| 70 | 0.00E+00 | 0.00E+00 | 0.00E+00 | 0.00E+00 | 0.00E+00 | 3.12E-07 | 8.96E-07 | 1.75E-06 | 2.33E-06 |
| 71 | 0.00E+00 | 0.00E+00 | 0.00E+00 | 0.00E+00 | 0.00E+00 | 2.64E-07 | 7.08E-07 | 1.18E-06 | 1.39E-06 |
| 72 | 0.00E+00 | 0.00E+00 | 0.00E+00 | 0.00E+00 | 0.00E+00 | 2.37E-07 | 6.30E-07 | 1.16E-06 | 1.40E-06 |
| 73 | 0.00E+00 | 0.00E+00 | 0.00E+00 | 0.00E+00 | 0.00E+00 | 2.05E-07 | 6.00E-07 | 1.11E-06 | 1.37E-06 |
| 74 | 0.00E+00 | 0.00E+00 | 0.00E+00 | 0.00E+00 | 0.00E+00 | 1.77E-07 | 5.73E-07 | 1.08E-06 | 1.34E-06 |
| 75 | 0.00E+00 | 0.00E+00 | 0.00E+00 | 0.00E+00 | 0.00E+00 | 1.48E-07 | 5.48E-07 | 1.05E-06 | 1.30E-06 |
| 76 | 0.00E+00 | 0.00E+00 | 0.00E+00 | 0.00E+00 | 0.00E+00 | 1.21E-07 | 5.25E-07 | 1.03E-06 | 1.27E-06 |
| 77 | 0.00E+00 | 0.00E+00 | 0.00E+00 | 0.00E+00 | 0.00E+00 | 9.48E-08 | 5.05E-07 | 9.96E-07 | 1.24E-06 |
| 78 | 0.00E+00 | 0.00E+00 | 0.00E+00 | 0.00E+00 | 0.00E+00 | 6.83E-08 | 4.85E-07 | 9.65E-07 | 1.22E-06 |
| 79 | 0.00E+00 | 0.00E+00 | 0.00E+00 | 0.00E+00 | 0.00E+00 | 4.16E-08 | 4.66E-07 | 9.36E-07 | 1.19E-06 |
| 80 | 0.00E+00 | 0.00E+00 | 0.00E+00 | 0.00E+00 | 0.00E+00 | 1.44E-08 | 4.43E-07 | 9.01E-07 | 1.17E-06 |
| 81 | 0.00E+00 | 0.00E+00 | 0.00E+00 | 0.00E+00 | 0.00E+00 | 0.00E+00 | 4.20E-07 | 8.67E-07 | 1.15E-06 |
| 82 | 0.00E+00 | 0.00E+00 | 0.00E+00 | 0.00E+00 | 0.00E+00 | 0.00E+00 | 3.96E-07 | 8.38E-07 | 1.12E-06 |
| 83 | 0.00E+00 | 0.00E+00 | 0.00E+00 | 0.00E+00 | 0.00E+00 | 0.00E+00 | 3.72E-07 | 8.09E-07 | 1.10E-06 |
| 84 | 0.00E+00 | 0.00E+00 | 0.00E+00 | 0.00E+00 | 0.00E+00 | 0.00E+00 | 3.48E-07 | 7.80E-07 | 1.07E-06 |
| 85 | 0.00E+00 | 0.00E+00 | 0.00E+00 | 0.00E+00 | 0.00E+00 | 0.00E+00 | 3.26E-07 | 7.57E-07 | 1.05E-06 |
| 86 | 0.00E+00 | 0.00E+00 | 0.00E+00 | 0.00E+00 | 0.00E+00 | 0.00E+00 | 3.04E-07 | 7.36E-07 | 1.02E-06 |
| 87 | 0.00E+00 | 0.00E+00 | 0.00E+00 | 0.00E+00 | 0.00E+00 | 0.00E+00 | 2.80E-07 | 7.16E-07 | 9.99E-07 |
| 88 | 0.00E+00 | 0.00E+00 | 0.00E+00 | 0.00E+00 | 0.00E+00 | 0.00E+00 | 2.57E-07 | 6.97E-07 | 9.74E-07 |
| 89 | 0.00E+00 | 0.00E+00 | 0.00E+00 | 0.00E+00 | 0.00E+00 | 0.00E+00 | 2.33E-07 | 6.78E-07 | 9.51E-07 |
| 90 | 0.00E+00 | 0.00E+00 | 0.00E+00 | 0.00E+00 | 0.00E+00 | 0.00E+00 | 2.09E-07 | 6.59E-07 | 9.29E-07 |
| 91 | 0.00E+00 | 0.00E+00 | 0.00E+00 | 0.00E+00 | 0.00E+00 | 0.00E+00 | 1.88E-07 | 6.40E-07 | 9.08E-07 |
| 92 | 0.00E+00 | 0.00E+00 | 0.00E+00 | 0.00E+00 | 0.00E+00 | 0.00E+00 | 1.68E-07 | 6.19E-07 | 8.85E-07 |
| 93 | 0.00E+00 | 0.00E+00 | 0.00E+00 | 0.00E+00 | 0.00E+00 | 0.00E+00 | 1.50E-07 | 6.00E-07 | 8.61E-07 |
| 94 | 0.00E+00 | 0.00E+00 | 0.00E+00 | 0.00E+00 | 0.00E+00 | 0.00E+00 | 1.31E-07 | 5.80E-07 | 8.35E-07 |
| 95 | 0.00E+00 | 0.00E+00 | 0.00E+00 | 0.00E+00 | 0.00E+00 | 0.00E+00 | 1.12E-07 | 5.58E-07 | 8.08E-07 |
| 96 | 0.00E+00 | 0.00E+00 | 0.00E+00 | 0.00E+00 | 0.00E+00 | 0.00E+00 | 9.18E-08 | 5.34E-07 | 7.81E-07 |
| 97 | 0.00E+00 | 0.00E+00 | 0.00E+00 | 0.00E+00 | 0.00E+00 | 0.00E+00 | 7.11E-08 | 5.11E-07 | 7.57E-07 |
| 98 | 0.00E+00 | 0.00E+00 | 0.00E+00 | 0.00E+00 | 0.00E+00 | 0.00E+00 | 5.04E-08 | 4.89E-07 | 7.34E-07 |
| 99 | 0.00E+00 | 0.00E+00 | 0.00E+00 | 0.00E+00 | 0.00E+00 | 0.00E+00 | 2.96E-08 | 4.69E-07 | 7.14E-07 |
| 100 | 0.00E+00 | 0.00E+00 | 0.00E+00 | 0.00E+00 | 0.00E+00 | 0.00E+00 | 1.17E-08 | 4.52E-07 | 6.98E-07 |
| 101 | 0.00E+00 | 0.00E+00 | 0.00E+00 | 0.00E+00 | 0.00E+00 | 0.00E+00 | 0.00E+00 | 4.38E-07 | 6.82E-07 |
| 102 | 0.00E+00 | 0.00E+00 | 0.00E+00 | 0.00E+00 | 0.00E+00 | 0.00E+00 | 0.00E+00 | 4.25E-07 | 6.65E-07 |
| 103 | 0.00E+00 | 0.00E+00 | 0.00E+00 | 0.00E+00 | 0.00E+00 | 0.00E+00 | 0.00E+00 | 4.08E-07 | 6.48E-07 |
| 104 | 0.00E+00 | 0.00E+00 | 0.00E+00 | 0.00E+00 | 0.00E+00 | 0.00E+00 | 0.00E+00 | 3.91E-07 | 6.30E-07 |
| 105 | 0.00E+00 | 0.00E+00 | 0.00E+00 | 0.00E+00 | 0.00E+00 | 0.00E+00 | 0.00E+00 | 3.72E-07 | 6.10E-07 |
| 106 | 0.00E+00 | 0.00E+00 | 0.00E+00 | 0.00E+00 | 0.00E+00 | 0.00E+00 | 0.00E+00 | 3.52E-07 | 5.92E-07 |
| 107 | 0.00E+00 | 0.00E+00 | 0.00E+00 | 0.00E+00 | 0.00E+00 | 0.00E+00 | 0.00E+00 | 3.32E-07 | 5.75E-07 |
| 108 | 0.00E+00 | 0.00E+00 | 0.00E+00 | 0.00E+00 | 0.00E+00 | 0.00E+00 | 0.00E+00 | 3.19E-07 | 5.59E-07 |
| 109 | 0.00E+00 | 0.00E+00 | 0.00E+00 | 0.00E+00 | 0.00E+00 | 0.00E+00 | 0.00E+00 | 3.04E-07 | 5.45E-07 |
| 110 | 0.00E+00 | 0.00E+00 | 0.00E+00 | 0.00E+00 | 0.00E+00 | 0.00E+00 | 0.00E+00 | 2.93E-07 | 5.32E-07 |
| 111 | 0.00E+00 | 0.00E+00 | 0.00E+00 | 0.00E+00 | 0.00E+00 | 0.00E+00 | 0.00E+00 | 2.79E-07 | 5.18E-07 |
| 112 | 0.00E+00 | 0.00E+00 | 0.00E+00 | 0.00E+00 | 0.00E+00 | 0.00E+00 | 0.00E+00 | 2.65E-07 | 5.04E-07 |
| 113 | 0.00E+00 | 0.00E+00 | 0.00E+00 | 0.00E+00 | 0.00E+00 | 0.00E+00 | 0.00E+00 | 2.45E-07 | 4.87E-07 |
| 114 | 0.00E+00 | 0.00E+00 | 0.00E+00 | 0.00E+00 | 0.00E+00 | 0.00E+00 | 0.00E+00 | 2.26E-07 | 4.68E-07 |
| 115 | 0.00E+00 | 0.00E+00 | 0.00E+00 | 0.00E+00 | 0.00E+00 | 0.00E+00 | 0.00E+00 | 2.04E-07 | 4.49E-07 |
| 116 | 0.00E+00 | 0.00E+00 | 0.00E+00 | 0.00E+00 | 0.00E+00 | 0.00E+00 | 0.00E+00 | 1.86E-07 | 4.29E-07 |
| 117 | 0.00E+00 | 0.00E+00 | 0.00E+00 | 0.00E+00 | 0.00E+00 | 0.00E+00 | 0.00E+00 | 1.69E-07 | 4.09E-07 |
| 118 | 0.00E+00 | 0.00E+00 | 0.00E+00 | 0.00E+00 | 0.00E+00 | 0.00E+00 | 0.00E+00 | 1.55E-07 | 3.91E-07 |
| 119 | 0.00E+00 | 0.00E+00 | 0.00E+00 | 0.00E+00 | 0.00E+00 | 0.00E+00 | 0.00E+00 | 1.43E-07 | 3.75E-07 |
| 120 | 0.00E+00 | 0.00E+00 | 0.00E+00 | 0.00E+00 | 0.00E+00 | 0.00E+00 | 0.00E+00 | 1.32E-07 | 3.61E-07 |
| 121 | 0.00E+00 | 0.00E+00 | 0.00E+00 | 0.00E+00 | 0.00E+00 | 0.00E+00 | 0.00E+00 | 1.21E-07 | 3.47E-07 |
| 122 | 0.00E+00 | 0.00E+00 | 0.00E+00 | 0.00E+00 | 0.00E+00 | 0.00E+00 | 0.00E+00 | 1.08E-07 | 3.35E-07 |
| 123 | 0.00E+00 | 0.00E+00 | 0.00E+00 | 0.00E+00 | 0.00E+00 | 0.00E+00 | 0.00E+00 | 9.49E-08 | 3.23E-07 |
| 124 | 0.00E+00 | 0.00E+00 | 0.00E+00 | 0.00E+00 | 0.00E+00 | 0.00E+00 | 0.00E+00 | 8.11E-08 | 3.10E-07 |
| 125 | 0.00E+00 | 0.00E+00 | 0.00E+00 | 0.00E+00 | 0.00E+00 | 0.00E+00 | 0.00E+00 | 6.77E-08 | 2.98E-07 |
| 126 | 0.00E+00 | 0.00E+00 | 0.00E+00 | 0.00E+00 | 0.00E+00 | 0.00E+00 | 0.00E+00 | 5.42E-08 | 2.85E-07 |
| 127 | 0.00E+00 | 0.00E+00 | 0.00E+00 | 0.00E+00 | 0.00E+00 | 0.00E+00 | 0.00E+00 | 4.19E-08 | 2.71E-07 |
| 128 | 0.00E+00 | 0.00E+00 | 0.00E+00 | 0.00E+00 | 0.00E+00 | 0.00E+00 | 0.00E+00 | 2.97E-08 | 2.57E-07 |
| 129 | 0.00E+00 | 0.00E+00 | 0.00E+00 | 0.00E+00 | 0.00E+00 | 0.00E+00 | 0.00E+00 | 1.76E-08 | 2.42E-07 |
| 130 | 0.00E+00 | 0.00E+00 | 0.00E+00 | 0.00E+00 | 0.00E+00 | 0.00E+00 | 0.00E+00 | 4.44E-09 | 2.26E-07 |
| 131 | 0.00E+00 | 0.00E+00 | 0.00E+00 | 0.00E+00 | 0.00E+00 | 0.00E+00 | 0.00E+00 | 0.00E+00 | 2.12E-07 |
| 132 | 0.00E+00 | 0.00E+00 | 0.00E+00 | 0.00E+00 | 0.00E+00 | 0.00E+00 | 0.00E+00 | 0.00E+00 | 1.98E-07 |
| 133 | 0.00E+00 | 0.00E+00 | 0.00E+00 | 0.00E+00 | 0.00E+00 | 0.00E+00 | 0.00E+00 | 0.00E+00 | 1.86E-07 |
| 134 | 0.00E+00 | 0.00E+00 | 0.00E+00 | 0.00E+00 | 0.00E+00 | 0.00E+00 | 0.00E+00 | 0.00E+00 | 1.74E-07 |
| 135 | 0.00E+00 | 0.00E+00 | 0.00E+00 | 0.00E+00 | 0.00E+00 | 0.00E+00 | 0.00E+00 | 0.00E+00 | 1.63E-07 |
| 136 | 0.00E+00 | 0.00E+00 | 0.00E+00 | 0.00E+00 | 0.00E+00 | 0.00E+00 | 0.00E+00 | 0.00E+00 | 1.52E-07 |
| 137 | 0.00E+00 | 0.00E+00 | 0.00E+00 | 0.00E+00 | 0.00E+00 | 0.00E+00 | 0.00E+00 | 0.00E+00 | 1.40E-07 |
| 138 | 0.00E+00 | 0.00E+00 | 0.00E+00 | 0.00E+00 | 0.00E+00 | 0.00E+00 | 0.00E+00 | 0.00E+00 | 1.28E-07 |
| 139 | 0.00E+00 | 0.00E+00 | 0.00E+00 | 0.00E+00 | 0.00E+00 | 0.00E+00 | 0.00E+00 | 0.00E+00 | 1.17E-07 |
| 140 | 0.00E+00 | 0.00E+00 | 0.00E+00 | 0.00E+00 | 0.00E+00 | 0.00E+00 | 0.00E+00 | 0.00E+00 | 1.06E-07 |
| 141 | 0.00E+00 | 0.00E+00 | 0.00E+00 | 0.00E+00 | 0.00E+00 | 0.00E+00 | 0.00E+00 | 0.00E+00 | 9.51E-08 |
| 142 | 0.00E+00 | 0.00E+00 | 0.00E+00 | 0.00E+00 | 0.00E+00 | 0.00E+00 | 0.00E+00 | 0.00E+00 | 8.49E-08 |
| 143 | 0.00E+00 | 0.00E+00 | 0.00E+00 | 0.00E+00 | 0.00E+00 | 0.00E+00 | 0.00E+00 | 0.00E+00 | 7.52E-08 |
| 144 | 0.00E+00 | 0.00E+00 | 0.00E+00 | 0.00E+00 | 0.00E+00 | 0.00E+00 | 0.00E+00 | 0.00E+00 | 6.58E-08 |
| 145 | 0.00E+00 | 0.00E+00 | 0.00E+00 | 0.00E+00 | 0.00E+00 | 0.00E+00 | 0.00E+00 | 0.00E+00 | 5.63E-08 |
| 146 | 0.00E+00 | 0.00E+00 | 0.00E+00 | 0.00E+00 | 0.00E+00 | 0.00E+00 | 0.00E+00 | 0.00E+00 | 4.68E-08 |
| 147 | 0.00E+00 | 0.00E+00 | 0.00E+00 | 0.00E+00 | 0.00E+00 | 0.00E+00 | 0.00E+00 | 0.00E+00 | 3.68E-08 |
| 148 | 0.00E+00 | 0.00E+00 | 0.00E+00 | 0.00E+00 | 0.00E+00 | 0.00E+00 | 0.00E+00 | 0.00E+00 | 2.61E-08 |
| 149 | 0.00E+00 | 0.00E+00 | 0.00E+00 | 0.00E+00 | 0.00E+00 | 0.00E+00 | 0.00E+00 | 0.00E+00 | 1.54E-08 |
| 150 | 0.00E+00 | 0.00E+00 | 0.00E+00 | 0.00E+00 | 0.00E+00 | 0.00E+00 | 0.00E+00 | 0.00E+00 | 3.64E-09 |

Table.12. Spectra predicted by MLP neural network: No filter

| energy bin (kev)/voltage(kv) | 20kv | 30kv | 40kv | 50kv | 60kv | 80kv | 100kv | 130kv | 150kv |
| --- | --- | --- | --- | --- | --- | --- | --- | --- | --- |
| 1 | 0 | 0 | 0 | 0 | 0 | 0 | 0 | 0 | 0 |
| 2 | 1.18E-09 | 2.12E-12 | 1.71E-12 | 1.35E-12 | 1.22E-12 | 1.98E-12 | 4.53E-12 | 7.23E-12 | 5.68E-12 |
| 3 | 2.39E-07 | 3.38E-08 | 2.88E-08 | 2.81E-08 | 2.73E-08 | 2.67E-08 | 2.93E-08 | 3.59E-08 | 4.01E-08 |
| 4 | 1.11E-06 | 7.79E-07 | 6.26E-07 | 5.52E-07 | 5.20E-07 | 5.07E-07 | 5.18E-07 | 5.45E-07 | 5.01E-07 |
| 5 | 1.71E-06 | 2.32E-06 | 2.24E-06 | 2.15E-06 | 2.05E-06 | 1.83E-06 | 1.73E-06 | 1.67E-06 | 1.53E-06 |
| 6 | 2.29E-06 | 3.54E-06 | 4.03E-06 | 3.91E-06 | 3.58E-06 | 3.19E-06 | 3.12E-06 | 3.11E-06 | 3.11E-06 |
| 7 | 2.78E-06 | 4.29E-06 | 5.01E-06 | 5.40E-06 | 5.35E-06 | 4.96E-06 | 5.03E-06 | 4.86E-06 | 4.47E-06 |
| 8 | 2.86E-06 | 4.16E-06 | 5.07E-06 | 5.84E-06 | 6.42E-06 | 6.88E-06 | 6.08E-06 | 3.21E-06 | 1.68E-06 |
| 9 | 2.68E-06 | 5.49E-06 | 7.51E-06 | 9.32E-06 | 1.07E-05 | 1.24E-05 | 1.34E-05 | 1.56E-05 | 1.90E-05 |
| 10 | 2.40E-06 | 4.17E-06 | 5.40E-06 | 6.45E-06 | 7.24E-06 | 8.29E-06 | 9.07E-06 | 9.60E-06 | 8.93E-06 |
| 11 | 2.08E-06 | 3.18E-06 | 4.06E-06 | 4.66E-06 | 5.03E-06 | 5.38E-06 | 5.66E-06 | 5.87E-06 | 5.01E-06 |
| 12 | 1.69E-06 | 2.83E-06 | 3.61E-06 | 4.04E-06 | 4.26E-06 | 4.47E-06 | 4.60E-06 | 4.69E-06 | 3.93E-06 |
| 13 | 1.40E-06 | 2.39E-06 | 3.10E-06 | 3.48E-06 | 3.65E-06 | 3.72E-06 | 3.69E-06 | 4.59E-06 | 6.27E-06 |
| 14 | 1.17E-06 | 2.16E-06 | 2.88E-06 | 3.40E-06 | 3.75E-06 | 4.12E-06 | 4.26E-06 | 4.14E-06 | 3.56E-06 |
| 15 | 9.71E-07 | 2.04E-06 | 2.82E-06 | 3.33E-06 | 3.65E-06 | 4.00E-06 | 4.20E-06 | 4.49E-06 | 3.91E-06 |
| 16 | 7.88E-07 | 1.70E-06 | 2.54E-06 | 3.22E-06 | 3.68E-06 | 4.09E-06 | 4.38E-06 | 4.70E-06 | 4.43E-06 |
| 17 | 6.15E-07 | 1.72E-06 | 2.54E-06 | 3.13E-06 | 3.57E-06 | 4.19E-06 | 4.53E-06 | 4.71E-06 | 4.74E-06 |
| 18 | 4.45E-07 | 1.53E-06 | 2.36E-06 | 2.99E-06 | 3.49E-06 | 4.16E-06 | 4.56E-06 | 5.20E-06 | 4.72E-06 |
| 19 | 2.73E-07 | 1.40E-06 | 2.19E-06 | 2.79E-06 | 3.29E-06 | 4.14E-06 | 4.86E-06 | 5.34E-06 | 4.93E-06 |
| 20 | 1.03E-07 | 1.22E-06 | 2.08E-06 | 2.73E-06 | 3.24E-06 | 4.04E-06 | 4.69E-06 | 5.40E-06 | 5.34E-06 |
| 21 | 4.02E-10 | 1.10E-06 | 1.89E-06 | 2.59E-06 | 3.12E-06 | 3.95E-06 | 4.65E-06 | 5.36E-06 | 5.22E-06 |
| 22 | 1.91E-09 | 9.78E-07 | 1.78E-06 | 2.43E-06 | 2.94E-06 | 3.76E-06 | 4.57E-06 | 5.51E-06 | 5.33E-06 |
| 23 | 1.92E-09 | 8.55E-07 | 1.61E-06 | 2.26E-06 | 2.84E-06 | 3.78E-06 | 4.55E-06 | 5.47E-06 | 5.39E-06 |
| 24 | 7.38E-10 | 8.52E-07 | 1.52E-06 | 2.15E-06 | 2.73E-06 | 3.76E-06 | 4.61E-06 | 5.45E-06 | 5.43E-06 |
| 25 | 1.21E-09 | 6.41E-07 | 1.35E-06 | 2.03E-06 | 2.62E-06 | 3.54E-06 | 4.37E-06 | 5.37E-06 | 5.44E-06 |
| 26 | -7.94E-10 | 5.42E-07 | 1.20E-06 | 1.87E-06 | 2.47E-06 | 3.44E-06 | 4.24E-06 | 5.14E-06 | 5.43E-06 |
| 27 | -1.59E-09 | 5.73E-07 | 1.18E-06 | 1.78E-06 | 2.33E-06 | 3.31E-06 | 4.12E-06 | 5.00E-06 | 5.39E-06 |
| 28 | 1.60E-09 | 3.18E-07 | 9.04E-07 | 1.64E-06 | 2.27E-06 | 3.21E-06 | 4.10E-06 | 5.10E-06 | 5.38E-06 |
| 29 | -7.19E-10 | 1.83E-07 | 8.32E-07 | 1.54E-06 | 2.11E-06 | 3.07E-06 | 3.90E-06 | 5.01E-06 | 5.29E-06 |
| 30 | 5.33E-10 | 1.10E-07 | 5.25E-07 | 1.09E-06 | 1.71E-06 | 2.92E-06 | 3.88E-06 | 4.87E-06 | 5.31E-06 |
| 31 | -1.69E-10 | 2.42E-07 | 8.60E-07 | 1.40E-06 | 1.88E-06 | 2.73E-06 | 3.51E-06 | 4.52E-06 | 5.00E-06 |
| 32 | -4.43E-10 | 7.38E-09 | 6.44E-07 | 1.24E-06 | 1.77E-06 | 2.77E-06 | 3.58E-06 | 4.57E-06 | 4.90E-06 |
| 33 | 3.30E-10 | 1.43E-08 | 5.83E-07 | 1.16E-06 | 1.65E-06 | 2.59E-06 | 3.45E-06 | 4.44E-06 | 4.88E-06 |
| 34 | 1.46E-09 | 1.24E-08 | 4.92E-07 | 1.08E-06 | 1.57E-06 | 2.49E-06 | 3.30E-06 | 4.30E-06 | 4.90E-06 |
| 35 | 1.83E-09 | 8.50E-08 | 3.96E-07 | 9.93E-07 | 1.50E-06 | 2.32E-06 | 3.20E-06 | 4.19E-06 | 4.79E-06 |
| 36 | 5.12E-10 | 4.14E-08 | 3.52E-07 | 9.33E-07 | 1.39E-06 | 2.22E-06 | 3.03E-06 | 4.07E-06 | 4.67E-06 |
| 37 | -1.38E-09 | 2.02E-07 | 5.12E-07 | 9.01E-07 | 1.31E-06 | 2.10E-06 | 2.97E-06 | 4.00E-06 | 4.29E-06 |
| 38 | -6.85E-10 | -3.54E-09 | 1.98E-07 | 6.89E-07 | 1.23E-06 | 2.01E-06 | 2.82E-06 | 3.85E-06 | 4.42E-06 |
| 39 | 7.10E-10 | -3.77E-08 | 1.14E-07 | 7.08E-07 | 1.15E-06 | 1.94E-06 | 2.64E-06 | 3.75E-06 | 4.30E-06 |
| 40 | 1.64E-09 | -5.91E-09 | 6.04E-08 | 5.77E-07 | 1.07E-06 | 1.85E-06 | 2.63E-06 | 3.65E-06 | 4.18E-06 |
| 41 | 9.92E-10 | -1.83E-08 | 4.05E-09 | 4.83E-07 | 1.01E-06 | 1.80E-06 | 2.52E-06 | 3.54E-06 | 4.08E-06 |
| 42 | -1.89E-09 | 3.36E-09 | 1.80E-08 | 4.91E-07 | 9.42E-07 | 1.71E-06 | 2.43E-06 | 3.37E-06 | 3.85E-06 |
| 43 | 1.67E-09 | -3.80E-08 | 9.52E-09 | 4.33E-07 | 8.92E-07 | 1.65E-06 | 2.36E-06 | 3.30E-06 | 3.83E-06 |
| 44 | -1.39E-09 | 3.16E-09 | -2.29E-09 | 3.78E-07 | 8.22E-07 | 1.61E-06 | 2.27E-06 | 3.14E-06 | 3.76E-06 |
| 45 | 6.78E-10 | -1.72E-08 | 2.19E-09 | 3.18E-07 | 7.47E-07 | 1.49E-06 | 2.18E-06 | 3.12E-06 | 3.66E-06 |
| 46 | 2.29E-10 | -2.33E-09 | 3.51E-09 | 2.57E-07 | 6.94E-07 | 1.43E-06 | 2.12E-06 | 3.07E-06 | 3.56E-06 |
| 47 | 1.91E-09 | -4.13E-09 | 5.84E-09 | 2.08E-07 | 6.44E-07 | 1.36E-06 | 2.05E-06 | 2.94E-06 | 3.47E-06 |
| 48 | -1.87E-09 | -1.63E-09 | 7.30E-10 | 1.52E-07 | 5.88E-07 | 1.30E-06 | 1.98E-06 | 2.85E-06 | 3.37E-06 |
| 49 | 9.81E-10 | -8.42E-08 | 5.00E-08 | 2.71E-07 | 5.42E-07 | 1.25E-06 | 1.87E-06 | 2.75E-06 | 3.29E-06 |
| 50 | -1.38E-09 | -4.39E-10 | -4.91E-10 | 3.97E-08 | 4.81E-07 | 1.19E-06 | 1.81E-06 | 2.68E-06 | 3.21E-06 |
| 51 | -1.47E-09 | 4.29E-09 | -2.61E-08 | 3.19E-08 | 4.32E-07 | 1.15E-06 | 1.70E-06 | 2.52E-06 | 3.01E-06 |
| 52 | -1.66E-09 | 2.12E-08 | 5.97E-08 | 1.51E-07 | 3.90E-07 | 1.17E-06 | 1.70E-06 | 2.49E-06 | 3.03E-06 |
| 53 | -1.22E-09 | -1.58E-08 | 1.43E-08 | 9.62E-08 | 2.82E-07 | 1.00E-06 | 1.48E-06 | 2.44E-06 | 2.98E-06 |
| 54 | -7.34E-10 | 1.01E-08 | -1.78E-08 | 3.43E-08 | 2.61E-07 | 9.59E-07 | 1.58E-06 | 2.38E-06 | 2.87E-06 |
| 55 | 2.36E-11 | -9.68E-09 | -4.23E-08 | 5.61E-10 | 2.52E-07 | 8.99E-07 | 1.52E-06 | 2.34E-06 | 2.81E-06 |
| 56 | -7.55E-10 | 2.32E-08 | -2.84E-08 | 2.18E-08 | 2.24E-07 | 8.33E-07 | 1.46E-06 | 2.29E-06 | 2.75E-06 |
| 57 | 9.71E-10 | 6.37E-09 | -7.46E-09 | 4.15E-09 | 1.55E-07 | 8.10E-07 | 1.42E-06 | 2.18E-06 | 2.71E-06 |
| 58 | 1.96E-09 | 2.77E-09 | -2.39E-10 | 1.87E-08 | 1.62E-07 | 7.68E-07 | 1.31E-06 | 2.08E-06 | 2.59E-06 |
| 59 | 8.46E-10 | -6.76E-10 | 3.61E-10 | -2.66E-09 | 7.41E-08 | 8.85E-07 | 2.34E-06 | 5.05E-06 | 6.83E-06 |
| 60 | 9.14E-10 | 1.17E-08 | 1.10E-08 | -7.28E-09 | 5.90E-08 | 9.83E-07 | 3.09E-06 | 7.43E-06 | 1.03E-05 |
| 61 | 1.56E-09 | 1.23E-08 | 1.76E-08 | -6.86E-09 | 3.96E-08 | 5.93E-07 | 1.14E-06 | 1.94E-06 | 2.48E-06 |
| 62 | 1.35E-09 | 3.30E-09 | -2.19E-09 | 3.73E-10 | 2.22E-07 | 6.20E-07 | 1.24E-06 | 2.05E-06 | 2.67E-06 |
| 63 | -3.20E-10 | 9.96E-10 | 2.83E-09 | -2.16E-09 | -4.86E-10 | 6.01E-07 | 1.08E-06 | 1.69E-06 | 2.18E-06 |
| 64 | -1.64E-09 | 6.45E-10 | 8.09E-10 | -1.08E-09 | 5.92E-10 | 5.35E-07 | 1.06E-06 | 1.61E-06 | 2.12E-06 |
| 65 | -4.26E-10 | -2.96E-09 | -1.22E-09 | -4.57E-10 | 2.93E-10 | 4.99E-07 | 1.02E-06 | 1.63E-06 | 2.10E-06 |
| 66 | -6.59E-11 | 2.18E-08 | 7.79E-11 | -2.43E-08 | 1.84E-08 | 3.70E-07 | 8.81E-07 | 1.60E-06 | 2.13E-06 |
| 67 | -1.78E-09 | -1.02E-09 | -5.39E-10 | 1.80E-10 | 4.80E-10 | 4.40E-07 | 8.79E-07 | 1.53E-06 | 2.02E-06 |
| 68 | -3.51E-10 | -1.37E-09 | -3.72E-09 | -3.66E-09 | 2.92E-09 | 4.99E-07 | 1.57E-06 | 3.37E-06 | 4.78E-06 |
| 69 | 5.97E-10 | 2.80E-10 | 1.77E-09 | -2.61E-09 | 1.26E-09 | 3.38E-07 | 8.04E-07 | 1.43E-06 | 1.77E-06 |
| 70 | 1.63E-09 | 3.09E-09 | -3.16E-09 | 1.14E-09 | 7.03E-08 | 4.09E-07 | 9.79E-07 | 1.91E-06 | 2.20E-06 |
| 71 | 2.30E-10 | -1.31E-09 | -9.55E-10 | -1.43E-10 | 4.11E-09 | 2.87E-07 | 7.69E-07 | 1.29E-06 | 1.52E-06 |
| 72 | -1.79E-10 | -2.74E-10 | -1.79E-10 | 6.92E-10 | 9.32E-09 | 2.58E-07 | 6.56E-07 | 1.26E-06 | 1.53E-06 |
| 73 | -1.77E-09 | 4.42E-09 | 5.31E-09 | -6.70E-10 | 8.73E-09 | 2.20E-07 | 6.41E-07 | 1.19E-06 | 1.48E-06 |
| 74 | 4.82E-10 | 9.53E-09 | -6.99E-10 | -1.18E-08 | 3.68E-10 | 2.18E-07 | 6.17E-07 | 1.18E-06 | 1.45E-06 |
| 75 | 1.66E-09 | 8.33E-10 | 1.27E-09 | 1.72E-10 | 4.18E-09 | 1.59E-07 | 5.96E-07 | 1.21E-06 | 1.41E-06 |
| 76 | -1.70E-09 | 2.18E-10 | 8.51E-10 | -7.44E-10 | -1.52E-09 | 1.33E-07 | 5.65E-07 | 1.11E-06 | 1.38E-06 |
| 77 | -1.14E-10 | 2.59E-09 | 6.97E-10 | -9.33E-10 | 2.59E-10 | 1.12E-07 | 5.45E-07 | 1.09E-06 | 1.37E-06 |
| 78 | -5.58E-10 | 2.64E-11 | 2.96E-10 | -5.03E-11 | 4.65E-10 | 7.70E-08 | 5.24E-07 | 1.04E-06 | 1.31E-06 |
| 79 | -7.78E-10 | 4.61E-10 | 2.28E-10 | -7.96E-10 | -1.78E-09 | 4.48E-08 | 5.01E-07 | 1.01E-06 | 1.29E-06 |
| 80 | -1.30E-09 | -2.04E-10 | 2.31E-09 | 4.07E-09 | -2.87E-09 | 1.52E-08 | 4.79E-07 | 9.68E-07 | 1.26E-06 |
| 81 | -5.97E-10 | -3.84E-11 | -4.32E-11 | -5.45E-11 | -5.81E-11 | 6.58E-10 | 4.52E-07 | 9.43E-07 | 1.24E-06 |
| 82 | 1.77E-09 | 2.33E-10 | 2.43E-10 | 1.41E-10 | -2.92E-10 | 9.62E-10 | 4.20E-07 | 8.82E-07 | 1.21E-06 |
| 83 | -1.93E-09 | -1.22E-10 | -5.06E-11 | -3.47E-11 | -4.07E-10 | 5.67E-10 | 3.99E-07 | 8.65E-07 | 1.16E-06 |
| 84 | -6.04E-10 | -3.11E-08 | -2.38E-08 | -9.31E-09 | 1.65E-08 | 1.25E-07 | 3.44E-07 | 8.48E-07 | 1.13E-06 |
| 85 | -1.07E-09 | -5.44E-11 | -5.24E-11 | -1.15E-10 | -2.16E-10 | 7.10E-10 | 3.49E-07 | 8.09E-07 | 1.13E-06 |
| 86 | 2.57E-10 | -1.25E-09 | 4.09E-09 | 1.77E-08 | 4.11E-08 | 1.32E-07 | 3.16E-07 | 7.84E-07 | 1.10E-06 |
| 87 | 4.72E-10 | -7.55E-11 | 7.57E-11 | 2.81E-10 | 1.08E-10 | -3.43E-11 | 3.00E-07 | 7.67E-07 | 1.07E-06 |
| 88 | -1.56E-09 | 8.73E-10 | -2.07E-09 | 1.42E-09 | -1.73E-10 | 3.91E-09 | 2.76E-07 | 7.47E-07 | 1.04E-06 |
| 89 | 2.56E-10 | -7.92E-11 | -7.85E-11 | -2.25E-11 | -1.89E-11 | 1.99E-10 | 2.48E-07 | 7.33E-07 | 1.04E-06 |
| 90 | 1.02E-09 | 1.02E-11 | 7.95E-11 | 1.07E-10 | -1.60E-10 | 1.72E-11 | 2.21E-07 | 7.06E-07 | 9.96E-07 |
| 91 | -6.69E-10 | 1.18E-10 | -1.85E-10 | -5.17E-10 | -1.18E-09 | 2.81E-10 | 2.00E-07 | 6.82E-07 | 9.69E-07 |
| 92 | 1.11E-09 | -4.51E-11 | -6.97E-11 | -9.96E-11 | -1.29E-10 | 6.07E-11 | 1.78E-07 | 6.64E-07 | 9.50E-07 |
| 93 | 1.49E-09 | 1.70E-11 | -1.50E-12 | 9.79E-11 | -1.28E-10 | 7.08E-11 | 1.61E-07 | 6.42E-07 | 9.19E-07 |
| 94 | -1.59E-09 | 5.57E-11 | 1.50E-10 | 2.13E-10 | -5.25E-10 | 2.90E-10 | 1.40E-07 | 6.19E-07 | 8.95E-07 |
| 95 | 5.57E-10 | 3.21E-10 | 1.95E-10 | 1.49E-10 | -2.59E-10 | 3.16E-10 | 1.23E-07 | 6.01E-07 | 8.65E-07 |
| 96 | -7.07E-10 | -5.61E-10 | -9.74E-10 | -1.26E-09 | -4.93E-10 | 1.84E-08 | 1.13E-07 | 5.28E-07 | 7.83E-07 |
| 97 | -1.92E-09 | -8.55E-10 | 3.23E-10 | 9.98E-10 | 5.86E-10 | 2.70E-09 | 8.22E-08 | 5.45E-07 | 8.00E-07 |
| 98 | 6.62E-10 | 2.14E-11 | 1.17E-10 | 1.33E-10 | -9.87E-11 | 4.66E-11 | 5.28E-08 | 5.11E-07 | 7.56E-07 |
| 99 | 1.48E-09 | -1.71E-10 | 2.88E-10 | 1.75E-10 | -3.53E-10 | 1.10E-10 | 2.94E-08 | 4.96E-07 | 7.60E-07 |
| 100 | -9.89E-11 | -3.74E-10 | 6.97E-12 | 1.48E-10 | 5.90E-13 | -2.02E-11 | 1.33E-08 | 4.81E-07 | 7.40E-07 |
| 101 | -1.44E-10 | -4.17E-10 | -2.62E-10 | -1.83E-10 | -1.69E-10 | -2.33E-10 | 1.51E-09 | 4.63E-07 | 7.26E-07 |
| 102 | 1.68E-09 | -1.52E-11 | -9.71E-12 | 2.62E-12 | 2.76E-11 | 6.07E-12 | 4.86E-11 | 4.50E-07 | 7.13E-07 |
| 103 | 1.07E-10 | -4.42E-11 | -6.48E-12 | 5.13E-11 | 1.09E-10 | -8.81E-11 | 6.33E-11 | 4.40E-07 | 7.01E-07 |
| 104 | 7.22E-10 | 1.33E-11 | 2.53E-12 | -4.97E-12 | -3.92E-11 | -6.91E-10 | 4.99E-12 | 4.23E-07 | 6.69E-07 |
| 105 | -1.25E-09 | 1.29E-11 | 4.64E-11 | 5.24E-11 | 3.72E-11 | -5.51E-11 | 7.62E-10 | 3.90E-07 | 6.50E-07 |
| 106 | -1.46E-09 | 1.63E-11 | -1.03E-10 | -8.87E-11 | -4.89E-12 | -4.59E-11 | 1.29E-11 | 3.76E-07 | 6.28E-07 |
| 107 | -2.98E-10 | 6.18E-11 | 1.67E-11 | -7.51E-12 | -1.85E-11 | -5.13E-11 | 1.69E-11 | 3.51E-07 | 6.07E-07 |
| 108 | 1.48E-09 | 1.30E-11 | -6.42E-12 | -2.37E-11 | -4.07E-11 | -1.05E-10 | -2.71E-10 | 3.33E-07 | 5.93E-07 |
| 109 | 1.24E-09 | -7.02E-11 | 1.42E-11 | -2.49E-11 | -1.37E-10 | -4.00E-10 | 4.68E-10 | 3.19E-07 | 5.78E-07 |
| 110 | 1.87E-09 | -2.73E-11 | -1.04E-11 | 1.82E-11 | 5.71E-11 | -2.81E-11 | 3.18E-11 | 3.19E-07 | 5.65E-07 |
| 111 | -1.10E-09 | 5.68E-11 | 5.34E-11 | 4.02E-11 | 1.82E-11 | 7.47E-12 | 9.46E-10 | 2.97E-07 | 5.52E-07 |
| 112 | 1.30E-10 | 1.42E-11 | -6.49E-12 | -1.30E-11 | -1.07E-11 | 3.31E-12 | 2.75E-10 | 2.82E-07 | 5.37E-07 |
| 113 | -4.54E-10 | 3.71E-10 | 5.80E-10 | 6.03E-10 | 5.44E-10 | 1.33E-10 | 5.28E-10 | 2.49E-07 | 4.89E-07 |
| 114 | -1.14E-09 | 1.20E-10 | 1.28E-10 | 1.46E-10 | 1.82E-10 | 3.51E-10 | 1.15E-09 | 2.34E-07 | 4.98E-07 |
| 115 | 1.08E-09 | -4.09E-11 | -2.82E-11 | -2.13E-11 | -1.61E-11 | 8.43E-13 | 3.35E-10 | 2.13E-07 | 4.76E-07 |
| 116 | 2.00E-09 | 1.66E-11 | 4.57E-11 | -7.60E-12 | -1.93E-10 | -3.92E-10 | -1.05E-11 | 1.94E-07 | 4.46E-07 |
| 117 | -9.98E-10 | 6.20E-10 | -1.72E-12 | -3.13E-10 | 6.60E-11 | -4.72E-09 | 5.92E-11 | 1.87E-07 | 4.32E-07 |
| 118 | -1.73E-09 | 3.49E-11 | -1.22E-10 | -1.56E-10 | -1.28E-10 | -2.64E-10 | 1.81E-11 | 1.63E-07 | 4.12E-07 |
| 119 | -3.91E-10 | 1.64E-11 | 5.48E-12 | -4.03E-11 | -1.42E-10 | -5.47E-10 | 1.27E-11 | 1.50E-07 | 3.95E-07 |
| 120 | 9.49E-10 | -1.76E-12 | -7.71E-12 | -6.24E-12 | 8.39E-12 | 1.43E-10 | 8.78E-10 | 1.41E-07 | 3.82E-07 |
| 121 | 1.17E-09 | -9.94E-11 | -1.84E-10 | -8.02E-11 | 1.97E-10 | 7.91E-11 | 2.20E-10 | 1.30E-07 | 3.69E-07 |
| 122 | 7.19E-10 | -3.49E-12 | -2.13E-11 | -3.80E-12 | 1.58E-10 | -1.04E-10 | 3.64E-10 | 1.16E-07 | 3.52E-07 |
| 123 | 9.27E-10 | -2.57E-11 | -6.86E-11 | -7.97E-11 | -8.14E-11 | -3.89E-12 | 1.79E-09 | 1.02E-07 | 3.41E-07 |
| 124 | -1.03E-09 | -5.43E-11 | 2.80E-11 | 1.14E-10 | 1.78E-10 | 1.36E-10 | 3.63E-11 | 8.57E-08 | 3.29E-07 |
| 125 | 1.71E-09 | 7.61E-13 | 6.62E-12 | -3.58E-12 | -2.62E-11 | -1.90E-10 | -1.02E-09 | 6.90E-08 | 3.17E-07 |
| 126 | 6.95E-11 | -8.81E-11 | 9.02E-12 | 6.36E-11 | 6.48E-11 | -9.45E-11 | 3.73E-11 | 5.70E-08 | 3.02E-07 |
| 127 | -5.26E-10 | 2.95E-11 | 2.54E-11 | 1.37E-11 | 1.18E-11 | -4.93E-11 | -1.63E-10 | 4.47E-08 | 2.89E-07 |
| 128 | -7.23E-10 | -2.21E-11 | -8.19E-12 | 1.48E-11 | 4.42E-11 | 1.14E-11 | -1.23E-11 | 3.41E-08 | 2.73E-07 |
| 129 | 2.38E-10 | 2.55E-11 | 5.07E-11 | -7.99E-12 | -5.21E-11 | 3.50E-11 | -2.73E-11 | 2.05E-08 | 2.55E-07 |
| 130 | 6.91E-10 | -1.02E-11 | -7.80E-12 | -5.71E-12 | -2.63E-12 | 1.30E-11 | 6.55E-11 | 4.65E-09 | 2.38E-07 |
| 131 | -7.15E-10 | 4.97E-10 | 9.42E-11 | -2.68E-10 | -4.57E-10 | -4.06E-11 | 1.09E-09 | 2.81E-09 | 3.73E-09 |
| 132 | -6.72E-10 | -3.72E-11 | -2.78E-12 | 2.97E-11 | 5.67E-11 | 4.45E-11 | -1.83E-10 | -3.61E-11 | 2.08E-07 |
| 133 | -1.97E-09 | 2.31E-12 | 4.77E-12 | 5.06E-12 | 9.98E-14 | -2.65E-11 | -4.36E-11 | 1.84E-11 | 1.96E-07 |
| 134 | 1.36E-10 | -6.78E-12 | -1.31E-11 | -1.37E-11 | -9.67E-12 | 1.34E-11 | -4.02E-12 | 2.58E-13 | 1.84E-07 |
| 135 | -3.04E-11 | 8.21E-10 | 2.17E-10 | -8.52E-11 | -1.65E-10 | -7.96E-11 | -8.62E-11 | 3.15E-10 | 1.72E-07 |
| 136 | 1.72E-09 | -1.37E-10 | -6.11E-11 | -8.35E-13 | 3.79E-11 | 4.34E-11 | -6.11E-11 | 1.46E-10 | 1.60E-07 |
| 137 | -3.97E-10 | 4.74E-11 | 3.18E-11 | 2.01E-11 | 1.19E-11 | 1.44E-12 | -8.59E-12 | 3.39E-10 | 1.48E-07 |
| 138 | 5.68E-10 | -7.66E-13 | -1.96E-12 | -3.42E-12 | -4.91E-12 | -5.72E-12 | -1.35E-11 | -6.21E-12 | 1.36E-07 |
| 139 | -1.20E-09 | 4.69E-12 | -5.78E-12 | -7.11E-12 | -1.43E-12 | 7.61E-12 | -3.71E-12 | 4.23E-11 | 1.23E-07 |
| 140 | -8.03E-10 | 6.99E-12 | 5.37E-12 | 1.56E-12 | -5.01E-12 | -6.32E-12 | -1.27E-11 | 8.14E-12 | 1.11E-07 |
| 141 | -1.80E-09 | 6.23E-13 | 5.26E-13 | 5.13E-13 | 6.03E-13 | 1.27E-12 | -3.92E-12 | 1.64E-13 | 9.95E-08 |
| 142 | -2.69E-10 | -3.77E-14 | -6.74E-13 | -1.73E-12 | -2.99E-12 | -2.22E-12 | 2.17E-11 | 1.50E-10 | 8.92E-08 |
| 143 | 1.79E-09 | 1.59E-12 | 1.64E-12 | 1.10E-12 | -4.47E-13 | -1.35E-11 | -8.05E-11 | 8.39E-11 | 7.81E-08 |
| 144 | -5.45E-11 | 4.87E-12 | 8.74E-12 | 1.13E-11 | 1.19E-11 | 5.47E-12 | -2.90E-11 | 5.57E-12 | 6.93E-08 |
| 145 | 5.41E-10 | 2.55E-12 | 2.15E-12 | 1.51E-12 | 5.36E-13 | -3.62E-12 | -1.38E-11 | 1.48E-11 | 5.98E-08 |
| 146 | 1.68E-11 | -3.83E-12 | -3.81E-12 | -3.73E-12 | -3.57E-12 | -2.91E-12 | -7.98E-12 | 3.39E-11 | 4.98E-08 |
| 147 | 5.41E-10 | -4.02E-13 | -7.89E-14 | -1.21E-12 | -4.09E-12 | -2.01E-11 | -8.88E-11 | 2.07E-11 | 3.93E-08 |
| 148 | 1.64E-09 | -6.31E-13 | -7.09E-13 | 6.86E-13 | 3.67E-12 | 8.20E-12 | 6.36E-14 | -6.01E-14 | 2.81E-08 |
| 149 | -1.13E-10 | 2.31E-12 | -1.52E-12 | -5.05E-12 | -8.28E-12 | -1.38E-11 | -1.81E-11 | -9.12E-13 | 1.60E-08 |

Table.13. Spectra predicted by MLP neural network: Be- 0.4mm

| energy bin (kev)/voltage(kv) | 20kv | 30kv | 40kv | 50kv | 60kv | 80kv | 100kv | 130kv | 150kv |
| --- | --- | --- | --- | --- | --- | --- | --- | --- | --- |
| 1 | 0 | 0 | 0 | 0 | 0 | 0 | 0 | 0 | 0 |
| 2 | 1.08E-09 | -1.51E-09 | 2.15E-14 | 5.72E-15 | 1.64E-14 | -5.38E-14 | -1.25E-13 | 2.55E-14 | 6.81E-14 |
| 3 | 1.50E-09 | -9.00E-10 | 2.87E-11 | 2.48E-10 | 2.83E-10 | -8.17E-11 | -2.46E-10 | 7.24E-10 | 1.36E-09 |
| 4 | 1.88E-09 | 1.03E-09 | -6.93E-09 | -1.58E-08 | -1.71E-08 | -8.53E-09 | 1.11E-08 | 5.77E-08 | 4.55E-08 |
| 5 | -5.79E-10 | -1.25E-09 | 5.92E-08 | 7.52E-08 | 7.01E-08 | 2.08E-08 | 3.56E-08 | 6.89E-08 | 1.16E-08 |
| 6 | 1.65E-09 | -3.35E-10 | -1.91E-08 | -2.26E-08 | -2.21E-08 | -2.04E-08 | -1.19E-08 | 7.04E-08 | 3.12E-07 |
| 7 | 7.88E-09 | 8.70E-09 | 6.88E-08 | 8.58E-08 | 8.07E-08 | -4.19E-09 | 1.38E-07 | 2.04E-08 | 6.38E-08 |
| 8 | 7.08E-08 | 1.10E-07 | 1.29E-07 | 1.36E-07 | 1.45E-07 | 1.63E-07 | 2.04E-07 | 2.28E-07 | 8.01E-08 |
| 9 | 1.56E-07 | 3.24E-07 | 4.44E-07 | 4.97E-07 | 5.08E-07 | 5.22E-07 | 7.79E-07 | 8.78E-07 | 8.54E-07 |
| 10 | 3.43E-07 | 5.80E-07 | 8.71E-07 | 9.90E-07 | 9.66E-07 | 8.94E-07 | 1.11E-06 | 1.51E-06 | 1.31E-06 |
| 11 | 3.93E-07 | 5.93E-07 | 7.37E-07 | 8.52E-07 | 9.31E-07 | 9.86E-07 | 9.81E-07 | 1.11E-06 | 9.50E-07 |
| 12 | 4.18E-07 | 6.92E-07 | 8.57E-07 | 9.32E-07 | 9.80E-07 | 1.05E-06 | 1.11E-06 | 1.16E-06 | 1.03E-06 |
| 13 | 4.49E-07 | 7.77E-07 | 8.72E-07 | 1.02E-06 | 1.14E-06 | 1.19E-06 | 1.30E-06 | 1.10E-06 | 9.59E-07 |
| 14 | 4.97E-07 | 9.36E-07 | 1.29E-06 | 1.46E-06 | 1.56E-06 | 1.65E-06 | 1.70E-06 | 1.72E-06 | 1.75E-06 |
| 15 | 5.01E-07 | 1.06E-06 | 1.43E-06 | 1.67E-06 | 1.87E-06 | 2.09E-06 | 2.23E-06 | 2.09E-06 | 2.05E-06 |
| 16 | 4.39E-07 | 1.07E-06 | 1.57E-06 | 1.92E-06 | 2.13E-06 | 2.38E-06 | 2.53E-06 | 2.63E-06 | 2.47E-06 |
| 17 | 3.79E-07 | 1.08E-06 | 1.61E-06 | 1.94E-06 | 2.20E-06 | 2.54E-06 | 2.82E-06 | 3.26E-06 | 3.50E-06 |
| 18 | 2.88E-07 | 1.05E-06 | 1.59E-06 | 2.02E-06 | 2.35E-06 | 2.80E-06 | 3.10E-06 | 3.52E-06 | 3.28E-06 |
| 19 | 1.94E-07 | 9.99E-07 | 1.57E-06 | 2.02E-06 | 2.38E-06 | 2.92E-06 | 3.32E-06 | 3.81E-06 | 3.54E-06 |
| 20 | 8.09E-08 | 9.37E-07 | 1.57E-06 | 2.05E-06 | 2.42E-06 | 3.02E-06 | 3.49E-06 | 4.04E-06 | 3.87E-06 |
| 21 | -7.37E-10 | 8.68E-07 | 1.50E-06 | 2.00E-06 | 2.43E-06 | 3.11E-06 | 3.60E-06 | 4.15E-06 | 4.05E-06 |
| 22 | 4.00E-11 | 7.87E-07 | 1.43E-06 | 1.95E-06 | 2.39E-06 | 3.10E-06 | 3.66E-06 | 4.40E-06 | 4.25E-06 |
| 23 | -5.45E-10 | 6.99E-07 | 1.33E-06 | 1.86E-06 | 2.31E-06 | 3.04E-06 | 3.67E-06 | 4.49E-06 | 4.37E-06 |
| 24 | -1.09E-09 | 6.08E-07 | 1.20E-06 | 1.77E-06 | 2.26E-06 | 3.04E-06 | 3.68E-06 | 4.50E-06 | 4.49E-06 |
| 25 | 1.00E-09 | 5.23E-07 | 1.13E-06 | 1.70E-06 | 2.19E-06 | 2.97E-06 | 3.68E-06 | 4.52E-06 | 4.58E-06 |
| 26 | -5.23E-10 | 4.36E-07 | 9.87E-07 | 1.56E-06 | 2.08E-06 | 2.93E-06 | 3.65E-06 | 4.48E-06 | 4.58E-06 |
| 27 | 1.58E-09 | 3.47E-07 | 9.45E-07 | 1.53E-06 | 2.06E-06 | 2.86E-06 | 3.59E-06 | 4.54E-06 | 4.70E-06 |
| 28 | 1.40E-10 | 2.55E-07 | 8.94E-07 | 1.51E-06 | 2.00E-06 | 2.80E-06 | 3.56E-06 | 4.54E-06 | 4.74E-06 |
| 29 | -1.90E-09 | 1.61E-07 | 8.25E-07 | 1.42E-06 | 1.89E-06 | 2.77E-06 | 3.51E-06 | 4.49E-06 | 4.76E-06 |
| 30 | 8.33E-10 | 6.08E-08 | 7.00E-07 | 1.23E-06 | 1.77E-06 | 2.74E-06 | 3.50E-06 | 4.39E-06 | 4.77E-06 |
| 31 | -8.31E-11 | -9.89E-10 | 6.61E-07 | 1.13E-06 | 1.64E-06 | 2.64E-06 | 3.45E-06 | 4.38E-06 | 5.04E-06 |
| 32 | 9.64E-10 | -7.14E-10 | 6.42E-07 | 1.20E-06 | 1.67E-06 | 2.51E-06 | 3.25E-06 | 4.22E-06 | 4.60E-06 |
| 33 | -1.34E-09 | -1.00E-09 | 5.33E-07 | 1.09E-06 | 1.55E-06 | 2.41E-06 | 3.20E-06 | 4.15E-06 | 4.64E-06 |
| 34 | -6.89E-10 | -5.04E-10 | 4.73E-07 | 1.02E-06 | 1.47E-06 | 2.30E-06 | 3.11E-06 | 4.03E-06 | 4.57E-06 |
| 35 | 8.24E-10 | 1.18E-09 | 3.85E-07 | 9.59E-07 | 1.40E-06 | 2.18E-06 | 3.02E-06 | 3.94E-06 | 4.48E-06 |
| 36 | -1.77E-09 | 8.11E-10 | 3.05E-07 | 8.65E-07 | 1.31E-06 | 2.10E-06 | 2.87E-06 | 3.80E-06 | 4.37E-06 |
| 37 | 3.32E-10 | 9.85E-11 | 2.26E-07 | 7.35E-07 | 1.24E-06 | 2.02E-06 | 2.79E-06 | 3.76E-06 | 4.27E-06 |
| 38 | -1.91E-09 | 1.13E-09 | 1.95E-07 | 7.34E-07 | 1.19E-06 | 1.94E-06 | 2.72E-06 | 3.66E-06 | 4.21E-06 |
| 39 | -4.60E-10 | -1.69E-09 | 1.15E-07 | 6.65E-07 | 1.11E-06 | 1.85E-06 | 2.64E-06 | 3.58E-06 | 4.03E-06 |
| 40 | 3.74E-10 | 3.73E-10 | 7.01E-08 | 6.02E-07 | 1.07E-06 | 1.78E-06 | 2.52E-06 | 3.50E-06 | 4.01E-06 |
| 41 | -1.64E-09 | -1.38E-09 | 1.37E-08 | 5.31E-07 | 9.76E-07 | 1.73E-06 | 2.43E-06 | 3.39E-06 | 3.92E-06 |
| 42 | 8.37E-10 | 3.43E-10 | 1.60E-08 | 4.78E-07 | 9.15E-07 | 1.65E-06 | 2.35E-06 | 3.30E-06 | 3.81E-06 |
| 43 | 1.04E-09 | 1.82E-09 | 1.33E-08 | 4.15E-07 | 8.61E-07 | 1.59E-06 | 2.27E-06 | 3.20E-06 | 3.73E-06 |
| 44 | 1.11E-09 | 5.83E-10 | -2.16E-09 | 3.62E-07 | 7.77E-07 | 1.52E-06 | 2.19E-06 | 3.10E-06 | 3.63E-06 |
| 45 | 7.34E-10 | 1.39E-09 | 4.94E-09 | 3.10E-07 | 7.34E-07 | 1.45E-06 | 2.12E-06 | 3.00E-06 | 3.53E-06 |
| 46 | -1.11E-09 | 1.44E-09 | -8.10E-10 | 2.52E-07 | 6.81E-07 | 1.39E-06 | 2.05E-06 | 2.93E-06 | 3.45E-06 |
| 47 | -1.12E-10 | 2.75E-10 | -3.58E-09 | 1.97E-07 | 6.30E-07 | 1.33E-06 | 1.98E-06 | 2.83E-06 | 3.37E-06 |
| 48 | -4.21E-10 | 4.28E-10 | 9.10E-10 | 1.48E-07 | 5.78E-07 | 1.27E-06 | 1.91E-06 | 2.75E-06 | 3.28E-06 |
| 49 | -9.25E-11 | -1.61E-10 | -1.99E-08 | 1.64E-07 | 5.11E-07 | 1.24E-06 | 1.79E-06 | 2.65E-06 | 3.20E-06 |
| 50 | 7.64E-10 | 5.13E-10 | -1.04E-09 | 3.98E-08 | 4.81E-07 | 1.15E-06 | 1.76E-06 | 2.59E-06 | 3.12E-06 |
| 51 | -1.41E-10 | 8.60E-10 | -3.83E-08 | 8.08E-09 | 4.07E-07 | 1.12E-06 | 1.68E-06 | 2.52E-06 | 3.02E-06 |
| 52 | 8.30E-10 | -1.00E-09 | 2.39E-08 | 1.51E-07 | 4.02E-07 | 1.05E-06 | 1.65E-06 | 2.37E-06 | 2.95E-06 |
| 53 | -1.14E-09 | 6.99E-10 | -2.27E-08 | 3.89E-08 | 3.18E-07 | 9.65E-07 | 1.62E-06 | 2.38E-06 | 2.89E-06 |
| 54 | -9.81E-10 | -2.11E-10 | -2.77E-08 | 2.54E-08 | 2.47E-07 | 9.26E-07 | 1.53E-06 | 2.31E-06 | 2.78E-06 |
| 55 | -1.87E-09 | -1.71E-09 | -4.62E-09 | 1.70E-08 | 2.49E-07 | 8.85E-07 | 1.49E-06 | 2.27E-06 | 2.76E-06 |
| 56 | 1.16E-09 | 8.43E-10 | -3.25E-08 | 2.34E-08 | 2.24E-07 | 8.10E-07 | 1.42E-06 | 2.23E-06 | 2.68E-06 |
| 57 | -1.18E-09 | -1.59E-09 | -5.89E-09 | 6.97E-09 | 1.54E-07 | 7.91E-07 | 1.38E-06 | 2.12E-06 | 2.64E-06 |
| 58 | -7.30E-10 | -1.02E-09 | -1.31E-08 | -7.21E-09 | 1.29E-07 | 7.43E-07 | 1.29E-06 | 2.04E-06 | 2.56E-06 |
| 59 | -4.22E-10 | -1.83E-10 | 1.96E-09 | -9.79E-10 | 7.26E-08 | 8.65E-07 | 2.28E-06 | 4.94E-06 | 6.69E-06 |
| 60 | -7.40E-10 | -1.04E-09 | 1.35E-09 | -2.16E-08 | 4.23E-08 | 9.57E-07 | 3.03E-06 | 7.28E-06 | 1.01E-05 |
| 61 | 1.31E-09 | -1.35E-09 | 1.06E-08 | -2.39E-08 | 2.04E-08 | 6.16E-07 | 1.12E-06 | 1.91E-06 | 2.60E-06 |
| 62 | -1.73E-10 | 1.01E-10 | -1.38E-09 | -1.41E-10 | 2.74E-09 | 6.05E-07 | 1.22E-06 | 1.96E-06 | 2.33E-06 |
| 63 | -1.55E-09 | -1.97E-09 | 2.30E-09 | -2.27E-09 | 5.05E-10 | 6.02E-07 | 1.07E-06 | 1.66E-06 | 2.13E-06 |
| 64 | -1.62E-09 | 9.10E-10 | 1.65E-09 | -2.06E-11 | 4.15E-10 | 5.26E-07 | 1.04E-06 | 1.58E-06 | 2.06E-06 |
| 65 | -5.18E-12 | 1.25E-09 | -1.00E-09 | 7.46E-10 | 9.36E-10 | 4.89E-07 | 9.93E-07 | 1.58E-06 | 2.06E-06 |
| 66 | -8.30E-10 | -8.09E-10 | -5.17E-09 | -7.02E-09 | 6.36E-08 | 4.48E-07 | 9.45E-07 | 1.56E-06 | 2.03E-06 |
| 67 | 3.36E-10 | -2.99E-10 | 1.97E-10 | 4.26E-10 | -1.43E-11 | 4.37E-07 | 8.64E-07 | 1.50E-06 | 1.98E-06 |
| 68 | -1.85E-09 | 4.51E-10 | -1.84E-09 | -2.58E-09 | 2.03E-09 | 4.89E-07 | 1.54E-06 | 3.31E-06 | 4.69E-06 |
| 69 | 1.16E-09 | 3.55E-10 | 3.74E-09 | -3.56E-10 | 4.39E-09 | 3.48E-07 | 8.18E-07 | 1.46E-06 | 1.80E-06 |
| 70 | 9.26E-10 | -6.88E-10 | 4.23E-11 | -7.99E-10 | 1.69E-08 | 3.34E-07 | 9.39E-07 | 1.86E-06 | 2.50E-06 |
| 71 | 6.99E-10 | 1.43E-09 | -2.37E-09 | -2.33E-09 | 1.57E-09 | 2.85E-07 | 7.61E-07 | 1.27E-06 | 1.49E-06 |
| 72 | -1.04E-09 | 1.11E-09 | 1.64E-10 | -6.57E-10 | 1.22E-10 | 2.54E-07 | 6.75E-07 | 1.24E-06 | 1.51E-06 |
| 73 | 6.78E-10 | 1.67E-09 | 3.49E-10 | -4.04E-09 | 7.44E-09 | 2.22E-07 | 6.43E-07 | 1.19E-06 | 1.47E-06 |
| 74 | 1.48E-09 | -1.82E-10 | 2.47E-10 | -4.34E-09 | -3.51E-10 | 1.90E-07 | 6.12E-07 | 1.15E-06 | 1.43E-06 |
| 75 | 8.27E-10 | 1.74E-09 | 2.10E-09 | 3.18E-10 | 2.69E-09 | 1.59E-07 | 5.84E-07 | 1.11E-06 | 1.39E-06 |
| 76 | 8.73E-10 | 4.19E-10 | 5.13E-10 | 3.62E-10 | 1.66E-09 | 1.33E-07 | 5.60E-07 | 1.10E-06 | 1.36E-06 |
| 77 | 1.94E-09 | -3.22E-10 | -5.61E-10 | -1.21E-09 | -4.53E-10 | 1.02E-07 | 5.37E-07 | 1.06E-06 | 1.32E-06 |
| 78 | 1.91E-09 | 1.84E-09 | 9.03E-10 | -4.30E-10 | -9.95E-10 | 7.31E-08 | 5.17E-07 | 1.03E-06 | 1.30E-06 |
| 79 | 1.54E-09 | -6.31E-10 | 2.71E-10 | 4.59E-10 | 5.77E-10 | 4.49E-08 | 4.95E-07 | 9.93E-07 | 1.27E-06 |
| 80 | -5.90E-10 | 1.47E-09 | 1.51E-09 | 3.91E-09 | -1.37E-09 | 1.54E-08 | 4.71E-07 | 9.53E-07 | 1.24E-06 |
| 81 | -1.48E-10 | -1.56E-09 | -9.86E-11 | -6.36E-11 | -1.16E-10 | 4.74E-11 | 4.45E-07 | 9.23E-07 | 1.22E-06 |
| 82 | -1.16E-09 | -1.98E-09 | -7.42E-10 | -1.19E-09 | -1.87E-09 | -8.86E-11 | 4.20E-07 | 8.89E-07 | 1.19E-06 |
| 83 | 7.18E-11 | 3.50E-10 | 4.46E-11 | 2.62E-11 | -4.19E-10 | -4.83E-10 | 3.93E-07 | 8.58E-07 | 1.16E-06 |
| 84 | 3.52E-10 | -5.23E-10 | -2.45E-08 | -1.77E-08 | -1.72E-09 | 8.29E-08 | 2.97E-07 | 8.36E-07 | 1.13E-06 |
| 85 | -2.41E-11 | 6.02E-11 | -2.96E-10 | -3.56E-10 | -5.17E-10 | -5.28E-11 | 3.44E-07 | 7.94E-07 | 1.10E-06 |
| 86 | 1.36E-09 | 1.07E-09 | -1.96E-08 | -1.77E-08 | -2.86E-09 | 8.64E-08 | 3.00E-07 | 7.58E-07 | 1.05E-06 |
| 87 | -1.75E-09 | -8.39E-10 | -4.57E-11 | 5.97E-11 | -2.06E-10 | -7.07E-10 | 2.96E-07 | 7.59E-07 | 1.06E-06 |
| 88 | -9.22E-10 | 1.89E-09 | -1.58E-09 | 2.08E-09 | -8.84E-10 | -1.73E-10 | 2.69E-07 | 7.39E-07 | 1.03E-06 |
| 89 | 8.24E-10 | -2.49E-10 | 7.69E-12 | 2.45E-11 | -2.25E-11 | -8.01E-11 | 2.46E-07 | 7.16E-07 | 1.00E-06 |
| 90 | 1.09E-09 | 6.12E-10 | 8.58E-11 | 6.24E-11 | -7.39E-11 | 1.20E-10 | 2.21E-07 | 6.97E-07 | 9.82E-07 |
| 91 | -1.52E-09 | 1.54E-09 | -8.24E-10 | -1.55E-09 | -1.94E-09 | 4.88E-09 | 2.00E-07 | 6.75E-07 | 9.59E-07 |
| 92 | -7.49E-11 | -9.71E-10 | 1.30E-11 | 5.17E-12 | -1.35E-11 | 1.23E-10 | 1.77E-07 | 6.51E-07 | 9.32E-07 |
| 93 | -3.64E-10 | 1.34E-09 | 1.62E-11 | 5.96E-11 | -6.14E-11 | -1.97E-11 | 1.58E-07 | 6.33E-07 | 9.08E-07 |
| 94 | 6.46E-12 | 1.75E-09 | 3.21E-10 | 5.10E-10 | -1.13E-10 | 5.78E-10 | 1.39E-07 | 6.11E-07 | 8.80E-07 |
| 95 | -7.16E-10 | 1.43E-09 | -2.03E-10 | 7.06E-11 | -5.63E-11 | -2.06E-10 | 1.18E-07 | 5.88E-07 | 8.51E-07 |
| 96 | 4.39E-10 | -1.03E-09 | 6.54E-10 | -3.96E-09 | -1.02E-08 | -5.43E-09 | 1.05E-07 | 5.68E-07 | 8.17E-07 |
| 97 | -3.67E-10 | -1.51E-09 | -6.09E-10 | 3.60E-11 | -5.24E-10 | -1.65E-10 | 7.57E-08 | 5.38E-07 | 7.97E-07 |
| 98 | 1.57E-09 | -1.49E-09 | 1.10E-10 | 1.33E-10 | -1.11E-10 | -5.31E-12 | 5.34E-08 | 5.15E-07 | 7.72E-07 |
| 99 | 1.10E-09 | -2.85E-10 | 3.64E-11 | -4.01E-11 | -8.13E-11 | -7.95E-11 | 3.16E-08 | 4.93E-07 | 7.51E-07 |
| 100 | -5.84E-10 | -4.92E-10 | -3.41E-11 | -3.18E-13 | -2.15E-11 | 7.52E-11 | 1.23E-08 | 4.76E-07 | 7.33E-07 |
| 101 | 1.98E-09 | -1.73E-09 | 9.00E-11 | -5.24E-11 | -2.44E-10 | -6.21E-10 | 9.34E-10 | 4.61E-07 | 7.16E-07 |
| 102 | -5.39E-10 | -1.27E-09 | -1.33E-12 | 7.74E-12 | 2.98E-11 | 5.20E-12 | -3.68E-11 | 4.47E-07 | 7.01E-07 |
| 103 | 2.89E-10 | 1.22E-09 | 3.70E-11 | 9.88E-11 | 1.67E-10 | 1.74E-10 | -1.17E-10 | 4.28E-07 | 6.81E-07 |
| 104 | 1.93E-09 | -1.67E-09 | -3.83E-12 | 2.25E-11 | 3.87E-13 | -9.51E-11 | 4.31E-11 | 4.11E-07 | 6.62E-07 |
| 105 | -1.76E-09 | 1.67E-09 | -2.18E-12 | 5.51E-11 | 1.04E-10 | -6.68E-11 | 1.87E-11 | 3.91E-07 | 6.41E-07 |
| 106 | -1.07E-09 | -1.41E-10 | -2.67E-11 | -6.91E-11 | -8.52E-12 | 6.93E-11 | 8.71E-10 | 3.72E-07 | 6.22E-07 |
| 107 | 4.57E-11 | -1.61E-09 | 4.46E-11 | -6.63E-11 | -2.23E-10 | -4.56E-10 | -2.66E-11 | 3.50E-07 | 6.04E-07 |
| 108 | -1.16E-09 | 8.49E-10 | 8.56E-12 | 1.05E-11 | 2.96E-12 | -5.38E-11 | 2.80E-11 | 3.35E-07 | 5.87E-07 |
| 109 | 7.71E-10 | 1.56E-09 | -9.66E-11 | -2.83E-10 | -4.73E-10 | -8.22E-10 | -8.83E-11 | 3.19E-07 | 5.72E-07 |
| 110 | -1.56E-09 | 1.57E-11 | 2.92E-11 | 6.73E-11 | 1.39E-10 | 3.37E-10 | 5.08E-10 | 3.07E-07 | 5.58E-07 |
| 111 | -2.28E-10 | 1.21E-09 | -2.93E-11 | -2.97E-11 | -3.69E-11 | -4.62E-11 | 5.67E-10 | 2.94E-07 | 5.44E-07 |
| 112 | 1.55E-09 | -9.63E-10 | 1.32E-11 | 1.84E-11 | 8.18E-12 | -2.67E-11 | 2.13E-10 | 2.78E-07 | 5.28E-07 |
| 113 | -7.51E-10 | -6.01E-10 | -4.05E-10 | -1.57E-10 | 2.22E-11 | 2.46E-10 | 2.48E-09 | 2.57E-07 | 5.05E-07 |
| 114 | 8.05E-10 | -8.86E-10 | -1.19E-10 | -1.22E-10 | -1.03E-10 | -1.42E-11 | 3.67E-10 | 2.34E-07 | 4.92E-07 |
| 115 | -1.93E-09 | 9.28E-10 | 2.00E-11 | -8.92E-12 | -5.17E-11 | -1.04E-10 | 1.81E-10 | 2.14E-07 | 4.71E-07 |
| 116 | -4.88E-10 | 4.77E-10 | -3.07E-11 | -1.27E-11 | 2.59E-11 | -4.58E-11 | 1.08E-11 | 1.95E-07 | 4.49E-07 |
| 117 | 7.44E-10 | 1.44E-09 | -5.47E-10 | -4.43E-10 | 1.26E-10 | 3.69E-10 | -7.16E-10 | 1.77E-07 | 4.29E-07 |
| 118 | 6.53E-10 | 1.99E-09 | 1.27E-11 | -8.54E-12 | -3.96E-11 | -5.88E-11 | 5.56E-10 | 1.62E-07 | 4.09E-07 |
| 119 | 1.11E-09 | -4.40E-10 | 5.60E-11 | 4.19E-11 | -6.31E-11 | -4.44E-10 | 5.41E-11 | 1.50E-07 | 3.93E-07 |
| 120 | -1.74E-09 | -9.26E-10 | 1.94E-11 | -4.05E-12 | -2.77E-11 | -8.60E-11 | -1.88E-12 | 1.38E-07 | 3.78E-07 |
| 121 | 8.96E-10 | -6.78E-10 | -8.34E-11 | 1.27E-11 | 2.03E-10 | -1.74E-10 | -7.04E-10 | 1.26E-07 | 3.64E-07 |
| 122 | -7.49E-10 | -6.77E-10 | 3.95E-11 | 5.94E-11 | 1.94E-10 | 1.28E-11 | 1.29E-10 | 1.14E-07 | 3.49E-07 |
| 123 | 1.82E-09 | 4.19E-10 | 5.06E-11 | -5.13E-11 | 2.72E-12 | -5.59E-11 | 1.02E-10 | 9.94E-08 | 3.38E-07 |
| 124 | 1.47E-09 | -1.26E-09 | 4.81E-11 | 4.04E-11 | -1.37E-11 | -2.74E-10 | -2.23E-10 | 8.49E-08 | 3.24E-07 |
| 125 | 1.53E-09 | -1.28E-09 | 6.71E-11 | 1.23E-10 | 1.44E-10 | 3.84E-11 | -2.22E-10 | 6.96E-08 | 3.12E-07 |
| 126 | 1.59E-09 | 1.95E-10 | 8.57E-12 | 3.89E-11 | 6.09E-11 | -7.77E-11 | 1.20E-10 | 5.69E-08 | 2.99E-07 |
| 127 | -1.20E-09 | -1.82E-12 | -6.66E-12 | -1.98E-11 | -7.20E-12 | -2.90E-11 | -6.41E-11 | 4.39E-08 | 2.84E-07 |
| 128 | 1.33E-11 | -1.29E-09 | -1.44E-11 | 8.03E-12 | 4.31E-11 | 6.88E-11 | 9.08E-11 | 3.11E-08 | 2.68E-07 |
| 129 | 1.07E-10 | 7.53E-10 | -1.67E-12 | 8.80E-12 | 1.73E-11 | -7.97E-11 | -6.71E-10 | 1.85E-08 | 2.53E-07 |
| 130 | 3.47E-10 | 4.06E-10 | 7.98E-12 | 1.23E-11 | 1.35E-11 | 8.56E-12 | -5.18E-12 | 4.60E-09 | 2.37E-07 |
| 131 | 1.63E-09 | 7.98E-10 | -5.98E-10 | 5.33E-10 | 9.97E-10 | 2.44E-10 | -6.77E-10 | 4.88E-09 | 2.23E-07 |
| 132 | 1.11E-09 | -3.61E-10 | 4.70E-12 | 6.14E-12 | 7.00E-12 | 2.73E-12 | -5.01E-11 | -1.18E-10 | 2.07E-07 |
| 133 | 6.86E-10 | 1.18E-09 | 1.12E-11 | 2.71E-15 | -5.16E-12 | 7.19E-13 | 2.06E-11 | 2.29E-11 | 1.94E-07 |
| 134 | -1.74E-09 | 9.61E-10 | -2.15E-13 | -2.32E-12 | -4.83E-12 | -3.37E-12 | 6.47E-12 | 1.46E-11 | 1.82E-07 |
| 135 | 1.10E-11 | 8.09E-10 | 2.93E-10 | 3.35E-10 | 3.75E-10 | 3.60E-10 | 8.00E-11 | -4.35E-11 | 1.70E-07 |
| 136 | -1.70E-09 | -1.70E-09 | 9.46E-11 | 1.45E-10 | 1.65E-10 | 5.82E-11 | -2.01E-10 | -1.59E-10 | 1.58E-07 |
| 137 | 2.01E-10 | -5.27E-10 | 3.17E-11 | 3.37E-11 | 3.09E-11 | 1.37E-11 | -2.12E-11 | 1.56E-10 | 1.46E-07 |
| 138 | 1.30E-10 | -2.19E-10 | -2.85E-12 | -4.28E-12 | -6.47E-12 | -1.28E-11 | -3.08E-11 | -7.13E-11 | 1.34E-07 |
| 139 | -9.69E-10 | 7.57E-10 | -2.41E-12 | 7.42E-12 | 1.59E-11 | 1.65E-11 | -5.75E-12 | 1.71E-11 | 1.22E-07 |
| 140 | -1.58E-09 | -1.95E-09 | 2.32E-13 | -2.54E-12 | -5.26E-12 | -5.92E-12 | 3.03E-11 | 1.79E-11 | 1.11E-07 |
| 141 | 1.09E-09 | 5.32E-10 | 2.35E-12 | 2.49E-12 | 1.92E-12 | 3.79E-13 | -4.95E-12 | 2.91E-11 | 9.92E-08 |
| 142 | -3.04E-10 | -6.35E-10 | 3.08E-13 | -5.14E-13 | -1.20E-12 | 2.10E-12 | 2.77E-12 | 2.60E-11 | 8.86E-08 |
| 143 | 1.25E-09 | -5.07E-10 | -1.20E-12 | -2.19E-12 | -3.04E-12 | -4.95E-12 | -1.43E-11 | 9.93E-11 | 7.83E-08 |
| 144 | 1.79E-09 | 1.17E-09 | -8.69E-12 | -9.75E-12 | -9.44E-12 | -3.77E-12 | -2.23E-11 | 5.67E-12 | 6.86E-08 |
| 145 | -1.48E-09 | -9.05E-10 | -1.12E-13 | -6.40E-13 | -1.20E-12 | -2.79E-12 | -7.38E-12 | 1.42E-11 | 5.90E-08 |
| 146 | -1.76E-09 | 1.93E-09 | 5.23E-12 | 6.13E-12 | 7.14E-12 | 9.37E-12 | 4.20E-12 | -1.12E-11 | 4.87E-08 |
| 147 | -4.88E-10 | -1.41E-09 | 7.49E-12 | 1.20E-11 | 1.36E-11 | 6.08E-12 | -5.89E-11 | -6.60E-12 | 3.83E-08 |
| 148 | -1.73E-10 | 9.83E-10 | 5.62E-13 | 4.61E-13 | 4.20E-13 | 5.97E-13 | -1.48E-13 | 8.17E-13 | 2.72E-08 |
| 149 | 1.46E-09 | 6.65E-10 | -3.21E-12 | -2.75E-12 | -2.63E-12 | -3.46E-12 | -5.08E-12 | 1.32E-11 | 1.60E-08 |
| 150 | 8.08E-10 | 6.12E-10 | 6.57E-13 | 2.87E-13 | -1.39E-14 | -4.30E-13 | -3.33E-13 | 7.06E-13 | 3.79E-09 |

Table.14. Spectra predicted by MLP neural network: Be- 0.8mm

| energy bin (kev)/voltage(kv) | 20kv | 30kv | 40kv | 50kv | 60kv | 80kv | 100kv | 130kv | 150kv |
| --- | --- | --- | --- | --- | --- | --- | --- | --- | --- |
| 1 | 0 | 0 | 0 | 0 | 0 | 0 | 0 | 0 | 0 |
| 2 | -8.91E-10 | -1.86E-09 | -1.19E-14 | -2.50E-14 | -3.28E-14 | -9.12E-14 | -1.12E-13 | 5.43E-14 | 5.99E-14 |
| 3 | 1.55E-09 | -1.17E-10 | -5.40E-11 | -2.77E-11 | -3.01E-11 | -9.92E-11 | -1.10E-10 | -2.92E-10 | 1.80E-11 |
| 4 | -1.59E-09 | 5.38E-10 | -1.20E-09 | -3.76E-09 | -5.76E-09 | -9.41E-09 | -1.03E-08 | 4.30E-09 | 3.02E-09 |
| 5 | 7.29E-10 | -1.61E-09 | 2.96E-08 | 4.86E-08 | 5.14E-08 | 3.61E-09 | -6.38E-09 | 4.61E-08 | 5.28E-09 |
| 6 | -1.66E-09 | 9.81E-10 | 8.93E-10 | -1.71E-10 | -7.11E-10 | -6.17E-09 | -1.26E-08 | -6.07E-09 | 3.56E-08 |
| 7 | 1.20E-09 | 1.15E-09 | 1.26E-08 | 2.77E-08 | 3.18E-08 | -1.63E-08 | 1.11E-07 | -4.22E-08 | -1.49E-08 |
| 8 | 2.67E-09 | 3.15E-09 | 2.53E-08 | 6.97E-09 | -1.07E-08 | -3.34E-08 | 1.09E-09 | 4.46E-08 | -5.33E-08 |
| 9 | 8.03E-09 | 1.87E-08 | 4.66E-08 | 5.56E-08 | 3.42E-08 | -2.99E-08 | 7.90E-08 | 5.32E-08 | -1.55E-08 |
| 10 | 4.78E-08 | 8.45E-08 | 1.94E-07 | 1.55E-07 | 1.08E-07 | 1.69E-07 | 3.19E-07 | 4.91E-07 | 2.27E-07 |
| 11 | 7.14E-08 | 1.10E-07 | 1.35E-07 | 1.88E-07 | 2.25E-07 | 2.53E-07 | 2.06E-07 | 2.28E-07 | 1.92E-07 |
| 12 | 1.02E-07 | 1.73E-07 | 2.26E-07 | 2.59E-07 | 2.76E-07 | 2.94E-07 | 3.17E-07 | 3.34E-07 | 2.26E-07 |
| 13 | 1.47E-07 | 2.49E-07 | 2.81E-07 | 3.42E-07 | 3.92E-07 | 3.33E-07 | 3.95E-07 | 4.04E-07 | 2.08E-07 |
| 14 | 2.11E-07 | 3.99E-07 | 5.39E-07 | 6.05E-07 | 6.56E-07 | 7.57E-07 | 8.23E-07 | 7.13E-07 | 7.21E-07 |
| 15 | 2.63E-07 | 5.64E-07 | 8.06E-07 | 9.29E-07 | 1.00E-06 | 1.16E-06 | 1.21E-06 | 1.13E-06 | 1.09E-06 |
| 16 | 2.49E-07 | 6.18E-07 | 9.84E-07 | 1.18E-06 | 1.27E-06 | 1.38E-06 | 1.59E-06 | 1.68E-06 | 1.48E-06 |
| 17 | 2.34E-07 | 6.87E-07 | 1.02E-06 | 1.26E-06 | 1.45E-06 | 1.68E-06 | 1.85E-06 | 2.07E-06 | 1.85E-06 |
| 18 | 1.87E-07 | 7.12E-07 | 1.10E-06 | 1.39E-06 | 1.61E-06 | 1.93E-06 | 2.13E-06 | 2.43E-06 | 2.22E-06 |
| 19 | 1.36E-07 | 7.18E-07 | 1.15E-06 | 1.48E-06 | 1.75E-06 | 2.14E-06 | 2.42E-06 | 2.78E-06 | 2.54E-06 |
| 20 | 6.20E-08 | 7.05E-07 | 1.17E-06 | 1.53E-06 | 1.82E-06 | 2.30E-06 | 2.66E-06 | 3.07E-06 | 2.89E-06 |
| 21 | -1.65E-09 | 6.75E-07 | 1.17E-06 | 1.56E-06 | 1.89E-06 | 2.42E-06 | 2.81E-06 | 3.31E-06 | 3.22E-06 |
| 22 | -1.95E-09 | 6.28E-07 | 1.13E-06 | 1.55E-06 | 1.92E-06 | 2.49E-06 | 2.94E-06 | 3.54E-06 | 3.41E-06 |
| 23 | 8.33E-11 | 5.66E-07 | 1.08E-06 | 1.52E-06 | 1.89E-06 | 2.50E-06 | 3.01E-06 | 3.66E-06 | 3.55E-06 |
| 24 | 1.22E-09 | 5.02E-07 | 1.01E-06 | 1.48E-06 | 1.89E-06 | 2.52E-06 | 3.05E-06 | 3.73E-06 | 3.73E-06 |
| 25 | 5.23E-10 | 4.39E-07 | 9.50E-07 | 1.44E-06 | 1.86E-06 | 2.52E-06 | 3.10E-06 | 3.83E-06 | 3.90E-06 |
| 26 | -1.54E-09 | 3.74E-07 | 8.77E-07 | 1.38E-06 | 1.83E-06 | 2.53E-06 | 3.14E-06 | 3.90E-06 | 3.99E-06 |
| 27 | -4.25E-10 | 3.00E-07 | 8.24E-07 | 1.34E-06 | 1.80E-06 | 2.50E-06 | 3.16E-06 | 3.98E-06 | 4.10E-06 |
| 28 | -7.92E-10 | 2.24E-07 | 7.92E-07 | 1.39E-06 | 1.83E-06 | 2.49E-06 | 3.20E-06 | 4.03E-06 | 4.23E-06 |
| 29 | 1.10E-09 | 1.44E-07 | 7.46E-07 | 1.27E-06 | 1.69E-06 | 2.49E-06 | 3.16E-06 | 4.07E-06 | 4.32E-06 |
| 30 | 1.87E-09 | 5.45E-08 | 5.89E-07 | 1.11E-06 | 1.61E-06 | 2.46E-06 | 3.14E-06 | 4.00E-06 | 4.28E-06 |
| 31 | -1.45E-09 | -7.72E-10 | 5.59E-07 | 1.02E-06 | 1.49E-06 | 2.42E-06 | 3.19E-06 | 4.00E-06 | 4.50E-06 |
| 32 | -9.17E-10 | -8.94E-10 | 5.76E-07 | 1.09E-06 | 1.52E-06 | 2.32E-06 | 3.06E-06 | 3.98E-06 | 4.34E-06 |
| 33 | 1.69E-09 | 2.07E-11 | 5.08E-07 | 1.02E-06 | 1.44E-06 | 2.25E-06 | 2.98E-06 | 3.86E-06 | 4.32E-06 |
| 34 | 1.12E-09 | 1.84E-09 | 4.37E-07 | 9.53E-07 | 1.37E-06 | 2.16E-06 | 2.92E-06 | 3.78E-06 | 4.28E-06 |
| 35 | 7.22E-10 | -1.04E-09 | 3.59E-07 | 8.95E-07 | 1.31E-06 | 2.05E-06 | 2.83E-06 | 3.69E-06 | 4.22E-06 |
| 36 | 1.73E-09 | 8.63E-10 | 2.90E-07 | 8.28E-07 | 1.25E-06 | 1.98E-06 | 2.73E-06 | 3.62E-06 | 4.15E-06 |
| 37 | -1.04E-09 | -3.22E-11 | 3.02E-07 | 7.29E-07 | 1.18E-06 | 1.90E-06 | 2.65E-06 | 3.60E-06 | 4.07E-06 |
| 38 | 1.08E-09 | -1.95E-09 | 1.81E-07 | 6.94E-07 | 1.12E-06 | 1.83E-06 | 2.57E-06 | 3.48E-06 | 3.99E-06 |
| 39 | -1.20E-09 | 9.07E-10 | 1.07E-07 | 6.34E-07 | 1.06E-06 | 1.77E-06 | 2.50E-06 | 3.42E-06 | 3.88E-06 |
| 40 | -1.82E-09 | 1.13E-09 | 5.55E-08 | 5.69E-07 | 1.00E-06 | 1.70E-06 | 2.41E-06 | 3.34E-06 | 3.84E-06 |
| 41 | -4.95E-11 | 1.67E-09 | 9.55E-09 | 5.10E-07 | 9.32E-07 | 1.65E-06 | 2.35E-06 | 3.25E-06 | 3.77E-06 |
| 42 | -6.37E-10 | 1.78E-09 | -1.59E-09 | 4.50E-07 | 8.76E-07 | 1.59E-06 | 2.25E-06 | 3.16E-06 | 3.67E-06 |
| 43 | -3.59E-10 | -1.82E-09 | 1.89E-09 | 3.92E-07 | 8.24E-07 | 1.52E-06 | 2.18E-06 | 3.07E-06 | 3.60E-06 |
| 44 | -7.32E-10 | 2.00E-10 | 2.16E-09 | 3.54E-07 | 7.53E-07 | 1.47E-06 | 2.11E-06 | 2.99E-06 | 3.51E-06 |
| 45 | 1.06E-09 | -9.13E-10 | 1.04E-09 | 2.97E-07 | 7.08E-07 | 1.41E-06 | 2.05E-06 | 2.91E-06 | 3.42E-06 |
| 46 | -5.41E-10 | -8.50E-10 | 1.33E-09 | 2.46E-07 | 6.62E-07 | 1.35E-06 | 1.98E-06 | 2.83E-06 | 3.34E-06 |
| 47 | -1.89E-10 | -1.68E-09 | -3.15E-10 | 1.91E-07 | 6.10E-07 | 1.29E-06 | 1.92E-06 | 2.74E-06 | 3.26E-06 |
| 48 | 1.78E-09 | -5.64E-10 | -2.89E-10 | 1.41E-07 | 5.60E-07 | 1.23E-06 | 1.85E-06 | 2.67E-06 | 3.19E-06 |
| 49 | -1.52E-09 | 7.22E-10 | -2.98E-08 | 1.47E-07 | 4.86E-07 | 1.20E-06 | 1.76E-06 | 2.59E-06 | 3.11E-06 |
| 50 | -5.69E-10 | 1.44E-09 | -2.51E-10 | 3.74E-08 | 4.67E-07 | 1.12E-06 | 1.72E-06 | 2.52E-06 | 3.03E-06 |
| 51 | -4.88E-10 | 1.34E-09 | -1.99E-08 | 2.30E-08 | 4.08E-07 | 1.10E-06 | 1.63E-06 | 2.46E-06 | 2.95E-06 |
| 52 | -1.27E-09 | -8.18E-10 | 2.52E-08 | 1.22E-07 | 3.57E-07 | 1.03E-06 | 1.61E-06 | 2.36E-06 | 2.89E-06 |
| 53 | 8.90E-10 | 1.26E-09 | -2.77E-08 | 3.40E-08 | 3.15E-07 | 9.53E-07 | 1.58E-06 | 2.34E-06 | 2.82E-06 |
| 54 | 4.30E-10 | 6.93E-10 | -1.86E-08 | 3.60E-08 | 2.58E-07 | 9.23E-07 | 1.51E-06 | 2.28E-06 | 2.75E-06 |
| 55 | 1.99E-09 | -1.78E-09 | -7.39E-09 | 9.58E-09 | 2.37E-07 | 8.63E-07 | 1.46E-06 | 2.21E-06 | 2.69E-06 |
| 56 | 1.55E-10 | 1.02E-09 | -1.55E-08 | 4.52E-08 | 2.50E-07 | 8.19E-07 | 1.40E-06 | 2.18E-06 | 2.61E-06 |
| 57 | 5.89E-10 | -1.92E-09 | -1.82E-09 | 9.83E-09 | 1.54E-07 | 7.78E-07 | 1.35E-06 | 2.08E-06 | 2.58E-06 |
| 58 | 1.68E-09 | 4.63E-10 | -1.57E-08 | -1.50E-08 | 1.13E-07 | 7.14E-07 | 1.26E-06 | 2.00E-06 | 2.53E-06 |
| 59 | 9.24E-10 | 3.15E-10 | 1.78E-09 | -1.44E-09 | 7.02E-08 | 8.49E-07 | 2.24E-06 | 4.84E-06 | 6.55E-06 |
| 60 | 3.62E-10 | -8.26E-10 | 1.00E-08 | -1.56E-08 | 4.24E-08 | 9.34E-07 | 2.97E-06 | 7.12E-06 | 9.89E-06 |
| 61 | -1.46E-10 | -1.72E-09 | 9.04E-09 | -2.30E-08 | 2.05E-08 | 6.00E-07 | 1.09E-06 | 1.85E-06 | 2.43E-06 |
| 62 | -7.18E-10 | -5.32E-10 | -1.68E-09 | -5.59E-10 | 1.16E-09 | 5.94E-07 | 1.19E-06 | 1.93E-06 | 2.35E-06 |
| 63 | 1.35E-10 | -6.45E-10 | 2.20E-09 | -1.26E-09 | 2.74E-09 | 5.96E-07 | 1.05E-06 | 1.63E-06 | 2.09E-06 |
| 64 | -1.48E-09 | -8.42E-10 | 1.36E-10 | -7.88E-10 | -6.27E-10 | 5.14E-07 | 1.02E-06 | 1.55E-06 | 2.02E-06 |
| 65 | -7.67E-10 | -6.46E-11 | 3.82E-10 | 2.39E-10 | -2.68E-10 | 4.79E-07 | 9.76E-07 | 1.55E-06 | 2.02E-06 |
| 66 | 4.89E-10 | -1.61E-09 | 2.23E-10 | -9.63E-09 | 5.51E-08 | 4.31E-07 | 9.20E-07 | 1.53E-06 | 1.99E-06 |
| 67 | -1.35E-09 | 1.59E-09 | 2.36E-10 | 9.64E-12 | -5.33E-10 | 4.29E-07 | 8.44E-07 | 1.47E-06 | 1.95E-06 |
| 68 | -1.22E-09 | -5.66E-10 | -8.43E-10 | -2.86E-09 | 3.92E-10 | 4.82E-07 | 1.51E-06 | 3.25E-06 | 4.61E-06 |
| 69 | 1.59E-09 | 8.06E-10 | 2.40E-09 | -1.84E-09 | 2.66E-09 | 3.40E-07 | 8.04E-07 | 1.44E-06 | 1.77E-06 |
| 70 | 1.25E-09 | -1.64E-10 | 1.57E-09 | -7.03E-09 | 8.53E-09 | 3.29E-07 | 9.42E-07 | 1.84E-06 | 2.46E-06 |
| 71 | 9.92E-10 | -4.43E-10 | -1.20E-10 | -6.61E-10 | 1.33E-09 | 2.79E-07 | 7.48E-07 | 1.25E-06 | 1.47E-06 |
| 72 | -1.95E-09 | 1.20E-09 | 3.05E-10 | -3.21E-10 | 5.44E-10 | 2.50E-07 | 6.64E-07 | 1.22E-06 | 1.48E-06 |
| 73 | 1.96E-09 | 2.24E-10 | 1.37E-09 | -3.84E-09 | 6.26E-09 | 2.17E-07 | 6.32E-07 | 1.17E-06 | 1.44E-06 |
| 74 | 4.66E-10 | 3.96E-10 | 2.17E-09 | -2.35E-09 | 5.57E-10 | 1.86E-07 | 6.01E-07 | 1.13E-06 | 1.40E-06 |
| 75 | 3.19E-10 | 1.10E-09 | 1.25E-09 | -8.00E-10 | 1.35E-09 | 1.56E-07 | 5.76E-07 | 1.10E-06 | 1.37E-06 |
| 76 | 3.22E-10 | -5.86E-10 | -4.01E-11 | -7.55E-10 | -2.80E-10 | 1.28E-07 | 5.51E-07 | 1.08E-06 | 1.34E-06 |
| 77 | 4.65E-10 | 1.85E-09 | 2.33E-10 | -9.75E-11 | 5.22E-10 | 9.90E-08 | 5.29E-07 | 1.05E-06 | 1.30E-06 |
| 78 | 5.97E-10 | 1.44E-10 | 1.13E-09 | 3.36E-10 | 6.72E-10 | 7.33E-08 | 5.12E-07 | 1.01E-06 | 1.28E-06 |
| 79 | -1.37E-09 | -1.48E-09 | 1.67E-10 | 4.66E-10 | 6.95E-10 | 4.37E-08 | 4.88E-07 | 9.78E-07 | 1.25E-06 |
| 80 | -1.16E-09 | -1.18E-09 | 8.60E-10 | 3.42E-09 | -6.36E-10 | 1.63E-08 | 4.66E-07 | 9.42E-07 | 1.22E-06 |
| 81 | -9.07E-10 | -1.22E-09 | 1.17E-10 | 1.64E-10 | 4.09E-11 | -2.36E-10 | 4.39E-07 | 9.08E-07 | 1.20E-06 |
| 82 | 8.25E-10 | 1.23E-09 | -1.99E-10 | -7.77E-10 | -1.62E-09 | -5.54E-10 | 4.14E-07 | 8.76E-07 | 1.17E-06 |
| 83 | -1.07E-09 | 1.78E-09 | 1.15E-10 | 1.26E-10 | -2.59E-10 | -3.87E-10 | 3.88E-07 | 8.46E-07 | 1.14E-06 |
| 84 | -6.59E-10 | -1.06E-09 | -4.54E-09 | -1.05E-08 | -5.48E-09 | 6.76E-08 | 2.82E-07 | 8.40E-07 | 1.14E-06 |
| 85 | 8.81E-11 | 6.91E-11 | -3.56E-11 | -7.64E-11 | -3.04E-10 | -9.21E-11 | 3.41E-07 | 7.88E-07 | 1.09E-06 |
| 86 | -1.84E-09 | 4.77E-10 | -1.32E-08 | -1.75E-08 | -5.68E-09 | 8.51E-08 | 3.03E-07 | 7.49E-07 | 1.03E-06 |
| 87 | -3.13E-11 | -1.37E-09 | 2.26E-10 | 4.18E-10 | 1.90E-10 | -3.94E-10 | 2.93E-07 | 7.46E-07 | 1.04E-06 |
| 88 | 1.62E-09 | 1.50E-10 | -3.36E-09 | 3.19E-10 | -6.61E-11 | 3.04E-09 | 2.66E-07 | 7.28E-07 | 1.02E-06 |
| 89 | -1.30E-09 | 1.57E-09 | 6.69E-11 | 5.89E-11 | -3.39E-13 | -1.72E-10 | 2.43E-07 | 7.07E-07 | 9.91E-07 |
| 90 | 1.48E-09 | -1.18E-10 | 1.94E-11 | 3.43E-11 | -4.65E-11 | 1.16E-10 | 2.18E-07 | 6.87E-07 | 9.68E-07 |
| 91 | 8.77E-10 | -2.44E-10 | -1.05E-09 | -1.77E-09 | -2.33E-09 | 3.30E-09 | 1.95E-07 | 6.63E-07 | 9.44E-07 |
| 92 | 6.18E-10 | 1.19E-09 | 1.39E-11 | -1.08E-11 | -4.80E-11 | 5.20E-11 | 1.75E-07 | 6.44E-07 | 9.21E-07 |
| 93 | 1.81E-09 | 7.38E-10 | -6.71E-11 | -1.60E-12 | -2.25E-11 | -1.32E-11 | 1.56E-07 | 6.25E-07 | 8.96E-07 |
| 94 | -9.96E-10 | 6.19E-10 | 1.89E-10 | 3.65E-10 | -2.95E-10 | 3.33E-11 | 1.37E-07 | 6.03E-07 | 8.69E-07 |
| 95 | -9.99E-10 | 1.17E-10 | -1.38E-10 | 7.22E-11 | 9.38E-11 | 2.84E-11 | 1.16E-07 | 5.80E-07 | 8.41E-07 |
| 96 | 3.65E-10 | -1.67E-09 | -2.59E-10 | -3.99E-09 | -9.42E-09 | -5.91E-09 | 9.88E-08 | 5.60E-07 | 8.13E-07 |
| 97 | -4.89E-10 | 6.13E-10 | 1.70E-10 | 7.34E-10 | 4.56E-10 | 1.69E-09 | 7.58E-08 | 5.33E-07 | 7.89E-07 |
| 98 | 1.62E-09 | -6.89E-10 | 1.06E-10 | 1.13E-10 | -1.35E-10 | 1.94E-11 | 5.24E-08 | 5.08E-07 | 7.63E-07 |
| 99 | -1.48E-09 | -6.90E-10 | 1.44E-10 | 5.47E-11 | 6.49E-11 | 4.67E-11 | 3.10E-08 | 4.87E-07 | 7.42E-07 |
| 100 | -5.13E-10 | -1.18E-09 | -2.54E-11 | 2.42E-11 | -6.94E-12 | 5.05E-11 | 1.21E-08 | 4.70E-07 | 7.24E-07 |
| 101 | 7.34E-10 | 1.43E-09 | 1.17E-10 | -1.58E-10 | -4.34E-10 | -7.35E-10 | 1.24E-09 | 4.58E-07 | 7.10E-07 |
| 102 | -1.53E-09 | -1.39E-09 | 5.30E-13 | 6.14E-12 | 2.49E-11 | 6.67E-12 | -2.29E-11 | 4.41E-07 | 6.91E-07 |
| 103 | 4.75E-10 | -4.55E-10 | -1.59E-11 | 2.81E-11 | 7.89E-11 | 8.95E-11 | -4.05E-11 | 4.23E-07 | 6.73E-07 |
| 104 | 1.98E-09 | 1.16E-09 | 1.59E-11 | -3.23E-11 | -9.33E-11 | -1.72E-10 | -2.32E-11 | 4.06E-07 | 6.54E-07 |
| 105 | -7.10E-10 | 1.01E-10 | -2.96E-11 | 2.08E-11 | 9.69E-11 | 4.70E-11 | 3.89E-12 | 3.86E-07 | 6.34E-07 |
| 106 | -1.16E-09 | 1.97E-09 | 2.15E-11 | 1.23E-10 | 2.66E-10 | 1.73E-10 | 1.74E-10 | 3.66E-07 | 6.14E-07 |
| 107 | -1.29E-09 | 1.59E-09 | -9.98E-12 | -1.20E-11 | -3.21E-11 | -1.53E-10 | 1.25E-11 | 3.45E-07 | 5.96E-07 |
| 108 | -1.72E-09 | 6.60E-10 | 6.91E-12 | 1.15E-11 | 1.35E-11 | -2.36E-11 | 2.58E-11 | 3.31E-07 | 5.80E-07 |
| 109 | 6.99E-10 | -1.75E-09 | 1.39E-10 | -6.94E-11 | -3.28E-10 | -8.84E-10 | -4.45E-10 | 3.16E-07 | 5.65E-07 |
| 110 | 1.73E-09 | 1.37E-09 | 9.38E-12 | 2.70E-11 | 5.69E-11 | 7.88E-11 | -5.92E-11 | 3.04E-07 | 5.51E-07 |
| 111 | 1.27E-09 | 5.13E-10 | 3.24E-11 | 2.10E-11 | 2.85E-12 | -2.80E-11 | 4.52E-10 | 2.90E-07 | 5.37E-07 |
| 112 | 3.31E-10 | -1.91E-09 | -2.86E-12 | 1.41E-11 | 1.77E-11 | 1.98E-11 | 2.89E-10 | 2.75E-07 | 5.22E-07 |
| 113 | 2.60E-10 | -7.65E-10 | 2.91E-10 | 3.00E-10 | 1.94E-10 | -1.63E-10 | 1.37E-09 | 2.55E-07 | 5.03E-07 |
| 114 | 1.25E-09 | 1.53E-09 | 2.85E-11 | 4.89E-11 | 6.88E-11 | 1.34E-10 | 5.07E-10 | 2.33E-07 | 4.87E-07 |
| 115 | -1.16E-09 | 1.16E-09 | -3.28E-12 | 1.37E-11 | 3.09E-12 | -2.04E-11 | 2.43E-10 | 2.11E-07 | 4.65E-07 |
| 116 | -1.26E-09 | -1.69E-10 | -2.09E-11 | 5.68E-12 | 5.30E-11 | 1.15E-10 | 6.06E-12 | 1.92E-07 | 4.44E-07 |
| 117 | -6.70E-10 | 1.27E-09 | -2.26E-10 | -6.30E-11 | 4.53E-10 | 7.40E-10 | 5.86E-10 | 1.75E-07 | 4.23E-07 |
| 118 | 6.38E-11 | -6.22E-10 | -2.29E-11 | -3.88E-11 | -6.16E-11 | -9.34E-11 | 2.50E-10 | 1.61E-07 | 4.05E-07 |
| 119 | -4.91E-10 | 3.65E-10 | 8.35E-11 | 1.34E-10 | 1.59E-10 | -2.79E-11 | 2.50E-10 | 1.48E-07 | 3.89E-07 |
| 120 | 6.96E-10 | 5.02E-10 | -1.01E-13 | -1.05E-11 | -2.05E-11 | -5.09E-11 | 3.26E-11 | 1.37E-07 | 3.73E-07 |
| 121 | -7.01E-10 | 9.07E-10 | -5.15E-11 | 2.98E-11 | 2.64E-10 | 1.20E-10 | -2.43E-10 | 1.25E-07 | 3.60E-07 |
| 122 | 5.87E-10 | 1.96E-09 | -3.48E-11 | -3.60E-11 | 1.19E-10 | -2.78E-11 | -1.23E-10 | 1.13E-07 | 3.46E-07 |
| 123 | 5.02E-10 | -1.50E-09 | 1.25E-11 | -5.55E-11 | 2.97E-11 | 1.86E-11 | 1.01E-10 | 9.82E-08 | 3.34E-07 |
| 124 | 1.34E-09 | -6.04E-10 | -4.99E-12 | 1.07E-11 | -5.01E-12 | -1.56E-10 | 3.68E-10 | 8.40E-08 | 3.21E-07 |
| 125 | 5.92E-10 | 2.00E-09 | 1.00E-10 | 1.61E-10 | 1.78E-10 | 2.89E-11 | -1.01E-10 | 6.97E-08 | 3.09E-07 |
| 126 | -1.19E-09 | 1.77E-09 | -3.98E-12 | 1.62E-11 | 4.43E-11 | -6.55E-11 | 1.17E-10 | 5.61E-08 | 2.95E-07 |
| 127 | 1.63E-09 | -9.63E-10 | 1.00E-11 | 6.67E-12 | 3.56E-11 | 1.09E-10 | 6.59E-11 | 4.30E-08 | 2.81E-07 |
| 128 | 1.67E-09 | 6.33E-10 | -1.24E-11 | -1.12E-11 | -9.81E-12 | -6.22E-11 | 1.39E-11 | 3.06E-08 | 2.65E-07 |
| 129 | 1.15E-10 | 2.33E-10 | 1.66E-12 | 1.26E-11 | 2.54E-11 | -6.21E-12 | -3.41E-10 | 1.81E-08 | 2.50E-07 |
| 130 | 1.93E-09 | 1.94E-11 | 1.56E-11 | 2.11E-11 | 2.39E-11 | 1.10E-11 | -7.13E-11 | 4.61E-09 | 2.34E-07 |
| 131 | -1.67E-09 | 3.28E-10 | -3.25E-11 | 9.66E-10 | 1.37E-09 | 5.06E-10 | -1.03E-09 | 3.68E-09 | 2.20E-07 |
| 132 | -1.82E-09 | 1.73E-09 | 2.65E-11 | 1.52E-11 | 3.52E-12 | -2.63E-11 | -7.65E-11 | -1.21E-11 | 2.05E-07 |
| 133 | 1.64E-09 | -2.60E-10 | -6.33E-12 | -7.44E-12 | -5.63E-12 | 3.43E-12 | 8.40E-12 | -6.28E-11 | 1.92E-07 |
| 134 | 2.75E-10 | 8.30E-11 | 4.13E-13 | -1.63E-12 | -4.20E-12 | -3.60E-12 | 3.57E-12 | 7.10E-12 | 1.80E-07 |
| 135 | -2.35E-10 | -4.50E-10 | 7.54E-11 | 9.70E-11 | 1.31E-10 | 2.03E-10 | 1.84E-11 | -6.98E-11 | 1.69E-07 |
| 136 | -4.48E-10 | -8.58E-10 | -2.33E-11 | 4.54E-12 | 1.88E-11 | -5.80E-11 | -2.98E-10 | -6.40E-11 | 1.57E-07 |
| 137 | -2.57E-10 | 1.55E-10 | -1.68E-11 | -1.34E-11 | -8.76E-12 | -6.83E-12 | -3.99E-11 | 6.33E-11 | 1.45E-07 |
| 138 | -1.93E-09 | -2.34E-10 | -2.65E-12 | -4.01E-12 | -6.10E-12 | -1.20E-11 | -2.92E-11 | -6.67E-11 | 1.33E-07 |
| 139 | 1.69E-10 | -1.20E-09 | 1.68E-12 | 4.92E-12 | 3.78E-12 | -1.04E-11 | -3.18E-11 | 9.31E-12 | 1.21E-07 |
| 140 | -9.63E-11 | -9.81E-10 | -1.40E-12 | -3.40E-12 | -4.60E-12 | -4.65E-12 | 4.57E-13 | -9.93E-11 | 1.09E-07 |
| 141 | -7.21E-10 | 1.42E-09 | -1.36E-12 | -7.39E-13 | -6.49E-13 | -2.30E-12 | -9.26E-12 | 2.82E-12 | 9.81E-08 |
| 142 | -6.05E-11 | -3.03E-11 | -1.75E-13 | -4.59E-13 | -4.38E-13 | 2.58E-12 | -4.82E-13 | -1.99E-11 | 8.76E-08 |
| 143 | -7.42E-10 | -4.49E-10 | 1.62E-12 | 8.68E-13 | -3.43E-13 | -4.08E-12 | -1.54E-11 | 9.55E-11 | 7.75E-08 |
| 144 | -1.68E-09 | -1.38E-09 | -8.67E-12 | -8.99E-12 | -6.17E-12 | 2.87E-12 | -1.72E-11 | -2.93E-12 | 6.79E-08 |
| 145 | 1.11E-09 | -5.26E-10 | 9.20E-13 | 1.16E-12 | 9.57E-13 | -1.28E-12 | -9.10E-12 | 3.93E-12 | 5.83E-08 |
| 146 | -5.57E-10 | 1.53E-09 | 1.51E-12 | 2.10E-12 | 2.73E-12 | 4.20E-12 | -1.05E-12 | -1.46E-11 | 4.82E-08 |
| 147 | 9.92E-10 | 3.88E-10 | 1.17E-11 | 2.66E-11 | 3.89E-11 | 4.59E-11 | -2.30E-11 | -2.44E-11 | 3.78E-08 |
| 148 | 1.47E-09 | 1.70E-09 | 1.45E-13 | 1.28E-13 | 1.49E-13 | 4.25E-13 | -2.06E-13 | -1.76E-12 | 2.69E-08 |
| 149 | -6.77E-10 | 5.99E-10 | -1.44E-12 | -2.37E-12 | -3.80E-12 | -7.90E-12 | -1.36E-11 | -2.97E-12 | 1.58E-08 |
| 150 | 6.01E-10 | 1.39E-09 | 1.92E-14 | -3.87E-14 | -1.01E-13 | -1.43E-13 | 1.95E-13 | -1.26E-12 | 3.75E-09 |

Table.15. Spectra predicted by MLP neural network: Be- 1.2mm

| energy bin (kev)/voltage(kv) | 20kv | 30kv | 40kv | 50kv | 60kv | 80kv | 100kv | 130kv | 150kv |
| --- | --- | --- | --- | --- | --- | --- | --- | --- | --- |
| 1 | 0 | 0 | 0 | 0 | 0 | 0 | 0 | 0 | 0 |
| 2 | -1.64E-09 | -3.65E-10 | -2.05E-14 | -2.60E-14 | -3.77E-14 | -1.15E-13 | -8.54E-14 | 8.60E-14 | 1.14E-13 |
| 3 | -3.92E-10 | 1.78E-10 | 1.53E-10 | 1.53E-10 | 1.47E-10 | 1.61E-10 | 3.21E-10 | 2.37E-11 | 6.00E-11 |
| 4 | -1.28E-09 | 8.51E-10 | -4.39E-09 | -1.11E-08 | -1.58E-08 | -1.90E-08 | -1.44E-08 | 1.89E-09 | -2.31E-09 |
| 5 | -9.53E-10 | 4.23E-10 | 3.97E-08 | 4.52E-08 | 3.94E-08 | 1.26E-08 | -3.51E-09 | 1.90E-08 | 1.12E-08 |
| 6 | -1.28E-09 | -4.13E-10 | 1.41E-08 | 1.28E-08 | 1.13E-08 | 5.92E-09 | 7.58E-10 | -1.88E-09 | 1.90E-09 |
| 7 | 1.85E-09 | 1.96E-09 | -2.91E-08 | -3.09E-08 | -1.84E-08 | 5.17E-09 | 4.57E-08 | -5.88E-08 | -4.29E-08 |
| 8 | -1.34E-09 | 1.95E-09 | 2.68E-08 | 1.17E-08 | -1.36E-08 | -6.30E-08 | -3.21E-08 | 1.04E-08 | -4.57E-08 |
| 9 | -4.98E-10 | 1.00E-09 | 5.35E-08 | 6.47E-08 | 5.81E-08 | 1.07E-08 | 6.28E-08 | 2.30E-08 | -4.41E-08 |
| 10 | 6.96E-09 | 1.13E-08 | 3.93E-09 | -1.98E-09 | -3.06E-08 | -1.04E-07 | -7.89E-08 | 1.78E-07 | -4.41E-08 |
| 11 | 1.47E-08 | 1.98E-08 | 2.48E-08 | 3.21E-08 | 3.60E-08 | 3.00E-08 | 3.18E-08 | -9.82E-09 | 1.25E-08 |
| 12 | 2.57E-08 | 4.17E-08 | 6.69E-08 | 7.43E-08 | 7.23E-08 | 6.58E-08 | 7.52E-08 | 1.01E-07 | 4.80E-08 |
| 13 | 4.57E-08 | 7.98E-08 | 1.13E-07 | 1.50E-07 | 1.80E-07 | 8.63E-08 | 7.86E-08 | 1.07E-07 | -8.61E-08 |
| 14 | 8.71E-08 | 1.69E-07 | 3.07E-07 | 2.86E-07 | 2.54E-07 | 2.64E-07 | 3.00E-07 | 2.05E-07 | 2.04E-07 |
| 15 | 1.41E-07 | 3.05E-07 | 4.51E-07 | 5.24E-07 | 5.36E-07 | 6.57E-07 | 6.53E-07 | 6.66E-07 | 6.11E-07 |
| 16 | 1.44E-07 | 3.66E-07 | 5.60E-07 | 6.45E-07 | 6.87E-07 | 7.43E-07 | 9.59E-07 | 1.03E-06 | 7.75E-07 |
| 17 | 1.45E-07 | 4.39E-07 | 6.44E-07 | 8.07E-07 | 9.47E-07 | 1.10E-06 | 1.20E-06 | 1.35E-06 | 1.19E-06 |
| 18 | 1.20E-07 | 4.86E-07 | 7.48E-07 | 9.44E-07 | 1.10E-06 | 1.34E-06 | 1.48E-06 | 1.70E-06 | 1.52E-06 |
| 19 | 9.68E-08 | 5.18E-07 | 8.40E-07 | 1.08E-06 | 1.27E-06 | 1.54E-06 | 1.76E-06 | 2.05E-06 | 1.88E-06 |
| 20 | 4.91E-08 | 5.30E-07 | 8.87E-07 | 1.15E-06 | 1.37E-06 | 1.74E-06 | 2.02E-06 | 2.36E-06 | 2.22E-06 |
| 21 | 3.63E-11 | 5.24E-07 | 9.03E-07 | 1.20E-06 | 1.47E-06 | 1.89E-06 | 2.20E-06 | 2.61E-06 | 2.49E-06 |
| 22 | -8.96E-10 | 5.04E-07 | 9.09E-07 | 1.25E-06 | 1.55E-06 | 2.00E-06 | 2.36E-06 | 2.84E-06 | 2.74E-06 |
| 23 | 1.25E-09 | 4.61E-07 | 8.88E-07 | 1.24E-06 | 1.54E-06 | 2.05E-06 | 2.45E-06 | 2.98E-06 | 2.98E-06 |
| 24 | -8.33E-10 | 4.16E-07 | 8.51E-07 | 1.24E-06 | 1.56E-06 | 2.09E-06 | 2.53E-06 | 3.10E-06 | 3.10E-06 |
| 25 | 5.56E-10 | 3.71E-07 | 7.93E-07 | 1.21E-06 | 1.56E-06 | 2.13E-06 | 2.61E-06 | 3.24E-06 | 3.24E-06 |
| 26 | -5.07E-10 | 3.20E-07 | 7.61E-07 | 1.21E-06 | 1.59E-06 | 2.17E-06 | 2.69E-06 | 3.37E-06 | 3.45E-06 |
| 27 | 5.59E-10 | 2.59E-07 | 7.25E-07 | 1.17E-06 | 1.57E-06 | 2.19E-06 | 2.77E-06 | 3.49E-06 | 3.60E-06 |
| 28 | 5.96E-10 | 1.97E-07 | 7.12E-07 | 1.20E-06 | 1.59E-06 | 2.23E-06 | 2.83E-06 | 3.61E-06 | 3.76E-06 |
| 29 | 4.56E-10 | 1.30E-07 | 6.76E-07 | 1.14E-06 | 1.52E-06 | 2.24E-06 | 2.86E-06 | 3.68E-06 | 3.90E-06 |
| 30 | 3.96E-11 | 5.23E-08 | 5.43E-07 | 1.03E-06 | 1.50E-06 | 2.29E-06 | 2.94E-06 | 3.68E-06 | 3.98E-06 |
| 31 | -1.67E-09 | -1.71E-09 | 5.03E-07 | 9.18E-07 | 1.37E-06 | 2.24E-06 | 2.93E-06 | 3.76E-06 | 4.34E-06 |
| 32 | 1.61E-10 | 1.38E-09 | 5.31E-07 | 1.00E-06 | 1.40E-06 | 2.15E-06 | 2.85E-06 | 3.69E-06 | 4.05E-06 |
| 33 | 7.98E-10 | -1.16E-09 | 4.66E-07 | 9.40E-07 | 1.35E-06 | 2.10E-06 | 2.78E-06 | 3.59E-06 | 4.03E-06 |
| 34 | -1.75E-09 | -1.32E-10 | 4.02E-07 | 8.86E-07 | 1.28E-06 | 2.02E-06 | 2.72E-06 | 3.53E-06 | 3.99E-06 |
| 35 | -1.10E-09 | -5.26E-10 | 2.85E-07 | 8.22E-07 | 1.23E-06 | 1.94E-06 | 2.66E-06 | 3.45E-06 | 3.95E-06 |
| 36 | -1.56E-09 | 7.34E-10 | 2.60E-07 | 7.78E-07 | 1.17E-06 | 1.87E-06 | 2.59E-06 | 3.42E-06 | 3.91E-06 |
| 37 | 1.49E-09 | -1.86E-09 | 2.67E-07 | 6.75E-07 | 1.12E-06 | 1.81E-06 | 2.54E-06 | 3.41E-06 | 3.85E-06 |
| 38 | 6.61E-10 | -1.17E-09 | 1.65E-07 | 6.58E-07 | 1.06E-06 | 1.74E-06 | 2.44E-06 | 3.33E-06 | 3.80E-06 |
| 39 | 2.48E-10 | -1.28E-09 | 1.01E-07 | 6.05E-07 | 1.01E-06 | 1.69E-06 | 2.38E-06 | 3.26E-06 | 3.72E-06 |
| 40 | -8.44E-10 | 9.76E-10 | 4.29E-08 | 5.43E-07 | 9.48E-07 | 1.62E-06 | 2.31E-06 | 3.19E-06 | 3.68E-06 |
| 41 | 2.77E-10 | 7.53E-10 | 7.42E-09 | 4.93E-07 | 8.92E-07 | 1.58E-06 | 2.26E-06 | 3.11E-06 | 3.61E-06 |
| 42 | 9.61E-10 | -7.72E-11 | -5.81E-09 | 4.32E-07 | 8.45E-07 | 1.54E-06 | 2.18E-06 | 3.05E-06 | 3.53E-06 |
| 43 | -8.79E-11 | 1.44E-09 | -2.55E-12 | 3.79E-07 | 8.00E-07 | 1.47E-06 | 2.11E-06 | 2.96E-06 | 3.46E-06 |
| 44 | -1.06E-10 | -1.69E-09 | 6.89E-09 | 3.46E-07 | 7.30E-07 | 1.42E-06 | 2.03E-06 | 2.87E-06 | 3.38E-06 |
| 45 | 1.21E-09 | 3.01E-11 | -5.38E-09 | 2.82E-07 | 6.82E-07 | 1.36E-06 | 1.98E-06 | 2.81E-06 | 3.30E-06 |
| 46 | -1.34E-09 | -1.49E-09 | -3.11E-09 | 2.35E-07 | 6.38E-07 | 1.30E-06 | 1.91E-06 | 2.74E-06 | 3.23E-06 |
| 47 | -1.86E-09 | -1.46E-09 | 3.15E-09 | 1.86E-07 | 5.93E-07 | 1.24E-06 | 1.86E-06 | 2.66E-06 | 3.16E-06 |
| 48 | 8.53E-10 | 1.57E-09 | -7.47E-10 | 1.36E-07 | 5.44E-07 | 1.19E-06 | 1.81E-06 | 2.59E-06 | 3.09E-06 |
| 49 | -3.45E-10 | 1.74E-09 | -3.55E-08 | 1.36E-07 | 4.66E-07 | 1.15E-06 | 1.72E-06 | 2.52E-06 | 3.02E-06 |
| 50 | 1.51E-09 | -8.21E-10 | 5.42E-10 | 3.66E-08 | 4.56E-07 | 1.09E-06 | 1.67E-06 | 2.45E-06 | 2.95E-06 |
| 51 | 1.65E-09 | 1.94E-09 | -7.21E-09 | 3.20E-08 | 4.02E-07 | 1.06E-06 | 1.57E-06 | 2.37E-06 | 2.87E-06 |
| 52 | 1.42E-09 | -3.67E-10 | 8.78E-09 | 7.93E-08 | 2.86E-07 | 9.86E-07 | 1.55E-06 | 2.31E-06 | 2.82E-06 |
| 53 | -4.28E-10 | 1.71E-09 | -2.37E-08 | 3.43E-08 | 3.11E-07 | 9.30E-07 | 1.52E-06 | 2.27E-06 | 2.74E-06 |
| 54 | -8.43E-10 | 1.98E-10 | -1.53E-08 | 4.15E-08 | 2.62E-07 | 9.07E-07 | 1.48E-06 | 2.23E-06 | 2.69E-06 |
| 55 | -1.70E-10 | 5.57E-11 | -5.71E-09 | 6.07E-09 | 2.26E-07 | 8.41E-07 | 1.43E-06 | 2.16E-06 | 2.63E-06 |
| 56 | 7.48E-10 | -5.75E-10 | -2.36E-08 | 3.19E-08 | 2.37E-07 | 8.11E-07 | 1.37E-06 | 2.09E-06 | 2.51E-06 |
| 57 | -1.93E-09 | 1.82E-09 | -5.29E-09 | 5.14E-09 | 1.46E-07 | 7.60E-07 | 1.32E-06 | 2.03E-06 | 2.52E-06 |
| 58 | -4.66E-10 | 1.09E-09 | -7.68E-09 | -7.75E-09 | 1.15E-07 | 7.03E-07 | 1.23E-06 | 1.94E-06 | 2.47E-06 |
| 59 | 2.75E-11 | 1.05E-09 | 1.25E-09 | -3.40E-09 | 6.53E-08 | 8.30E-07 | 2.20E-06 | 4.74E-06 | 6.41E-06 |
| 60 | 4.35E-10 | 1.36E-09 | 1.54E-08 | -1.22E-08 | 4.07E-08 | 9.12E-07 | 2.91E-06 | 6.97E-06 | 9.68E-06 |
| 61 | 2.10E-10 | 5.53E-10 | 9.50E-09 | -2.01E-08 | 2.34E-08 | 5.90E-07 | 1.07E-06 | 1.81E-06 | 2.34E-06 |
| 62 | 1.27E-09 | -9.31E-10 | -9.92E-10 | -2.65E-10 | 3.04E-11 | 5.83E-07 | 1.17E-06 | 1.90E-06 | 2.37E-06 |
| 63 | 6.39E-10 | -1.44E-09 | 2.46E-09 | 4.20E-11 | 4.18E-09 | 5.86E-07 | 1.03E-06 | 1.59E-06 | 2.05E-06 |
| 64 | 1.06E-09 | 1.54E-09 | 6.74E-10 | -2.61E-12 | 3.37E-10 | 5.05E-07 | 1.00E-06 | 1.52E-06 | 1.99E-06 |
| 65 | 9.65E-10 | -1.75E-09 | 3.90E-10 | 3.92E-10 | 5.89E-11 | 4.70E-07 | 9.58E-07 | 1.52E-06 | 1.98E-06 |
| 66 | -1.49E-10 | -2.82E-10 | -1.48E-08 | -2.96E-08 | 3.30E-08 | 4.10E-07 | 8.94E-07 | 1.50E-06 | 1.96E-06 |
| 67 | -1.50E-09 | 9.74E-10 | -4.73E-10 | -7.14E-10 | -2.13E-09 | 4.19E-07 | 8.31E-07 | 1.45E-06 | 1.91E-06 |
| 68 | 1.07E-09 | 1.48E-09 | -2.52E-09 | -3.49E-09 | -3.94E-10 | 4.76E-07 | 1.49E-06 | 3.19E-06 | 4.52E-06 |
| 69 | -9.14E-10 | 7.40E-10 | 8.55E-10 | -3.68E-09 | 5.21E-10 | 3.32E-07 | 7.89E-07 | 1.41E-06 | 1.74E-06 |
| 70 | 1.11E-09 | 8.78E-10 | 1.98E-09 | -4.59E-09 | 8.77E-09 | 3.21E-07 | 9.33E-07 | 1.81E-06 | 2.42E-06 |
| 71 | -3.06E-10 | 1.01E-09 | -1.28E-09 | -1.06E-09 | 4.00E-10 | 2.73E-07 | 7.34E-07 | 1.22E-06 | 1.44E-06 |
| 72 | 9.74E-10 | -1.73E-09 | 1.96E-10 | -3.87E-10 | 4.77E-10 | 2.45E-07 | 6.53E-07 | 1.20E-06 | 1.45E-06 |
| 73 | -1.13E-09 | -1.73E-09 | 3.14E-09 | -3.06E-09 | 5.55E-09 | 2.12E-07 | 6.23E-07 | 1.15E-06 | 1.42E-06 |
| 74 | -8.24E-10 | 3.60E-10 | 1.75E-09 | -2.33E-09 | 7.58E-10 | 1.83E-07 | 5.92E-07 | 1.11E-06 | 1.38E-06 |
| 75 | -1.61E-09 | -7.42E-10 | 5.22E-10 | -1.74E-09 | 2.47E-10 | 1.54E-07 | 5.67E-07 | 1.09E-06 | 1.35E-06 |
| 76 | 1.38E-09 | 1.91E-09 | 3.55E-10 | -5.25E-10 | -3.90E-10 | 1.25E-07 | 5.42E-07 | 1.06E-06 | 1.31E-06 |
| 77 | -1.99E-09 | 5.46E-10 | 5.31E-10 | 2.44E-10 | 8.60E-10 | 9.74E-08 | 5.21E-07 | 1.03E-06 | 1.28E-06 |
| 78 | -7.68E-10 | -1.52E-09 | -4.59E-10 | -3.13E-10 | 1.12E-09 | 7.21E-08 | 5.06E-07 | 1.00E-06 | 1.26E-06 |
| 79 | 5.52E-10 | 1.18E-09 | 1.44E-10 | 6.23E-10 | 1.16E-09 | 4.29E-08 | 4.81E-07 | 9.63E-07 | 1.23E-06 |
| 80 | -1.13E-10 | -9.76E-10 | -1.52E-10 | 2.21E-09 | -8.85E-10 | 1.61E-08 | 4.58E-07 | 9.30E-07 | 1.21E-06 |
| 81 | 7.83E-10 | 1.68E-09 | 1.63E-10 | 2.25E-10 | 3.99E-11 | -5.59E-10 | 4.32E-07 | 8.94E-07 | 1.18E-06 |
| 82 | 1.61E-09 | -3.92E-10 | 4.20E-10 | 2.33E-11 | -7.58E-10 | -2.55E-10 | 4.09E-07 | 8.64E-07 | 1.15E-06 |
| 83 | -2.54E-10 | -6.73E-10 | 1.11E-10 | 1.17E-10 | -2.18E-10 | -3.10E-10 | 3.83E-07 | 8.33E-07 | 1.13E-06 |
| 84 | -1.91E-09 | -1.99E-09 | -5.02E-09 | -1.60E-09 | 1.30E-08 | 9.76E-08 | 3.06E-07 | 8.36E-07 | 1.12E-06 |
| 85 | 4.28E-10 | 4.69E-10 | -2.21E-10 | 1.19E-10 | 2.23E-10 | -4.59E-11 | 3.37E-07 | 7.79E-07 | 1.08E-06 |
| 86 | 8.51E-10 | -2.12E-10 | -5.37E-09 | -1.71E-08 | -1.17E-08 | 7.76E-08 | 2.99E-07 | 7.35E-07 | 1.02E-06 |
| 87 | -2.68E-10 | -2.25E-10 | 4.75E-12 | 3.62E-10 | 3.55E-10 | 3.43E-10 | 2.89E-07 | 7.37E-07 | 1.03E-06 |
| 88 | -1.65E-09 | -1.15E-09 | -2.26E-09 | -2.84E-10 | -2.98E-09 | -2.71E-09 | 2.64E-07 | 7.18E-07 | 1.00E-06 |
| 89 | 1.08E-09 | 1.45E-09 | 3.79E-11 | 1.90E-11 | -2.89E-11 | -2.20E-10 | 2.39E-07 | 6.97E-07 | 9.77E-07 |
| 90 | -1.65E-09 | 3.03E-10 | -6.31E-11 | -3.01E-11 | -1.01E-10 | -8.53E-11 | 2.15E-07 | 6.78E-07 | 9.55E-07 |
| 91 | 2.91E-10 | 7.90E-11 | -4.88E-10 | -1.43E-09 | -2.15E-09 | 2.81E-09 | 1.92E-07 | 6.53E-07 | 9.32E-07 |
| 92 | -7.93E-10 | -1.22E-09 | 3.30E-11 | 3.29E-11 | 5.43E-12 | 3.69E-11 | 1.73E-07 | 6.36E-07 | 9.09E-07 |
| 93 | -7.69E-10 | -1.16E-09 | -5.13E-11 | 1.02E-12 | 2.45E-11 | -9.30E-11 | 1.54E-07 | 6.16E-07 | 8.84E-07 |
| 94 | 4.17E-10 | -9.45E-10 | 1.47E-10 | 3.53E-10 | -2.58E-10 | -9.77E-11 | 1.35E-07 | 5.95E-07 | 8.58E-07 |
| 95 | -9.66E-10 | 1.34E-09 | -7.68E-11 | 1.00E-10 | 1.80E-10 | 1.86E-10 | 1.15E-07 | 5.72E-07 | 8.30E-07 |
| 96 | -5.60E-10 | -1.83E-09 | -1.25E-10 | -3.24E-09 | -8.07E-09 | -6.31E-09 | 9.14E-08 | 5.53E-07 | 8.10E-07 |
| 97 | -1.67E-09 | -1.33E-09 | 3.93E-10 | 5.85E-10 | 9.12E-11 | 1.31E-09 | 7.47E-08 | 5.27E-07 | 7.80E-07 |
| 98 | -1.69E-09 | 6.08E-10 | 9.82E-11 | 1.11E-10 | -1.07E-10 | 1.57E-10 | 5.16E-08 | 5.02E-07 | 7.53E-07 |
| 99 | -1.50E-10 | -1.38E-09 | 1.77E-11 | -5.58E-11 | 3.23E-11 | 3.29E-11 | 3.02E-08 | 4.80E-07 | 7.32E-07 |
| 100 | -1.49E-09 | 1.96E-09 | -1.17E-11 | 3.83E-11 | -1.32E-12 | 1.16E-11 | 1.19E-08 | 4.64E-07 | 7.16E-07 |
| 101 | 8.81E-10 | 8.78E-10 | -1.14E-10 | -3.56E-10 | -5.54E-10 | -4.48E-10 | 1.19E-09 | 4.53E-07 | 7.00E-07 |
| 102 | -7.66E-10 | -1.70E-09 | -1.99E-12 | -1.96E-13 | 1.43E-11 | -2.17E-12 | -1.42E-11 | 4.36E-07 | 6.82E-07 |
| 103 | -4.83E-10 | 2.41E-10 | -2.88E-11 | -8.15E-12 | 1.59E-11 | -8.15E-12 | -5.75E-11 | 4.18E-07 | 6.65E-07 |
| 104 | -9.34E-10 | -5.10E-10 | 9.32E-11 | 5.73E-11 | 1.16E-11 | -4.92E-11 | 2.57E-11 | 4.01E-07 | 6.46E-07 |
| 105 | -1.38E-09 | 1.25E-09 | -4.54E-11 | -3.57E-11 | 1.83E-11 | -4.05E-12 | -2.47E-11 | 3.81E-07 | 6.26E-07 |
| 106 | -1.27E-09 | -7.79E-10 | -1.29E-10 | -4.65E-11 | 7.06E-11 | 7.66E-11 | -1.03E-10 | 3.61E-07 | 6.07E-07 |
| 107 | 1.79E-09 | 1.92E-09 | -8.84E-12 | 1.84E-11 | 4.95E-11 | 1.72E-11 | 5.50E-11 | 3.41E-07 | 5.89E-07 |
| 108 | -1.39E-10 | 6.87E-10 | 7.60E-12 | 1.51E-11 | 2.20E-11 | -2.63E-12 | 1.13E-11 | 3.27E-07 | 5.73E-07 |
| 109 | 7.10E-10 | 1.60E-09 | 6.69E-11 | -1.56E-11 | -1.94E-10 | -7.59E-10 | -5.96E-10 | 3.12E-07 | 5.58E-07 |
| 110 | 5.79E-10 | -5.33E-10 | -1.63E-12 | 6.15E-12 | 1.65E-11 | -2.91E-11 | -1.89E-10 | 3.00E-07 | 5.45E-07 |
| 111 | 1.15E-09 | -1.68E-09 | -6.43E-12 | -3.85E-13 | -3.36E-12 | -2.66E-11 | 3.04E-10 | 2.86E-07 | 5.31E-07 |
| 112 | -7.15E-11 | 7.97E-10 | 6.98E-12 | 2.26E-11 | 2.35E-11 | 1.07E-11 | 2.85E-10 | 2.71E-07 | 5.16E-07 |
| 113 | -1.49E-09 | -9.72E-10 | 6.00E-10 | 4.72E-10 | 2.54E-10 | -2.81E-10 | 9.73E-10 | 2.51E-07 | 4.99E-07 |
| 114 | -1.93E-09 | 1.06E-09 | -9.29E-12 | -2.00E-12 | -1.04E-11 | -1.11E-10 | -2.40E-12 | 2.30E-07 | 4.82E-07 |
| 115 | 1.95E-09 | 1.07E-09 | -3.76E-11 | -7.93E-12 | -1.00E-11 | -2.34E-11 | 2.05E-10 | 2.09E-07 | 4.60E-07 |
| 116 | 1.73E-09 | -1.65E-09 | -1.34E-11 | 3.58E-12 | 2.12E-11 | 8.24E-12 | 1.54E-11 | 1.90E-07 | 4.39E-07 |
| 117 | -1.73E-09 | 1.65E-09 | -1.37E-10 | -4.29E-12 | 3.37E-10 | 1.33E-10 | 5.18E-10 | 1.73E-07 | 4.18E-07 |
| 118 | 6.73E-10 | -1.65E-09 | -2.83E-11 | -3.92E-11 | -4.65E-11 | -4.93E-11 | 4.94E-11 | 1.59E-07 | 4.00E-07 |
| 119 | -2.52E-10 | -9.99E-10 | 5.41E-11 | 4.93E-11 | 3.35E-11 | -5.66E-11 | 2.88E-10 | 1.46E-07 | 3.84E-07 |
| 120 | -1.89E-10 | -1.09E-09 | 1.54E-11 | 3.75E-12 | -2.21E-12 | 1.14E-11 | 1.20E-10 | 1.35E-07 | 3.69E-07 |
| 121 | 1.44E-09 | -6.25E-10 | -1.39E-10 | -9.55E-11 | 1.07E-10 | -1.93E-11 | -3.71E-10 | 1.23E-07 | 3.56E-07 |
| 122 | 1.54E-10 | -1.75E-09 | -5.22E-11 | -6.80E-11 | 8.97E-11 | -1.45E-10 | -2.25E-10 | 1.11E-07 | 3.43E-07 |
| 123 | -6.38E-10 | 3.40E-10 | 5.37E-11 | -1.88E-11 | 5.88E-11 | 3.31E-11 | 2.26E-11 | 9.71E-08 | 3.30E-07 |
| 124 | -1.38E-09 | -4.06E-10 | 2.69E-13 | -1.10E-11 | -2.50E-11 | -7.51E-11 | 8.52E-10 | 8.31E-08 | 3.18E-07 |
| 125 | -8.38E-10 | -1.52E-09 | 1.05E-11 | 5.79E-12 | -1.78E-11 | -1.68E-10 | -9.89E-11 | 6.95E-08 | 3.05E-07 |
| 126 | 4.23E-12 | 6.39E-10 | -3.62E-11 | -3.17E-11 | -9.37E-12 | -8.74E-11 | 3.93E-11 | 5.54E-08 | 2.92E-07 |
| 127 | -1.66E-09 | 6.84E-10 | -7.85E-13 | -1.98E-11 | -2.04E-11 | -9.88E-12 | -3.16E-12 | 4.28E-08 | 2.78E-07 |
| 128 | -3.91E-10 | -2.93E-10 | 6.76E-12 | 1.49E-11 | 1.74E-11 | -6.38E-11 | -1.82E-11 | 3.03E-08 | 2.62E-07 |
| 129 | -1.84E-09 | -8.92E-10 | 2.04E-12 | 1.11E-11 | 2.38E-11 | 2.62E-11 | -1.09E-10 | 1.79E-08 | 2.47E-07 |
| 130 | -5.07E-10 | 7.31E-10 | 1.32E-11 | 1.75E-11 | 1.79E-11 | -2.56E-11 | -5.13E-11 | 4.61E-09 | 2.31E-07 |
| 131 | -7.94E-10 | -1.78E-09 | -5.47E-12 | 9.66E-10 | 1.31E-09 | 2.36E-10 | -1.56E-09 | 2.63E-09 | 2.16E-07 |
| 132 | -3.96E-10 | -8.46E-10 | 1.28E-11 | -4.56E-12 | -2.42E-11 | -6.52E-11 | -1.03E-10 | 1.13E-10 | 2.03E-07 |
| 133 | 1.75E-09 | 1.39E-09 | -7.31E-12 | -4.61E-12 | -1.80E-12 | 4.26E-12 | -6.27E-12 | -8.55E-11 | 1.90E-07 |
| 134 | -8.91E-10 | 1.16E-10 | 9.60E-13 | -8.17E-13 | -3.31E-12 | -3.35E-12 | 1.44E-12 | 2.34E-12 | 1.78E-07 |
| 135 | -6.22E-10 | 1.54E-09 | 2.07E-11 | 2.52E-11 | 3.92E-11 | 1.20E-10 | 1.34E-10 | -9.83E-11 | 1.66E-07 |
| 136 | 1.61E-10 | 6.50E-10 | -2.52E-11 | -6.79E-12 | 1.21E-11 | -1.27E-11 | -2.06E-10 | -1.36E-10 | 1.55E-07 |
| 137 | 5.54E-12 | -5.81E-10 | -1.84E-11 | -2.93E-11 | -3.74E-11 | -4.08E-11 | -6.51E-11 | -5.76E-13 | 1.43E-07 |
| 138 | -1.34E-09 | 1.66E-09 | -2.40E-12 | -3.74E-12 | -5.75E-12 | -1.12E-11 | -2.77E-11 | -6.23E-11 | 1.32E-07 |
| 139 | 1.73E-09 | 6.06E-10 | -6.05E-13 | -2.49E-12 | -4.14E-12 | -6.45E-12 | -7.04E-12 | 3.82E-11 | 1.20E-07 |
| 140 | -1.90E-09 | 1.29E-09 | 1.39E-12 | 7.16E-13 | 1.08E-13 | -1.22E-12 | -7.04E-12 | -2.09E-11 | 1.08E-07 |
| 141 | -4.90E-10 | -5.42E-10 | -1.90E-12 | -1.65E-12 | -1.84E-13 | 4.61E-12 | 3.53E-12 | 1.26E-13 | 9.71E-08 |
| 142 | -4.23E-10 | -1.49E-10 | -9.07E-13 | -1.13E-12 | -3.33E-13 | 3.79E-12 | -1.55E-12 | -5.26E-11 | 8.67E-08 |
| 143 | -1.49E-09 | 6.86E-10 | 1.67E-12 | 2.01E-12 | 1.78E-12 | -7.53E-13 | -1.32E-11 | 9.33E-11 | 7.67E-08 |
| 144 | -6.88E-10 | -2.32E-10 | -5.35E-12 | -3.94E-12 | 1.69E-13 | 9.55E-12 | -1.33E-11 | -8.82E-12 | 6.72E-08 |
| 145 | 1.16E-09 | -1.49E-09 | 6.15E-12 | 7.18E-12 | 6.38E-12 | -1.19E-12 | -1.91E-11 | -2.39E-11 | 5.75E-08 |
| 146 | 1.12E-09 | -1.03E-09 | -8.88E-13 | 2.95E-13 | 1.50E-12 | 3.80E-12 | -9.10E-13 | -1.17E-11 | 4.77E-08 |
| 147 | 1.17E-09 | -8.90E-10 | -4.39E-12 | 5.81E-12 | 1.97E-11 | 4.47E-11 | -1.92E-11 | -2.96E-11 | 3.75E-08 |
| 148 | 7.92E-10 | -9.35E-10 | -8.35E-14 | -5.94E-14 | 6.64E-15 | 4.19E-13 | -4.76E-14 | -1.00E-12 | 2.66E-08 |
| 149 | 5.47E-10 | 6.73E-10 | 3.47E-13 | -1.64E-12 | -3.19E-12 | -5.14E-12 | -6.79E-12 | 1.09E-11 | 1.57E-08 |
| 150 | -1.70E-09 | -1.68E-09 | -3.09E-13 | -2.95E-13 | -3.09E-13 | -1.81E-13 | 7.92E-13 | 7.56E-14 | 3.71E-09 |

Table.16. Spectra predicted by MLP neural network: Be- 1.6mm

| energy bin (kev)/voltage(kv) | 20kv | 30kv | 40kv | 50kv | 60kv | 80kv | 100kv | 130kv | 150kv |
| --- | --- | --- | --- | --- | --- | --- | --- | --- | --- |
| 1 | 0 | 0 | 0 | 0 | 0 | 0 | 0 | 0 | 0 |
| 2 | -1.24E-10 | -1.58E-09 | -4.64E-15 | -2.36E-15 | -1.04E-14 | -1.10E-13 | -8.99E-14 | 8.38E-14 | 2.62E-13 |
| 3 | -3.72E-11 | -1.88E-09 | 3.89E-12 | -2.14E-12 | -8.97E-12 | 1.68E-11 | 1.96E-10 | -5.41E-12 | -3.39E-11 |
| 4 | 3.43E-11 | 1.36E-09 | -1.39E-08 | -1.42E-08 | -1.12E-08 | -1.70E-09 | 7.70E-09 | 1.50E-08 | -2.89E-09 |
| 5 | -1.71E-10 | -1.25E-09 | 4.47E-08 | 4.59E-08 | 3.07E-08 | 7.37E-09 | 2.15E-08 | 8.79E-09 | 8.15E-09 |
| 6 | 1.48E-10 | 1.16E-09 | 2.43E-08 | 1.99E-08 | 1.50E-08 | 4.98E-09 | -3.17E-09 | -1.40E-08 | -2.44E-08 |
| 7 | -2.26E-12 | -1.79E-09 | -3.86E-08 | -2.89E-08 | 6.15E-09 | 4.69E-09 | -7.26E-09 | 9.83E-10 | -3.38E-09 |
| 8 | -1.08E-10 | -5.80E-10 | 9.15E-08 | 9.87E-08 | 7.98E-08 | -5.61E-09 | -2.00E-08 | -1.94E-08 | -4.54E-08 |
| 9 | 2.00E-10 | -1.47E-09 | -1.12E-08 | -6.45E-09 | -3.89E-09 | -3.26E-08 | -1.74E-08 | -1.45E-08 | -2.48E-08 |
| 10 | 8.55E-10 | 1.44E-10 | 1.91E-08 | -2.51E-08 | -7.70E-08 | -1.09E-07 | -2.12E-08 | 1.19E-07 | -1.62E-07 |
| 11 | 2.76E-09 | 2.58E-09 | -1.64E-08 | -4.94E-09 | 1.37E-09 | -2.07E-08 | 8.90E-09 | 1.38E-09 | 1.17E-08 |
| 12 | 6.17E-09 | 1.22E-08 | 1.56E-08 | 1.29E-08 | 1.15E-08 | 5.37E-09 | 7.43E-10 | 2.14E-08 | 2.45E-08 |
| 13 | 1.53E-08 | 2.66E-08 | 3.38E-08 | 6.90E-08 | 1.02E-07 | 2.64E-08 | 1.42E-08 | 5.05E-08 | -6.80E-08 |
| 14 | 3.78E-08 | 7.01E-08 | 8.06E-08 | 7.35E-08 | 1.01E-07 | 1.82E-07 | 1.18E-07 | -1.49E-08 | 4.70E-08 |
| 15 | 7.58E-08 | 1.68E-07 | 2.46E-07 | 2.87E-07 | 2.71E-07 | 3.56E-07 | 3.32E-07 | 3.58E-07 | 3.95E-07 |
| 16 | 8.40E-08 | 2.20E-07 | 3.54E-07 | 3.92E-07 | 4.18E-07 | 4.72E-07 | 6.06E-07 | 6.27E-07 | 3.73E-07 |
| 17 | 9.16E-08 | 2.85E-07 | 4.11E-07 | 5.20E-07 | 6.27E-07 | 7.31E-07 | 7.74E-07 | 8.78E-07 | 7.98E-07 |
| 18 | 8.10E-08 | 3.35E-07 | 5.15E-07 | 6.44E-07 | 7.56E-07 | 9.42E-07 | 1.04E-06 | 1.20E-06 | 1.08E-06 |
| 19 | 6.90E-08 | 3.76E-07 | 5.93E-07 | 7.65E-07 | 9.08E-07 | 1.13E-06 | 1.33E-06 | 1.50E-06 | 1.40E-06 |
| 20 | 3.70E-08 | 3.99E-07 | 6.79E-07 | 8.76E-07 | 1.05E-06 | 1.34E-06 | 1.54E-06 | 1.79E-06 | 1.70E-06 |
| 21 | 1.35E-10 | 4.09E-07 | 7.07E-07 | 9.42E-07 | 1.15E-06 | 1.48E-06 | 1.73E-06 | 2.09E-06 | 1.96E-06 |
| 22 | 1.80E-10 | 4.01E-07 | 7.25E-07 | 1.00E-06 | 1.24E-06 | 1.60E-06 | 1.89E-06 | 2.27E-06 | 2.20E-06 |
| 23 | -7.94E-11 | 3.73E-07 | 7.18E-07 | 1.00E-06 | 1.25E-06 | 1.67E-06 | 2.01E-06 | 2.43E-06 | 2.49E-06 |
| 24 | -3.67E-11 | 3.46E-07 | 7.01E-07 | 1.02E-06 | 1.29E-06 | 1.73E-06 | 2.10E-06 | 2.58E-06 | 2.58E-06 |
| 25 | 1.87E-10 | 3.08E-07 | 6.80E-07 | 1.02E-06 | 1.32E-06 | 1.79E-06 | 2.21E-06 | 2.75E-06 | 2.68E-06 |
| 26 | -1.16E-10 | 2.71E-07 | 6.54E-07 | 1.04E-06 | 1.36E-06 | 1.86E-06 | 2.30E-06 | 2.89E-06 | 2.96E-06 |
| 27 | -5.10E-11 | 2.27E-07 | 6.60E-07 | 1.04E-06 | 1.37E-06 | 1.91E-06 | 2.41E-06 | 3.05E-06 | 3.16E-06 |
| 28 | -8.53E-11 | 1.72E-07 | 6.40E-07 | 1.14E-06 | 1.47E-06 | 1.95E-06 | 2.53E-06 | 3.20E-06 | 3.35E-06 |
| 29 | 1.97E-10 | 1.17E-07 | 6.13E-07 | 1.03E-06 | 1.36E-06 | 2.03E-06 | 2.59E-06 | 3.32E-06 | 3.50E-06 |
| 30 | -3.28E-11 | 4.47E-08 | 5.51E-07 | 9.85E-07 | 1.42E-06 | 2.17E-06 | 2.73E-06 | 3.36E-06 | 3.63E-06 |
| 31 | -1.47E-10 | 2.17E-10 | 5.65E-07 | 9.55E-07 | 1.37E-06 | 2.15E-06 | 2.76E-06 | 3.39E-06 | 3.82E-06 |
| 32 | -1.87E-10 | 3.07E-11 | 4.99E-07 | 9.26E-07 | 1.29E-06 | 1.99E-06 | 2.63E-06 | 3.38E-06 | 3.74E-06 |
| 33 | 1.53E-10 | -1.24E-09 | 4.34E-07 | 8.80E-07 | 1.26E-06 | 1.96E-06 | 2.59E-06 | 3.34E-06 | 3.75E-06 |
| 34 | 7.03E-11 | 9.80E-10 | 3.72E-07 | 8.27E-07 | 1.19E-06 | 1.88E-06 | 2.54E-06 | 3.30E-06 | 3.73E-06 |
| 35 | 8.76E-11 | 1.41E-10 | 2.69E-07 | 7.78E-07 | 1.15E-06 | 1.81E-06 | 2.50E-06 | 3.26E-06 | 3.72E-06 |
| 36 | -1.55E-11 | -6.46E-10 | 2.47E-07 | 7.46E-07 | 1.11E-06 | 1.76E-06 | 2.43E-06 | 3.21E-06 | 3.68E-06 |
| 37 | 1.31E-10 | 1.93E-09 | 2.44E-07 | 6.39E-07 | 1.07E-06 | 1.72E-06 | 2.39E-06 | 3.20E-06 | 3.65E-06 |
| 38 | -1.37E-10 | 4.80E-10 | 1.50E-07 | 6.27E-07 | 1.00E-06 | 1.66E-06 | 2.32E-06 | 3.18E-06 | 3.62E-06 |
| 39 | -1.14E-10 | -1.97E-09 | 9.74E-08 | 5.80E-07 | 9.62E-07 | 1.61E-06 | 2.27E-06 | 3.11E-06 | 3.56E-06 |
| 40 | -1.47E-10 | 9.69E-10 | 3.29E-08 | 5.23E-07 | 9.07E-07 | 1.56E-06 | 2.21E-06 | 3.05E-06 | 3.53E-06 |
| 41 | -8.12E-11 | -1.93E-10 | 6.70E-09 | 4.77E-07 | 8.53E-07 | 1.51E-06 | 2.16E-06 | 2.98E-06 | 3.46E-06 |
| 42 | 1.70E-10 | 1.07E-09 | -2.03E-09 | 4.19E-07 | 8.16E-07 | 1.48E-06 | 2.09E-06 | 2.93E-06 | 3.40E-06 |
| 43 | 1.33E-10 | 1.46E-09 | 8.47E-09 | 3.74E-07 | 7.81E-07 | 1.43E-06 | 2.03E-06 | 2.85E-06 | 3.34E-06 |
| 44 | 7.32E-11 | 9.11E-10 | -5.49E-09 | 3.33E-07 | 7.08E-07 | 1.36E-06 | 1.96E-06 | 2.77E-06 | 3.26E-06 |
| 45 | -6.79E-11 | 1.59E-10 | -6.08E-09 | 2.74E-07 | 6.64E-07 | 1.31E-06 | 1.91E-06 | 2.71E-06 | 3.18E-06 |
| 46 | 8.06E-11 | 9.58E-10 | -4.70E-09 | 2.26E-07 | 6.16E-07 | 1.26E-06 | 1.85E-06 | 2.65E-06 | 3.12E-06 |
| 47 | -1.35E-10 | 7.11E-10 | 2.16E-09 | 1.78E-07 | 5.73E-07 | 1.20E-06 | 1.80E-06 | 2.58E-06 | 3.06E-06 |
| 48 | 7.73E-11 | 1.06E-10 | -6.62E-10 | 1.32E-07 | 5.29E-07 | 1.16E-06 | 1.77E-06 | 2.52E-06 | 3.00E-06 |
| 49 | 7.02E-11 | 8.38E-10 | -3.66E-08 | 1.36E-07 | 4.59E-07 | 1.11E-06 | 1.68E-06 | 2.46E-06 | 2.94E-06 |
| 50 | 8.45E-11 | -5.29E-10 | 9.89E-10 | 3.57E-08 | 4.44E-07 | 1.06E-06 | 1.63E-06 | 2.38E-06 | 2.87E-06 |
| 51 | -1.90E-10 | 4.59E-10 | -1.20E-08 | 2.58E-08 | 3.84E-07 | 1.02E-06 | 1.53E-06 | 2.33E-06 | 2.84E-06 |
| 52 | -1.55E-10 | -3.09E-10 | -8.04E-09 | 4.48E-08 | 2.22E-07 | 9.57E-07 | 1.50E-06 | 2.26E-06 | 2.75E-06 |
| 53 | 1.21E-10 | 1.40E-09 | -2.72E-08 | 2.55E-08 | 2.99E-07 | 9.04E-07 | 1.46E-06 | 2.21E-06 | 2.68E-06 |
| 54 | 1.36E-10 | -1.21E-09 | -2.18E-08 | 3.48E-08 | 2.51E-07 | 8.75E-07 | 1.43E-06 | 2.16E-06 | 2.63E-06 |
| 55 | 1.28E-10 | 1.80E-09 | 1.41E-09 | 8.08E-09 | 2.21E-07 | 8.23E-07 | 1.39E-06 | 2.11E-06 | 2.57E-06 |
| 56 | 7.13E-11 | -4.56E-10 | -5.68E-08 | -1.49E-08 | 1.82E-07 | 7.46E-07 | 1.30E-06 | 2.06E-06 | 2.50E-06 |
| 57 | 1.43E-10 | -1.32E-09 | -7.49E-09 | 2.00E-09 | 1.40E-07 | 7.44E-07 | 1.29E-06 | 1.99E-06 | 2.46E-06 |
| 58 | 1.20E-10 | -1.69E-09 | -1.57E-09 | -2.93E-09 | 1.17E-07 | 7.06E-07 | 1.23E-06 | 1.89E-06 | 2.41E-06 |
| 59 | 9.18E-11 | -1.62E-09 | 2.58E-09 | -1.34E-09 | 6.56E-08 | 8.15E-07 | 2.15E-06 | 4.64E-06 | 6.28E-06 |
| 60 | 9.63E-11 | -5.43E-10 | 1.67E-08 | -1.29E-08 | 3.43E-08 | 8.86E-07 | 2.85E-06 | 6.83E-06 | 9.48E-06 |
| 61 | -1.77E-10 | 2.09E-10 | 1.17E-08 | -1.52E-08 | 2.86E-08 | 5.84E-07 | 1.05E-06 | 1.79E-06 | 2.29E-06 |
| 62 | -1.56E-10 | -1.97E-09 | 5.48E-11 | 2.36E-10 | -1.03E-09 | 5.72E-07 | 1.14E-06 | 1.86E-06 | 2.40E-06 |
| 63 | 1.88E-10 | 6.02E-10 | 2.39E-09 | -9.43E-10 | 1.41E-09 | 5.70E-07 | 9.99E-07 | 1.56E-06 | 2.01E-06 |
| 64 | -1.20E-10 | -9.46E-10 | 6.24E-10 | -9.22E-11 | 4.26E-10 | 4.96E-07 | 9.82E-07 | 1.49E-06 | 1.96E-06 |
| 65 | -1.36E-10 | 1.86E-10 | 1.36E-10 | -2.98E-10 | -1.08E-09 | 4.60E-07 | 9.39E-07 | 1.49E-06 | 1.94E-06 |
| 66 | -8.22E-11 | -1.23E-09 | -1.78E-08 | -3.63E-08 | 2.34E-08 | 3.96E-07 | 8.71E-07 | 1.46E-06 | 1.93E-06 |
| 67 | 1.77E-10 | -1.03E-09 | 9.13E-10 | 9.79E-10 | -6.27E-10 | 4.12E-07 | 8.15E-07 | 1.42E-06 | 1.88E-06 |
| 68 | -1.98E-10 | -1.23E-09 | -1.72E-10 | -6.11E-10 | 9.12E-10 | 4.69E-07 | 1.46E-06 | 3.13E-06 | 4.44E-06 |
| 69 | 2.09E-11 | 5.46E-10 | -2.20E-10 | -4.98E-09 | -9.42E-10 | 3.25E-07 | 7.76E-07 | 1.39E-06 | 1.70E-06 |
| 70 | 1.33E-10 | -1.85E-09 | 5.37E-09 | -3.34E-09 | 6.43E-09 | 3.11E-07 | 9.16E-07 | 1.78E-06 | 2.39E-06 |
| 71 | 1.82E-11 | 1.19E-09 | 6.45E-10 | 2.24E-10 | 4.84E-10 | 2.68E-07 | 7.21E-07 | 1.20E-06 | 1.42E-06 |
| 72 | -2.99E-11 | 1.70E-09 | 8.78E-12 | -6.83E-10 | 7.88E-11 | 2.40E-07 | 6.41E-07 | 1.18E-06 | 1.42E-06 |
| 73 | 1.21E-10 | -2.34E-10 | 3.98E-09 | -3.17E-09 | 4.18E-09 | 2.07E-07 | 6.13E-07 | 1.13E-06 | 1.39E-06 |
| 74 | 8.64E-11 | 1.12E-09 | 1.48E-09 | -2.24E-09 | 1.21E-09 | 1.80E-07 | 5.83E-07 | 1.10E-06 | 1.36E-06 |
| 75 | -1.37E-10 | -1.38E-09 | -5.79E-11 | -2.57E-09 | -7.98E-10 | 1.51E-07 | 5.56E-07 | 1.07E-06 | 1.32E-06 |
| 76 | 1.89E-10 | 1.48E-09 | 6.90E-10 | 1.76E-10 | 6.70E-10 | 1.23E-07 | 5.33E-07 | 1.04E-06 | 1.29E-06 |
| 77 | -1.43E-10 | 1.26E-10 | 5.45E-10 | 4.30E-11 | 4.71E-10 | 9.62E-08 | 5.13E-07 | 1.01E-06 | 1.26E-06 |
| 78 | 1.01E-10 | -1.71E-09 | 3.90E-12 | 3.06E-10 | 1.95E-09 | 7.05E-08 | 4.97E-07 | 9.82E-07 | 1.24E-06 |
| 79 | -1.73E-10 | -1.56E-09 | 8.58E-12 | 4.93E-10 | 1.18E-09 | 4.16E-08 | 4.74E-07 | 9.48E-07 | 1.21E-06 |
| 80 | 1.76E-10 | 5.22E-11 | -1.06E-09 | 5.88E-10 | -2.00E-09 | 1.47E-08 | 4.50E-07 | 9.15E-07 | 1.19E-06 |
| 81 | -5.04E-11 | -1.67E-09 | 1.75E-10 | 3.32E-10 | 2.45E-10 | -2.15E-10 | 4.27E-07 | 8.80E-07 | 1.16E-06 |
| 82 | 1.40E-10 | -8.59E-10 | 3.86E-10 | 3.84E-11 | -7.11E-10 | -6.12E-10 | 4.03E-07 | 8.51E-07 | 1.14E-06 |
| 83 | -1.03E-10 | -1.62E-09 | 1.31E-10 | 1.00E-10 | -2.39E-10 | -3.47E-10 | 3.78E-07 | 8.21E-07 | 1.11E-06 |
| 84 | -1.29E-10 | -1.95E-09 | -1.45E-08 | -3.27E-09 | 1.94E-08 | 1.21E-07 | 3.42E-07 | 8.42E-07 | 1.09E-06 |
| 85 | 1.88E-10 | 6.83E-10 | -8.25E-11 | 4.07E-10 | 6.94E-10 | -5.98E-10 | 3.31E-07 | 7.68E-07 | 1.06E-06 |
| 86 | -8.60E-11 | -1.10E-09 | -1.36E-09 | -1.38E-08 | -1.51E-08 | 6.56E-08 | 2.89E-07 | 7.20E-07 | 1.01E-06 |
| 87 | 7.55E-11 | 1.74E-09 | 2.27E-10 | 3.46E-10 | 1.42E-10 | 1.25E-10 | 2.84E-07 | 7.30E-07 | 1.01E-06 |
| 88 | -4.74E-11 | -8.38E-10 | -1.90E-09 | 8.96E-10 | -1.16E-09 | -4.49E-09 | 2.54E-07 | 7.08E-07 | 9.87E-07 |
| 89 | -1.16E-10 | 1.58E-10 | -1.60E-12 | -1.75E-11 | -3.06E-11 | -1.53E-10 | 2.36E-07 | 6.88E-07 | 9.64E-07 |
| 90 | 2.89E-11 | -9.35E-10 | -7.83E-11 | 8.43E-11 | 6.52E-11 | -1.36E-10 | 2.12E-07 | 6.69E-07 | 9.42E-07 |
| 91 | 1.03E-10 | -8.24E-10 | 4.65E-10 | -1.01E-09 | -2.58E-09 | 1.05E-09 | 1.89E-07 | 6.47E-07 | 9.21E-07 |
| 92 | -4.47E-11 | 1.76E-09 | 1.45E-11 | 3.68E-11 | 7.55E-12 | -6.39E-11 | 1.70E-07 | 6.28E-07 | 8.97E-07 |
| 93 | 2.20E-11 | -1.50E-09 | -5.42E-11 | -5.61E-12 | 1.25E-10 | 2.57E-11 | 1.52E-07 | 6.07E-07 | 8.72E-07 |
| 94 | 7.44E-11 | -6.40E-10 | 9.08E-11 | 2.75E-10 | -3.55E-10 | -4.03E-10 | 1.33E-07 | 5.87E-07 | 8.47E-07 |
| 95 | 8.37E-11 | -2.66E-10 | -8.68E-11 | 6.82E-11 | 1.39E-10 | 1.70E-10 | 1.13E-07 | 5.65E-07 | 8.19E-07 |
| 96 | -7.27E-11 | 8.59E-10 | 1.48E-09 | -1.52E-09 | -6.30E-09 | -7.18E-09 | 8.32E-08 | 5.46E-07 | 8.07E-07 |
| 97 | -1.15E-11 | -1.08E-09 | 3.60E-10 | 4.26E-10 | -1.15E-10 | 1.06E-09 | 7.37E-08 | 5.22E-07 | 7.68E-07 |
| 98 | 1.93E-10 | 1.28E-09 | 5.37E-11 | 9.15E-11 | -9.75E-11 | 2.12E-10 | 5.07E-08 | 4.96E-07 | 7.43E-07 |
| 99 | -1.17E-10 | -2.20E-10 | -1.16E-10 | -1.60E-10 | 3.10E-11 | 1.46E-10 | 2.98E-08 | 4.75E-07 | 7.23E-07 |
| 100 | -6.70E-11 | 6.82E-10 | 3.80E-11 | 7.18E-11 | 1.95E-11 | -3.05E-11 | 1.18E-08 | 4.58E-07 | 7.07E-07 |
| 101 | -5.01E-11 | -1.73E-10 | -1.88E-10 | -2.41E-10 | -1.45E-10 | 2.73E-10 | -1.67E-10 | 4.46E-07 | 6.89E-07 |
| 102 | -1.14E-10 | -6.35E-10 | -3.22E-12 | -5.71E-12 | 4.10E-12 | -1.37E-11 | -2.67E-11 | 4.30E-07 | 6.73E-07 |
| 103 | -9.90E-12 | -1.28E-09 | 2.34E-11 | 4.26E-11 | 5.50E-11 | -9.44E-12 | -8.82E-11 | 4.13E-07 | 6.56E-07 |
| 104 | 3.08E-11 | -3.69E-10 | -2.24E-11 | -1.95E-11 | -3.77E-12 | 2.11E-11 | -1.16E-11 | 3.96E-07 | 6.38E-07 |
| 105 | -9.63E-11 | -4.79E-11 | -3.39E-11 | -6.47E-11 | -6.03E-11 | -1.28E-10 | -8.18E-11 | 3.76E-07 | 6.18E-07 |
| 106 | -3.40E-11 | 9.71E-10 | -8.30E-11 | -5.96E-11 | -1.73E-11 | -3.97E-11 | -1.25E-10 | 3.56E-07 | 5.99E-07 |
| 107 | 1.17E-11 | -1.53E-09 | -1.97E-11 | 1.81E-11 | 6.76E-11 | 8.92E-11 | 3.69E-12 | 3.36E-07 | 5.82E-07 |
| 108 | -1.22E-12 | 8.89E-10 | 2.94E-12 | 1.04E-11 | 1.84E-11 | 3.56E-12 | -1.13E-11 | 3.23E-07 | 5.66E-07 |
| 109 | 1.55E-11 | 1.29E-09 | 3.77E-11 | 1.09E-10 | 9.31E-11 | -2.74E-10 | -2.74E-10 | 3.08E-07 | 5.51E-07 |
| 110 | -8.16E-11 | 1.09E-09 | -9.28E-12 | -5.98E-12 | -3.67E-12 | -6.19E-11 | -4.74E-11 | 2.96E-07 | 5.39E-07 |
| 111 | 1.75E-10 | 1.66E-09 | 1.90E-11 | 3.83E-11 | 4.50E-11 | -1.44E-11 | 8.62E-11 | 2.83E-07 | 5.24E-07 |
| 112 | -6.24E-11 | -1.26E-12 | 1.36E-11 | 6.52E-12 | -1.55E-11 | -9.09E-11 | 1.50E-10 | 2.68E-07 | 5.10E-07 |
| 113 | 1.44E-10 | -1.61E-09 | 6.04E-10 | 4.20E-10 | 1.37E-10 | -5.51E-10 | 5.62E-10 | 2.48E-07 | 4.94E-07 |
| 114 | 1.30E-10 | -1.09E-09 | -5.04E-11 | -6.16E-11 | -9.74E-11 | -3.80E-10 | -7.71E-10 | 2.27E-07 | 4.77E-07 |
| 115 | 2.62E-11 | -3.36E-10 | 7.37E-13 | 2.32E-11 | 4.28E-12 | -6.16E-11 | 8.25E-11 | 2.07E-07 | 4.54E-07 |
| 116 | 1.67E-10 | -4.15E-10 | 1.71E-12 | -1.04E-11 | -1.59E-11 | -4.08E-11 | 1.85E-11 | 1.88E-07 | 4.34E-07 |
| 117 | 1.28E-10 | -8.07E-11 | -1.05E-10 | -4.41E-12 | 1.90E-10 | -5.36E-10 | 1.53E-10 | 1.71E-07 | 4.13E-07 |
| 118 | -7.53E-11 | 3.26E-10 | -2.80E-11 | -3.24E-11 | -1.57E-11 | 3.59E-11 | -7.77E-11 | 1.57E-07 | 3.96E-07 |
| 119 | 1.13E-10 | 8.94E-12 | 8.42E-11 | 8.82E-11 | 3.87E-11 | -2.04E-10 | 7.76E-11 | 1.45E-07 | 3.80E-07 |
| 120 | -1.42E-10 | -1.91E-09 | 1.56E-12 | -4.91E-11 | -7.43E-11 | -8.46E-12 | 1.26E-11 | 1.34E-07 | 3.65E-07 |
| 121 | 1.42E-10 | 1.27E-09 | -9.73E-11 | -8.11E-11 | 6.80E-11 | -5.81E-11 | -3.32E-10 | 1.22E-07 | 3.52E-07 |
| 122 | -1.51E-12 | -1.63E-09 | -3.14E-11 | -1.09E-10 | 2.48E-11 | -6.22E-13 | -1.74E-10 | 1.10E-07 | 3.39E-07 |
| 123 | 1.74E-10 | -1.98E-09 | 4.42E-11 | -3.22E-11 | 4.45E-11 | 2.99E-11 | -4.32E-11 | 9.60E-08 | 3.26E-07 |
| 124 | 1.25E-10 | -1.68E-10 | 5.12E-12 | -8.42E-12 | -9.25E-12 | 1.94E-11 | 1.19E-09 | 8.20E-08 | 3.14E-07 |
| 125 | 1.56E-11 | 1.25E-09 | 4.18E-11 | 5.02E-11 | 4.29E-11 | -7.18E-11 | 1.17E-10 | 6.91E-08 | 3.02E-07 |
| 126 | 1.54E-10 | -4.39E-10 | -2.50E-11 | -2.97E-11 | -2.22E-11 | -8.59E-11 | 1.49E-11 | 5.48E-08 | 2.89E-07 |
| 127 | -4.82E-13 | -3.50E-10 | -1.99E-12 | -2.48E-11 | -3.91E-11 | -5.87E-11 | 1.43E-11 | 4.23E-08 | 2.75E-07 |
| 128 | 2.63E-11 | 1.40E-10 | -9.17E-14 | 1.06E-11 | 2.17E-11 | -3.77E-11 | 1.10E-11 | 3.01E-08 | 2.59E-07 |
| 129 | 3.86E-11 | 1.38E-09 | 4.49E-12 | 9.03E-12 | 1.70E-11 | 2.54E-11 | 4.91E-12 | 1.77E-08 | 2.44E-07 |
| 130 | -7.04E-11 | 5.60E-10 | 2.16E-12 | 1.63E-11 | 2.59E-11 | 1.21E-11 | 2.44E-11 | 4.50E-09 | 2.29E-07 |
| 131 | 4.44E-11 | -1.98E-09 | -4.25E-10 | 4.28E-10 | 7.43E-10 | -1.58E-10 | -1.56E-09 | 2.46E-09 | 2.13E-07 |
| 132 | 1.78E-10 | -5.11E-10 | 7.04E-12 | -2.93E-12 | -1.62E-11 | -5.11E-11 | -9.35E-11 | 1.56E-10 | 2.01E-07 |
| 133 | -4.52E-11 | 5.68E-10 | 1.41E-12 | 2.43E-12 | 1.13E-12 | -4.99E-12 | -3.41E-11 | -4.83E-11 | 1.89E-07 |
| 134 | -3.31E-11 | -1.20E-09 | 7.15E-13 | -3.50E-13 | -2.39E-12 | -2.40E-12 | 6.74E-13 | 2.61E-13 | 1.76E-07 |
| 135 | -1.79E-10 | 1.57E-09 | -1.31E-11 | -1.18E-11 | -3.17E-12 | 5.82E-11 | 1.03E-10 | 1.58E-10 | 1.65E-07 |
| 136 | 1.16E-10 | 4.21E-10 | -8.92E-13 | 8.31E-12 | 2.22E-11 | 4.01E-11 | -4.39E-11 | 1.17E-10 | 1.53E-07 |
| 137 | -2.73E-11 | -1.90E-09 | 4.10E-11 | 3.79E-11 | 3.20E-11 | 1.72E-11 | -3.05E-11 | 2.37E-11 | 1.42E-07 |
| 138 | 7.61E-11 | -5.08E-11 | -2.29E-12 | -3.51E-12 | -5.36E-12 | -1.03E-11 | -2.57E-11 | -5.76E-11 | 1.31E-07 |
| 139 | 6.05E-11 | -3.39E-10 | -6.36E-13 | 3.14E-12 | 6.88E-12 | 1.32E-11 | 1.52E-11 | 2.02E-11 | 1.19E-07 |
| 140 | 8.76E-11 | 1.20E-09 | 4.92E-12 | 2.74E-12 | 6.98E-13 | -1.88E-12 | -7.00E-12 | 2.57E-11 | 1.07E-07 |
| 141 | -4.82E-11 | -1.96E-09 | -2.43E-14 | -1.56E-12 | -1.99E-12 | 3.05E-12 | 1.06E-11 | 5.74E-12 | 9.61E-08 |
| 142 | 4.28E-11 | -1.68E-09 | -8.64E-13 | -1.40E-12 | -9.43E-13 | 3.68E-12 | -1.94E-12 | -8.24E-11 | 8.58E-08 |
| 143 | 5.81E-11 | 1.74E-10 | 5.46E-13 | 1.35E-12 | 1.81E-12 | 8.63E-13 | -1.10E-11 | 9.36E-11 | 7.60E-08 |
| 144 | -7.21E-11 | -1.44E-09 | -1.44E-12 | -1.25E-13 | 3.69E-12 | 1.08E-11 | -1.44E-11 | -1.21E-11 | 6.65E-08 |
| 145 | 2.00E-10 | -1.43E-09 | -3.40E-12 | -1.27E-12 | 1.78E-12 | 6.16E-12 | 3.62E-13 | 1.24E-11 | 5.69E-08 |
| 146 | 3.09E-11 | -1.44E-09 | -2.39E-12 | -8.97E-13 | 6.53E-13 | 3.86E-12 | 7.86E-13 | -3.03E-12 | 4.73E-08 |
| 147 | 9.56E-11 | -5.25E-10 | -3.95E-13 | 3.80E-12 | 8.33E-12 | 1.20E-11 | -6.94E-11 | -3.27E-11 | 3.72E-08 |
| 148 | -1.86E-10 | -1.23E-10 | -2.39E-13 | -1.95E-13 | -7.70E-14 | 5.46E-13 | 6.27E-14 | 5.26E-13 | 2.64E-08 |
| 149 | -1.17E-10 | -1.37E-09 | 3.36E-12 | 1.37E-12 | -1.37E-12 | -8.61E-12 | -1.60E-11 | 1.59E-13 | 1.55E-08 |
| 150 | 1.28E-10 | -3.25E-10 | -3.76E-14 | -7.30E-14 | -1.82E-13 | -3.22E-13 | 5.54E-13 | 4.51E-13 | 3.68E-09 |

Table.17. Spectra predicted by MLP neural network: Be- 2mm

| energy bin (kev)/voltage(kv) | 20kv | 30kv | 40kv | 50kv | 60kv | 80kv | 100kv | 130kv | 150kv |
| --- | --- | --- | --- | --- | --- | --- | --- | --- | --- |
| 1 | 0 | 0 | 0 | 0 | 0 | 0 | 0 | 0 | 0 |
| 2 | -5.56E-11 | 1.28E-09 | -1.27E-09 | -3.02E-10 | 2.28E-14 | -9.06E-14 | -1.46E-13 | 1.42E-14 | 5.33E-13 |
| 3 | -7.06E-11 | 4.84E-10 | 5.20E-10 | 1.42E-09 | -6.15E-11 | -6.91E-11 | 4.38E-11 | -3.42E-11 | -7.92E-11 |
| 4 | -1.87E-10 | 8.28E-10 | 1.39E-09 | -1.74E-09 | -8.49E-09 | 1.15E-09 | 1.52E-08 | 1.88E-08 | -4.53E-09 |
| 5 | -8.23E-11 | 3.75E-10 | 3.73E-10 | 1.45E-09 | 1.74E-08 | 1.19E-09 | 3.34E-08 | 3.33E-08 | 8.87E-09 |
| 6 | 1.89E-10 | -3.80E-10 | 1.26E-09 | 1.25E-09 | 6.92E-09 | -6.14E-09 | -2.07E-08 | -5.27E-08 | -8.15E-08 |
| 7 | -1.38E-10 | 1.26E-09 | -5.49E-10 | 4.89E-10 | 8.81E-08 | -5.67E-08 | -4.96E-08 | 5.48E-08 | 3.49E-09 |
| 8 | -4.42E-12 | 1.21E-09 | 1.38E-09 | 1.43E-09 | 1.72E-07 | 5.66E-08 | -1.11E-08 | -2.88E-08 | -6.35E-08 |
| 9 | 5.42E-11 | -2.04E-10 | -2.61E-10 | 5.10E-10 | 1.26E-08 | 1.63E-08 | -4.75E-09 | 7.19E-09 | 1.11E-08 |
| 10 | 1.72E-10 | -9.65E-10 | 1.33E-09 | -7.95E-10 | -8.49E-09 | 5.55E-08 | 9.65E-08 | 6.26E-08 | -2.76E-07 |
| 11 | 4.65E-10 | 8.19E-10 | 1.72E-09 | 1.95E-09 | 6.60E-09 | -5.18E-10 | 4.62E-09 | 4.84E-08 | 5.02E-09 |
| 12 | 1.51E-09 | 3.64E-09 | 4.33E-09 | 2.04E-09 | -4.39E-08 | -3.58E-08 | -1.89E-08 | 8.28E-09 | 4.27E-08 |
| 13 | 4.72E-09 | 7.91E-09 | 1.07E-08 | 1.29E-08 | 2.98E-08 | 2.41E-08 | 4.47E-08 | 6.21E-08 | 1.41E-08 |
| 14 | 1.60E-08 | 3.04E-08 | 3.97E-08 | 4.58E-08 | 6.85E-08 | 7.65E-08 | 1.98E-08 | 5.08E-08 | 1.06E-07 |
| 15 | 4.16E-08 | 9.15E-08 | 1.29E-07 | 1.55E-07 | 1.61E-07 | 1.99E-07 | 1.96E-07 | 1.44E-07 | 2.08E-07 |
| 16 | 5.04E-08 | 1.36E-07 | 1.94E-07 | 2.44E-07 | 2.73E-07 | 3.57E-07 | 4.10E-07 | 3.66E-07 | 2.70E-07 |
| 17 | 5.79E-08 | 1.87E-07 | 2.74E-07 | 3.50E-07 | 4.15E-07 | 4.93E-07 | 5.05E-07 | 5.70E-07 | 5.45E-07 |
| 18 | 5.38E-08 | 2.36E-07 | 3.58E-07 | 4.66E-07 | 5.47E-07 | 6.74E-07 | 7.18E-07 | 8.16E-07 | 7.78E-07 |
| 19 | 4.92E-08 | 2.73E-07 | 4.35E-07 | 5.77E-07 | 6.83E-07 | 8.47E-07 | 1.03E-06 | 1.10E-06 | 1.15E-06 |
| 20 | 2.90E-08 | 3.04E-07 | 4.99E-07 | 6.73E-07 | 7.96E-07 | 1.03E-06 | 1.18E-06 | 1.36E-06 | 1.29E-06 |
| 21 | -1.70E-10 | 3.22E-07 | 5.47E-07 | 7.53E-07 | 9.22E-07 | 1.17E-06 | 1.36E-06 | 1.67E-06 | 1.53E-06 |
| 22 | 1.49E-10 | 3.22E-07 | 5.75E-07 | 8.06E-07 | 9.87E-07 | 1.29E-06 | 1.52E-06 | 1.80E-06 | 1.77E-06 |
| 23 | 1.11E-10 | 3.04E-07 | 5.79E-07 | 8.23E-07 | 1.04E-06 | 1.43E-06 | 1.76E-06 | 1.84E-06 | 1.89E-06 |
| 24 | -5.72E-11 | 2.85E-07 | 5.81E-07 | 8.44E-07 | 1.07E-06 | 1.45E-06 | 1.76E-06 | 2.15E-06 | 2.15E-06 |
| 25 | 1.37E-10 | 2.63E-07 | 5.81E-07 | 8.63E-07 | 1.12E-06 | 1.52E-06 | 1.87E-06 | 2.34E-06 | 2.20E-06 |
| 26 | 5.89E-11 | 2.32E-07 | 5.77E-07 | 8.81E-07 | 1.17E-06 | 1.59E-06 | 1.98E-06 | 2.50E-06 | 2.55E-06 |
| 27 | -7.52E-11 | 1.98E-07 | 5.79E-07 | 8.97E-07 | 1.21E-06 | 1.68E-06 | 2.07E-06 | 2.63E-06 | 2.76E-06 |
| 28 | 2.86E-11 | 1.54E-07 | 5.74E-07 | 9.15E-07 | 1.22E-06 | 1.76E-06 | 2.24E-06 | 2.85E-06 | 2.96E-06 |
| 29 | 1.16E-10 | 1.05E-07 | 5.58E-07 | 9.20E-07 | 1.23E-06 | 1.84E-06 | 2.35E-06 | 3.00E-06 | 3.14E-06 |
| 30 | -1.87E-10 | 4.26E-08 | 5.32E-07 | 9.10E-07 | 1.24E-06 | 1.88E-06 | 2.38E-06 | 3.08E-06 | 3.33E-06 |
| 31 | -1.01E-10 | -1.38E-09 | 5.00E-07 | 8.90E-07 | 1.33E-06 | 2.04E-06 | 2.62E-06 | 3.22E-06 | 3.55E-06 |
| 32 | -1.49E-10 | -7.98E-10 | 4.57E-07 | 8.58E-07 | 1.20E-06 | 1.86E-06 | 2.44E-06 | 3.10E-06 | 3.47E-06 |
| 33 | -5.13E-11 | 1.09E-09 | 4.05E-07 | 8.14E-07 | 1.17E-06 | 1.82E-06 | 2.41E-06 | 3.15E-06 | 3.56E-06 |
| 34 | 1.15E-10 | -4.20E-10 | 3.59E-07 | 7.71E-07 | 1.12E-06 | 1.76E-06 | 2.38E-06 | 3.10E-06 | 3.49E-06 |
| 35 | 6.34E-11 | -1.51E-09 | 3.05E-07 | 7.31E-07 | 1.09E-06 | 1.70E-06 | 2.35E-06 | 3.06E-06 | 3.50E-06 |
| 36 | 8.55E-11 | -1.22E-09 | 2.55E-07 | 6.86E-07 | 1.05E-06 | 1.65E-06 | 2.30E-06 | 3.04E-06 | 3.48E-06 |
| 37 | -4.68E-11 | 7.12E-10 | 2.00E-07 | 6.39E-07 | 9.75E-07 | 1.60E-06 | 2.25E-06 | 3.02E-06 | 3.46E-06 |
| 38 | 6.01E-11 | -1.70E-09 | 1.49E-07 | 5.98E-07 | 9.64E-07 | 1.59E-06 | 2.22E-06 | 3.05E-06 | 3.45E-06 |
| 39 | 8.47E-11 | 1.92E-09 | 9.35E-08 | 5.52E-07 | 9.14E-07 | 1.54E-06 | 2.17E-06 | 2.97E-06 | 3.41E-06 |
| 40 | 1.81E-10 | -1.68E-10 | 3.99E-08 | 5.04E-07 | 8.77E-07 | 1.50E-06 | 2.11E-06 | 2.93E-06 | 3.37E-06 |
| 41 | -4.97E-11 | -1.66E-09 | -1.27E-09 | 4.57E-07 | 8.07E-07 | 1.44E-06 | 2.06E-06 | 2.87E-06 | 3.34E-06 |
| 42 | 1.22E-10 | 1.55E-09 | -3.44E-10 | 4.09E-07 | 7.78E-07 | 1.42E-06 | 2.00E-06 | 2.81E-06 | 3.29E-06 |
| 43 | -1.97E-10 | 1.54E-09 | 8.11E-10 | 3.58E-07 | 7.43E-07 | 1.37E-06 | 1.95E-06 | 2.74E-06 | 3.21E-06 |
| 44 | 1.45E-10 | -3.94E-10 | -2.03E-10 | 3.13E-07 | 6.62E-07 | 1.32E-06 | 1.90E-06 | 2.68E-06 | 3.15E-06 |
| 45 | -1.49E-10 | 1.28E-09 | -3.43E-10 | 2.67E-07 | 6.47E-07 | 1.26E-06 | 1.84E-06 | 2.63E-06 | 3.09E-06 |
| 46 | -1.73E-10 | 1.65E-09 | -1.01E-09 | 2.21E-07 | 6.01E-07 | 1.22E-06 | 1.80E-06 | 2.56E-06 | 3.02E-06 |
| 47 | 1.16E-10 | 1.26E-09 | 2.76E-10 | 1.73E-07 | 5.56E-07 | 1.16E-06 | 1.75E-06 | 2.50E-06 | 2.96E-06 |
| 48 | 1.85E-10 | 1.99E-09 | 9.00E-10 | 1.29E-07 | 5.15E-07 | 1.12E-06 | 1.73E-06 | 2.46E-06 | 2.91E-06 |
| 49 | 1.43E-10 | -1.17E-09 | -1.99E-10 | 8.26E-08 | 4.65E-07 | 1.08E-06 | 1.64E-06 | 2.38E-06 | 2.85E-06 |
| 50 | 1.88E-10 | -1.56E-09 | -6.48E-10 | 3.45E-08 | 4.30E-07 | 1.03E-06 | 1.58E-06 | 2.31E-06 | 2.79E-06 |
| 51 | 1.99E-10 | -6.01E-10 | 1.60E-09 | 1.50E-09 | 3.64E-07 | 1.01E-06 | 1.52E-06 | 2.33E-06 | 2.84E-06 |
| 52 | 7.27E-11 | 8.46E-10 | 8.83E-10 | 3.99E-10 | 1.72E-07 | 9.33E-07 | 1.48E-06 | 2.20E-06 | 2.68E-06 |
| 53 | -1.97E-10 | -7.75E-10 | 6.71E-10 | -1.23E-09 | 2.99E-07 | 8.98E-07 | 1.41E-06 | 2.12E-06 | 2.61E-06 |
| 54 | 1.43E-10 | 1.59E-09 | -1.88E-09 | 1.84E-09 | 2.43E-07 | 8.48E-07 | 1.38E-06 | 2.11E-06 | 2.57E-06 |
| 55 | -9.21E-11 | 8.89E-10 | -1.32E-09 | 1.69E-09 | 2.19E-07 | 8.09E-07 | 1.36E-06 | 2.07E-06 | 2.51E-06 |
| 56 | -1.19E-10 | 6.07E-10 | 9.92E-10 | 8.06E-10 | 1.96E-07 | 7.63E-07 | 1.30E-06 | 2.00E-06 | 2.40E-06 |
| 57 | -3.65E-11 | 1.55E-10 | 6.07E-10 | 7.28E-10 | 1.38E-07 | 7.31E-07 | 1.26E-06 | 1.94E-06 | 2.41E-06 |
| 58 | 1.02E-10 | 7.22E-10 | 1.96E-09 | 1.69E-09 | 1.08E-07 | 6.91E-07 | 1.21E-06 | 1.85E-06 | 2.36E-06 |
| 59 | 6.92E-12 | 5.31E-10 | 4.33E-10 | -8.58E-10 | 6.56E-08 | 7.98E-07 | 2.10E-06 | 4.54E-06 | 6.15E-06 |
| 60 | 1.75E-10 | -2.45E-10 | 3.72E-10 | 1.64E-09 | 2.98E-08 | 8.61E-07 | 2.80E-06 | 6.70E-06 | 9.29E-06 |
| 61 | -1.73E-10 | 6.82E-10 | -6.15E-10 | -4.40E-10 | 3.53E-08 | 5.82E-07 | 1.02E-06 | 1.77E-06 | 2.26E-06 |
| 62 | -1.57E-10 | -9.75E-10 | -6.31E-10 | 1.32E-09 | -2.12E-09 | 5.61E-07 | 1.12E-06 | 1.83E-06 | 2.42E-06 |
| 63 | -1.16E-10 | -6.73E-11 | 1.43E-09 | -1.64E-09 | -2.19E-09 | 5.55E-07 | 9.76E-07 | 1.53E-06 | 1.98E-06 |
| 64 | 7.59E-11 | 5.22E-10 | 1.64E-09 | 4.80E-10 | -1.47E-09 | 4.86E-07 | 9.61E-07 | 1.47E-06 | 1.94E-06 |
| 65 | -5.41E-11 | 1.08E-09 | -7.35E-10 | -3.92E-11 | -3.86E-10 | 4.53E-07 | 9.27E-07 | 1.47E-06 | 1.91E-06 |
| 66 | -6.94E-11 | -1.63E-09 | -1.96E-09 | 1.12E-09 | 2.63E-08 | 4.01E-07 | 8.73E-07 | 1.45E-06 | 1.90E-06 |
| 67 | -1.15E-10 | -8.38E-10 | -5.13E-10 | -1.49E-09 | -2.85E-09 | 4.04E-07 | 8.01E-07 | 1.39E-06 | 1.84E-06 |
| 68 | 6.80E-11 | 9.31E-10 | -1.88E-09 | 7.99E-10 | -4.08E-09 | 4.55E-07 | 1.43E-06 | 3.08E-06 | 4.36E-06 |
| 69 | 1.50E-10 | -6.62E-10 | 3.12E-10 | -7.02E-10 | 1.82E-09 | 3.22E-07 | 7.66E-07 | 1.35E-06 | 1.67E-06 |
| 70 | -1.20E-10 | -3.45E-11 | 1.89E-09 | 1.97E-09 | 3.31E-09 | 2.99E-07 | 8.94E-07 | 1.75E-06 | 2.37E-06 |
| 71 | 2.57E-11 | -9.38E-10 | 3.64E-10 | 3.16E-10 | -2.69E-10 | 2.65E-07 | 7.11E-07 | 1.18E-06 | 1.40E-06 |
| 72 | -1.56E-10 | 1.67E-09 | -8.63E-10 | 5.70E-10 | -3.98E-10 | 2.35E-07 | 6.30E-07 | 1.16E-06 | 1.40E-06 |
| 73 | -5.04E-11 | -1.36E-09 | -1.62E-09 | 1.82E-09 | 1.72E-09 | 2.01E-07 | 6.03E-07 | 1.11E-06 | 1.37E-06 |
| 74 | 4.99E-11 | 1.05E-09 | -8.29E-10 | 1.56E-09 | 1.88E-09 | 1.76E-07 | 5.73E-07 | 1.08E-06 | 1.34E-06 |
| 75 | -1.05E-10 | -7.85E-10 | -1.41E-10 | 1.40E-09 | -2.26E-09 | 1.48E-07 | 5.41E-07 | 1.05E-06 | 1.29E-06 |
| 76 | 2.20E-11 | -1.29E-09 | -1.71E-09 | 1.83E-09 | 4.19E-10 | 1.19E-07 | 5.25E-07 | 1.03E-06 | 1.27E-06 |
| 77 | 1.19E-10 | -6.52E-10 | -1.66E-09 | 1.86E-09 | -4.95E-10 | 9.46E-08 | 5.05E-07 | 9.89E-07 | 1.24E-06 |
| 78 | 6.70E-11 | -8.95E-10 | 5.23E-10 | -5.33E-10 | 1.34E-09 | 6.58E-08 | 4.83E-07 | 9.62E-07 | 1.22E-06 |
| 79 | 1.65E-10 | 1.70E-09 | 6.74E-10 | 5.14E-10 | -1.87E-10 | 3.90E-08 | 4.65E-07 | 9.34E-07 | 1.19E-06 |
| 80 | -1.68E-10 | -6.31E-10 | 1.61E-09 | 1.27E-09 | -4.25E-10 | 1.58E-08 | 4.44E-07 | 9.04E-07 | 1.17E-06 |
| 81 | 1.22E-10 | 3.67E-10 | -7.88E-10 | 8.12E-10 | 2.33E-10 | 2.88E-11 | 4.20E-07 | 8.67E-07 | 1.14E-06 |
| 82 | -8.53E-12 | -1.06E-09 | -1.41E-09 | -4.12E-10 | -8.41E-10 | -1.39E-09 | 3.96E-07 | 8.38E-07 | 1.12E-06 |
| 83 | -7.29E-11 | -1.36E-09 | 7.63E-10 | 1.67E-09 | -3.05E-10 | -4.09E-10 | 3.74E-07 | 8.09E-07 | 1.10E-06 |
| 84 | 9.10E-11 | -1.44E-09 | -5.97E-10 | 4.22E-10 | 2.23E-08 | 1.17E-07 | 3.28E-07 | 8.17E-07 | 1.07E-06 |
| 85 | -1.00E-10 | -1.57E-09 | 1.23E-09 | -1.38E-09 | -7.30E-11 | -2.55E-09 | 3.22E-07 | 7.57E-07 | 1.05E-06 |
| 86 | -1.09E-10 | 1.97E-09 | 1.16E-09 | 1.16E-10 | -2.61E-08 | 4.59E-08 | 2.67E-07 | 6.97E-07 | 1.01E-06 |
| 87 | 8.39E-11 | 1.60E-09 | 1.20E-09 | -3.44E-10 | -5.04E-10 | -3.93E-11 | 2.80E-07 | 7.16E-07 | 9.79E-07 |
| 88 | 1.98E-10 | -6.80E-10 | 1.50E-09 | 2.08E-10 | 1.50E-09 | -3.86E-10 | 2.57E-07 | 6.98E-07 | 9.75E-07 |
| 89 | 1.56E-10 | 6.15E-10 | -1.16E-09 | 1.64E-09 | 2.92E-11 | 6.61E-11 | 2.33E-07 | 6.78E-07 | 9.51E-07 |
| 90 | 1.63E-10 | -1.11E-10 | 5.60E-10 | -1.51E-09 | 6.43E-11 | -7.42E-10 | 2.09E-07 | 6.59E-07 | 9.29E-07 |
| 91 | -1.14E-10 | 3.09E-10 | -5.60E-11 | -1.66E-09 | -2.98E-09 | 7.29E-10 | 1.87E-07 | 6.41E-07 | 9.11E-07 |
| 92 | 1.97E-11 | -1.96E-09 | -7.97E-10 | 1.71E-09 | -7.41E-12 | 6.92E-12 | 1.68E-07 | 6.21E-07 | 8.87E-07 |
| 93 | -7.02E-11 | -1.32E-09 | -1.99E-09 | -5.85E-10 | 1.44E-11 | -7.37E-11 | 1.49E-07 | 5.99E-07 | 8.61E-07 |
| 94 | 1.37E-10 | -1.96E-10 | 9.34E-10 | -1.51E-10 | -6.45E-10 | -1.16E-09 | 1.31E-07 | 5.80E-07 | 8.37E-07 |
| 95 | -1.29E-10 | 9.16E-10 | 1.44E-09 | 6.94E-10 | -1.04E-10 | -2.68E-10 | 1.11E-07 | 5.58E-07 | 8.08E-07 |
| 96 | -9.15E-11 | -1.79E-09 | -8.05E-10 | 1.38E-09 | -4.63E-09 | -8.81E-09 | 7.56E-08 | 5.39E-07 | 8.04E-07 |
| 97 | 1.90E-10 | -1.62E-09 | 1.89E-09 | -5.33E-10 | 3.52E-10 | 1.57E-09 | 7.28E-08 | 5.16E-07 | 7.58E-07 |
| 98 | 1.88E-10 | -1.54E-11 | 1.13E-09 | 3.43E-10 | -1.85E-10 | 1.28E-10 | 4.97E-08 | 4.89E-07 | 7.32E-07 |
| 99 | -1.35E-10 | 1.00E-09 | -2.42E-10 | -1.29E-09 | 1.01E-10 | 3.54E-10 | 2.95E-08 | 4.69E-07 | 7.14E-07 |
| 100 | -6.05E-11 | -7.87E-10 | 1.95E-09 | 1.55E-09 | -1.03E-11 | -2.11E-11 | 1.17E-08 | 4.52E-07 | 6.98E-07 |
| 101 | 9.87E-11 | 1.07E-09 | 1.00E-09 | -1.63E-09 | 1.91E-09 | 1.68E-09 | -1.56E-09 | 4.41E-07 | 6.84E-07 |
| 102 | -8.07E-11 | -5.42E-10 | 1.59E-09 | -2.50E-11 | 1.12E-11 | -2.60E-11 | -1.43E-10 | 4.24E-07 | 6.65E-07 |
| 103 | 4.35E-11 | 5.14E-10 | 3.31E-10 | -7.81E-10 | 7.62E-11 | 3.43E-11 | -8.65E-11 | 4.08E-07 | 6.48E-07 |
| 104 | 1.45E-10 | -1.34E-09 | 1.42E-09 | -1.55E-09 | 6.09E-11 | 7.02E-11 | -1.00E-10 | 3.91E-07 | 6.30E-07 |
| 105 | -8.60E-11 | -5.07E-10 | 1.29E-09 | -2.98E-10 | 4.28E-11 | -3.14E-11 | 1.42E-11 | 3.72E-07 | 6.10E-07 |
| 106 | 9.22E-11 | -1.16E-09 | 1.54E-09 | -9.49E-10 | 1.28E-11 | -4.45E-12 | 8.41E-11 | 3.52E-07 | 5.92E-07 |
| 107 | -9.17E-11 | -1.98E-09 | -1.24E-09 | 5.11E-10 | 5.26E-11 | 9.34E-11 | -6.51E-11 | 3.32E-07 | 5.75E-07 |
| 108 | 1.13E-10 | 5.60E-10 | 8.51E-10 | 1.32E-09 | 1.19E-11 | 1.49E-12 | -4.08E-11 | 3.19E-07 | 5.59E-07 |
| 109 | 1.76E-10 | 1.68E-09 | 4.88E-10 | -1.19E-09 | 7.19E-11 | 1.17E-11 | 2.10E-11 | 3.04E-07 | 5.45E-07 |
| 110 | -6.61E-11 | -1.74E-09 | -1.78E-09 | 4.97E-10 | -1.53E-11 | -6.36E-11 | 1.89E-10 | 2.93E-07 | 5.32E-07 |
| 111 | 1.36E-10 | -1.17E-09 | 1.86E-09 | -1.85E-09 | 1.30E-10 | 1.96E-11 | -7.33E-11 | 2.80E-07 | 5.18E-07 |
| 112 | -1.30E-10 | 1.27E-09 | 8.69E-10 | 1.95E-09 | -1.91E-11 | -1.25E-10 | 4.25E-11 | 2.64E-07 | 5.05E-07 |
| 113 | -4.01E-11 | -1.59E-09 | -9.75E-10 | 1.44E-09 | -2.29E-10 | -1.25E-09 | -6.50E-10 | 2.44E-07 | 4.86E-07 |
| 114 | -1.96E-10 | 1.07E-10 | -9.21E-10 | -1.64E-09 | -1.57E-10 | -6.23E-10 | -1.69E-09 | 2.23E-07 | 4.72E-07 |
| 115 | 1.95E-10 | -1.11E-09 | -1.30E-09 | -1.81E-09 | 8.83E-11 | -4.80E-11 | -2.99E-11 | 2.04E-07 | 4.49E-07 |
| 116 | -8.54E-11 | 1.33E-09 | -1.19E-09 | -1.49E-09 | 4.05E-11 | 1.03E-11 | -3.00E-11 | 1.86E-07 | 4.29E-07 |
| 117 | -1.55E-10 | -1.96E-09 | -9.00E-10 | 1.85E-09 | 2.14E-11 | -1.16E-09 | -1.70E-10 | 1.69E-07 | 4.10E-07 |
| 118 | 1.77E-10 | -1.64E-09 | -1.81E-10 | -1.64E-09 | 2.31E-11 | 1.33E-10 | -1.86E-10 | 1.55E-07 | 3.91E-07 |
| 119 | -1.59E-10 | -1.58E-09 | 5.03E-11 | -1.61E-09 | 1.47E-10 | -1.10E-10 | -6.76E-11 | 1.43E-07 | 3.75E-07 |
| 120 | -1.71E-10 | -1.37E-09 | 8.26E-10 | 1.72E-09 | -3.04E-11 | 1.87E-10 | -4.25E-12 | 1.32E-07 | 3.61E-07 |
| 121 | 5.67E-11 | 1.38E-09 | 8.93E-10 | -1.62E-09 | 8.77E-11 | -2.24E-11 | -1.60E-10 | 1.21E-07 | 3.49E-07 |
| 122 | -1.14E-10 | 8.06E-10 | -1.63E-09 | 3.01E-10 | 1.93E-10 | 3.38E-10 | -1.38E-10 | 1.08E-07 | 3.35E-07 |
| 123 | 7.94E-11 | -1.66E-09 | 1.27E-09 | -6.20E-10 | 2.68E-11 | 4.39E-11 | -7.22E-11 | 9.50E-08 | 3.23E-07 |
| 124 | -1.46E-10 | -3.48E-10 | 1.85E-09 | 4.29E-10 | -8.22E-11 | -3.53E-11 | 1.21E-09 | 8.05E-08 | 3.10E-07 |
| 125 | -9.12E-11 | -1.68E-09 | 1.51E-09 | -1.30E-09 | -7.08E-11 | -2.30E-10 | -5.52E-11 | 6.78E-08 | 2.98E-07 |
| 126 | -5.58E-12 | -1.44E-09 | -9.81E-10 | -1.37E-09 | 3.70E-11 | 5.75E-11 | 2.80E-10 | 5.41E-08 | 2.85E-07 |
| 127 | -1.96E-10 | -3.53E-10 | 1.93E-10 | 1.37E-09 | 3.66E-11 | 1.28E-10 | 3.43E-10 | 4.14E-08 | 2.71E-07 |
| 128 | -1.93E-10 | -4.32E-10 | -1.16E-09 | 1.96E-09 | -5.25E-12 | -5.22E-11 | 2.31E-11 | 2.97E-08 | 2.57E-07 |
| 129 | -1.03E-10 | -5.35E-10 | -6.23E-10 | -1.67E-09 | -2.22E-11 | -5.22E-11 | -2.15E-11 | 1.77E-08 | 2.41E-07 |
| 130 | -9.67E-11 | 8.92E-10 | 9.97E-10 | 2.88E-11 | -3.60E-12 | -3.52E-11 | -2.03E-11 | 4.35E-09 | 2.26E-07 |
| 131 | -2.25E-12 | 4.88E-10 | -1.21E-09 | -1.14E-09 | 4.56E-10 | -2.68E-11 | -6.70E-10 | 4.06E-09 | 2.12E-07 |
| 132 | -1.45E-10 | 8.45E-11 | -8.21E-11 | 1.53E-09 | -7.84E-11 | -1.12E-10 | -1.49E-10 | 1.05E-10 | 1.99E-07 |
| 133 | -1.80E-10 | -1.88E-09 | 6.58E-10 | -1.30E-09 | -5.72E-12 | -1.69E-11 | -4.89E-11 | 5.42E-11 | 1.88E-07 |
| 134 | 1.71E-10 | -8.43E-10 | -2.97E-11 | -4.72E-10 | -5.39E-13 | -5.62E-13 | 3.14E-13 | -8.45E-13 | 1.74E-07 |
| 135 | 3.72E-11 | 9.45E-10 | -1.25E-09 | 8.59E-10 | -3.60E-11 | 1.18E-11 | 4.42E-11 | 6.04E-11 | 1.63E-07 |
| 136 | 1.77E-10 | 1.02E-09 | 1.99E-09 | -4.40E-10 | 3.52E-11 | 6.00E-11 | 4.72E-11 | 4.23E-10 | 1.52E-07 |
| 137 | 1.45E-10 | -6.51E-10 | 1.87E-09 | -4.16E-10 | 2.13E-11 | 1.13E-11 | -1.05E-10 | -2.02E-10 | 1.40E-07 |
| 138 | 5.73E-11 | 1.95E-10 | 4.31E-10 | 1.50E-09 | -4.93E-12 | -9.11E-12 | -2.33E-11 | -5.26E-11 | 1.30E-07 |
| 139 | 3.74E-11 | 1.05E-09 | -2.07E-10 | -1.87E-09 | 1.08E-11 | 1.42E-11 | 8.21E-12 | -5.91E-11 | 1.17E-07 |
| 140 | 1.66E-10 | -1.82E-09 | 4.80E-10 | -1.55E-10 | -1.12E-12 | -3.91E-12 | -7.56E-12 | -4.33E-12 | 1.06E-07 |
| 141 | -1.61E-10 | 1.44E-09 | 1.41E-09 | 1.19E-09 | -2.03E-12 | -4.78E-15 | 4.51E-12 | -5.78E-12 | 9.51E-08 |
| 142 | 1.42E-10 | 1.33E-09 | -6.62E-10 | -6.13E-10 | -1.03E-12 | 2.04E-12 | -4.84E-12 | -1.16E-10 | 8.49E-08 |
| 143 | 2.36E-11 | 1.20E-09 | 9.81E-10 | -7.82E-10 | 4.77E-13 | 5.46E-13 | -1.13E-11 | 9.31E-11 | 7.52E-08 |
| 144 | -4.13E-11 | 1.90E-09 | 4.15E-10 | -1.22E-09 | 5.10E-13 | 3.35E-12 | -2.32E-11 | -1.74E-11 | 6.58E-08 |
| 145 | -1.33E-10 | 1.55E-09 | -1.32E-09 | 1.31E-09 | 2.21E-12 | 2.83E-12 | -1.40E-12 | 2.11E-11 | 5.62E-08 |
| 146 | -1.70E-10 | 1.17E-10 | 8.30E-10 | -5.77E-10 | 3.03E-12 | 6.18E-12 | 3.53E-12 | 5.04E-12 | 4.68E-08 |
| 147 | 1.64E-10 | 6.81E-10 | -1.15E-09 | -6.45E-10 | 5.82E-12 | -8.42E-12 | -1.32E-10 | -3.64E-11 | 3.68E-08 |
| 148 | -8.42E-12 | 1.78E-09 | 1.81E-09 | 6.79E-10 | -5.98E-15 | 7.36E-13 | -9.09E-14 | 1.98E-12 | 2.62E-08 |
| 149 | -2.16E-11 | 7.84E-11 | -1.13E-09 | -2.50E-10 | 2.19E-13 | -1.13E-12 | -4.71E-12 | 5.22E-12 | 1.54E-08 |
| 150 | -7.53E-11 | -1.60E-09 | -1.73E-09 | 1.52E-09 | -1.74E-13 | -9.09E-13 | -7.83E-13 | -2.15E-12 | 3.64E-09 |

Table.18. Spectra predicted by MLP neural network: Al- 0.4mm

| energy bin (kev)/voltage(kv) | 20kv | 30kv | 40kv | 50kv | 60kv | 80kv | 100kv | 130kv | 150kv |
| --- | --- | --- | --- | --- | --- | --- | --- | --- | --- |
| 1 | 0 | 0 | 0 | 0 | 0 | 0 | 0 | 0 | 0 |
| 2 | -1.40E-14 | -1.68E-14 | -3.91E-15 | 2.72E-14 | 5.76E-14 | -2.70E-14 | -6.93E-14 | 8.00E-14 | 9.00E-14 |
| 3 | 4.98E-08 | 6.33E-09 | 5.65E-09 | 5.74E-09 | 5.68E-09 | 5.36E-09 | 5.70E-09 | 7.46E-09 | 8.26E-09 |
| 4 | 5.85E-07 | 4.21E-07 | 3.39E-07 | 2.98E-07 | 2.78E-07 | 2.62E-07 | 2.67E-07 | 2.80E-07 | 2.27E-07 |
| 5 | 1.67E-06 | 1.71E-06 | 1.67E-06 | 1.57E-06 | 1.45E-06 | 1.31E-06 | 1.31E-06 | 1.32E-06 | 1.21E-06 |
| 6 | 1.96E-06 | 2.88E-06 | 3.30E-06 | 3.35E-06 | 3.20E-06 | 2.87E-06 | 2.74E-06 | 2.63E-06 | 2.41E-06 |
| 7 | 2.45E-06 | 3.68E-06 | 4.38E-06 | 4.61E-06 | 4.66E-06 | 4.52E-06 | 4.35E-06 | 4.29E-06 | 3.58E-06 |
| 8 | 2.74E-06 | 4.05E-06 | 4.98E-06 | 5.54E-06 | 5.83E-06 | 6.03E-06 | 6.03E-06 | 5.73E-06 | 5.36E-06 |
| 9 | 2.38E-06 | 5.10E-06 | 7.10E-06 | 8.68E-06 | 9.97E-06 | 1.16E-05 | 1.26E-05 | 1.46E-05 | 1.29E-05 |
| 10 | 2.36E-06 | 3.67E-06 | 4.98E-06 | 6.07E-06 | 6.87E-06 | 7.84E-06 | 8.56E-06 | 9.37E-06 | 8.64E-06 |
| 11 | 2.01E-06 | 3.01E-06 | 3.88E-06 | 4.48E-06 | 4.84E-06 | 5.12E-06 | 5.13E-06 | 5.68E-06 | 4.78E-06 |
| 12 | 1.64E-06 | 2.69E-06 | 3.43E-06 | 3.89E-06 | 4.15E-06 | 4.29E-06 | 4.32E-06 | 4.57E-06 | 3.80E-06 |
| 13 | 1.38E-06 | 2.29E-06 | 2.97E-06 | 3.36E-06 | 3.54E-06 | 3.59E-06 | 3.61E-06 | 3.63E-06 | 3.11E-06 |
| 14 | 1.13E-06 | 2.11E-06 | 2.87E-06 | 3.36E-06 | 3.65E-06 | 3.91E-06 | 4.01E-06 | 4.05E-06 | 4.00E-06 |
| 15 | 9.88E-07 | 1.96E-06 | 2.74E-06 | 3.24E-06 | 3.54E-06 | 3.94E-06 | 4.09E-06 | 4.42E-06 | 3.77E-06 |
| 16 | 8.63E-07 | 1.83E-06 | 2.58E-06 | 3.16E-06 | 3.61E-06 | 4.22E-06 | 4.52E-06 | 4.56E-06 | 4.21E-06 |
| 17 | 6.04E-07 | 1.69E-06 | 2.50E-06 | 3.08E-06 | 3.49E-06 | 4.03E-06 | 4.36E-06 | 4.87E-06 | 4.35E-06 |
| 18 | 4.39E-07 | 1.50E-06 | 2.32E-06 | 2.94E-06 | 3.40E-06 | 4.02E-06 | 4.45E-06 | 5.05E-06 | 4.59E-06 |
| 19 | 2.70E-07 | 1.37E-06 | 2.20E-06 | 2.83E-06 | 3.32E-06 | 4.02E-06 | 4.51E-06 | 5.10E-06 | 4.80E-06 |
| 20 | 1.06E-07 | 1.18E-06 | 2.07E-06 | 2.73E-06 | 3.21E-06 | 3.93E-06 | 4.55E-06 | 5.30E-06 | 4.97E-06 |
| 21 | -1.05E-08 | 1.07E-06 | 1.89E-06 | 2.54E-06 | 3.07E-06 | 3.90E-06 | 4.55E-06 | 5.35E-06 | 5.12E-06 |
| 22 | 3.32E-09 | 9.47E-07 | 1.75E-06 | 2.41E-06 | 2.96E-06 | 3.80E-06 | 4.49E-06 | 5.44E-06 | 5.21E-06 |
| 23 | -6.42E-08 | 7.76E-07 | 1.55E-06 | 2.24E-06 | 2.81E-06 | 3.70E-06 | 4.44E-06 | 5.38E-06 | 5.39E-06 |
| 24 | 6.86E-09 | 7.33E-07 | 1.46E-06 | 2.12E-06 | 2.69E-06 | 3.59E-06 | 4.39E-06 | 5.33E-06 | 5.32E-06 |
| 25 | -7.42E-09 | 6.32E-07 | 1.32E-06 | 1.98E-06 | 2.57E-06 | 3.49E-06 | 4.33E-06 | 5.29E-06 | 5.34E-06 |
| 26 | -1.44E-08 | 5.36E-07 | 1.19E-06 | 1.86E-06 | 2.47E-06 | 3.42E-06 | 4.23E-06 | 5.22E-06 | 5.32E-06 |
| 27 | -7.86E-09 | 4.05E-07 | 1.10E-06 | 1.73E-06 | 2.30E-06 | 3.24E-06 | 4.07E-06 | 5.12E-06 | 5.31E-06 |
| 28 | 6.90E-08 | 3.63E-07 | 9.90E-07 | 1.65E-06 | 2.20E-06 | 3.12E-06 | 3.96E-06 | 5.04E-06 | 5.25E-06 |
| 29 | 4.04E-10 | 1.76E-07 | 9.06E-07 | 1.53E-06 | 2.07E-06 | 3.02E-06 | 3.84E-06 | 4.93E-06 | 5.22E-06 |
| 30 | -6.34E-08 | 2.00E-07 | 7.10E-07 | 1.32E-06 | 1.92E-06 | 2.96E-06 | 3.78E-06 | 4.74E-06 | 5.27E-06 |
| 31 | -1.44E-07 | 1.93E-07 | 6.65E-07 | 1.23E-06 | 1.81E-06 | 2.80E-06 | 3.56E-06 | 4.55E-06 | 5.07E-06 |
| 32 | -1.01E-09 | 5.73E-09 | 6.59E-07 | 1.24E-06 | 1.74E-06 | 2.67E-06 | 3.52E-06 | 4.55E-06 | 5.00E-06 |
| 33 | 4.41E-08 | -4.23E-09 | 5.67E-07 | 1.16E-06 | 1.66E-06 | 2.55E-06 | 3.37E-06 | 4.39E-06 | 4.92E-06 |
| 34 | -2.71E-09 | -1.40E-09 | 4.86E-07 | 1.08E-06 | 1.56E-06 | 2.43E-06 | 3.29E-06 | 4.26E-06 | 4.83E-06 |
| 35 | -2.08E-08 | 2.94E-08 | 3.80E-07 | 9.79E-07 | 1.46E-06 | 2.32E-06 | 3.16E-06 | 4.15E-06 | 4.71E-06 |
| 36 | 2.41E-09 | 7.29E-08 | 3.42E-07 | 9.10E-07 | 1.39E-06 | 2.21E-06 | 3.04E-06 | 4.02E-06 | 4.61E-06 |
| 37 | -3.52E-07 | -6.79E-09 | 3.59E-07 | 7.80E-07 | 1.25E-06 | 2.19E-06 | 2.86E-06 | 3.92E-06 | 4.48E-06 |
| 38 | -1.55E-09 | -1.25E-08 | 1.84E-07 | 7.67E-07 | 1.22E-06 | 2.01E-06 | 2.81E-06 | 3.83E-06 | 4.43E-06 |
| 39 | -1.35E-09 | -4.02E-09 | 1.16E-07 | 6.90E-07 | 1.15E-06 | 1.92E-06 | 2.71E-06 | 3.70E-06 | 4.21E-06 |
| 40 | -8.86E-10 | -2.28E-09 | 5.76E-08 | 6.04E-07 | 1.07E-06 | 1.84E-06 | 2.60E-06 | 3.60E-06 | 4.19E-06 |
| 41 | -8.30E-10 | 5.07E-09 | 6.21E-09 | 5.55E-07 | 1.00E-06 | 1.77E-06 | 2.51E-06 | 3.49E-06 | 3.96E-06 |
| 42 | 5.22E-10 | 1.82E-09 | -1.03E-09 | 4.87E-07 | 9.41E-07 | 1.69E-06 | 2.41E-06 | 3.38E-06 | 3.91E-06 |
| 43 | 1.27E-09 | -1.89E-08 | -1.33E-08 | 4.05E-07 | 8.74E-07 | 1.63E-06 | 2.33E-06 | 3.28E-06 | 3.83E-06 |
| 44 | 3.53E-09 | -1.74E-09 | 2.39E-09 | 3.74E-07 | 8.00E-07 | 1.57E-06 | 2.24E-06 | 3.18E-06 | 3.71E-06 |
| 45 | 4.32E-09 | -3.33E-09 | -3.00E-09 | 3.12E-07 | 7.54E-07 | 1.50E-06 | 2.17E-06 | 3.07E-06 | 3.63E-06 |
| 46 | 2.14E-08 | 9.28E-09 | 8.62E-10 | 2.60E-07 | 6.97E-07 | 1.42E-06 | 2.08E-06 | 2.99E-06 | 3.53E-06 |
| 47 | 1.50E-08 | -1.80E-09 | -1.86E-09 | 2.03E-07 | 6.42E-07 | 1.35E-06 | 2.02E-06 | 2.90E-06 | 3.43E-06 |
| 48 | 2.79E-09 | -2.18E-09 | -2.83E-09 | 1.49E-07 | 5.94E-07 | 1.32E-06 | 1.97E-06 | 2.81E-06 | 3.34E-06 |
| 49 | 3.01E-09 | 1.02E-08 | -2.58E-08 | 1.18E-07 | 5.32E-07 | 1.27E-06 | 1.87E-06 | 2.72E-06 | 3.25E-06 |
| 50 | 1.92E-10 | -2.79E-10 | 2.76E-11 | 3.96E-08 | 4.90E-07 | 1.17E-06 | 1.80E-06 | 2.63E-06 | 3.17E-06 |
| 51 | -1.14E-08 | 2.51E-08 | -3.96E-08 | 7.95E-09 | 4.28E-07 | 1.17E-06 | 1.73E-06 | 2.57E-06 | 3.08E-06 |
| 52 | -2.61E-08 | -2.27E-08 | -8.04E-09 | 7.34E-08 | 3.88E-07 | 1.05E-06 | 1.67E-06 | 2.48E-06 | 3.01E-06 |
| 53 | 1.77E-08 | -1.38E-08 | -1.09E-09 | 1.05E-07 | 3.36E-07 | 1.05E-06 | 1.63E-06 | 2.41E-06 | 2.68E-06 |
| 54 | -5.13E-09 | 1.08E-08 | -1.36E-08 | 3.83E-08 | 2.61E-07 | 9.54E-07 | 1.58E-06 | 2.37E-06 | 2.85E-06 |
| 55 | -4.67E-09 | 3.69E-09 | -9.96E-09 | 1.28E-08 | 2.49E-07 | 8.94E-07 | 1.51E-06 | 2.29E-06 | 2.80E-06 |
| 56 | 1.57E-09 | 3.99E-08 | -1.42E-08 | 2.29E-08 | 2.14E-07 | 8.09E-07 | 1.42E-06 | 2.23E-06 | 2.72E-06 |
| 57 | -2.32E-09 | 6.72E-09 | -7.05E-09 | 4.65E-09 | 1.55E-07 | 8.04E-07 | 1.39E-06 | 2.15E-06 | 2.67E-06 |
| 58 | -3.71E-09 | 7.26E-09 | -1.50E-08 | -1.14E-08 | 1.28E-07 | 7.61E-07 | 1.32E-06 | 2.07E-06 | 2.59E-06 |
| 59 | -1.44E-09 | 1.06E-09 | 2.22E-09 | -8.09E-10 | 7.32E-08 | 8.75E-07 | 2.31E-06 | 4.99E-06 | 6.75E-06 |
| 60 | -2.02E-09 | 2.06E-08 | 1.01E-08 | -1.85E-08 | 4.78E-08 | 9.77E-07 | 3.06E-06 | 7.34E-06 | 1.02E-05 |
| 61 | -2.91E-09 | 6.95E-09 | 2.75E-09 | -2.41E-08 | 1.07E-08 | 6.05E-07 | 1.13E-06 | 1.93E-06 | 2.41E-06 |
| 62 | 2.07E-09 | -2.33E-09 | 2.34E-09 | -9.39E-10 | 1.85E-09 | 6.14E-07 | 1.23E-06 | 2.00E-06 | 2.64E-06 |
| 63 | -5.27E-10 | 1.67E-09 | 2.75E-09 | -2.38E-09 | 5.93E-10 | 6.11E-07 | 1.07E-06 | 1.67E-06 | 2.15E-06 |
| 64 | -1.74E-09 | -2.85E-11 | 2.78E-10 | -3.44E-09 | -4.37E-09 | 5.28E-07 | 1.05E-06 | 1.59E-06 | 2.10E-06 |
| 65 | 2.46E-10 | 2.42E-10 | 2.88E-10 | -4.69E-10 | -1.23E-09 | 4.94E-07 | 1.00E-06 | 1.60E-06 | 2.07E-06 |
| 66 | -1.83E-09 | 2.64E-08 | -5.17E-10 | -2.08E-08 | 3.29E-08 | 4.03E-07 | 9.20E-07 | 1.55E-06 | 1.98E-06 |
| 67 | 5.28E-10 | -1.03E-10 | -6.08E-10 | -8.41E-10 | 4.94E-10 | 4.46E-07 | 8.70E-07 | 1.51E-06 | 2.00E-06 |
| 68 | -3.52E-09 | -3.04E-09 | -1.99E-09 | 4.51E-10 | 8.04E-09 | 4.98E-07 | 1.55E-06 | 3.33E-06 | 4.73E-06 |
| 69 | -7.96E-10 | 1.37E-09 | 1.57E-09 | -4.99E-09 | -1.86E-09 | 3.48E-07 | 8.23E-07 | 1.47E-06 | 1.80E-06 |
| 70 | 7.92E-10 | 2.29E-09 | 3.82E-09 | 3.11E-10 | -1.35E-09 | 3.37E-07 | 9.68E-07 | 1.89E-06 | 2.52E-06 |
| 71 | 1.18E-09 | 1.17E-09 | 3.32E-10 | -5.56E-10 | 1.93E-09 | 2.85E-07 | 7.62E-07 | 1.28E-06 | 1.50E-06 |
| 72 | 2.55E-09 | 1.21E-09 | 3.43E-10 | -2.58E-10 | -4.35E-10 | 2.54E-07 | 6.80E-07 | 1.25E-06 | 1.54E-06 |
| 73 | -1.29E-09 | 4.30E-09 | 3.00E-09 | -4.55E-09 | 5.90E-09 | 2.24E-07 | 6.46E-07 | 1.19E-06 | 1.48E-06 |
| 74 | -1.38E-09 | 2.04E-09 | 3.50E-09 | -1.54E-10 | 7.01E-09 | 1.97E-07 | 6.16E-07 | 1.16E-06 | 1.44E-06 |
| 75 | -9.86E-10 | 5.09E-10 | 5.23E-10 | -2.22E-09 | -1.15E-09 | 1.60E-07 | 5.88E-07 | 1.13E-06 | 1.40E-06 |
| 76 | -3.14E-10 | -3.63E-11 | 6.68E-10 | -1.99E-10 | 9.85E-12 | 1.31E-07 | 5.63E-07 | 1.10E-06 | 1.36E-06 |
| 77 | -1.97E-10 | 5.56E-10 | 7.20E-10 | -2.53E-10 | 4.94E-10 | 1.02E-07 | 5.41E-07 | 1.07E-06 | 1.33E-06 |
| 78 | 4.34E-10 | 4.40E-10 | -3.43E-12 | -1.25E-09 | -1.78E-09 | 7.56E-08 | 5.22E-07 | 1.03E-06 | 1.30E-06 |
| 79 | 2.64E-10 | 1.96E-10 | 1.24E-10 | 1.89E-10 | 1.07E-09 | 4.71E-08 | 4.97E-07 | 9.99E-07 | 1.27E-06 |
| 80 | -1.74E-09 | -7.89E-10 | 1.57E-09 | 3.89E-09 | -1.03E-09 | 1.69E-08 | 4.76E-07 | 9.63E-07 | 1.25E-06 |
| 81 | 4.47E-11 | 9.28E-10 | 9.78E-10 | 4.34E-10 | -8.40E-11 | 3.80E-10 | 4.48E-07 | 9.24E-07 | 1.22E-06 |
| 82 | 1.36E-10 | 1.39E-10 | 8.63E-11 | -9.31E-11 | -5.29E-10 | 1.82E-09 | 4.23E-07 | 8.93E-07 | 1.19E-06 |
| 83 | -1.31E-10 | 2.24E-11 | 1.49E-10 | 1.58E-10 | -2.74E-10 | -2.89E-10 | 3.97E-07 | 8.63E-07 | 1.17E-06 |
| 84 | 1.00E-08 | 5.24E-09 | 4.12E-09 | 8.51E-09 | 2.17E-08 | 1.00E-07 | 3.08E-07 | 8.39E-07 | 1.13E-06 |
| 85 | 4.42E-10 | 1.69E-10 | -1.32E-10 | -4.33E-10 | -6.60E-10 | 7.47E-10 | 3.47E-07 | 8.04E-07 | 1.11E-06 |
| 86 | 2.15E-08 | 9.53E-09 | -1.76E-09 | -5.41E-09 | 2.50E-09 | 7.55E-08 | 2.79E-07 | 8.01E-07 | 1.07E-06 |
| 87 | -5.60E-10 | -4.96E-10 | -3.28E-10 | -2.59E-11 | 8.57E-11 | 1.70E-10 | 2.98E-07 | 7.66E-07 | 1.07E-06 |
| 88 | -1.49E-10 | 2.18E-09 | -2.83E-09 | -8.39E-10 | -4.28E-09 | 1.88E-09 | 2.73E-07 | 7.39E-07 | 1.03E-06 |
| 89 | -3.93E-11 | 1.04E-12 | -2.13E-11 | -4.84E-12 | -3.60E-11 | 1.16E-10 | 2.49E-07 | 7.20E-07 | 1.01E-06 |
| 90 | 9.29E-11 | -1.81E-10 | -1.68E-10 | 7.67E-11 | 2.19E-10 | -2.44E-10 | 2.22E-07 | 6.99E-07 | 9.85E-07 |
| 91 | 9.53E-10 | 3.90E-10 | -3.72E-10 | -1.35E-09 | -2.37E-09 | 3.09E-09 | 1.98E-07 | 6.83E-07 | 9.64E-07 |
| 92 | -4.97E-11 | -1.09E-11 | 7.05E-12 | 3.33E-12 | -2.48E-11 | 5.49E-11 | 1.78E-07 | 6.57E-07 | 9.39E-07 |
| 93 | -4.01E-11 | -7.79E-11 | 7.02E-12 | 2.73E-10 | 2.48E-10 | 2.45E-10 | 1.58E-07 | 6.35E-07 | 9.13E-07 |
| 94 | -3.09E-10 | -1.86E-10 | 6.48E-11 | 2.49E-10 | -5.17E-10 | -2.85E-10 | 1.39E-07 | 6.13E-07 | 8.84E-07 |
| 95 | -1.38E-11 | 7.16E-11 | -1.14E-10 | 6.67E-11 | 3.07E-11 | -2.16E-10 | 1.19E-07 | 5.90E-07 | 8.54E-07 |
| 96 | -1.90E-09 | -1.94E-09 | 1.26E-09 | 1.90E-09 | -2.31E-09 | 2.53E-09 | 1.02E-07 | 5.66E-07 | 8.21E-07 |
| 97 | 8.36E-11 | 2.18E-10 | 1.65E-10 | -2.69E-10 | -1.43E-09 | -4.43E-10 | 7.61E-08 | 5.38E-07 | 7.97E-07 |
| 98 | -1.08E-10 | -1.98E-11 | 5.38E-11 | 1.70E-11 | -3.28E-10 | -6.01E-10 | 5.34E-08 | 5.16E-07 | 7.75E-07 |
| 99 | 5.09E-11 | 4.47E-11 | 2.77E-11 | -1.38E-11 | -9.75E-11 | 2.82E-10 | 3.12E-08 | 4.95E-07 | 7.54E-07 |
| 100 | -4.72E-12 | -3.77E-11 | -1.03E-11 | 2.22E-11 | -7.64E-11 | -4.70E-11 | 1.26E-08 | 4.79E-07 | 7.36E-07 |
| 101 | 8.26E-10 | -4.92E-11 | -5.18E-10 | -7.64E-10 | -9.22E-10 | -1.07E-09 | 1.15E-09 | 4.61E-07 | 7.17E-07 |
| 102 | -3.39E-12 | -6.31E-12 | -6.76E-12 | -4.20E-13 | 1.99E-11 | -3.76E-12 | 1.17E-11 | 4.52E-07 | 7.01E-07 |
| 103 | -2.55E-11 | -2.86E-11 | -3.25E-11 | -3.86E-11 | -5.12E-11 | -1.55E-10 | -5.87E-11 | 4.29E-07 | 6.83E-07 |
| 104 | -2.65E-11 | 3.22E-11 | 3.46E-11 | 2.22E-11 | 3.73E-12 | -4.32E-11 | 1.40E-10 | 4.12E-07 | 6.64E-07 |
| 105 | -3.37E-13 | -1.39E-11 | -9.26E-12 | -7.91E-13 | -9.89E-13 | -1.31E-10 | -3.12E-12 | 3.91E-07 | 6.37E-07 |
| 106 | 2.30E-11 | -1.51E-11 | -8.38E-12 | 1.11E-12 | -5.17E-12 | -1.12E-10 | -1.15E-10 | 3.71E-07 | 6.23E-07 |
| 107 | 6.37E-12 | 2.87E-13 | 6.63E-12 | 1.55E-11 | 1.40E-11 | -8.93E-11 | -9.17E-11 | 3.50E-07 | 6.05E-07 |
| 108 | 1.55E-11 | 4.48E-12 | -1.16E-11 | -3.48E-11 | -6.80E-11 | -1.73E-10 | -7.79E-11 | 3.36E-07 | 5.88E-07 |
| 109 | -3.21E-11 | -5.65E-11 | -5.92E-11 | -3.74E-11 | -6.20E-12 | -8.49E-11 | 2.37E-10 | 3.20E-07 | 5.74E-07 |
| 110 | -1.42E-11 | -1.71E-11 | -1.83E-11 | -1.38E-11 | 4.18E-12 | 6.91E-11 | 1.16E-10 | 3.08E-07 | 5.59E-07 |
| 111 | 3.08E-11 | 1.52E-11 | 7.28E-12 | 1.14E-11 | 2.21E-11 | -2.40E-11 | 1.03E-11 | 2.94E-07 | 5.45E-07 |
| 112 | -3.18E-11 | -1.32E-11 | 2.14E-12 | 1.29E-11 | 1.65E-11 | -3.33E-11 | -1.97E-11 | 2.79E-07 | 5.30E-07 |
| 113 | 2.17E-10 | 4.06E-10 | 5.41E-10 | 5.43E-10 | 2.59E-10 | -1.35E-09 | -3.04E-11 | 2.55E-07 | 5.03E-07 |
| 114 | -7.32E-10 | -7.41E-10 | -7.17E-10 | -6.29E-10 | -4.77E-10 | -3.74E-10 | 1.42E-11 | 2.37E-07 | 4.93E-07 |
| 115 | 2.91E-10 | -3.78E-11 | -8.69E-11 | -9.09E-12 | 8.11E-11 | 1.29E-10 | -1.32E-10 | 2.15E-07 | 4.72E-07 |
| 116 | 1.00E-10 | 5.48E-11 | 1.64E-11 | 7.76E-12 | 3.68E-11 | 9.39E-11 | -5.20E-11 | 1.95E-07 | 4.50E-07 |
| 117 | 2.26E-10 | 4.65E-11 | 4.03E-11 | 3.16E-10 | 7.04E-10 | -6.80E-10 | 1.02E-10 | 1.80E-07 | 4.30E-07 |
| 118 | 1.34E-11 | 1.02E-11 | 1.72E-12 | -3.04E-11 | -1.06E-10 | -3.61E-10 | 5.52E-12 | 1.62E-07 | 4.11E-07 |
| 119 | -6.27E-11 | -4.13E-11 | -1.78E-11 | 4.90E-12 | 1.82E-11 | -1.11E-10 | -3.97E-11 | 1.50E-07 | 3.94E-07 |
| 120 | 6.47E-12 | 3.66E-12 | 5.72E-13 | -2.49E-12 | -5.55E-12 | -1.93E-11 | 1.16E-10 | 1.39E-07 | 3.79E-07 |
| 121 | 9.58E-11 | -9.22E-12 | -1.13E-10 | -1.14E-10 | 2.38E-11 | -2.51E-10 | -4.97E-10 | 1.27E-07 | 3.64E-07 |
| 122 | -2.40E-10 | -1.88E-10 | -1.49E-10 | -1.09E-10 | -5.36E-12 | -1.73E-10 | -5.08E-13 | 1.14E-07 | 3.52E-07 |
| 123 | 3.11E-11 | 2.89E-11 | 2.80E-11 | 2.60E-11 | 1.45E-11 | -1.64E-10 | 2.18E-11 | 9.96E-08 | 3.40E-07 |
| 124 | -1.87E-11 | 3.63E-11 | 6.79E-11 | 7.66E-11 | 6.16E-11 | -8.88E-11 | -1.82E-10 | 8.58E-08 | 3.26E-07 |
| 125 | -2.87E-11 | -2.84E-11 | -2.21E-11 | -1.07E-11 | 3.44E-12 | 9.55E-12 | 2.60E-10 | 7.09E-08 | 3.13E-07 |
| 126 | 1.00E-11 | 2.70E-11 | 5.46E-11 | 9.55E-11 | 1.39E-10 | 3.29E-12 | -1.22E-10 | 5.68E-08 | 2.99E-07 |
| 127 | 1.76E-11 | 1.19E-11 | 1.00E-11 | 1.29E-11 | 1.94E-11 | -1.12E-11 | -3.00E-11 | 4.39E-08 | 2.85E-07 |
| 128 | 6.81E-11 | -4.54E-13 | -1.89E-11 | -3.12E-12 | 2.61E-11 | -4.05E-12 | 3.30E-11 | 3.12E-08 | 2.69E-07 |
| 129 | -9.52E-12 | -7.91E-12 | -8.21E-12 | -1.15E-11 | -1.81E-11 | -3.86E-11 | 6.75E-11 | 1.98E-08 | 2.53E-07 |
| 130 | -2.82E-11 | -2.88E-11 | -2.77E-11 | -2.45E-11 | -2.08E-11 | -2.21E-11 | -1.04E-11 | 1.11E-08 | 2.37E-07 |
| 131 | 9.65E-10 | 9.58E-10 | 9.20E-10 | 8.22E-10 | 6.15E-10 | -5.27E-10 | -5.43E-10 | 3.63E-10 | 2.22E-07 |
| 132 | 6.82E-11 | 4.86E-11 | 3.15E-11 | 1.76E-11 | 6.86E-12 | -9.63E-12 | -4.04E-11 | 1.74E-11 | 2.08E-07 |
| 133 | -3.98E-13 | -6.59E-12 | -6.19E-12 | -1.87E-12 | 3.24E-12 | 1.38E-11 | 2.47E-11 | 9.01E-11 | 1.95E-07 |
| 134 | 1.87E-12 | 1.86E-12 | 1.73E-12 | 1.44E-12 | 1.02E-12 | 1.99E-12 | 7.25E-13 | -3.63E-13 | 1.83E-07 |
| 135 | 2.73E-11 | 1.34E-11 | -4.45E-12 | -2.61E-11 | -4.86E-11 | -5.97E-11 | -9.90E-11 | 4.85E-12 | 1.70E-07 |
| 136 | 5.12E-12 | 6.17E-12 | 1.54E-11 | 3.76E-11 | 6.95E-11 | 1.04E-10 | -2.62E-11 | -4.36E-10 | 1.58E-07 |
| 137 | -1.61E-11 | 5.17E-12 | 2.36E-11 | 3.61E-11 | 4.03E-11 | 7.96E-12 | -1.37E-10 | -2.49E-10 | 1.46E-07 |
| 138 | -3.36E-12 | -2.64E-12 | -3.58E-12 | -5.82E-12 | -9.11E-12 | -1.85E-11 | -4.94E-11 | -1.57E-10 | 1.34E-07 |
| 139 | 8.60E-11 | 3.46E-12 | -4.72E-12 | 1.33E-11 | 3.15E-11 | 3.65E-11 | 3.48E-12 | 6.04E-13 | 1.22E-07 |
| 140 | 1.75E-12 | 9.39E-13 | -8.99E-14 | -1.53E-12 | -3.51E-12 | -7.82E-12 | 7.73E-12 | -1.00E-11 | 1.11E-07 |
| 141 | 4.69E-12 | 1.73E-12 | -7.83E-13 | -2.48E-12 | -2.93E-12 | 8.04E-13 | 9.57E-13 | -1.02E-11 | 9.94E-08 |
| 142 | -2.05E-13 | -2.56E-13 | -5.52E-13 | -1.22E-12 | -1.88E-12 | 5.83E-13 | -2.35E-12 | -5.78E-12 | 8.87E-08 |
| 143 | -1.40E-12 | -1.41E-12 | -1.46E-12 | -1.57E-12 | -1.81E-12 | -3.69E-12 | -1.49E-11 | 3.13E-11 | 7.86E-08 |
| 144 | 9.65E-12 | 5.22E-12 | 2.44E-12 | 3.72E-12 | 8.11E-12 | 1.79E-11 | -2.60E-12 | 6.72E-13 | 6.87E-08 |
| 145 | -1.56E-12 | -6.51E-13 | -6.41E-14 | 2.97E-13 | 4.82E-13 | 1.88E-13 | -3.05E-12 | 1.06E-11 | 5.90E-08 |
| 146 | -3.12E-12 | -1.92E-12 | -6.48E-13 | 7.32E-13 | 2.25E-12 | 5.65E-12 | 1.98E-12 | 5.06E-12 | 4.89E-08 |
| 147 | -2.81E-11 | -1.15E-11 | 4.46E-12 | 1.73E-11 | 2.60E-11 | 2.76E-11 | -3.13E-11 | 6.48E-11 | 3.84E-08 |
| 148 | 6.09E-13 | 4.41E-13 | 3.14E-13 | 2.21E-13 | 1.58E-13 | 1.49E-14 | -2.49E-12 | -2.88E-12 | 2.73E-08 |
| 149 | 4.82E-14 | -1.01E-12 | -2.10E-12 | -3.20E-12 | -4.30E-12 | -6.96E-12 | -1.20E-11 | -5.34E-12 | 1.60E-08 |
| 150 | 4.07E-13 | 2.05E-13 | 1.15E-13 | 7.70E-14 | 5.43E-14 | 5.17E-14 | -2.68E-13 | 5.04E-13 | 3.80E-09 |

Table.19. Spectra predicted by MLP neural network: Al- 0.8mm

| energy bin (kev)/voltage(kv) | 20kv | 30kv | 40kv | 50kv | 60kv | 80kv | 100kv | 130kv | 150kv |
| --- | --- | --- | --- | --- | --- | --- | --- | --- | --- |
| 1 | 0 | 0 | 0 | 0 | 0 | 0 | 0 | 0 | 0 |
| 2 | -1.73E-14 | -1.63E-14 | -9.95E-15 | 2.04E-15 | 1.37E-14 | -2.78E-14 | -2.67E-14 | 1.56E-13 | 7.91E-14 |
| 3 | 1.05E-09 | 1.13E-09 | 1.20E-09 | 1.24E-09 | 1.24E-09 | 1.14E-09 | 1.15E-09 | 1.29E-09 | 1.80E-09 |
| 4 | 2.64E-07 | 2.29E-07 | 2.00E-07 | 1.77E-07 | 1.58E-07 | 1.30E-07 | 1.23E-07 | 1.52E-07 | 1.49E-07 |
| 5 | 1.07E-06 | 1.16E-06 | 1.17E-06 | 1.12E-06 | 1.05E-06 | 9.43E-07 | 9.28E-07 | 9.71E-07 | 9.09E-07 |
| 6 | 1.63E-06 | 2.44E-06 | 2.77E-06 | 2.77E-06 | 2.63E-06 | 2.40E-06 | 2.32E-06 | 2.25E-06 | 2.06E-06 |
| 7 | 2.14E-06 | 3.22E-06 | 3.87E-06 | 4.04E-06 | 4.01E-06 | 3.79E-06 | 3.71E-06 | 3.80E-06 | 3.22E-06 |
| 8 | 2.47E-06 | 3.72E-06 | 4.59E-06 | 5.11E-06 | 5.37E-06 | 5.49E-06 | 5.39E-06 | 5.04E-06 | 4.68E-06 |
| 9 | 2.34E-06 | 4.84E-06 | 6.68E-06 | 8.14E-06 | 9.34E-06 | 1.09E-05 | 1.18E-05 | 1.37E-05 | 1.21E-05 |
| 10 | 2.16E-06 | 3.58E-06 | 4.79E-06 | 5.71E-06 | 6.41E-06 | 7.50E-06 | 8.34E-06 | 8.90E-06 | 8.03E-06 |
| 11 | 1.90E-06 | 2.89E-06 | 3.72E-06 | 4.29E-06 | 4.63E-06 | 4.90E-06 | 4.93E-06 | 5.36E-06 | 4.57E-06 |
| 12 | 1.57E-06 | 2.60E-06 | 3.32E-06 | 3.77E-06 | 4.02E-06 | 4.16E-06 | 4.16E-06 | 4.38E-06 | 3.63E-06 |
| 13 | 1.33E-06 | 2.22E-06 | 2.89E-06 | 3.26E-06 | 3.44E-06 | 3.49E-06 | 3.49E-06 | 3.50E-06 | 3.00E-06 |
| 14 | 1.14E-06 | 2.05E-06 | 2.74E-06 | 3.21E-06 | 3.51E-06 | 3.78E-06 | 3.85E-06 | 3.83E-06 | 3.74E-06 |
| 15 | 9.96E-07 | 1.90E-06 | 2.66E-06 | 3.17E-06 | 3.46E-06 | 3.84E-06 | 3.99E-06 | 4.28E-06 | 3.66E-06 |
| 16 | 7.72E-07 | 1.80E-06 | 2.55E-06 | 3.07E-06 | 3.44E-06 | 4.01E-06 | 4.36E-06 | 4.43E-06 | 4.02E-06 |
| 17 | 5.89E-07 | 1.64E-06 | 2.42E-06 | 2.98E-06 | 3.40E-06 | 3.94E-06 | 4.26E-06 | 4.76E-06 | 4.26E-06 |
| 18 | 4.49E-07 | 1.48E-06 | 2.28E-06 | 2.88E-06 | 3.33E-06 | 3.94E-06 | 4.37E-06 | 4.95E-06 | 4.50E-06 |
| 19 | 3.21E-07 | 1.35E-06 | 2.14E-06 | 2.74E-06 | 3.22E-06 | 3.93E-06 | 4.44E-06 | 5.10E-06 | 4.71E-06 |
| 20 | 1.07E-07 | 1.17E-06 | 2.00E-06 | 2.62E-06 | 3.09E-06 | 3.81E-06 | 4.44E-06 | 5.22E-06 | 4.88E-06 |
| 21 | 2.77E-08 | 1.07E-06 | 1.87E-06 | 2.50E-06 | 3.02E-06 | 3.82E-06 | 4.46E-06 | 5.25E-06 | 5.01E-06 |
| 22 | 3.52E-09 | 9.30E-07 | 1.71E-06 | 2.36E-06 | 2.90E-06 | 3.74E-06 | 4.42E-06 | 5.33E-06 | 5.11E-06 |
| 23 | -3.76E-09 | 8.26E-07 | 1.58E-06 | 2.23E-06 | 2.78E-06 | 3.64E-06 | 4.37E-06 | 5.28E-06 | 5.20E-06 |
| 24 | -1.03E-08 | 7.14E-07 | 1.43E-06 | 2.09E-06 | 2.65E-06 | 3.53E-06 | 4.30E-06 | 5.27E-06 | 5.24E-06 |
| 25 | -9.07E-09 | 6.27E-07 | 1.31E-06 | 1.96E-06 | 2.53E-06 | 3.42E-06 | 4.22E-06 | 5.20E-06 | 5.27E-06 |
| 26 | -9.42E-09 | 5.41E-07 | 1.18E-06 | 1.82E-06 | 2.40E-06 | 3.32E-06 | 4.13E-06 | 5.14E-06 | 5.26E-06 |
| 27 | -4.43E-09 | 4.05E-07 | 1.10E-06 | 1.73E-06 | 2.28E-06 | 3.19E-06 | 4.01E-06 | 5.07E-06 | 5.25E-06 |
| 28 | -2.35E-08 | 3.18E-07 | 9.84E-07 | 1.64E-06 | 2.16E-06 | 3.08E-06 | 3.97E-06 | 4.94E-06 | 5.21E-06 |
| 29 | -2.18E-09 | 1.78E-07 | 9.02E-07 | 1.51E-06 | 2.04E-06 | 2.98E-06 | 3.79E-06 | 4.86E-06 | 5.13E-06 |
| 30 | -1.08E-07 | 2.54E-07 | 7.84E-07 | 1.36E-06 | 1.92E-06 | 2.93E-06 | 3.74E-06 | 4.62E-06 | 5.08E-06 |
| 31 | -4.51E-08 | 2.97E-07 | 7.61E-07 | 1.30E-06 | 1.84E-06 | 2.73E-06 | 3.48E-06 | 4.60E-06 | 5.13E-06 |
| 32 | 4.95E-09 | 2.47E-09 | 6.45E-07 | 1.22E-06 | 1.72E-06 | 2.64E-06 | 3.47E-06 | 4.47E-06 | 4.94E-06 |
| 33 | 1.11E-08 | -7.16E-09 | 5.62E-07 | 1.14E-06 | 1.62E-06 | 2.50E-06 | 3.32E-06 | 4.33E-06 | 4.85E-06 |
| 34 | 5.33E-09 | 3.53E-09 | 4.81E-07 | 1.06E-06 | 1.53E-06 | 2.40E-06 | 3.24E-06 | 4.19E-06 | 4.75E-06 |
| 35 | -1.41E-08 | 2.68E-08 | 3.80E-07 | 9.69E-07 | 1.43E-06 | 2.29E-06 | 3.13E-06 | 4.11E-06 | 4.66E-06 |
| 36 | -2.09E-09 | 6.72E-08 | 3.35E-07 | 8.99E-07 | 1.37E-06 | 2.17E-06 | 3.01E-06 | 4.01E-06 | 4.61E-06 |
| 37 | -8.34E-08 | 1.21E-07 | 4.00E-07 | 7.70E-07 | 1.22E-06 | 2.15E-06 | 2.86E-06 | 3.86E-06 | 4.42E-06 |
| 38 | 3.30E-09 | -1.47E-09 | 1.89E-07 | 7.62E-07 | 1.21E-06 | 1.99E-06 | 2.78E-06 | 3.76E-06 | 4.33E-06 |
| 39 | 1.76E-10 | -1.35E-09 | 1.16E-07 | 6.81E-07 | 1.13E-06 | 1.89E-06 | 2.67E-06 | 3.66E-06 | 4.20E-06 |
| 40 | 7.34E-10 | -1.81E-09 | 5.67E-08 | 6.02E-07 | 1.05E-06 | 1.82E-06 | 2.57E-06 | 3.55E-06 | 4.10E-06 |
| 41 | -2.48E-09 | 2.51E-09 | 9.16E-10 | 5.47E-07 | 9.87E-07 | 1.75E-06 | 2.46E-06 | 3.45E-06 | 3.95E-06 |
| 42 | 5.03E-09 | -1.92E-09 | -6.80E-09 | 4.71E-07 | 9.20E-07 | 1.67E-06 | 2.36E-06 | 3.33E-06 | 3.89E-06 |
| 43 | 1.13E-08 | -1.99E-09 | 4.65E-09 | 4.15E-07 | 8.77E-07 | 1.62E-06 | 2.31E-06 | 3.24E-06 | 3.78E-06 |
| 44 | -3.36E-09 | -3.17E-09 | 1.60E-09 | 3.72E-07 | 7.96E-07 | 1.55E-06 | 2.22E-06 | 3.14E-06 | 3.67E-06 |
| 45 | 4.30E-09 | 1.49E-09 | 1.91E-09 | 3.12E-07 | 7.46E-07 | 1.47E-06 | 2.14E-06 | 3.03E-06 | 3.58E-06 |
| 46 | 5.07E-09 | -1.84E-09 | -4.67E-09 | 2.54E-07 | 6.85E-07 | 1.40E-06 | 2.06E-06 | 2.96E-06 | 3.48E-06 |
| 47 | 2.29E-08 | 5.10E-09 | 2.46E-09 | 1.99E-07 | 6.29E-07 | 1.33E-06 | 1.99E-06 | 2.86E-06 | 3.39E-06 |
| 48 | 2.77E-09 | -6.88E-10 | -1.96E-09 | 1.46E-07 | 5.81E-07 | 1.28E-06 | 1.92E-06 | 2.77E-06 | 3.31E-06 |
| 49 | 2.41E-09 | 1.09E-08 | -2.39E-08 | 1.15E-07 | 5.18E-07 | 1.23E-06 | 1.85E-06 | 2.70E-06 | 3.23E-06 |
| 50 | -1.81E-10 | -4.24E-10 | -3.17E-10 | 3.85E-08 | 4.84E-07 | 1.16E-06 | 1.78E-06 | 2.60E-06 | 3.13E-06 |
| 51 | -1.95E-09 | 3.67E-08 | -2.30E-08 | 2.62E-08 | 4.36E-07 | 1.16E-06 | 1.70E-06 | 2.54E-06 | 3.05E-06 |
| 52 | -2.08E-08 | -1.89E-08 | -7.46E-09 | 6.06E-08 | 3.46E-07 | 1.04E-06 | 1.64E-06 | 2.45E-06 | 2.96E-06 |
| 53 | 6.88E-09 | -2.68E-08 | -1.89E-08 | 7.93E-08 | 3.03E-07 | 1.02E-06 | 1.61E-06 | 2.39E-06 | 2.68E-06 |
| 54 | 3.67E-09 | 1.59E-08 | -9.68E-09 | 4.47E-08 | 2.67E-07 | 9.45E-07 | 1.55E-06 | 2.34E-06 | 2.82E-06 |
| 55 | -5.40E-11 | 4.07E-09 | -5.30E-09 | 1.05E-08 | 2.43E-07 | 8.88E-07 | 1.49E-06 | 2.27E-06 | 2.76E-06 |
| 56 | -4.84E-08 | 2.38E-09 | -4.36E-08 | -5.26E-09 | 1.82E-07 | 7.69E-07 | 1.39E-06 | 2.21E-06 | 2.69E-06 |
| 57 | -1.02E-09 | 8.60E-09 | -4.55E-09 | 6.34E-09 | 1.54E-07 | 7.96E-07 | 1.38E-06 | 2.13E-06 | 2.64E-06 |
| 58 | 1.41E-09 | 1.24E-08 | -1.08E-08 | -1.14E-08 | 1.21E-07 | 7.43E-07 | 1.30E-06 | 2.02E-06 | 2.56E-06 |
| 59 | -4.46E-09 | -3.28E-09 | -3.43E-10 | -1.05E-09 | 7.43E-08 | 8.70E-07 | 2.29E-06 | 4.94E-06 | 6.68E-06 |
| 60 | -3.88E-09 | 2.03E-08 | 1.19E-08 | -1.55E-08 | 4.93E-08 | 9.68E-07 | 3.03E-06 | 7.25E-06 | 1.01E-05 |
| 61 | -3.35E-09 | 6.67E-09 | 3.29E-09 | -2.24E-08 | 1.17E-08 | 6.01E-07 | 1.12E-06 | 1.91E-06 | 2.40E-06 |
| 62 | 1.75E-09 | -1.94E-09 | 2.55E-09 | -1.40E-09 | 1.39E-10 | 6.06E-07 | 1.21E-06 | 1.98E-06 | 2.62E-06 |
| 63 | -1.57E-09 | -2.29E-10 | 3.65E-10 | -4.48E-09 | -8.48E-10 | 6.03E-07 | 1.06E-06 | 1.65E-06 | 2.13E-06 |
| 64 | -1.44E-09 | 1.92E-10 | 1.35E-09 | -5.32E-10 | -1.38E-09 | 5.24E-07 | 1.04E-06 | 1.58E-06 | 2.07E-06 |
| 65 | 1.48E-11 | 1.62E-10 | 3.81E-10 | 1.35E-10 | 1.21E-10 | 4.88E-07 | 9.93E-07 | 1.58E-06 | 2.05E-06 |
| 66 | -2.76E-08 | 1.18E-08 | -1.80E-09 | -7.85E-09 | 5.90E-08 | 4.44E-07 | 9.58E-07 | 1.57E-06 | 1.99E-06 |
| 67 | -7.58E-10 | 1.56E-10 | 9.49E-10 | 4.75E-10 | -6.97E-10 | 4.35E-07 | 8.60E-07 | 1.50E-06 | 1.98E-06 |
| 68 | -9.59E-10 | -8.25E-10 | -2.34E-10 | 1.48E-09 | 7.87E-09 | 4.94E-07 | 1.53E-06 | 3.30E-06 | 4.68E-06 |
| 69 | -6.81E-11 | 2.20E-09 | 2.67E-09 | -3.27E-09 | 1.43E-10 | 3.44E-07 | 8.14E-07 | 1.46E-06 | 1.78E-06 |
| 70 | -5.25E-09 | -1.66E-09 | 2.03E-09 | 7.15E-10 | 5.17E-10 | 3.35E-07 | 9.59E-07 | 1.87E-06 | 2.49E-06 |
| 71 | -6.80E-10 | 2.07E-10 | -8.32E-11 | -9.70E-10 | 8.89E-10 | 2.81E-07 | 7.54E-07 | 1.26E-06 | 1.49E-06 |
| 72 | -3.37E-11 | -4.68E-10 | -4.69E-10 | -4.42E-10 | -4.19E-10 | 2.54E-07 | 6.71E-07 | 1.23E-06 | 1.50E-06 |
| 73 | -1.51E-09 | 4.32E-09 | 3.08E-09 | -4.69E-09 | 4.97E-09 | 2.20E-07 | 6.38E-07 | 1.18E-06 | 1.46E-06 |
| 74 | -2.74E-09 | -6.33E-10 | 2.78E-10 | -3.98E-09 | 1.26E-09 | 1.89E-07 | 6.10E-07 | 1.15E-06 | 1.42E-06 |
| 75 | -1.14E-09 | 3.05E-10 | 4.11E-10 | -1.98E-09 | -3.52E-10 | 1.58E-07 | 5.84E-07 | 1.12E-06 | 1.38E-06 |
| 76 | -9.86E-12 | -3.55E-11 | 2.70E-10 | -9.36E-10 | -8.59E-10 | 1.28E-07 | 5.56E-07 | 1.09E-06 | 1.35E-06 |
| 77 | -3.86E-10 | 3.56E-10 | 6.29E-10 | -2.25E-10 | 4.74E-10 | 1.01E-07 | 5.35E-07 | 1.05E-06 | 1.31E-06 |
| 78 | 1.64E-09 | 2.15E-09 | 2.19E-09 | 1.39E-09 | 1.28E-09 | 7.57E-08 | 5.17E-07 | 1.02E-06 | 1.29E-06 |
| 79 | 2.22E-10 | 1.31E-10 | -2.36E-12 | -4.97E-11 | 7.77E-10 | 4.64E-08 | 4.93E-07 | 9.89E-07 | 1.26E-06 |
| 80 | -7.20E-10 | -1.19E-09 | 8.65E-11 | 2.00E-09 | -2.07E-09 | 1.58E-08 | 4.69E-07 | 9.53E-07 | 1.24E-06 |
| 81 | -6.31E-10 | 2.48E-10 | 5.63E-10 | 1.98E-10 | -4.11E-10 | 1.10E-10 | 4.43E-07 | 9.14E-07 | 1.21E-06 |
| 82 | 3.94E-10 | 2.84E-10 | 1.05E-10 | -2.03E-10 | -7.65E-10 | 1.14E-09 | 4.17E-07 | 8.84E-07 | 1.18E-06 |
| 83 | -1.20E-10 | 1.90E-11 | 1.31E-10 | 1.40E-10 | -2.31E-10 | -1.72E-10 | 3.92E-07 | 8.53E-07 | 1.15E-06 |
| 84 | 3.11E-08 | 2.25E-08 | 1.42E-08 | 9.56E-09 | 1.41E-08 | 8.13E-08 | 2.84E-07 | 8.25E-07 | 1.11E-06 |
| 85 | 7.00E-10 | 2.82E-10 | -1.17E-10 | -4.57E-10 | -6.82E-10 | 5.96E-10 | 3.43E-07 | 7.96E-07 | 1.10E-06 |
| 86 | 1.81E-08 | 8.44E-09 | -6.44E-09 | -1.41E-08 | -7.84E-09 | 6.64E-08 | 2.71E-07 | 7.97E-07 | 1.07E-06 |
| 87 | -1.09E-10 | -1.95E-11 | 1.38E-10 | 3.59E-10 | 2.51E-10 | -3.76E-11 | 2.95E-07 | 7.53E-07 | 1.05E-06 |
| 88 | -1.38E-09 | 1.46E-09 | -1.94E-09 | 1.97E-09 | -2.64E-10 | 1.08E-09 | 2.66E-07 | 7.32E-07 | 1.02E-06 |
| 89 | -6.35E-11 | -1.76E-12 | -2.19E-11 | -2.73E-11 | -9.01E-11 | -1.27E-10 | 2.45E-07 | 7.13E-07 | 9.99E-07 |
| 90 | 1.47E-10 | -1.08E-10 | -5.80E-11 | 2.39E-10 | 4.46E-10 | 1.34E-10 | 2.20E-07 | 6.92E-07 | 9.75E-07 |
| 91 | 1.37E-09 | 6.28E-10 | -2.38E-10 | -1.30E-09 | -2.43E-09 | 2.65E-09 | 1.96E-07 | 6.73E-07 | 9.53E-07 |
| 92 | -6.28E-11 | 1.28E-11 | 4.66E-11 | 3.44E-11 | -3.01E-11 | -1.23E-10 | 1.76E-07 | 6.50E-07 | 9.28E-07 |
| 93 | 2.81E-11 | -4.64E-11 | -1.07E-10 | 7.01E-14 | 3.63E-11 | 3.24E-10 | 1.57E-07 | 6.29E-07 | 9.03E-07 |
| 94 | -2.97E-10 | -1.02E-10 | 2.24E-10 | 4.68E-10 | -2.68E-10 | -2.71E-10 | 1.38E-07 | 6.06E-07 | 8.75E-07 |
| 95 | -8.54E-12 | 8.20E-11 | -1.18E-10 | -1.05E-11 | -7.05E-11 | 1.08E-10 | 1.17E-07 | 5.83E-07 | 8.46E-07 |
| 96 | 1.82E-10 | -1.52E-09 | 6.58E-10 | 1.69E-09 | -1.81E-09 | 1.75E-09 | 9.78E-08 | 5.60E-07 | 8.17E-07 |
| 97 | 1.58E-11 | 1.97E-10 | 3.37E-10 | 2.17E-10 | -6.62E-10 | -1.95E-10 | 7.43E-08 | 5.33E-07 | 7.90E-07 |
| 98 | -8.21E-11 | 2.05E-11 | 1.16E-10 | 1.11E-10 | -1.90E-10 | -3.56E-10 | 5.28E-08 | 5.11E-07 | 7.67E-07 |
| 99 | 5.71E-11 | 5.21E-11 | 3.51E-11 | -8.85E-12 | -1.01E-10 | 2.08E-10 | 3.09E-08 | 4.90E-07 | 7.47E-07 |
| 100 | -1.33E-11 | -3.87E-11 | 6.77E-13 | 4.57E-11 | -3.82E-11 | 1.66E-11 | 1.23E-08 | 4.73E-07 | 7.29E-07 |
| 101 | 3.87E-10 | 1.69E-10 | 1.90E-11 | -2.00E-10 | -4.80E-10 | -6.35E-10 | 1.54E-09 | 4.59E-07 | 7.13E-07 |
| 102 | -2.13E-12 | -4.86E-12 | -4.55E-12 | 2.47E-12 | 2.27E-11 | 3.97E-12 | -1.08E-11 | 4.46E-07 | 6.94E-07 |
| 103 | -1.11E-11 | -1.36E-11 | -1.70E-11 | -2.28E-11 | -3.42E-11 | -1.18E-10 | 8.92E-11 | 4.25E-07 | 6.77E-07 |
| 104 | -4.09E-11 | 2.19E-11 | 3.54E-11 | 1.62E-11 | -2.18E-11 | -9.79E-11 | 6.08E-11 | 4.08E-07 | 6.57E-07 |
| 105 | 5.81E-11 | 2.23E-11 | 1.92E-11 | 3.01E-11 | 3.67E-11 | -7.88E-11 | 3.28E-11 | 3.88E-07 | 6.34E-07 |
| 106 | 4.66E-11 | 6.38E-11 | 9.14E-11 | 1.05E-10 | 9.34E-11 | -1.57E-11 | 8.75E-11 | 3.67E-07 | 6.17E-07 |
| 107 | -1.93E-11 | -2.46E-11 | -1.38E-11 | 7.68E-12 | 2.81E-11 | -3.99E-12 | 7.34E-11 | 3.47E-07 | 5.99E-07 |
| 108 | 3.05E-11 | 2.41E-11 | 1.39E-11 | -1.25E-12 | -2.21E-11 | -7.84E-11 | 7.42E-11 | 3.33E-07 | 5.83E-07 |
| 109 | 5.49E-11 | 3.03E-11 | 2.91E-11 | 3.93E-11 | 2.95E-11 | -1.88E-10 | -7.67E-11 | 3.17E-07 | 5.68E-07 |
| 110 | -7.34E-12 | -8.62E-12 | -7.79E-12 | -2.57E-12 | 9.37E-12 | -8.57E-12 | -1.24E-10 | 3.05E-07 | 5.54E-07 |
| 111 | 1.45E-11 | 2.75E-11 | 4.98E-11 | 8.15E-11 | 1.13E-10 | 9.22E-11 | 1.25E-10 | 2.91E-07 | 5.40E-07 |
| 112 | -2.15E-11 | 3.71E-12 | 2.62E-11 | 4.27E-11 | 4.86E-11 | 5.09E-12 | 7.30E-11 | 2.76E-07 | 5.25E-07 |
| 113 | -2.11E-10 | 2.22E-10 | 6.15E-10 | 8.82E-10 | 8.97E-10 | -2.69E-10 | 4.40E-10 | 2.54E-07 | 5.03E-07 |
| 114 | 1.30E-10 | 1.27E-10 | 1.16E-10 | 1.18E-10 | 1.65E-10 | 1.76E-10 | 2.19E-10 | 2.35E-07 | 4.87E-07 |
| 115 | 9.96E-11 | -1.40E-10 | -1.17E-10 | 1.74E-11 | 1.45E-10 | 1.77E-10 | -1.85E-10 | 2.13E-07 | 4.67E-07 |
| 116 | -7.96E-12 | -2.16E-11 | -3.97E-11 | -4.16E-11 | -1.86E-11 | 3.33E-11 | 5.31E-11 | 1.93E-07 | 4.46E-07 |
| 117 | 8.43E-11 | -1.47E-10 | -1.85E-10 | 7.25E-11 | 4.55E-10 | -8.33E-10 | -2.37E-10 | 1.77E-07 | 4.25E-07 |
| 118 | -5.80E-12 | 3.31E-12 | 2.82E-11 | 5.98E-11 | 6.77E-11 | -1.23E-10 | 1.43E-12 | 1.61E-07 | 4.07E-07 |
| 119 | -6.17E-11 | -3.42E-11 | -1.22E-12 | 3.37E-11 | 6.13E-11 | -3.63E-11 | 1.91E-11 | 1.49E-07 | 3.91E-07 |
| 120 | 9.94E-12 | 8.53E-12 | 6.08E-12 | 1.53E-12 | -7.32E-12 | -6.20E-11 | -8.48E-11 | 1.37E-07 | 3.74E-07 |
| 121 | 1.42E-10 | 8.63E-11 | 2.08E-12 | -3.00E-11 | 8.00E-11 | -6.43E-11 | -1.66E-10 | 1.25E-07 | 3.61E-07 |
| 122 | -1.07E-10 | -4.72E-11 | -4.10E-12 | 4.97E-11 | 1.98E-10 | 9.65E-11 | 5.72E-11 | 1.13E-07 | 3.48E-07 |
| 123 | 8.80E-12 | 1.52E-11 | 2.40E-11 | 3.26E-11 | 3.23E-11 | -1.22E-10 | 4.91E-11 | 9.87E-08 | 3.36E-07 |
| 124 | -7.75E-11 | -1.87E-11 | 2.62E-11 | 4.61E-11 | 3.67E-11 | -9.78E-11 | 9.34E-11 | 8.45E-08 | 3.23E-07 |
| 125 | 1.06E-10 | 4.12E-11 | -9.08E-12 | -4.79E-11 | -8.11E-11 | -1.73E-10 | 9.18E-11 | 7.05E-08 | 3.10E-07 |
| 126 | -1.36E-11 | -6.26E-12 | 9.32E-12 | 3.77E-11 | 7.89E-11 | 5.02E-11 | 9.10E-11 | 5.64E-08 | 2.96E-07 |
| 127 | 7.45E-12 | 3.09E-12 | 3.40E-12 | 1.10E-11 | 2.70E-11 | 3.51E-11 | 7.95E-11 | 4.35E-08 | 2.82E-07 |
| 128 | 7.37E-11 | -9.64E-13 | -2.44E-11 | -1.25E-11 | 1.06E-11 | -5.15E-11 | -4.08E-11 | 3.12E-08 | 2.66E-07 |
| 129 | -2.39E-13 | 5.13E-14 | -1.51E-12 | -5.68E-12 | -1.24E-11 | -2.71E-11 | 1.07E-10 | 1.98E-08 | 2.51E-07 |
| 130 | 8.68E-13 | 9.04E-13 | 1.51E-12 | 2.85E-12 | 3.02E-12 | -3.49E-11 | -1.47E-10 | 1.07E-08 | 2.35E-07 |
| 131 | 3.25E-10 | 3.20E-10 | 3.08E-10 | 2.78E-10 | 2.05E-10 | -4.33E-10 | -2.84E-10 | 4.67E-10 | 2.20E-07 |
| 132 | 4.07E-11 | 1.76E-11 | -5.39E-12 | -2.81E-11 | -5.07E-11 | -9.42E-11 | -1.37E-10 | 6.36E-11 | 2.06E-07 |
| 133 | -2.55E-12 | -3.73E-12 | -1.34E-12 | 2.29E-12 | 4.46E-12 | 3.81E-12 | -8.45E-12 | 5.98E-12 | 1.93E-07 |
| 134 | 8.16E-13 | 1.06E-12 | 1.17E-12 | 1.07E-12 | 8.18E-13 | 1.90E-12 | -8.77E-13 | -1.11E-12 | 1.81E-07 |
| 135 | 4.10E-11 | 3.15E-11 | 1.70E-11 | -3.61E-12 | -3.04E-11 | -9.02E-11 | -2.71E-10 | -9.72E-11 | 1.69E-07 |
| 136 | 3.50E-12 | -3.58E-12 | -1.18E-11 | -1.88E-11 | -2.22E-11 | -4.35E-11 | -1.59E-10 | -4.55E-11 | 1.57E-07 |
| 137 | -5.28E-11 | -4.17E-11 | -2.53E-11 | -5.89E-12 | 1.13E-11 | 1.82E-13 | -1.59E-10 | -2.24E-10 | 1.45E-07 |
| 138 | -3.04E-12 | -2.34E-12 | -3.28E-12 | -5.50E-12 | -8.74E-12 | -1.78E-11 | -4.75E-11 | -1.50E-10 | 1.33E-07 |
| 139 | 5.91E-12 | -4.01E-11 | -3.99E-11 | -2.34E-11 | -5.54E-12 | 1.16E-11 | -6.25E-12 | -2.63E-11 | 1.21E-07 |
| 140 | -6.95E-13 | -8.27E-13 | -9.01E-13 | -9.45E-13 | -9.76E-13 | -1.81E-12 | -7.07E-12 | -8.80E-11 | 1.10E-07 |
| 141 | 2.52E-12 | 1.03E-12 | -2.38E-13 | -1.18E-12 | -1.57E-12 | 1.10E-12 | 3.85E-12 | -1.87E-14 | 9.85E-08 |
| 142 | 2.07E-13 | -2.28E-14 | -7.07E-13 | -1.73E-12 | -2.37E-12 | 5.72E-13 | -1.58E-12 | -3.37E-12 | 8.79E-08 |
| 143 | 5.79E-13 | -2.78E-13 | -1.04E-12 | -1.76E-12 | -2.55E-12 | -5.39E-12 | -1.64E-11 | 4.79E-11 | 7.79E-08 |
| 144 | -2.10E-12 | -6.74E-12 | -9.35E-12 | -7.30E-12 | -1.26E-12 | 1.32E-11 | -5.24E-12 | -2.80E-12 | 6.81E-08 |
| 145 | -1.03E-11 | -5.58E-12 | -2.08E-12 | 3.40E-13 | 1.86E-12 | 2.38E-12 | -3.75E-12 | 6.63E-13 | 5.83E-08 |
| 146 | -6.23E-12 | -3.89E-12 | -1.54E-12 | 7.54E-13 | 2.95E-12 | 6.85E-12 | 3.02E-12 | 8.87E-12 | 4.84E-08 |
| 147 | -3.43E-11 | -2.73E-11 | -1.67E-11 | -2.59E-12 | 1.31E-11 | 3.36E-11 | -2.34E-11 | 3.39E-11 | 3.80E-08 |
| 148 | 9.27E-14 | 4.58E-14 | 1.73E-14 | 1.25E-14 | 4.13E-14 | 1.73E-13 | -1.98E-12 | -1.92E-12 | 2.70E-08 |
| 149 | 3.50E-12 | 2.99E-12 | 3.18E-12 | 3.94E-12 | 4.98E-12 | 6.21E-12 | 5.41E-12 | 2.23E-11 | 1.59E-08 |
| 150 | -2.29E-13 | -3.03E-13 | -2.86E-13 | -2.21E-13 | -1.36E-13 | 1.20E-13 | -7.63E-15 | -1.37E-12 | 3.76E-09 |

Table.20. Spectra predicted by MLP neural network: Al- 1.2mm

| energy bin (kev)/voltage(kv) | 20kv | 30kv | 40kv | 50kv | 60kv | 80kv | 100kv | 130kv | 150kv |
| --- | --- | --- | --- | --- | --- | --- | --- | --- | --- |
| 1 | 0 | 0 | 0 | 0 | 0 | 0 | 0 | 0 | 0 |
| 2 | -1.95E-14 | -1.64E-14 | -1.11E-14 | -6.20E-15 | -8.76E-15 | -5.32E-14 | 2.46E-14 | 2.64E-13 | 3.72E-14 |
| 3 | 2.20E-10 | 2.26E-10 | 2.24E-10 | 2.12E-10 | 1.89E-10 | 1.37E-10 | 1.95E-10 | -6.03E-11 | 2.38E-10 |
| 4 | 1.37E-07 | 1.16E-07 | 9.64E-08 | 8.14E-08 | 7.09E-08 | 6.00E-08 | 6.01E-08 | 8.48E-08 | 9.79E-08 |
| 5 | 7.81E-07 | 8.66E-07 | 8.82E-07 | 8.38E-07 | 7.74E-07 | 6.99E-07 | 6.67E-07 | 6.73E-07 | 6.52E-07 |
| 6 | 1.34E-06 | 2.04E-06 | 2.29E-06 | 2.27E-06 | 2.16E-06 | 1.98E-06 | 1.92E-06 | 1.83E-06 | 1.65E-06 |
| 7 | 1.89E-06 | 2.85E-06 | 3.45E-06 | 3.54E-06 | 3.46E-06 | 3.26E-06 | 3.24E-06 | 3.34E-06 | 2.83E-06 |
| 8 | 2.29E-06 | 3.43E-06 | 4.24E-06 | 4.74E-06 | 4.99E-06 | 5.03E-06 | 4.88E-06 | 4.64E-06 | 4.28E-06 |
| 9 | 2.22E-06 | 4.52E-06 | 6.24E-06 | 7.61E-06 | 8.74E-06 | 1.02E-05 | 1.10E-05 | 1.28E-05 | 1.13E-05 |
| 10 | 2.06E-06 | 3.42E-06 | 4.51E-06 | 5.38E-06 | 6.10E-06 | 7.24E-06 | 8.04E-06 | 8.57E-06 | 7.74E-06 |
| 11 | 1.81E-06 | 2.79E-06 | 3.59E-06 | 4.14E-06 | 4.45E-06 | 4.71E-06 | 4.80E-06 | 5.15E-06 | 4.43E-06 |
| 12 | 1.49E-06 | 2.49E-06 | 3.18E-06 | 3.61E-06 | 3.85E-06 | 3.99E-06 | 4.01E-06 | 4.23E-06 | 3.48E-06 |
| 13 | 1.30E-06 | 2.18E-06 | 2.82E-06 | 3.18E-06 | 3.35E-06 | 3.40E-06 | 3.38E-06 | 3.43E-06 | 2.94E-06 |
| 14 | 1.13E-06 | 1.98E-06 | 2.61E-06 | 3.05E-06 | 3.34E-06 | 3.65E-06 | 3.74E-06 | 3.59E-06 | 3.33E-06 |
| 15 | 1.04E-06 | 1.86E-06 | 2.58E-06 | 3.10E-06 | 3.40E-06 | 3.73E-06 | 3.91E-06 | 4.13E-06 | 3.60E-06 |
| 16 | 7.50E-07 | 1.75E-06 | 2.50E-06 | 3.00E-06 | 3.33E-06 | 3.83E-06 | 4.23E-06 | 4.35E-06 | 3.90E-06 |
| 17 | 5.83E-07 | 1.60E-06 | 2.36E-06 | 2.92E-06 | 3.34E-06 | 3.87E-06 | 4.17E-06 | 4.68E-06 | 4.18E-06 |
| 18 | 4.46E-07 | 1.45E-06 | 2.23E-06 | 2.83E-06 | 3.27E-06 | 3.87E-06 | 4.29E-06 | 4.86E-06 | 4.42E-06 |
| 19 | 3.10E-07 | 1.32E-06 | 2.10E-06 | 2.71E-06 | 3.20E-06 | 3.89E-06 | 4.38E-06 | 5.06E-06 | 4.63E-06 |
| 20 | 1.09E-07 | 1.16E-06 | 1.96E-06 | 2.56E-06 | 3.03E-06 | 3.77E-06 | 4.39E-06 | 5.14E-06 | 4.81E-06 |
| 21 | 1.02E-08 | 1.02E-06 | 1.82E-06 | 2.45E-06 | 2.97E-06 | 3.76E-06 | 4.39E-06 | 5.18E-06 | 4.93E-06 |
| 22 | 8.66E-09 | 9.13E-07 | 1.68E-06 | 2.32E-06 | 2.85E-06 | 3.68E-06 | 4.35E-06 | 5.23E-06 | 5.02E-06 |
| 23 | 5.87E-10 | 8.11E-07 | 1.54E-06 | 2.17E-06 | 2.72E-06 | 3.60E-06 | 4.31E-06 | 5.14E-06 | 5.09E-06 |
| 24 | -1.19E-08 | 7.05E-07 | 1.41E-06 | 2.05E-06 | 2.61E-06 | 3.48E-06 | 4.23E-06 | 5.19E-06 | 5.16E-06 |
| 25 | -7.07E-09 | 6.14E-07 | 1.29E-06 | 1.93E-06 | 2.50E-06 | 3.37E-06 | 4.15E-06 | 5.12E-06 | 5.17E-06 |
| 26 | -3.42E-09 | 5.52E-07 | 1.18E-06 | 1.80E-06 | 2.36E-06 | 3.25E-06 | 4.05E-06 | 5.07E-06 | 5.18E-06 |
| 27 | -3.32E-09 | 4.01E-07 | 1.09E-06 | 1.72E-06 | 2.25E-06 | 3.14E-06 | 3.95E-06 | 5.01E-06 | 5.17E-06 |
| 28 | 2.47E-08 | 2.97E-07 | 9.20E-07 | 1.58E-06 | 2.13E-06 | 3.04E-06 | 3.87E-06 | 4.92E-06 | 5.11E-06 |
| 29 | -3.63E-09 | 1.78E-07 | 8.96E-07 | 1.49E-06 | 2.01E-06 | 2.94E-06 | 3.74E-06 | 4.80E-06 | 5.06E-06 |
| 30 | -5.91E-08 | 2.64E-07 | 7.38E-07 | 1.28E-06 | 1.82E-06 | 2.85E-06 | 3.72E-06 | 4.63E-06 | 5.06E-06 |
| 31 | -7.76E-08 | 2.51E-07 | 7.04E-07 | 1.25E-06 | 1.81E-06 | 2.73E-06 | 3.46E-06 | 4.52E-06 | 5.13E-06 |
| 32 | 5.90E-09 | 5.48E-09 | 6.41E-07 | 1.21E-06 | 1.70E-06 | 2.61E-06 | 3.43E-06 | 4.41E-06 | 4.87E-06 |
| 33 | -5.33E-09 | 2.22E-09 | 5.63E-07 | 1.12E-06 | 1.60E-06 | 2.49E-06 | 3.31E-06 | 4.29E-06 | 4.79E-06 |
| 34 | 2.85E-09 | 4.00E-09 | 4.75E-07 | 1.04E-06 | 1.51E-06 | 2.37E-06 | 3.20E-06 | 4.15E-06 | 4.69E-06 |
| 35 | -5.68E-10 | 4.59E-08 | 4.01E-07 | 9.77E-07 | 1.42E-06 | 2.26E-06 | 3.08E-06 | 4.05E-06 | 4.61E-06 |
| 36 | -2.59E-08 | 4.47E-08 | 3.16E-07 | 8.82E-07 | 1.35E-06 | 2.14E-06 | 2.97E-06 | 3.92E-06 | 4.49E-06 |
| 37 | -6.21E-08 | 1.14E-07 | 3.73E-07 | 7.28E-07 | 1.17E-06 | 2.11E-06 | 2.83E-06 | 3.81E-06 | 4.41E-06 |
| 38 | 9.08E-10 | 3.07E-10 | 1.84E-07 | 7.45E-07 | 1.19E-06 | 1.95E-06 | 2.75E-06 | 3.72E-06 | 4.26E-06 |
| 39 | 1.04E-09 | 2.87E-10 | 1.15E-07 | 6.71E-07 | 1.12E-06 | 1.87E-06 | 2.64E-06 | 3.61E-06 | 4.19E-06 |
| 40 | 1.32E-09 | -1.90E-09 | 5.55E-08 | 6.00E-07 | 1.04E-06 | 1.80E-06 | 2.54E-06 | 3.51E-06 | 4.03E-06 |
| 41 | -2.64E-09 | 1.68E-09 | -2.48E-09 | 5.42E-07 | 9.73E-07 | 1.73E-06 | 2.43E-06 | 3.41E-06 | 3.93E-06 |
| 42 | 4.94E-09 | -6.26E-10 | -4.34E-10 | 4.68E-07 | 9.11E-07 | 1.66E-06 | 2.36E-06 | 3.30E-06 | 3.83E-06 |
| 43 | 8.06E-09 | 1.90E-09 | 1.04E-08 | 4.14E-07 | 8.69E-07 | 1.60E-06 | 2.28E-06 | 3.19E-06 | 3.72E-06 |
| 44 | 6.76E-10 | 1.28E-10 | 8.48E-10 | 3.68E-07 | 7.88E-07 | 1.53E-06 | 2.18E-06 | 3.10E-06 | 3.63E-06 |
| 45 | -3.85E-10 | 1.20E-09 | 2.68E-09 | 3.09E-07 | 7.38E-07 | 1.45E-06 | 2.11E-06 | 3.00E-06 | 3.53E-06 |
| 46 | -4.22E-09 | -7.58E-09 | -6.31E-09 | 2.51E-07 | 6.75E-07 | 1.38E-06 | 2.05E-06 | 2.93E-06 | 3.43E-06 |
| 47 | 1.31E-08 | 7.38E-10 | 4.64E-09 | 2.01E-07 | 6.28E-07 | 1.32E-06 | 1.98E-06 | 2.84E-06 | 3.35E-06 |
| 48 | 2.67E-09 | 1.17E-09 | 5.98E-10 | 1.45E-07 | 5.72E-07 | 1.26E-06 | 1.89E-06 | 2.74E-06 | 3.27E-06 |
| 49 | 7.46E-10 | 1.11E-08 | -2.18E-08 | 1.13E-07 | 5.05E-07 | 1.21E-06 | 1.82E-06 | 2.67E-06 | 3.21E-06 |
| 50 | -3.23E-10 | -3.78E-10 | -5.24E-10 | 3.78E-08 | 4.77E-07 | 1.15E-06 | 1.76E-06 | 2.57E-06 | 3.10E-06 |
| 51 | -8.73E-09 | 2.97E-08 | -2.57E-08 | 2.68E-08 | 4.31E-07 | 1.14E-06 | 1.69E-06 | 2.51E-06 | 3.01E-06 |
| 52 | -2.06E-08 | -1.91E-08 | -9.60E-09 | 4.77E-08 | 3.04E-07 | 1.03E-06 | 1.62E-06 | 2.42E-06 | 2.92E-06 |
| 53 | 5.74E-09 | -2.75E-08 | -2.19E-08 | 7.03E-08 | 2.85E-07 | 9.97E-07 | 1.59E-06 | 2.35E-06 | 2.68E-06 |
| 54 | 4.67E-09 | 1.51E-08 | -1.00E-08 | 4.80E-08 | 2.71E-07 | 9.34E-07 | 1.53E-06 | 2.31E-06 | 2.80E-06 |
| 55 | 2.60E-09 | 2.49E-09 | -2.66E-09 | 5.85E-09 | 2.33E-07 | 8.78E-07 | 1.48E-06 | 2.24E-06 | 2.73E-06 |
| 56 | -3.39E-08 | 2.25E-08 | -1.79E-08 | 2.66E-08 | 2.21E-07 | 8.14E-07 | 1.41E-06 | 2.19E-06 | 2.65E-06 |
| 57 | -2.66E-09 | 7.19E-09 | -5.60E-09 | 4.35E-09 | 1.49E-07 | 7.84E-07 | 1.36E-06 | 2.10E-06 | 2.61E-06 |
| 58 | 1.12E-08 | 2.11E-08 | -3.87E-09 | -8.82E-09 | 1.18E-07 | 7.35E-07 | 1.29E-06 | 1.99E-06 | 2.53E-06 |
| 59 | 5.63E-10 | -1.48E-09 | -1.67E-10 | -2.87E-09 | 6.98E-08 | 8.55E-07 | 2.26E-06 | 4.88E-06 | 6.61E-06 |
| 60 | -8.07E-09 | 1.58E-08 | 8.15E-09 | -1.93E-08 | 4.27E-08 | 9.50E-07 | 2.99E-06 | 7.17E-06 | 9.98E-06 |
| 61 | -4.30E-09 | 5.98E-09 | 3.60E-09 | -2.06E-08 | 1.31E-08 | 5.97E-07 | 1.11E-06 | 1.88E-06 | 2.39E-06 |
| 62 | 2.97E-10 | -2.62E-09 | 2.07E-09 | -1.93E-09 | -9.96E-10 | 5.99E-07 | 1.20E-06 | 1.95E-06 | 2.60E-06 |
| 63 | -5.32E-11 | 4.49E-10 | 8.83E-10 | -3.13E-09 | 1.54E-09 | 5.97E-07 | 1.04E-06 | 1.64E-06 | 2.11E-06 |
| 64 | -8.79E-10 | 1.78E-10 | 1.40E-09 | 7.93E-10 | 9.40E-10 | 5.19E-07 | 1.03E-06 | 1.56E-06 | 2.05E-06 |
| 65 | -4.48E-10 | -2.51E-10 | -1.14E-11 | 4.61E-11 | 5.24E-10 | 4.81E-07 | 9.80E-07 | 1.56E-06 | 2.02E-06 |
| 66 | -1.46E-08 | 1.70E-08 | -5.37E-09 | -1.86E-08 | 4.37E-08 | 4.26E-07 | 9.43E-07 | 1.56E-06 | 1.99E-06 |
| 67 | -2.90E-10 | -3.95E-10 | -5.48E-10 | -7.11E-10 | -2.57E-10 | 4.35E-07 | 8.57E-07 | 1.49E-06 | 1.96E-06 |
| 68 | 6.10E-10 | 2.51E-10 | 2.38E-10 | 1.11E-09 | 6.22E-09 | 4.89E-07 | 1.52E-06 | 3.26E-06 | 4.63E-06 |
| 69 | -4.46E-10 | 1.75E-09 | 2.40E-09 | -2.85E-09 | 1.19E-09 | 3.41E-07 | 8.06E-07 | 1.44E-06 | 1.77E-06 |
| 70 | -3.73E-09 | 5.23E-10 | 3.81E-09 | 1.48E-09 | -7.98E-11 | 3.29E-07 | 9.48E-07 | 1.85E-06 | 2.47E-06 |
| 71 | 2.22E-10 | 5.94E-10 | 2.58E-10 | -4.69E-10 | 1.41E-09 | 2.80E-07 | 7.48E-07 | 1.25E-06 | 1.47E-06 |
| 72 | -5.08E-10 | -4.74E-10 | -5.49E-11 | 2.00E-10 | 1.78E-10 | 2.54E-07 | 6.66E-07 | 1.22E-06 | 1.48E-06 |
| 73 | -1.51E-09 | 4.51E-09 | 3.36E-09 | -4.52E-09 | 4.55E-09 | 2.17E-07 | 6.32E-07 | 1.17E-06 | 1.45E-06 |
| 74 | -3.73E-09 | -1.55E-09 | -1.74E-10 | -4.28E-09 | -1.18E-10 | 1.85E-07 | 6.05E-07 | 1.14E-06 | 1.41E-06 |
| 75 | -1.17E-09 | 2.18E-10 | 3.81E-10 | -1.71E-09 | 4.49E-10 | 1.56E-07 | 5.79E-07 | 1.10E-06 | 1.37E-06 |
| 76 | 1.50E-10 | 1.26E-11 | 2.24E-10 | -7.85E-10 | -9.58E-11 | 1.28E-07 | 5.50E-07 | 1.08E-06 | 1.34E-06 |
| 77 | -4.52E-10 | 2.44E-10 | 5.32E-10 | -3.09E-10 | 2.38E-10 | 9.99E-08 | 5.29E-07 | 1.04E-06 | 1.30E-06 |
| 78 | 1.02E-09 | 1.39E-09 | 1.32E-09 | 7.15E-10 | 1.24E-09 | 7.41E-08 | 5.12E-07 | 1.01E-06 | 1.28E-06 |
| 79 | 2.09E-10 | 1.04E-10 | -7.33E-11 | -2.12E-10 | 5.39E-10 | 4.57E-08 | 4.88E-07 | 9.79E-07 | 1.25E-06 |
| 80 | 9.54E-10 | -5.07E-10 | -4.96E-10 | 6.95E-10 | -2.72E-09 | 1.53E-08 | 4.62E-07 | 9.43E-07 | 1.22E-06 |
| 81 | -3.24E-10 | 2.90E-10 | 5.47E-10 | 2.19E-10 | -4.89E-10 | 9.97E-11 | 4.39E-07 | 9.05E-07 | 1.20E-06 |
| 82 | 4.16E-10 | 2.85E-10 | 1.10E-10 | -1.85E-10 | -7.53E-10 | 7.78E-10 | 4.12E-07 | 8.75E-07 | 1.17E-06 |
| 83 | -7.73E-11 | 6.45E-11 | 1.79E-10 | 1.99E-10 | -1.25E-10 | -2.62E-11 | 3.87E-07 | 8.43E-07 | 1.14E-06 |
| 84 | 1.34E-08 | 8.43E-09 | 8.37E-09 | 1.57E-08 | 3.35E-08 | 1.21E-07 | 3.25E-07 | 8.28E-07 | 1.08E-06 |
| 85 | 4.59E-10 | 7.47E-11 | -2.51E-10 | -4.78E-10 | -5.69E-10 | 8.34E-10 | 3.40E-07 | 7.88E-07 | 1.09E-06 |
| 86 | 1.17E-08 | 9.91E-09 | -2.82E-09 | -1.51E-08 | -1.46E-08 | 5.57E-08 | 2.62E-07 | 7.91E-07 | 1.06E-06 |
| 87 | 2.23E-11 | 2.69E-10 | 5.96E-10 | 9.69E-10 | 9.68E-10 | 7.94E-10 | 2.92E-07 | 7.44E-07 | 1.04E-06 |
| 88 | -1.61E-09 | 4.73E-10 | -3.59E-09 | 4.29E-10 | -3.24E-10 | 4.11E-09 | 2.67E-07 | 7.21E-07 | 1.01E-06 |
| 89 | -4.99E-11 | 9.37E-12 | 1.42E-12 | 1.27E-11 | -2.25E-11 | -9.35E-11 | 2.42E-07 | 7.05E-07 | 9.89E-07 |
| 90 | -2.77E-10 | -4.86E-10 | -3.79E-10 | -2.04E-11 | 2.53E-10 | 7.15E-11 | 2.18E-07 | 6.84E-07 | 9.65E-07 |
| 91 | 1.72E-09 | 1.24E-10 | -1.40E-09 | -2.74E-09 | -3.67E-09 | 2.58E-09 | 1.95E-07 | 6.66E-07 | 9.42E-07 |
| 92 | 8.72E-12 | 4.96E-11 | 5.91E-11 | 2.54E-11 | -6.54E-11 | -2.47E-10 | 1.74E-07 | 6.43E-07 | 9.18E-07 |
| 93 | 2.98E-11 | 7.86E-11 | -1.06E-13 | 5.19E-12 | 6.56E-11 | 3.58E-10 | 1.56E-07 | 6.23E-07 | 8.94E-07 |
| 94 | -2.98E-10 | -6.29E-11 | 3.16E-10 | 6.30E-10 | -1.55E-11 | -2.73E-11 | 1.36E-07 | 6.00E-07 | 8.66E-07 |
| 95 | -1.49E-12 | 9.97E-11 | -9.31E-11 | 2.81E-12 | -1.03E-10 | 2.94E-10 | 1.16E-07 | 5.77E-07 | 8.38E-07 |
| 96 | 1.06E-09 | -1.50E-09 | 2.65E-10 | 2.07E-09 | -4.79E-10 | 1.80E-09 | 9.46E-08 | 5.54E-07 | 8.13E-07 |
| 97 | -1.78E-10 | -3.24E-11 | 1.22E-10 | 1.20E-10 | -4.65E-10 | 6.89E-10 | 7.38E-08 | 5.29E-07 | 7.84E-07 |
| 98 | -2.99E-11 | 9.75E-11 | 2.12E-10 | 2.17E-10 | -6.75E-11 | -1.16E-10 | 5.23E-08 | 5.06E-07 | 7.60E-07 |
| 99 | 5.25E-11 | 4.91E-11 | 3.20E-11 | -1.55E-11 | -1.18E-10 | 1.18E-10 | 3.06E-08 | 4.85E-07 | 7.39E-07 |
| 100 | -1.28E-11 | -4.08E-11 | -4.67E-12 | 3.42E-11 | -5.25E-11 | 1.80E-11 | 1.21E-08 | 4.68E-07 | 7.22E-07 |
| 101 | 8.78E-10 | 5.98E-10 | 2.23E-10 | -1.77E-10 | -4.40E-10 | -2.35E-10 | 7.92E-10 | 4.55E-07 | 7.08E-07 |
| 102 | 2.22E-12 | -5.30E-12 | -8.86E-12 | -4.77E-12 | 1.35E-11 | 3.06E-12 | -1.00E-11 | 4.41E-07 | 6.87E-07 |
| 103 | 2.01E-11 | 1.99E-11 | 1.53E-11 | 7.20E-12 | -6.39E-12 | -8.68E-11 | 1.49E-10 | 4.21E-07 | 6.71E-07 |
| 104 | -1.32E-10 | -4.03E-11 | 7.19E-12 | 1.97E-11 | 4.93E-12 | -6.65E-11 | 4.36E-11 | 4.04E-07 | 6.51E-07 |
| 105 | 5.45E-11 | 5.66E-12 | -8.69E-12 | 3.42E-13 | 1.37E-11 | -8.06E-11 | 1.69E-11 | 3.85E-07 | 6.29E-07 |
| 106 | 1.81E-11 | -2.93E-12 | -1.15E-11 | -1.86E-11 | -2.77E-11 | -5.14E-11 | 2.56E-10 | 3.64E-07 | 6.11E-07 |
| 107 | -1.98E-11 | -2.41E-11 | -1.65E-11 | 2.67E-12 | 1.99E-11 | -3.98E-11 | -2.30E-12 | 3.43E-07 | 5.94E-07 |
| 108 | 2.73E-11 | 1.83E-11 | 7.02E-12 | -4.73E-12 | -1.58E-11 | -4.01E-11 | 1.39E-10 | 3.30E-07 | 5.77E-07 |
| 109 | -1.55E-10 | -8.76E-11 | -1.55E-11 | 3.48E-11 | 4.01E-11 | -1.65E-10 | -1.66E-10 | 3.14E-07 | 5.62E-07 |
| 110 | -2.42E-12 | -3.31E-12 | -2.67E-12 | 7.45E-13 | 7.12E-12 | -3.24E-11 | -1.00E-10 | 3.02E-07 | 5.49E-07 |
| 111 | -6.20E-11 | -4.14E-11 | -1.79E-11 | 1.02E-11 | 3.92E-11 | 3.02E-11 | 8.30E-11 | 2.88E-07 | 5.35E-07 |
| 112 | 9.54E-12 | 1.18E-11 | 1.12E-11 | 6.30E-12 | -5.62E-12 | -6.38E-11 | 5.87E-11 | 2.73E-07 | 5.20E-07 |
| 113 | -1.38E-10 | 1.87E-11 | 1.39E-10 | 2.00E-10 | 1.41E-10 | -6.32E-10 | 1.20E-10 | 2.52E-07 | 5.02E-07 |
| 114 | 2.48E-10 | 2.97E-10 | 3.46E-10 | 4.01E-10 | 4.77E-10 | 6.10E-10 | 5.92E-10 | 2.33E-07 | 4.83E-07 |
| 115 | 2.41E-11 | -1.76E-10 | -1.34E-10 | 5.34E-12 | 1.40E-10 | 2.15E-10 | -1.51E-10 | 2.11E-07 | 4.62E-07 |
| 116 | -6.53E-13 | 9.25E-12 | -5.84E-12 | -2.92E-11 | -4.93E-11 | -9.36E-11 | -8.09E-12 | 1.91E-07 | 4.42E-07 |
| 117 | -5.00E-11 | -3.20E-10 | -3.65E-10 | -8.91E-11 | 3.40E-10 | -6.86E-10 | 5.91E-12 | 1.74E-07 | 4.21E-07 |
| 118 | -4.81E-11 | -2.80E-11 | 1.34E-11 | 7.11E-11 | 1.19E-10 | 3.13E-11 | 1.00E-10 | 1.60E-07 | 4.03E-07 |
| 119 | -6.52E-11 | -3.86E-11 | -6.12E-13 | 4.33E-11 | 8.26E-11 | 8.28E-12 | 2.22E-11 | 1.47E-07 | 3.87E-07 |
| 120 | 1.14E-11 | 1.04E-11 | 8.66E-12 | 4.91E-12 | -3.50E-12 | -6.55E-11 | -1.51E-10 | 1.36E-07 | 3.71E-07 |
| 121 | 1.23E-10 | 9.35E-11 | 3.21E-11 | -1.49E-11 | 6.53E-11 | 7.64E-12 | -1.01E-11 | 1.24E-07 | 3.58E-07 |
| 122 | -3.75E-11 | -9.18E-12 | -1.83E-11 | -3.21E-11 | 4.49E-11 | -8.22E-11 | 9.89E-11 | 1.12E-07 | 3.45E-07 |
| 123 | -4.57E-11 | -2.82E-11 | -7.58E-12 | 1.38E-11 | 2.78E-11 | -9.41E-11 | 7.20E-11 | 9.78E-08 | 3.33E-07 |
| 124 | -4.66E-11 | -2.22E-11 | 8.50E-12 | 2.35E-11 | 7.00E-12 | -1.60E-10 | 1.73E-10 | 8.35E-08 | 3.20E-07 |
| 125 | 2.95E-10 | 2.29E-10 | 1.45E-10 | 4.99E-11 | -5.37E-11 | -2.82E-10 | 1.53E-12 | 7.00E-08 | 3.07E-07 |
| 126 | -1.67E-11 | -1.28E-11 | -4.45E-12 | 1.08E-11 | 3.28E-11 | -1.89E-12 | 5.58E-14 | 5.59E-08 | 2.94E-07 |
| 127 | -3.15E-12 | -7.89E-12 | -7.64E-12 | 5.40E-13 | 1.70E-11 | 1.25E-11 | -1.37E-11 | 4.30E-08 | 2.80E-07 |
| 128 | 7.54E-11 | 2.79E-12 | -2.34E-11 | -1.17E-11 | 1.53E-11 | -3.58E-11 | -3.79E-11 | 3.08E-08 | 2.64E-07 |
| 129 | 1.07E-11 | 1.06E-11 | 8.64E-12 | 4.20E-12 | -2.28E-12 | -1.32E-11 | 1.37E-10 | 1.96E-08 | 2.49E-07 |
| 130 | 8.79E-12 | 8.16E-12 | 5.90E-12 | -1.28E-12 | -2.16E-11 | -1.18E-10 | -1.69E-11 | 1.02E-08 | 2.33E-07 |
| 131 | 9.54E-11 | 5.72E-11 | 1.64E-11 | -3.12E-11 | -9.94E-11 | -5.84E-10 | -2.36E-10 | 6.89E-10 | 2.18E-07 |
| 132 | 3.86E-11 | 1.23E-11 | -1.13E-11 | -3.22E-11 | -5.09E-11 | -8.56E-11 | -1.22E-10 | 1.34E-10 | 2.04E-07 |
| 133 | 5.37E-12 | 3.30E-12 | 2.44E-12 | 1.26E-12 | -1.28E-12 | -5.84E-12 | -2.29E-11 | 1.65E-11 | 1.91E-07 |
| 134 | -6.74E-13 | -4.65E-13 | -2.62E-13 | -1.14E-13 | -1.57E-14 | 1.81E-12 | -1.76E-12 | -2.18E-14 | 1.79E-07 |
| 135 | 4.13E-11 | 3.22E-11 | 2.11E-11 | 9.62E-12 | -3.03E-13 | -1.86E-12 | -8.15E-11 | 9.87E-11 | 1.67E-07 |
| 136 | 8.79E-12 | 3.71E-12 | -3.00E-12 | -1.09E-11 | -1.84E-11 | -3.54E-11 | -1.22E-10 | 1.13E-10 | 1.56E-07 |
| 137 | -2.02E-11 | -7.13E-12 | 1.15E-11 | 3.30E-11 | 5.28E-11 | 5.24E-11 | -7.30E-11 | 7.58E-11 | 1.44E-07 |
| 138 | -2.72E-12 | -1.98E-12 | -2.87E-12 | -5.03E-12 | -8.18E-12 | -1.69E-11 | -4.56E-11 | -1.43E-10 | 1.32E-07 |
| 139 | -2.33E-11 | -2.04E-11 | -3.18E-12 | 1.34E-11 | 2.30E-11 | 1.62E-11 | -1.91E-11 | -3.33E-11 | 1.20E-07 |
| 140 | -3.31E-12 | -1.83E-12 | 1.75E-13 | 2.43E-12 | 4.37E-12 | 4.72E-12 | -5.59E-12 | -2.25E-11 | 1.09E-07 |
| 141 | 2.65E-12 | 1.33E-12 | 2.47E-13 | -6.33E-13 | -1.24E-12 | -1.46E-13 | 7.05E-14 | -1.17E-12 | 9.76E-08 |
| 142 | 6.99E-13 | 3.59E-13 | -7.22E-13 | -1.95E-12 | -2.47E-12 | 7.42E-13 | -7.46E-13 | -7.91E-13 | 8.71E-08 |
| 143 | 3.72E-12 | 1.79E-12 | -1.31E-13 | -1.99E-12 | -3.80E-12 | -8.03E-12 | -1.85E-11 | 6.17E-11 | 7.72E-08 |
| 144 | -7.73E-12 | -8.86E-12 | -1.04E-11 | -9.85E-12 | -4.93E-12 | 1.06E-11 | -8.15E-12 | -1.09E-12 | 6.75E-08 |
| 145 | -8.29E-12 | -3.27E-12 | 2.04E-12 | 6.93E-12 | 1.06E-11 | 1.15E-11 | -2.86E-12 | -1.25E-11 | 5.76E-08 |
| 146 | -6.85E-12 | -5.44E-12 | -4.00E-12 | -2.50E-12 | -9.07E-13 | 2.50E-12 | -5.52E-13 | 1.15E-11 | 4.80E-08 |
| 147 | -1.68E-11 | -1.44E-11 | -1.03E-11 | -3.91E-12 | 5.00E-12 | 2.32E-11 | -3.34E-11 | 4.00E-11 | 3.77E-08 |
| 148 | -1.04E-13 | -9.16E-14 | -6.08E-14 | 6.16E-15 | 1.38E-13 | 5.79E-13 | -1.48E-12 | 2.44E-13 | 2.68E-08 |
| 149 | 6.08E-12 | 4.66E-12 | 3.29E-12 | 1.95E-12 | 5.75E-13 | -1.71E-12 | -2.55E-12 | 1.91E-11 | 1.58E-08 |
| 150 | -3.66E-13 | -3.71E-13 | -3.05E-13 | -2.04E-13 | -8.57E-14 | 2.59E-13 | 3.86E-13 | 1.76E-13 | 3.73E-09 |

Table.21. Spectra predicted by MLP neural network: Al- 1.6mm

| energy bin (kev)/voltage(kv) | 20kv | 30kv | 40kv | 50kv | 60kv | 80kv | 100kv | 130kv | 150kv |
| --- | --- | --- | --- | --- | --- | --- | --- | --- | --- |
| 1 | 0 | 0 | 0 | 0 | 0 | 0 | 0 | 0 | 0 |
| 2 | 9.63E-10 | -1.06E-14 | -2.90E-15 | 3.47E-15 | -3.96E-16 | -6.87E-14 | 5.54E-14 | 3.96E-13 | -4.75E-14 |
| 3 | -1.71E-10 | 7.23E-11 | 6.54E-11 | 5.43E-11 | 3.81E-11 | 1.16E-11 | 9.55E-11 | 1.48E-11 | 1.66E-10 |
| 4 | 8.68E-08 | 6.62E-08 | 5.98E-08 | 5.16E-08 | 4.45E-08 | 3.67E-08 | 3.56E-08 | 4.43E-08 | 4.85E-08 |
| 5 | 4.67E-07 | 6.48E-07 | 6.60E-07 | 6.15E-07 | 5.55E-07 | 4.96E-07 | 4.85E-07 | 4.51E-07 | 4.38E-07 |
| 6 | 1.08E-06 | 1.70E-06 | 1.90E-06 | 1.88E-06 | 1.79E-06 | 1.64E-06 | 1.57E-06 | 1.48E-06 | 1.31E-06 |
| 7 | 1.62E-06 | 2.54E-06 | 3.09E-06 | 3.13E-06 | 3.01E-06 | 2.87E-06 | 2.87E-06 | 2.92E-06 | 2.44E-06 |
| 8 | 2.05E-06 | 3.16E-06 | 3.88E-06 | 4.34E-06 | 4.57E-06 | 4.62E-06 | 4.58E-06 | 4.47E-06 | 4.05E-06 |
| 9 | 2.06E-06 | 4.20E-06 | 5.82E-06 | 7.11E-06 | 8.17E-06 | 9.55E-06 | 1.03E-05 | 1.20E-05 | 1.06E-05 |
| 10 | 1.98E-06 | 3.30E-06 | 4.39E-06 | 5.28E-06 | 6.00E-06 | 7.01E-06 | 7.63E-06 | 8.05E-06 | 7.36E-06 |
| 11 | 1.75E-06 | 2.65E-06 | 3.42E-06 | 3.93E-06 | 4.23E-06 | 4.48E-06 | 4.57E-06 | 4.94E-06 | 4.22E-06 |
| 12 | 1.45E-06 | 2.41E-06 | 3.07E-06 | 3.48E-06 | 3.70E-06 | 3.83E-06 | 3.85E-06 | 4.10E-06 | 3.39E-06 |
| 13 | 1.23E-06 | 2.12E-06 | 2.74E-06 | 3.08E-06 | 3.24E-06 | 3.27E-06 | 3.24E-06 | 3.32E-06 | 2.83E-06 |
| 14 | 1.05E-06 | 1.94E-06 | 2.59E-06 | 3.04E-06 | 3.33E-06 | 3.61E-06 | 3.69E-06 | 3.57E-06 | 3.18E-06 |
| 15 | 8.85E-07 | 1.85E-06 | 2.51E-06 | 3.02E-06 | 3.34E-06 | 3.63E-06 | 3.83E-06 | 3.94E-06 | 3.53E-06 |
| 16 | 7.28E-07 | 1.72E-06 | 2.44E-06 | 2.96E-06 | 3.29E-06 | 3.70E-06 | 4.10E-06 | 4.27E-06 | 3.84E-06 |
| 17 | 5.67E-07 | 1.57E-06 | 2.32E-06 | 2.87E-06 | 3.29E-06 | 3.80E-06 | 4.10E-06 | 4.59E-06 | 4.10E-06 |
| 18 | 4.11E-07 | 1.41E-06 | 2.18E-06 | 2.77E-06 | 3.21E-06 | 3.80E-06 | 4.20E-06 | 4.77E-06 | 4.34E-06 |
| 19 | 2.53E-07 | 1.26E-06 | 2.03E-06 | 2.65E-06 | 3.14E-06 | 3.81E-06 | 4.27E-06 | 4.94E-06 | 4.54E-06 |
| 20 | 9.56E-08 | 1.13E-06 | 1.91E-06 | 2.51E-06 | 3.00E-06 | 3.75E-06 | 4.34E-06 | 5.00E-06 | 4.71E-06 |
| 21 | 9.66E-10 | 9.79E-07 | 1.76E-06 | 2.40E-06 | 2.91E-06 | 3.69E-06 | 4.32E-06 | 5.12E-06 | 4.87E-06 |
| 22 | -1.32E-09 | 9.01E-07 | 1.65E-06 | 2.27E-06 | 2.80E-06 | 3.62E-06 | 4.28E-06 | 5.13E-06 | 4.96E-06 |
| 23 | 1.39E-09 | 7.88E-07 | 1.50E-06 | 2.13E-06 | 2.69E-06 | 3.57E-06 | 4.22E-06 | 5.06E-06 | 5.04E-06 |
| 24 | -1.22E-09 | 7.02E-07 | 1.39E-06 | 2.02E-06 | 2.56E-06 | 3.43E-06 | 4.17E-06 | 5.10E-06 | 5.08E-06 |
| 25 | 8.90E-10 | 5.95E-07 | 1.26E-06 | 1.90E-06 | 2.46E-06 | 3.33E-06 | 4.10E-06 | 5.07E-06 | 5.10E-06 |
| 26 | -1.07E-09 | 5.43E-07 | 1.18E-06 | 1.79E-06 | 2.33E-06 | 3.22E-06 | 4.00E-06 | 5.00E-06 | 5.11E-06 |
| 27 | -6.42E-10 | 3.91E-07 | 1.07E-06 | 1.69E-06 | 2.22E-06 | 3.11E-06 | 3.89E-06 | 4.93E-06 | 5.08E-06 |
| 28 | -1.33E-09 | 3.50E-07 | 9.93E-07 | 1.61E-06 | 2.10E-06 | 2.98E-06 | 3.84E-06 | 4.81E-06 | 5.10E-06 |
| 29 | 1.32E-09 | 1.75E-07 | 8.86E-07 | 1.47E-06 | 1.98E-06 | 2.90E-06 | 3.68E-06 | 4.74E-06 | 4.99E-06 |
| 30 | -5.99E-10 | 2.70E-07 | 7.52E-07 | 1.31E-06 | 1.88E-06 | 2.91E-06 | 3.74E-06 | 4.60E-06 | 4.98E-06 |
| 31 | 1.89E-09 | 2.38E-07 | 6.63E-07 | 1.19E-06 | 1.76E-06 | 2.73E-06 | 3.46E-06 | 4.35E-06 | 4.77E-06 |
| 32 | 1.27E-09 | 7.02E-09 | 6.35E-07 | 1.19E-06 | 1.67E-06 | 2.57E-06 | 3.38E-06 | 4.35E-06 | 4.80E-06 |
| 33 | -1.06E-10 | 6.73E-09 | 5.51E-07 | 1.10E-06 | 1.57E-06 | 2.46E-06 | 3.26E-06 | 4.23E-06 | 4.73E-06 |
| 34 | -1.39E-11 | 9.24E-09 | 4.77E-07 | 1.04E-06 | 1.49E-06 | 2.36E-06 | 3.17E-06 | 4.11E-06 | 4.64E-06 |
| 35 | -1.34E-09 | 1.28E-08 | 3.82E-07 | 9.64E-07 | 1.41E-06 | 2.25E-06 | 3.03E-06 | 3.97E-06 | 4.53E-06 |
| 36 | -1.53E-09 | 5.17E-08 | 3.25E-07 | 8.88E-07 | 1.34E-06 | 2.12E-06 | 2.94E-06 | 3.86E-06 | 4.41E-06 |
| 37 | -7.03E-10 | 1.02E-07 | 3.49E-07 | 6.91E-07 | 1.12E-06 | 2.07E-06 | 2.79E-06 | 3.80E-06 | 4.42E-06 |
| 38 | -1.17E-09 | 3.83E-09 | 1.83E-07 | 7.33E-07 | 1.17E-06 | 1.92E-06 | 2.72E-06 | 3.67E-06 | 4.20E-06 |
| 39 | 5.00E-11 | 8.15E-11 | 1.13E-07 | 6.61E-07 | 1.10E-06 | 1.85E-06 | 2.61E-06 | 3.57E-06 | 4.18E-06 |
| 40 | 8.88E-10 | -2.28E-09 | 5.42E-08 | 5.98E-07 | 1.03E-06 | 1.78E-06 | 2.51E-06 | 3.47E-06 | 3.98E-06 |
| 41 | 2.62E-10 | 2.42E-09 | -4.04E-09 | 5.37E-07 | 9.60E-07 | 1.71E-06 | 2.41E-06 | 3.37E-06 | 3.89E-06 |
| 42 | -1.78E-09 | -1.62E-09 | 9.57E-09 | 4.72E-07 | 9.08E-07 | 1.64E-06 | 2.33E-06 | 3.28E-06 | 3.80E-06 |
| 43 | -1.86E-09 | -3.68E-10 | 6.23E-09 | 4.00E-07 | 8.45E-07 | 1.56E-06 | 2.23E-06 | 3.15E-06 | 3.70E-06 |
| 44 | -1.70E-09 | 5.01E-09 | 1.85E-09 | 3.63E-07 | 7.76E-07 | 1.51E-06 | 2.15E-06 | 3.05E-06 | 3.59E-06 |
| 45 | 1.64E-09 | -9.78E-10 | 3.19E-10 | 3.04E-07 | 7.29E-07 | 1.44E-06 | 2.09E-06 | 2.98E-06 | 3.48E-06 |
| 46 | 1.21E-09 | -3.47E-09 | 4.15E-10 | 2.54E-07 | 6.70E-07 | 1.37E-06 | 2.02E-06 | 2.89E-06 | 3.38E-06 |
| 47 | -1.02E-09 | -7.69E-09 | 2.06E-09 | 1.98E-07 | 6.21E-07 | 1.31E-06 | 1.97E-06 | 2.82E-06 | 3.32E-06 |
| 48 | 1.51E-09 | 8.40E-10 | 2.40E-09 | 1.46E-07 | 5.66E-07 | 1.24E-06 | 1.88E-06 | 2.70E-06 | 3.23E-06 |
| 49 | 1.43E-09 | 7.26E-09 | -2.12E-08 | 1.13E-07 | 4.99E-07 | 1.19E-06 | 1.78E-06 | 2.63E-06 | 3.19E-06 |
| 50 | -1.74E-09 | -4.44E-11 | -4.06E-10 | 3.74E-08 | 4.71E-07 | 1.13E-06 | 1.74E-06 | 2.54E-06 | 3.06E-06 |
| 51 | -2.48E-10 | 3.01E-08 | -2.26E-08 | 3.00E-08 | 4.25E-07 | 1.12E-06 | 1.66E-06 | 2.48E-06 | 2.98E-06 |
| 52 | 4.95E-10 | -1.95E-08 | -1.15E-08 | 3.67E-08 | 2.64E-07 | 1.03E-06 | 1.60E-06 | 2.40E-06 | 2.90E-06 |
| 53 | -1.80E-09 | -1.75E-08 | -2.00E-08 | 5.93E-08 | 2.59E-07 | 9.58E-07 | 1.56E-06 | 2.32E-06 | 2.70E-06 |
| 54 | -1.30E-09 | 1.07E-08 | -1.30E-08 | 4.91E-08 | 2.73E-07 | 9.22E-07 | 1.50E-06 | 2.28E-06 | 2.77E-06 |
| 55 | 1.10E-09 | -3.76E-10 | -9.38E-10 | 9.60E-10 | 2.21E-07 | 8.67E-07 | 1.46E-06 | 2.22E-06 | 2.70E-06 |
| 56 | 1.48E-11 | 1.92E-08 | -2.69E-08 | 1.08E-08 | 1.99E-07 | 7.86E-07 | 1.39E-06 | 2.17E-06 | 2.63E-06 |
| 57 | -1.70E-10 | 5.69E-09 | -6.57E-09 | 2.70E-09 | 1.45E-07 | 7.74E-07 | 1.34E-06 | 2.08E-06 | 2.58E-06 |
| 58 | -3.15E-10 | 1.92E-08 | -4.84E-09 | -9.98E-09 | 1.15E-07 | 7.33E-07 | 1.28E-06 | 1.97E-06 | 2.51E-06 |
| 59 | -7.16E-10 | 1.17E-10 | 2.04E-09 | -8.49E-10 | 7.07E-08 | 8.49E-07 | 2.24E-06 | 4.83E-06 | 6.53E-06 |
| 60 | 6.52E-10 | 1.29E-08 | 5.19E-09 | -2.25E-08 | 3.68E-08 | 9.34E-07 | 2.96E-06 | 7.09E-06 | 9.87E-06 |
| 61 | 8.61E-10 | 4.76E-09 | 3.59E-09 | -1.88E-08 | 1.50E-08 | 5.95E-07 | 1.10E-06 | 1.86E-06 | 2.37E-06 |
| 62 | -1.16E-09 | -2.78E-09 | 2.57E-09 | -1.29E-09 | -9.90E-10 | 5.93E-07 | 1.19E-06 | 1.93E-06 | 2.57E-06 |
| 63 | -6.68E-10 | 2.67E-09 | 3.78E-09 | 7.27E-10 | 5.75E-09 | 5.92E-07 | 1.03E-06 | 1.62E-06 | 2.09E-06 |
| 64 | 8.10E-10 | 9.30E-11 | 6.31E-10 | 4.71E-10 | 1.90E-09 | 5.13E-07 | 1.01E-06 | 1.54E-06 | 2.03E-06 |
| 65 | 1.18E-09 | 7.97E-11 | 8.85E-11 | 2.27E-10 | 1.05E-09 | 4.74E-07 | 9.68E-07 | 1.54E-06 | 2.00E-06 |
| 66 | -3.97E-11 | 2.27E-08 | -7.97E-09 | -2.88E-08 | 2.66E-08 | 3.97E-07 | 9.05E-07 | 1.53E-06 | 1.97E-06 |
| 67 | 1.19E-09 | -7.94E-11 | -4.13E-10 | -3.24E-10 | -4.23E-10 | 4.27E-07 | 8.48E-07 | 1.48E-06 | 1.93E-06 |
| 68 | 1.69E-09 | -1.59E-10 | -1.31E-09 | -1.67E-09 | 2.03E-09 | 4.81E-07 | 1.50E-06 | 3.23E-06 | 4.58E-06 |
| 69 | -6.24E-10 | 7.62E-10 | 1.51E-09 | -3.13E-09 | 1.49E-09 | 3.38E-07 | 7.98E-07 | 1.43E-06 | 1.75E-06 |
| 70 | 1.59E-09 | -1.71E-11 | 1.86E-09 | -1.21E-09 | -2.91E-09 | 3.24E-07 | 9.44E-07 | 1.83E-06 | 2.44E-06 |
| 71 | 1.08E-09 | -2.20E-10 | -1.28E-09 | -2.63E-09 | -1.29E-09 | 2.76E-07 | 7.42E-07 | 1.24E-06 | 1.46E-06 |
| 72 | -6.67E-11 | -4.13E-11 | 4.93E-10 | 6.18E-10 | 9.45E-11 | 2.52E-07 | 6.59E-07 | 1.21E-06 | 1.47E-06 |
| 73 | -1.61E-09 | 3.27E-09 | 2.31E-09 | -5.50E-09 | 3.25E-09 | 2.14E-07 | 6.27E-07 | 1.16E-06 | 1.43E-06 |
| 74 | 1.37E-09 | -2.65E-10 | 1.66E-09 | -2.13E-09 | 1.42E-09 | 1.84E-07 | 5.99E-07 | 1.13E-06 | 1.40E-06 |
| 75 | -1.09E-10 | 2.72E-10 | 4.14E-10 | -1.49E-09 | 1.11E-09 | 1.54E-07 | 5.72E-07 | 1.09E-06 | 1.36E-06 |
| 76 | -3.56E-11 | -2.07E-10 | 2.66E-10 | -2.26E-10 | 9.52E-10 | 1.27E-07 | 5.46E-07 | 1.07E-06 | 1.32E-06 |
| 77 | -1.57E-09 | 2.78E-10 | 5.16E-10 | -4.21E-10 | -9.57E-11 | 9.85E-08 | 5.24E-07 | 1.03E-06 | 1.29E-06 |
| 78 | -6.68E-10 | -6.47E-10 | -6.69E-10 | -8.50E-10 | 4.64E-10 | 7.22E-08 | 5.06E-07 | 1.00E-06 | 1.27E-06 |
| 79 | -2.00E-09 | 1.40E-10 | -3.43E-11 | -1.97E-10 | 5.31E-10 | 4.53E-08 | 4.84E-07 | 9.69E-07 | 1.23E-06 |
| 80 | -7.35E-10 | 4.06E-10 | 1.03E-10 | 7.04E-10 | -2.38E-09 | 1.55E-08 | 4.56E-07 | 9.33E-07 | 1.21E-06 |
| 81 | 1.73E-10 | 2.22E-10 | 2.01E-10 | -1.72E-10 | -8.53E-10 | -3.44E-10 | 4.34E-07 | 8.97E-07 | 1.18E-06 |
| 82 | 1.65E-09 | 4.69E-10 | 3.61E-10 | 1.08E-10 | -4.58E-10 | 6.55E-10 | 4.07E-07 | 8.66E-07 | 1.16E-06 |
| 83 | 1.10E-09 | 1.05E-10 | 2.11E-10 | 2.25E-10 | -6.37E-11 | 2.50E-10 | 3.83E-07 | 8.35E-07 | 1.13E-06 |
| 84 | -1.55E-10 | -5.79E-09 | 6.06E-10 | 1.34E-08 | 3.56E-08 | 1.29E-07 | 3.33E-07 | 8.18E-07 | 1.08E-06 |
| 85 | -1.82E-09 | 1.62E-10 | 8.40E-11 | -2.37E-11 | -1.59E-10 | 6.81E-10 | 3.36E-07 | 7.81E-07 | 1.08E-06 |
| 86 | -3.71E-10 | 8.84E-09 | 4.68E-09 | -4.64E-09 | -8.87E-09 | 4.90E-08 | 2.51E-07 | 7.83E-07 | 1.06E-06 |
| 87 | 1.63E-09 | -7.06E-11 | -1.78E-11 | 1.02E-10 | -9.91E-11 | -3.70E-10 | 2.87E-07 | 7.38E-07 | 1.03E-06 |
| 88 | -9.84E-10 | 1.32E-09 | -2.87E-09 | 6.91E-10 | -5.72E-10 | 1.71E-09 | 2.65E-07 | 7.15E-07 | 9.92E-07 |
| 89 | -6.99E-10 | 2.84E-11 | 5.26E-13 | 2.38E-11 | 4.05E-11 | 6.25E-11 | 2.40E-07 | 6.98E-07 | 9.79E-07 |
| 90 | 1.24E-09 | -1.54E-10 | -8.09E-11 | 2.37E-10 | 4.66E-10 | 2.10E-10 | 2.16E-07 | 6.77E-07 | 9.55E-07 |
| 91 | 1.39E-09 | -3.13E-10 | -1.75E-09 | -2.83E-09 | -3.41E-09 | 3.33E-09 | 1.95E-07 | 6.59E-07 | 9.32E-07 |
| 92 | -3.80E-10 | -1.65E-11 | -1.19E-11 | 2.93E-13 | 5.24E-13 | 8.11E-11 | 1.73E-07 | 6.37E-07 | 9.09E-07 |
| 93 | 1.07E-09 | 7.04E-11 | 8.61E-12 | -8.72E-11 | -7.46E-11 | 5.92E-11 | 1.54E-07 | 6.17E-07 | 8.85E-07 |
| 94 | 9.34E-10 | -5.80E-11 | 3.48E-10 | 7.08E-10 | 1.40E-10 | 1.61E-10 | 1.35E-07 | 5.94E-07 | 8.57E-07 |
| 95 | 9.42E-10 | 1.21E-10 | -5.75E-11 | 5.65E-11 | -2.41E-11 | 1.84E-10 | 1.15E-07 | 5.72E-07 | 8.29E-07 |
| 96 | -1.29E-09 | -2.58E-09 | -1.17E-09 | 1.58E-09 | 6.83E-10 | 3.65E-09 | 9.34E-08 | 5.48E-07 | 8.09E-07 |
| 97 | 1.74E-09 | -1.73E-10 | -3.55E-11 | -1.72E-11 | -5.01E-10 | 9.82E-10 | 7.37E-08 | 5.26E-07 | 7.77E-07 |
| 98 | -1.80E-09 | 7.76E-11 | 1.82E-10 | 1.83E-10 | -7.76E-11 | 2.42E-11 | 5.17E-08 | 5.01E-07 | 7.52E-07 |
| 99 | 1.30E-09 | 3.54E-11 | 2.08E-11 | -2.85E-11 | -1.41E-10 | 2.42E-11 | 3.03E-08 | 4.80E-07 | 7.32E-07 |
| 100 | 8.28E-10 | -3.44E-11 | -3.92E-12 | 2.83E-11 | -5.77E-11 | 4.96E-11 | 1.20E-08 | 4.63E-07 | 7.15E-07 |
| 101 | -1.01E-09 | 5.37E-10 | 8.29E-11 | -1.98E-10 | -2.42E-10 | -5.46E-10 | -1.46E-09 | 4.51E-07 | 7.02E-07 |
| 102 | 1.04E-09 | -1.32E-12 | -1.06E-11 | -1.28E-11 | -4.62E-13 | -7.70E-12 | 2.82E-12 | 4.35E-07 | 6.81E-07 |
| 103 | 1.74E-09 | 2.56E-12 | 3.11E-11 | 4.69E-11 | 4.85E-11 | -2.57E-11 | 1.58E-10 | 4.17E-07 | 6.64E-07 |
| 104 | -2.95E-10 | -2.62E-11 | -8.92E-12 | -9.65E-12 | -2.50E-11 | -9.24E-11 | -3.33E-11 | 4.00E-07 | 6.45E-07 |
| 105 | 8.47E-10 | -1.08E-11 | -2.49E-11 | -2.15E-11 | -1.10E-11 | -9.40E-11 | -1.92E-11 | 3.81E-07 | 6.24E-07 |
| 106 | -8.72E-10 | 3.54E-11 | -4.13E-11 | -1.02E-10 | -1.44E-10 | -1.63E-10 | 2.34E-10 | 3.60E-07 | 6.06E-07 |
| 107 | -1.54E-09 | -1.29E-11 | -5.89E-12 | 9.46E-12 | 2.05E-11 | -6.14E-11 | 4.18E-11 | 3.40E-07 | 5.88E-07 |
| 108 | -1.42E-09 | 6.49E-13 | -5.79E-12 | -1.04E-11 | -1.43E-11 | -2.59E-11 | 1.52E-10 | 3.26E-07 | 5.72E-07 |
| 109 | -7.56E-10 | -1.75E-11 | 9.14E-11 | 1.54E-10 | 1.70E-10 | -3.57E-12 | -1.25E-10 | 3.11E-07 | 5.57E-07 |
| 110 | -2.76E-10 | -8.56E-14 | 5.62E-16 | 1.49E-12 | 3.87E-12 | -3.66E-11 | 6.59E-11 | 2.99E-07 | 5.44E-07 |
| 111 | -1.87E-09 | -1.95E-11 | -1.92E-11 | -1.11E-11 | 2.64E-12 | -2.86E-11 | -4.75E-12 | 2.85E-07 | 5.29E-07 |
| 112 | 1.22E-09 | 1.43E-11 | 8.76E-12 | -1.16E-12 | -1.81E-11 | -8.13E-11 | 7.88E-11 | 2.70E-07 | 5.15E-07 |
| 113 | -7.96E-10 | -1.21E-10 | -1.34E-10 | -1.90E-10 | -3.32E-10 | -9.94E-10 | -1.88E-10 | 2.50E-07 | 5.01E-07 |
| 114 | -6.45E-10 | 1.23E-10 | 1.51E-10 | 1.74E-10 | 1.95E-10 | 1.75E-10 | -8.34E-11 | 2.30E-07 | 4.78E-07 |
| 115 | -1.88E-09 | -1.22E-10 | -1.14E-10 | -2.22E-11 | 7.30E-11 | 1.65E-10 | -7.03E-11 | 2.09E-07 | 4.58E-07 |
| 116 | 1.75E-09 | 2.11E-11 | -1.24E-11 | -5.26E-11 | -7.84E-11 | -1.02E-10 | -5.96E-12 | 1.90E-07 | 4.38E-07 |
| 117 | -9.01E-10 | -2.91E-10 | -3.58E-10 | -7.35E-11 | 3.99E-10 | -3.51E-10 | 5.29E-10 | 1.72E-07 | 4.17E-07 |
| 118 | -1.18E-09 | -3.17E-11 | 1.40E-11 | 7.42E-11 | 1.25E-10 | 3.78E-11 | 4.07E-11 | 1.59E-07 | 3.99E-07 |
| 119 | 8.32E-10 | -4.30E-11 | -9.62E-12 | 3.82E-11 | 8.64E-11 | 3.29E-11 | 1.01E-11 | 1.46E-07 | 3.83E-07 |
| 120 | -9.48E-10 | 1.30E-11 | 1.12E-11 | 7.91E-12 | 6.44E-13 | -5.77E-11 | -1.57E-10 | 1.35E-07 | 3.68E-07 |
| 121 | 1.49E-09 | 3.73E-11 | -2.09E-12 | -2.17E-11 | 6.29E-11 | 7.13E-11 | 1.47E-10 | 1.23E-07 | 3.54E-07 |
| 122 | -1.39E-09 | -7.15E-12 | -1.29E-10 | -2.85E-10 | -2.46E-10 | -1.01E-10 | 5.98E-11 | 1.11E-07 | 3.42E-07 |
| 123 | 5.73E-10 | -6.62E-11 | -1.87E-11 | 3.02E-11 | 7.08E-11 | -1.21E-11 | 1.22E-10 | 9.68E-08 | 3.29E-07 |
| 124 | -1.22E-09 | 2.04E-11 | 3.85E-11 | 4.53E-11 | 1.06E-11 | -2.52E-10 | 1.02E-10 | 8.27E-08 | 3.17E-07 |
| 125 | -1.17E-09 | 1.30E-10 | 1.28E-10 | 1.10E-10 | 6.68E-11 | -1.33E-10 | 9.76E-11 | 6.91E-08 | 3.04E-07 |
| 126 | 6.12E-11 | -1.24E-11 | -6.15E-12 | 3.69E-12 | 1.45E-11 | -4.17E-11 | -9.66E-11 | 5.53E-08 | 2.91E-07 |
| 127 | 2.00E-09 | -1.82E-11 | -1.71E-11 | -6.81E-12 | 1.22E-11 | 4.63E-12 | -5.45E-11 | 4.26E-08 | 2.77E-07 |
| 128 | 6.43E-10 | -1.04E-12 | -2.19E-11 | -9.68E-12 | 2.14E-11 | -5.00E-12 | 1.52E-11 | 3.04E-08 | 2.62E-07 |
| 129 | -1.97E-09 | 1.43E-11 | 1.16E-11 | 6.36E-12 | -1.10E-12 | -1.60E-11 | 1.26E-10 | 1.94E-08 | 2.46E-07 |
| 130 | 1.55E-09 | 5.47E-12 | -5.67E-13 | -1.77E-11 | -3.85E-11 | -1.34E-11 | 6.75E-11 | 9.64E-09 | 2.31E-07 |
| 131 | 1.33E-09 | 1.12E-10 | 9.10E-11 | 6.53E-11 | 2.46E-11 | -3.58E-10 | 9.82E-11 | 1.58E-09 | 2.16E-07 |
| 132 | 1.18E-12 | 3.48E-11 | 2.10E-11 | 5.48E-12 | -1.16E-11 | -4.64E-11 | -7.68E-11 | 2.14E-10 | 2.02E-07 |
| 133 | -7.85E-10 | -5.15E-12 | -2.59E-12 | 7.07E-13 | 2.85E-12 | 4.70E-12 | -1.67E-11 | 9.76E-11 | 1.90E-07 |
| 134 | -5.64E-10 | -5.66E-13 | -4.14E-13 | -2.72E-13 | -1.41E-13 | 1.65E-12 | -3.46E-12 | 9.10E-13 | 1.77E-07 |
| 135 | -1.99E-10 | 2.04E-11 | 1.51E-11 | 1.21E-11 | 1.24E-11 | 4.10E-11 | 3.22E-11 | 3.19E-10 | 1.66E-07 |
| 136 | -7.12E-10 | 9.39E-12 | 4.79E-12 | -8.90E-13 | -6.95E-12 | -1.50E-11 | -4.94E-11 | 3.21E-10 | 1.54E-07 |
| 137 | 2.39E-10 | -4.74E-11 | 3.30E-12 | 5.50E-11 | 1.00E-10 | 1.18E-10 | -5.72E-12 | 2.74E-10 | 1.43E-07 |
| 138 | 1.31E-09 | -1.82E-12 | -2.67E-12 | -4.74E-12 | -7.78E-12 | -1.61E-11 | -4.36E-11 | -1.37E-10 | 1.31E-07 |
| 139 | 9.39E-10 | 1.90E-12 | 2.56E-12 | 2.75E-12 | 1.18E-12 | -5.06E-12 | -9.14E-12 | 5.01E-11 | 1.19E-07 |
| 140 | -9.22E-10 | -6.40E-14 | 2.53E-12 | 4.35E-12 | 5.31E-12 | 4.50E-12 | -3.79E-12 | 1.72E-11 | 1.08E-07 |
| 141 | -7.32E-11 | 4.46E-13 | -1.28E-12 | -2.55E-12 | -3.14E-12 | -1.23E-12 | -7.80E-13 | -2.95E-12 | 9.67E-08 |
| 142 | 1.81E-09 | 3.27E-13 | -5.12E-13 | -1.46E-12 | -1.82E-12 | 1.29E-12 | 2.47E-13 | 1.87E-12 | 8.64E-08 |
| 143 | -4.00E-10 | 3.14E-12 | 1.02E-12 | -1.23E-12 | -3.49E-12 | -8.18E-12 | -1.76E-11 | 7.46E-11 | 7.65E-08 |
| 144 | 6.95E-10 | -2.32E-12 | -2.55E-12 | -2.95E-12 | -5.63E-13 | 1.07E-11 | -7.95E-12 | 1.04E-11 | 6.69E-08 |
| 145 | 3.51E-10 | 6.71E-12 | 3.75E-12 | 2.03E-12 | 1.61E-12 | 2.30E-12 | -7.04E-12 | 6.06E-12 | 5.69E-08 |
| 146 | 1.85E-09 | -4.15E-12 | -3.55E-12 | -2.88E-12 | -2.14E-12 | -5.36E-13 | -5.12E-12 | 1.04E-11 | 4.75E-08 |
| 147 | -1.47E-10 | -1.66E-12 | 1.30E-12 | 3.77E-12 | 5.42E-12 | -3.60E-13 | -9.10E-11 | 3.96E-11 | 3.74E-08 |
| 148 | -1.43E-09 | -1.48E-13 | -4.81E-14 | 1.29E-13 | 4.16E-13 | 1.07E-12 | -1.12E-12 | 2.68E-12 | 2.66E-08 |
| 149 | 5.24E-10 | 4.18E-12 | 4.52E-12 | 3.71E-12 | 1.78E-12 | -4.64E-12 | -1.30E-11 | -1.74E-12 | 1.56E-08 |
| 150 | 1.93E-09 | -1.47E-13 | -6.68E-14 | 1.98E-14 | 9.06E-14 | 2.02E-13 | -6.40E-14 | 7.93E-13 | 3.70E-09 |

Table.22. Spectra predicted by MLP neural network: Al- 2mm

| energy bin (kev)/voltage(kv) | 20kv | 30kv | 40kv | 50kv | 60kv | 80kv | 100kv | 130kv | 150kv |
| --- | --- | --- | --- | --- | --- | --- | --- | --- | --- |
| 1 | 0 | 0 | 0 | 0 | 0 | 0 | 0 | 0 | 0 |
| 2 | -1.05E-09 | 9.64E-15 | 2.47E-14 | 4.02E-14 | 4.73E-14 | -1.93E-14 | 7.89E-14 | 4.80E-13 | -2.49E-13 |
| 3 | 1.87E-09 | 1.08E-11 | 1.82E-12 | -1.28E-11 | -3.70E-11 | -1.15E-10 | -5.49E-11 | 3.18E-11 | 7.75E-11 |
| 4 | 4.43E-08 | 3.32E-08 | 3.07E-08 | 2.75E-08 | 2.42E-08 | 2.03E-08 | 1.99E-08 | 2.20E-08 | 1.51E-08 |
| 5 | 3.41E-07 | 4.90E-07 | 4.94E-07 | 4.48E-07 | 3.89E-07 | 3.35E-07 | 3.39E-07 | 3.19E-07 | 2.84E-07 |
| 6 | 8.98E-07 | 1.38E-06 | 1.56E-06 | 1.56E-06 | 1.50E-06 | 1.39E-06 | 1.34E-06 | 1.25E-06 | 1.08E-06 |
| 7 | 1.41E-06 | 2.12E-06 | 2.67E-06 | 2.73E-06 | 2.63E-06 | 2.53E-06 | 2.56E-06 | 2.60E-06 | 2.12E-06 |
| 8 | 1.89E-06 | 2.80E-06 | 3.40E-06 | 3.83E-06 | 4.11E-06 | 4.32E-06 | 4.41E-06 | 4.37E-06 | 3.83E-06 |
| 9 | 1.93E-06 | 3.93E-06 | 5.46E-06 | 6.67E-06 | 7.67E-06 | 8.96E-06 | 9.61E-06 | 1.12E-05 | 9.93E-06 |
| 10 | 1.89E-06 | 3.13E-06 | 4.05E-06 | 4.82E-06 | 5.46E-06 | 6.46E-06 | 7.15E-06 | 7.64E-06 | 7.07E-06 |
| 11 | 1.68E-06 | 2.55E-06 | 3.28E-06 | 3.75E-06 | 4.03E-06 | 4.26E-06 | 4.35E-06 | 4.76E-06 | 4.03E-06 |
| 12 | 1.40E-06 | 2.33E-06 | 2.96E-06 | 3.34E-06 | 3.56E-06 | 3.67E-06 | 3.69E-06 | 3.94E-06 | 3.27E-06 |
| 13 | 1.19E-06 | 2.04E-06 | 2.65E-06 | 2.98E-06 | 3.14E-06 | 3.19E-06 | 3.17E-06 | 3.22E-06 | 2.74E-06 |
| 14 | 1.02E-06 | 1.94E-06 | 2.50E-06 | 2.93E-06 | 3.24E-06 | 3.63E-06 | 3.76E-06 | 3.55E-06 | 3.20E-06 |
| 15 | 8.62E-07 | 1.91E-06 | 2.47E-06 | 2.95E-06 | 3.29E-06 | 3.56E-06 | 3.74E-06 | 3.73E-06 | 3.43E-06 |
| 16 | 7.08E-07 | 1.63E-06 | 2.33E-06 | 2.87E-06 | 3.25E-06 | 3.63E-06 | 3.94E-06 | 4.12E-06 | 3.81E-06 |
| 17 | 5.55E-07 | 1.53E-06 | 2.27E-06 | 2.82E-06 | 3.23E-06 | 3.72E-06 | 4.00E-06 | 4.49E-06 | 4.01E-06 |
| 18 | 4.04E-07 | 1.37E-06 | 2.13E-06 | 2.71E-06 | 3.14E-06 | 3.72E-06 | 4.12E-06 | 4.67E-06 | 4.26E-06 |
| 19 | 2.49E-07 | 1.26E-06 | 2.01E-06 | 2.61E-06 | 3.09E-06 | 3.74E-06 | 4.20E-06 | 4.85E-06 | 4.47E-06 |
| 20 | 9.46E-08 | 1.14E-06 | 1.88E-06 | 2.46E-06 | 2.95E-06 | 3.69E-06 | 4.27E-06 | 4.90E-06 | 4.65E-06 |
| 21 | -2.18E-10 | 9.76E-07 | 1.75E-06 | 2.38E-06 | 2.88E-06 | 3.63E-06 | 4.23E-06 | 5.03E-06 | 4.79E-06 |
| 22 | -1.22E-09 | 9.02E-07 | 1.63E-06 | 2.24E-06 | 2.75E-06 | 3.56E-06 | 4.22E-06 | 5.05E-06 | 4.92E-06 |
| 23 | -1.51E-09 | 7.91E-07 | 1.49E-06 | 2.10E-06 | 2.63E-06 | 3.49E-06 | 4.17E-06 | 4.89E-06 | 4.96E-06 |
| 24 | -6.78E-10 | 6.93E-07 | 1.37E-06 | 1.99E-06 | 2.52E-06 | 3.39E-06 | 4.11E-06 | 4.98E-06 | 4.99E-06 |
| 25 | 1.71E-09 | 5.88E-07 | 1.23E-06 | 1.86E-06 | 2.42E-06 | 3.27E-06 | 4.02E-06 | 5.00E-06 | 5.03E-06 |
| 26 | 6.84E-11 | 4.88E-07 | 1.14E-06 | 1.77E-06 | 2.31E-06 | 3.18E-06 | 3.95E-06 | 4.93E-06 | 5.02E-06 |
| 27 | -1.87E-09 | 3.79E-07 | 1.05E-06 | 1.66E-06 | 2.19E-06 | 3.08E-06 | 3.83E-06 | 4.84E-06 | 4.97E-06 |
| 28 | -1.08E-09 | 2.61E-07 | 8.72E-07 | 1.53E-06 | 2.08E-06 | 2.95E-06 | 3.74E-06 | 4.76E-06 | 4.98E-06 |
| 29 | -4.45E-10 | 1.71E-07 | 8.74E-07 | 1.45E-06 | 1.95E-06 | 2.86E-06 | 3.63E-06 | 4.68E-06 | 4.92E-06 |
| 30 | 1.01E-09 | 2.21E-07 | 7.17E-07 | 1.30E-06 | 1.90E-06 | 2.98E-06 | 3.81E-06 | 4.57E-06 | 4.84E-06 |
| 31 | -1.57E-09 | 3.17E-07 | 7.15E-07 | 1.21E-06 | 1.76E-06 | 2.73E-06 | 3.48E-06 | 4.32E-06 | 4.66E-06 |
| 32 | -6.37E-10 | 2.26E-09 | 6.29E-07 | 1.17E-06 | 1.64E-06 | 2.53E-06 | 3.34E-06 | 4.30E-06 | 4.74E-06 |
| 33 | 1.90E-09 | 1.63E-08 | 5.52E-07 | 1.09E-06 | 1.55E-06 | 2.43E-06 | 3.22E-06 | 4.18E-06 | 4.67E-06 |
| 34 | -1.90E-09 | 9.13E-09 | 4.73E-07 | 1.02E-06 | 1.47E-06 | 2.33E-06 | 3.12E-06 | 4.05E-06 | 4.58E-06 |
| 35 | 6.43E-10 | 2.42E-08 | 3.90E-07 | 9.58E-07 | 1.39E-06 | 2.22E-06 | 3.00E-06 | 3.94E-06 | 4.49E-06 |
| 36 | 4.87E-10 | 5.25E-08 | 3.22E-07 | 8.80E-07 | 1.33E-06 | 2.09E-06 | 2.91E-06 | 3.83E-06 | 4.38E-06 |
| 37 | -1.89E-09 | 8.84E-08 | 3.11E-07 | 6.31E-07 | 1.05E-06 | 2.02E-06 | 2.76E-06 | 3.78E-06 | 4.41E-06 |
| 38 | 8.48E-10 | 9.21E-09 | 1.85E-07 | 7.27E-07 | 1.16E-06 | 1.90E-06 | 2.69E-06 | 3.64E-06 | 4.15E-06 |
| 39 | -2.46E-10 | 3.33E-10 | 1.12E-07 | 6.53E-07 | 1.09E-06 | 1.83E-06 | 2.58E-06 | 3.53E-06 | 4.18E-06 |
| 40 | -1.57E-09 | -3.03E-09 | 5.28E-08 | 5.96E-07 | 1.02E-06 | 1.76E-06 | 2.48E-06 | 3.43E-06 | 3.95E-06 |
| 41 | 6.46E-10 | 4.60E-09 | -3.89E-09 | 5.35E-07 | 9.48E-07 | 1.69E-06 | 2.39E-06 | 3.32E-06 | 3.84E-06 |
| 42 | 1.69E-09 | 7.53E-10 | 1.15E-08 | 4.63E-07 | 8.91E-07 | 1.61E-06 | 2.29E-06 | 3.22E-06 | 3.74E-06 |
| 43 | -1.38E-09 | 6.46E-09 | 1.22E-08 | 3.98E-07 | 8.37E-07 | 1.54E-06 | 2.21E-06 | 3.11E-06 | 3.64E-06 |
| 44 | 3.56E-10 | -7.74E-09 | -1.02E-08 | 3.56E-07 | 7.71E-07 | 1.50E-06 | 2.13E-06 | 3.02E-06 | 3.56E-06 |
| 45 | -1.34E-09 | 3.18E-09 | 1.45E-09 | 2.99E-07 | 7.18E-07 | 1.41E-06 | 2.07E-06 | 2.94E-06 | 3.43E-06 |
| 46 | -5.27E-10 | -8.87E-10 | 5.21E-09 | 2.55E-07 | 6.62E-07 | 1.35E-06 | 2.00E-06 | 2.85E-06 | 3.32E-06 |
| 47 | -5.48E-10 | -2.76E-09 | 7.62E-09 | 1.96E-07 | 6.07E-07 | 1.28E-06 | 1.93E-06 | 2.78E-06 | 3.28E-06 |
| 48 | -1.50E-11 | 7.25E-10 | 5.79E-09 | 1.49E-07 | 5.64E-07 | 1.23E-06 | 1.88E-06 | 2.67E-06 | 3.17E-06 |
| 49 | 1.21E-10 | 7.61E-09 | -1.57E-08 | 1.17E-07 | 4.98E-07 | 1.19E-06 | 1.76E-06 | 2.59E-06 | 3.16E-06 |
| 50 | 1.60E-09 | 6.39E-10 | 1.23E-10 | 3.76E-08 | 4.64E-07 | 1.12E-06 | 1.72E-06 | 2.51E-06 | 3.02E-06 |
| 51 | 1.19E-09 | 3.04E-08 | -2.13E-08 | 2.78E-08 | 4.08E-07 | 1.08E-06 | 1.61E-06 | 2.45E-06 | 2.96E-06 |
| 52 | -1.90E-09 | -2.02E-08 | -1.35E-08 | 2.71E-08 | 2.28E-07 | 1.02E-06 | 1.59E-06 | 2.36E-06 | 2.88E-06 |
| 53 | -8.87E-10 | -1.99E-08 | -3.08E-08 | 3.79E-08 | 2.26E-07 | 9.18E-07 | 1.54E-06 | 2.33E-06 | 2.79E-06 |
| 54 | -1.38E-09 | -2.14E-10 | -2.21E-08 | 4.43E-08 | 2.68E-07 | 9.04E-07 | 1.47E-06 | 2.24E-06 | 2.73E-06 |
| 55 | -1.31E-09 | -3.67E-09 | 9.84E-10 | -2.37E-09 | 2.10E-07 | 8.57E-07 | 1.45E-06 | 2.19E-06 | 2.67E-06 |
| 56 | -5.37E-10 | 3.21E-08 | -1.77E-08 | 1.52E-08 | 1.99E-07 | 7.85E-07 | 1.40E-06 | 2.23E-06 | 2.73E-06 |
| 57 | -1.71E-09 | 4.95E-09 | -6.40E-09 | 2.70E-09 | 1.43E-07 | 7.67E-07 | 1.33E-06 | 2.05E-06 | 2.54E-06 |
| 58 | 3.54E-12 | 2.10E-08 | -4.04E-10 | -6.90E-09 | 1.13E-07 | 7.23E-07 | 1.27E-06 | 1.94E-06 | 2.48E-06 |
| 59 | 8.38E-10 | -3.63E-09 | 1.80E-09 | 2.97E-10 | 7.06E-08 | 8.37E-07 | 2.21E-06 | 4.78E-06 | 6.46E-06 |
| 60 | -2.25E-10 | 1.44E-08 | 6.87E-09 | -2.07E-08 | 3.72E-08 | 9.27E-07 | 2.94E-06 | 7.03E-06 | 9.74E-06 |
| 61 | 1.33E-09 | 4.41E-09 | 4.56E-09 | -1.60E-08 | 1.80E-08 | 5.94E-07 | 1.10E-06 | 1.83E-06 | 2.35E-06 |
| 62 | 8.38E-10 | -1.78E-09 | 4.02E-09 | 6.19E-11 | -4.75E-10 | 5.88E-07 | 1.17E-06 | 1.91E-06 | 2.55E-06 |
| 63 | -1.13E-09 | -9.23E-10 | 2.67E-10 | -2.85E-09 | 1.95E-09 | 5.79E-07 | 1.01E-06 | 1.59E-06 | 2.06E-06 |
| 64 | 1.80E-09 | 2.88E-11 | -7.29E-10 | -1.34E-09 | 9.76E-10 | 5.05E-07 | 9.95E-07 | 1.52E-06 | 2.01E-06 |
| 65 | -1.95E-09 | -2.32E-10 | -6.26E-10 | -2.84E-10 | 1.33E-09 | 4.69E-07 | 9.59E-07 | 1.53E-06 | 1.99E-06 |
| 66 | 3.18E-10 | 2.69E-08 | -5.00E-09 | -2.67E-08 | 2.75E-08 | 3.91E-07 | 8.85E-07 | 1.49E-06 | 1.94E-06 |
| 67 | -8.11E-10 | -1.42E-09 | -1.25E-09 | -1.72E-10 | 3.67E-10 | 4.21E-07 | 8.33E-07 | 1.45E-06 | 1.91E-06 |
| 68 | 1.21E-09 | -5.56E-10 | -2.55E-09 | -3.64E-09 | -6.13E-10 | 4.78E-07 | 1.49E-06 | 3.21E-06 | 4.53E-06 |
| 69 | 1.81E-09 | -3.47E-10 | 4.89E-10 | -3.55E-09 | 1.63E-09 | 3.34E-07 | 7.89E-07 | 1.41E-06 | 1.73E-06 |
| 70 | -4.93E-10 | -3.31E-10 | 1.89E-09 | -3.42E-10 | -1.19E-09 | 3.23E-07 | 9.38E-07 | 1.81E-06 | 2.41E-06 |
| 71 | -1.32E-09 | 4.00E-10 | -1.11E-09 | -2.84E-09 | -1.94E-09 | 2.74E-07 | 7.35E-07 | 1.22E-06 | 1.44E-06 |
| 72 | -1.01E-09 | 2.42E-10 | 7.29E-10 | 5.52E-10 | -6.98E-10 | 2.49E-07 | 6.51E-07 | 1.19E-06 | 1.45E-06 |
| 73 | 1.84E-09 | 9.81E-10 | 2.35E-10 | -7.18E-09 | 1.78E-09 | 2.12E-07 | 6.24E-07 | 1.15E-06 | 1.41E-06 |
| 74 | -1.96E-09 | -7.60E-10 | 1.73E-09 | -1.41E-09 | 2.19E-09 | 1.83E-07 | 5.89E-07 | 1.11E-06 | 1.38E-06 |
| 75 | 7.63E-10 | 2.28E-10 | 3.29E-10 | -1.43E-09 | 1.59E-09 | 1.53E-07 | 5.63E-07 | 1.08E-06 | 1.34E-06 |
| 76 | -1.95E-09 | 1.57E-10 | 7.19E-10 | 9.41E-12 | 5.89E-10 | 1.23E-07 | 5.41E-07 | 1.06E-06 | 1.31E-06 |
| 77 | 7.79E-10 | 4.11E-10 | 5.74E-10 | -5.48E-10 | -5.07E-10 | 9.69E-08 | 5.17E-07 | 1.02E-06 | 1.27E-06 |
| 78 | 1.14E-09 | -8.06E-10 | -6.39E-10 | -3.65E-10 | 1.70E-09 | 7.24E-08 | 5.02E-07 | 9.91E-07 | 1.25E-06 |
| 79 | 2.43E-10 | 3.71E-10 | 2.80E-10 | 1.52E-10 | 8.23E-10 | 4.48E-08 | 4.79E-07 | 9.59E-07 | 1.22E-06 |
| 80 | -8.76E-10 | -1.21E-10 | 4.98E-10 | 1.80E-09 | -5.46E-10 | 1.66E-08 | 4.50E-07 | 9.23E-07 | 1.20E-06 |
| 81 | 1.22E-11 | 4.61E-11 | 8.12E-11 | 6.16E-11 | -1.06E-10 | 5.32E-10 | 4.29E-07 | 8.87E-07 | 1.16E-06 |
| 82 | -9.41E-11 | 5.45E-10 | 4.31E-10 | 1.63E-10 | -4.44E-10 | 2.14E-10 | 4.01E-07 | 8.57E-07 | 1.15E-06 |
| 83 | 1.06E-10 | 6.52E-11 | 1.40E-10 | 1.66E-10 | 3.46E-12 | 9.13E-10 | 3.80E-07 | 8.27E-07 | 1.12E-06 |
| 84 | -1.23E-09 | -1.76E-08 | -1.47E-08 | -7.78E-09 | 6.93E-09 | 8.91E-08 | 3.01E-07 | 8.12E-07 | 1.08E-06 |
| 85 | -9.90E-10 | 1.50E-10 | 8.35E-11 | -6.18E-11 | -2.70E-10 | 3.59E-10 | 3.33E-07 | 7.72E-07 | 1.06E-06 |
| 86 | -1.32E-09 | 4.71E-09 | 8.41E-09 | 7.96E-09 | 7.68E-09 | 5.41E-08 | 2.47E-07 | 7.77E-07 | 1.06E-06 |
| 87 | 1.13E-09 | 2.49E-11 | -5.78E-11 | -6.33E-11 | -2.96E-10 | 2.36E-10 | 2.86E-07 | 7.30E-07 | 9.97E-07 |
| 88 | 8.46E-10 | -1.27E-09 | -4.58E-09 | -1.38E-10 | -4.56E-10 | 7.18E-10 | 2.61E-07 | 7.09E-07 | 9.84E-07 |
| 89 | -1.45E-09 | -8.23E-12 | -7.34E-11 | -5.85E-11 | 1.83E-11 | 2.34E-10 | 2.37E-07 | 6.91E-07 | 9.69E-07 |
| 90 | -9.07E-10 | -2.52E-10 | -3.69E-10 | -2.03E-10 | -6.27E-11 | -2.02E-10 | 2.13E-07 | 6.72E-07 | 9.46E-07 |
| 91 | -1.28E-09 | -3.31E-10 | -1.10E-09 | -1.95E-09 | -2.58E-09 | 3.95E-09 | 1.94E-07 | 6.52E-07 | 9.21E-07 |
| 92 | 1.78E-09 | 2.23E-11 | 7.37E-12 | 2.87E-12 | -1.91E-11 | -2.80E-11 | 1.71E-07 | 6.30E-07 | 9.00E-07 |
| 93 | 3.28E-10 | 1.98E-10 | 1.80E-10 | -1.89E-11 | -9.75E-11 | -3.57E-11 | 1.52E-07 | 6.09E-07 | 8.76E-07 |
| 94 | -1.42E-09 | -6.76E-11 | 3.51E-10 | 7.38E-10 | 2.31E-10 | 2.85E-10 | 1.34E-07 | 5.89E-07 | 8.49E-07 |
| 95 | -3.18E-10 | 1.49E-10 | -1.57E-11 | 1.23E-10 | 1.03E-10 | 7.47E-11 | 1.14E-07 | 5.67E-07 | 8.20E-07 |
| 96 | 1.36E-09 | -4.41E-09 | -4.04E-09 | -7.41E-10 | 4.63E-10 | 6.23E-09 | 9.40E-08 | 5.43E-07 | 8.05E-07 |
| 97 | -1.78E-09 | -2.45E-10 | -1.21E-10 | -1.00E-10 | -5.41E-10 | 9.04E-10 | 7.24E-08 | 5.21E-07 | 7.67E-07 |
| 98 | -7.68E-10 | 2.60E-11 | 1.22E-10 | 1.22E-10 | -1.22E-10 | 1.20E-10 | 5.12E-08 | 4.96E-07 | 7.45E-07 |
| 99 | 2.38E-10 | 4.56E-12 | -9.89E-13 | -4.69E-11 | -1.65E-10 | -6.39E-11 | 3.01E-08 | 4.76E-07 | 7.24E-07 |
| 100 | -1.60E-09 | 1.99E-11 | 2.49E-11 | 2.80E-11 | -8.03E-11 | 1.37E-11 | 1.19E-08 | 4.58E-07 | 7.08E-07 |
| 101 | -7.94E-10 | 3.78E-10 | 1.74E-10 | 1.19E-10 | -1.09E-10 | -2.24E-09 | -4.97E-09 | 4.45E-07 | 6.96E-07 |
| 102 | -9.69E-10 | -4.44E-12 | -8.41E-12 | -7.26E-12 | 6.32E-12 | 7.43E-12 | 5.19E-11 | 4.30E-07 | 6.75E-07 |
| 103 | 2.83E-10 | -7.88E-11 | -2.51E-11 | 2.50E-11 | 6.18E-11 | 3.43E-11 | 1.40E-10 | 4.12E-07 | 6.56E-07 |
| 104 | 1.61E-09 | 1.22E-11 | 1.87E-11 | 1.78E-11 | 1.07E-11 | -2.25E-11 | 4.85E-11 | 3.97E-07 | 6.39E-07 |
| 105 | 1.16E-09 | -4.73E-11 | -2.30E-11 | 2.43E-11 | 5.87E-11 | -1.36E-11 | 1.10E-11 | 3.77E-07 | 6.18E-07 |
| 106 | 1.84E-09 | 5.26E-11 | 2.29E-12 | -3.47E-11 | -6.05E-11 | -8.17E-11 | 2.57E-10 | 3.56E-07 | 6.00E-07 |
| 107 | 8.37E-10 | 6.78E-12 | 2.04E-11 | 3.41E-11 | 4.08E-11 | -1.89E-11 | 3.28E-10 | 3.37E-07 | 5.82E-07 |
| 108 | 7.69E-11 | -7.95E-12 | -9.79E-12 | -1.12E-11 | -1.28E-11 | -2.15E-11 | 1.42E-10 | 3.23E-07 | 5.66E-07 |
| 109 | -1.27E-09 | 1.41E-11 | 1.65E-10 | 2.47E-10 | 2.77E-10 | 1.05E-10 | -2.38E-10 | 3.07E-07 | 5.50E-07 |
| 110 | 6.41E-10 | 1.65E-12 | 1.63E-12 | 1.91E-12 | 1.76E-12 | -3.22E-11 | 2.75E-10 | 2.96E-07 | 5.38E-07 |
| 111 | 2.00E-09 | 3.65E-12 | -2.50E-12 | 3.91E-12 | 1.88E-11 | -1.06E-11 | 1.83E-12 | 2.83E-07 | 5.23E-07 |
| 112 | -1.61E-09 | 1.59E-11 | 1.08E-11 | 2.31E-12 | -1.14E-11 | -5.23E-11 | 2.04E-10 | 2.68E-07 | 5.10E-07 |
| 113 | 1.28E-09 | -2.30E-10 | -2.57E-10 | -3.19E-10 | -4.50E-10 | -9.27E-10 | 2.45E-10 | 2.49E-07 | 4.99E-07 |
| 114 | 1.68E-10 | -9.01E-12 | 2.66E-12 | 5.36E-13 | -2.98E-11 | -3.19E-10 | -1.12E-09 | 2.26E-07 | 4.73E-07 |
| 115 | -1.49E-10 | -1.65E-11 | -5.68E-11 | -3.85E-11 | -1.51E-11 | 2.96E-11 | -6.93E-11 | 2.08E-07 | 4.54E-07 |
| 116 | 5.63E-10 | 1.85E-11 | 3.51E-11 | 3.20E-11 | 2.38E-11 | -1.45E-11 | 8.96E-12 | 1.88E-07 | 4.34E-07 |
| 117 | 8.52E-11 | -1.49E-10 | -2.53E-10 | 1.83E-11 | 5.16E-10 | 2.14E-11 | 1.11E-09 | 1.71E-07 | 4.14E-07 |
| 118 | 1.85E-09 | -2.13E-11 | 2.14E-11 | 7.26E-11 | 1.12E-10 | -9.17E-12 | -1.73E-10 | 1.57E-07 | 3.96E-07 |
| 119 | 1.56E-09 | -2.01E-11 | -1.36E-11 | 2.41E-11 | 7.42E-11 | 3.94E-11 | -8.80E-12 | 1.45E-07 | 3.79E-07 |
| 120 | 2.77E-10 | 2.36E-11 | 2.12E-11 | 2.07E-11 | 2.12E-11 | 9.89E-12 | 1.99E-11 | 1.34E-07 | 3.64E-07 |
| 121 | 1.69E-11 | -4.26E-11 | -5.77E-11 | -2.03E-11 | 1.04E-10 | 1.54E-10 | 3.23E-10 | 1.22E-07 | 3.51E-07 |
| 122 | -1.06E-09 | -1.32E-10 | -3.66E-10 | -4.69E-10 | -2.59E-10 | 8.71E-11 | 9.54E-11 | 1.09E-07 | 3.38E-07 |
| 123 | -7.87E-10 | -9.63E-11 | -2.93E-11 | 3.05E-11 | 7.32E-11 | -2.89E-11 | 3.35E-11 | 9.58E-08 | 3.26E-07 |
| 124 | -1.75E-10 | -3.81E-12 | -1.98E-11 | -2.85E-11 | -6.30E-11 | -3.63E-10 | -4.32E-11 | 8.20E-08 | 3.14E-07 |
| 125 | -1.04E-09 | 4.01E-12 | 4.80E-12 | -1.23E-12 | -2.06E-11 | -1.31E-10 | 2.51E-10 | 6.82E-08 | 3.01E-07 |
| 126 | 7.33E-12 | -7.92E-12 | 3.77E-13 | 1.26E-11 | 2.54E-11 | -2.20E-11 | -2.24E-11 | 5.46E-08 | 2.88E-07 |
| 127 | -1.86E-09 | -2.61E-11 | -2.12E-11 | -4.43E-12 | 2.53E-11 | 5.90E-11 | 9.82E-11 | 4.24E-08 | 2.74E-07 |
| 128 | 1.67E-09 | -3.11E-11 | -3.16E-11 | -1.04E-11 | 2.50E-11 | 2.26E-11 | 7.73E-11 | 2.99E-08 | 2.59E-07 |
| 129 | 1.28E-09 | 6.34E-12 | -2.44E-12 | -1.67E-11 | -3.68E-11 | -8.92E-11 | 2.07E-11 | 1.90E-08 | 2.44E-07 |
| 130 | -5.13E-10 | 2.82E-11 | 2.50E-11 | 3.55E-12 | -9.51E-12 | -1.25E-12 | 5.59E-11 | 9.08E-09 | 2.29E-07 |
| 131 | -3.06E-10 | 2.24E-12 | -6.65E-12 | -2.06E-11 | -4.96E-11 | -4.03E-10 | -2.90E-11 | 1.98E-09 | 2.14E-07 |
| 132 | 1.92E-09 | 3.07E-11 | 2.57E-11 | 1.95E-11 | 1.15E-11 | -1.25E-11 | -4.98E-11 | 2.08E-10 | 2.00E-07 |
| 133 | -1.41E-10 | -5.61E-12 | 1.74E-13 | 5.45E-12 | 8.91E-12 | 1.24E-11 | -1.23E-11 | 2.24E-10 | 1.89E-07 |
| 134 | -1.80E-09 | 1.15E-12 | 1.24E-12 | 1.18E-12 | 9.61E-13 | 1.65E-12 | -5.89E-12 | 1.63E-12 | 1.75E-07 |
| 135 | -1.11E-09 | 1.28E-11 | 1.14E-11 | 1.16E-11 | 1.49E-11 | 5.26E-11 | 7.37E-11 | 3.38E-10 | 1.64E-07 |
| 136 | -3.60E-10 | 1.35E-11 | 1.03E-11 | 6.25E-12 | 1.52E-12 | -5.06E-12 | -7.25E-12 | 4.82E-10 | 1.53E-07 |
| 137 | -1.93E-09 | -3.80E-11 | 3.45E-11 | 8.78E-11 | 1.13E-10 | 3.62E-11 | -1.88E-10 | -2.08E-11 | 1.41E-07 |
| 138 | 1.40E-09 | -1.75E-12 | -2.57E-12 | -4.61E-12 | -7.57E-12 | -1.56E-11 | -4.18E-11 | -1.30E-10 | 1.30E-07 |
| 139 | -9.18E-10 | -2.79E-12 | -2.67E-12 | -1.80E-12 | -1.41E-12 | -3.87E-12 | -1.23E-11 | 5.31E-12 | 1.18E-07 |
| 140 | -1.46E-09 | -8.62E-13 | 1.28E-13 | 7.93E-13 | 1.15E-12 | 5.88E-13 | -5.46E-12 | -1.15E-11 | 1.07E-07 |
| 141 | 1.52E-09 | -6.70E-13 | -1.78E-12 | -2.77E-12 | -3.26E-12 | -9.67E-13 | 1.32E-13 | 5.54E-12 | 9.59E-08 |
| 142 | -6.55E-10 | -5.11E-13 | -9.17E-13 | -8.91E-13 | -5.25E-13 | 2.68E-12 | 1.69E-12 | 4.65E-12 | 8.56E-08 |
| 143 | 4.48E-10 | 1.93E-12 | 1.41E-12 | 6.35E-13 | -4.40E-13 | -4.03E-12 | -1.33E-11 | 8.66E-11 | 7.58E-08 |
| 144 | -1.90E-09 | 6.29E-12 | 7.95E-12 | 7.36E-12 | 6.24E-12 | 9.40E-12 | -9.01E-12 | 2.62E-11 | 6.63E-08 |
| 145 | 1.21E-09 | 1.68E-12 | 1.19E-12 | 8.01E-13 | 2.34E-13 | -5.00E-12 | -2.20E-11 | 4.26E-12 | 5.62E-08 |
| 146 | 4.45E-10 | -1.05E-12 | -6.88E-13 | -3.87E-13 | -1.37E-13 | 2.26E-13 | -5.40E-12 | 1.36E-11 | 4.71E-08 |
| 147 | -7.72E-10 | 5.67E-12 | 6.17E-12 | 6.09E-12 | 4.88E-12 | -1.13E-11 | -1.38E-10 | 4.89E-11 | 3.70E-08 |
| 148 | -1.66E-09 | -1.02E-13 | 8.83E-14 | 4.06E-13 | 8.31E-13 | 1.44E-12 | -9.12E-13 | 5.12E-12 | 2.63E-08 |
| 149 | -1.83E-09 | 1.20E-12 | 1.07E-12 | 4.33E-13 | -4.64E-13 | -2.77E-12 | -5.91E-12 | 7.04E-12 | 1.55E-08 |
| 150 | 1.46E-09 | 8.92E-14 | 1.53E-13 | 1.82E-13 | 1.41E-13 | -2.41E-13 | -1.41E-12 | -1.09E-13 | 3.67E-09 |
